# Pseudo-Complementary G:C Base Pair for Mixed Sequence dsDNA Invasion and Its Applications in Diagnostics (SARS-CoV-2 Detection)

**DOI:** 10.1021/jacsau.2c00588

**Published:** 2023-02-01

**Authors:** Miguel López-Tena, Lluc Farrera-Soler, Sofia Barluenga, Nicolas Winssinger

**Affiliations:** Department of Organic Chemistry, NCCR Chemical Biology, Faculty of Science, University of Geneva, 1211 Geneva, Switzerland

**Keywords:** PNA, artificial nucleobases, pseudo-complementarity, RPA

## Abstract

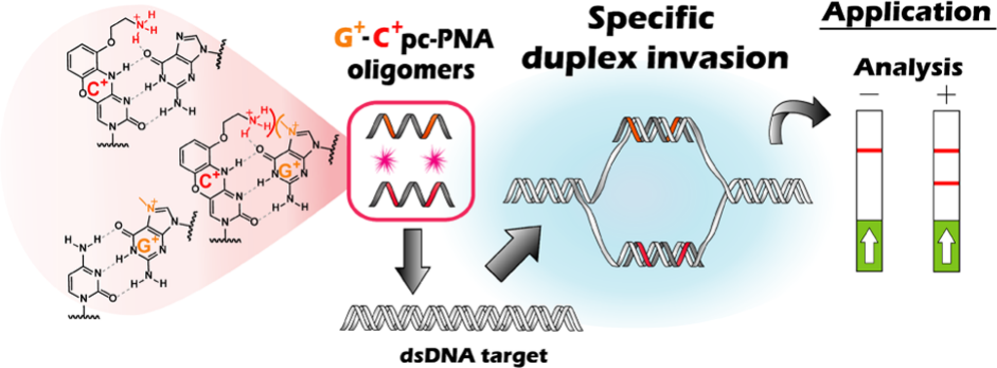

Pseudo-complementary oligonucleotides contain artificial nucleobases designed to reduce duplex formation in the pseudo-complementary pair without compromising duplex formation to targeted (complementary) oligomers. The development of a pseudo-complementary A:T base pair, U^s^:D, was important in achieving dsDNA invasion. Herein, we report pseudo-complementary analogues of the G:C base pair leveraged on steric and electrostatic repulsion between the cationic phenoxazine analogue of cytosine (G-clamp, C^+^) and N-7 methyl guanine (G^+^), which is also cationic. We show that while complementary peptide nucleic acids (PNA) form a much more stable homoduplex than the PNA:DNA heteroduplex, oligomers based on pseudo-C:G complementary PNA favor PNA:DNA hybridization. We show that this enables dsDNA invasion at physiological salt concentration and that stable invasion complexes are obtained with low equivalents of PNAs (2–4 equiv). We harnessed the high yield of dsDNA invasion for the detection of RT-RPA amplicon using a lateral flow assay (LFA) and showed that two strains of SARS-CoV-2 can be discriminated owing to single nucleotide resolution.

## Introduction

**Figure 1:**
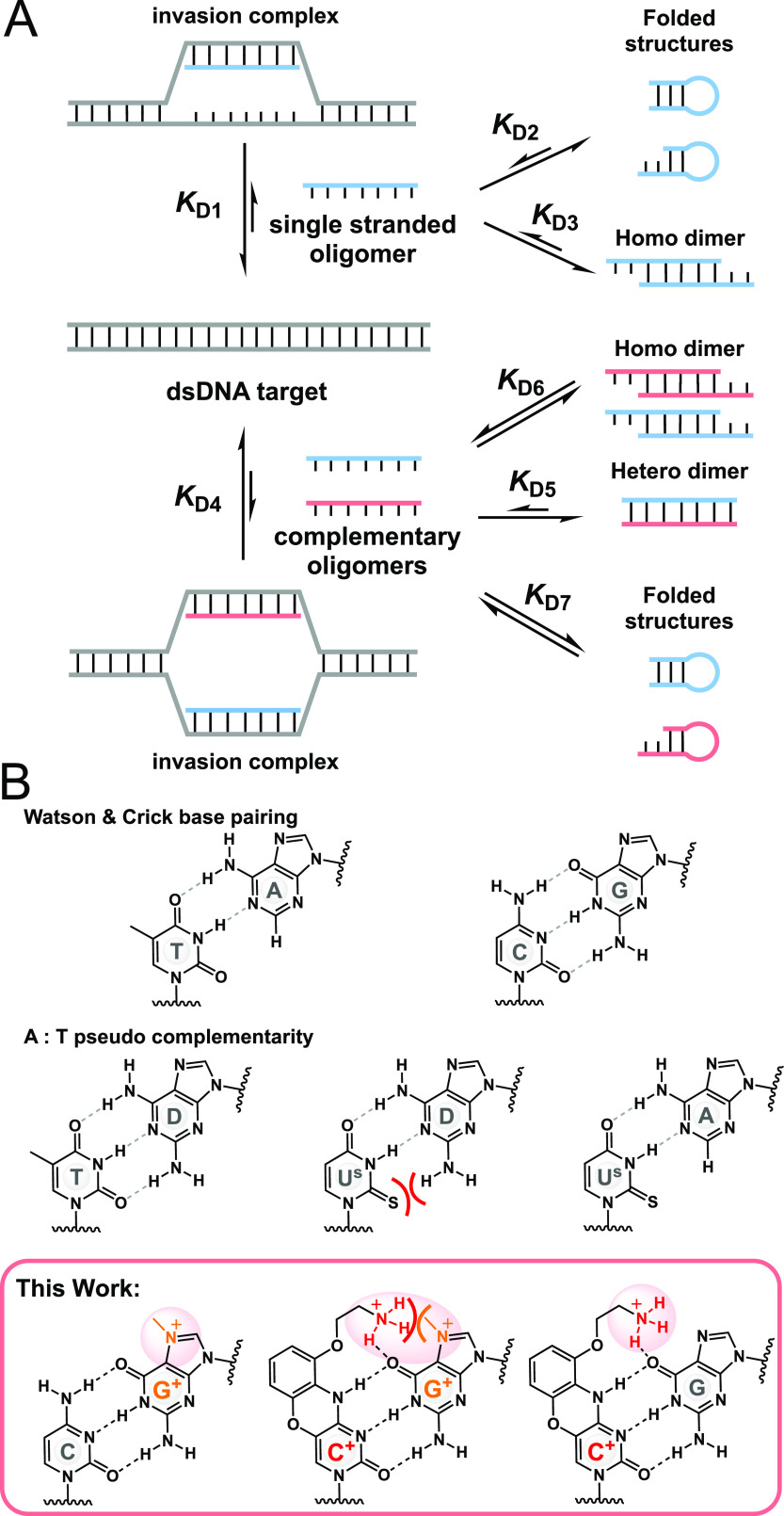
(A) Invasion of dsDNA through Watson and Crick pairing. (B) Pseudo-complementary nucleobases.

Given the double-stranded nature of genomic DNA, achieving stable invasion of dsDNA has inspired numerous developments. While antigene therapeutic^1−4^ and genetic manipulation technologies^5,6^ were amongst the first impetus, the scope of double-strand invasion technologies extends far beyond these early applications, and invasion into folded noncoding RNA targets,^7^ the manipulation of nanoscale DNA architectures, or DNA circuitry are also important.^8−13^ Invasion through Watson and Crick interactions with a single strand is very challenging since it must overcome the competing interaction of the complementary strand present at high effective concentrations, akin to reversing a toehold displacement (Figure 1A, *K*_D1_). Peptide nucleic acids (PNAs), first reported over 30 years ago,^14−16^ have attracted significant attention owing to the unique and desirable properties of this synthetic oligonucleotide mimic.^17^ Notably, PNAs form a more stable duplex with DNA or RNA than the respective DNA or RNA homoduplex. This is largely due to the lack of repulsive interaction in PNA:DNA or PNA:RNA duplexes relative to the DNA or RNA homoduplex owing to the neutral charge of the PNA backbone. Accordingly, this behavior is accentuated at low salt concentrations, which exacerbates the repulsive interactions of anionic charges in the DNA or RNA homoduplex.^18^ It was quickly recognized that the higher thermal stability of a PNA:DNA duplex relative to a DNA homoduplex could be harnessed for dsDNA invasion. However, achieving a stable invasion complex by Watson and Crick pairing with PNA requires binding to both strands of dsDNA and must overcome the higher PNA homoduplex stability (Figure 1, *K*_D_4 < *K*_D_5). A first solution was reported by Nielsen and co-workers using pseudo-complementary A:T analogues (diaminopurine (D) and 2-thiouracil (U^s^),^19^Figure 1B).^20,21^ This solution has been embraced with notable applications in gene correction^22^ and artificial cutters.^23−26^ It was shown to be further improved using a positively charged PNA backbone, further offsetting the homodimerization of PNAs.^27^ However, while the steric clash of 2-thiouracil with diaminopurine is detrimental to a PNA duplex, the stability of U^s^-PNA:D-PNA remains higher than that for U^s^-PNA:DNA. An elegant solution is to conjugate such PNA to a polyamide that binds in the minor groove of DNA and creates a highly effective concentration of the PNA.^28^ However, reaching the full potential of double-strand invasion would benefit from further improvements in pseudo-complementarity. Early efforts to create a pseudo-complementary G–C succeeded in suppressing the G–C binding of pseudo-complementary bases, but at the cost of overall affinity for target DNA.^29^ Backbone modifications,^30−32^ in particular γ-modified PNAs (γ-PNA) have proven to bring important benefits in preorganizing the otherwise flexible backbone of PNA into the DNA-binding conformation and to enhance the solubility of PNA.^33,34^ This modification was shown to bring significant improvements in dsDNA invasion using a single γ-PNA strand,^35−37^ but studies were performed at a low salt concentration (10 mM NaPi). This modification has been embraced in a number of applications including regulation of gene expression, imaging of cellular RNA, and gene editing.^17,38−41^ Most recently, it was shown to enable double-stranded DNA nicking by DNAzymes with higher sequence fidelity than CRISPR/Cas.^42^ Herein, we report the design of a new pseudo-complementarity G:C-based pair leveraged on N7-Me-G^43^ and G-clamp^44,45^ (Figure 1B).

## Results and Discussion

**Figure 2:**
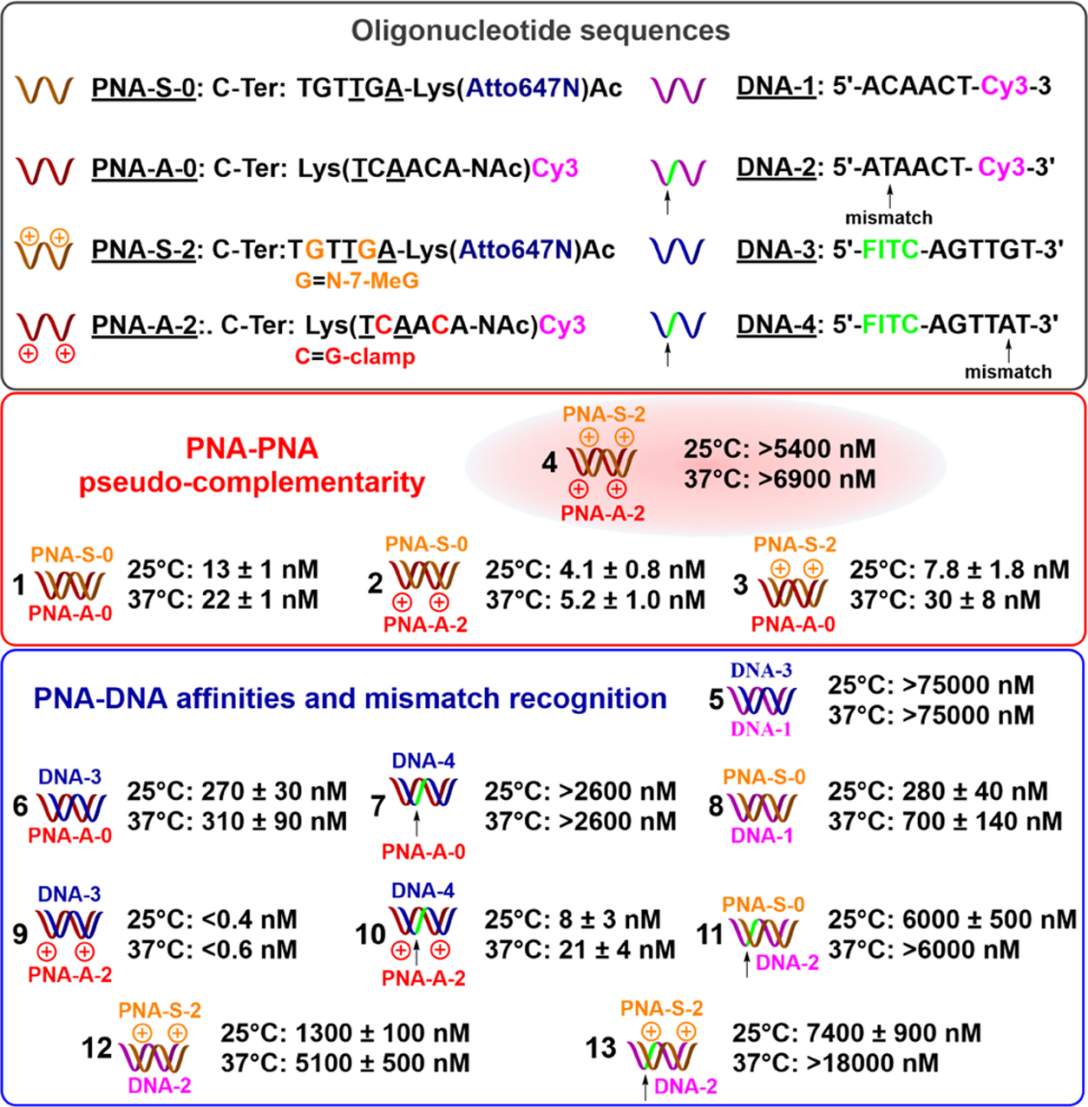
Measured *K*_D_ values for PNA:PNA and PNA:DNA duplexes at 25 and 37 °C in simulated physiological salt conditions (PS - 20 mM Tris, pH 7.5 containing KCl: 140 mM; NaCl 20 mM; CaCl_2_: 0.1 mM; MgSO_4_: 1 mM; 0,02% Tween-20).

N7 methylation of guanine (G^+^, Figure 1B) is a natural post-transcriptional modification found in mRNA, rRNA, tRNA, and miRNA.^46^ It was recently reported by Shoji and co-workers that the incorporation of N7-G in a PNA oligomer afforded a more stable PNA–DNA duplex at low salt concentrations while destabilizing the PNA–PNA duplex when in close proximity due to electrostatic repulsion.^43^ However, these benefits were reduced at higher NaCl concentrations (100 mM). Inspired by this advance, we reasoned that this effect could be enhanced in a pseudo-complementary nucleobase using a G-clamp^44^ (C^+^, Figure 1B) that would position the positive charge adjacent to the cationic N7-G and add steric congestion to an unfavorable charge repulsion (Figure 1B). Based on this hypothesis, we set out to evaluate the *K*_D_ of PNAs as homoduplexes and PNA:DNA duplexes using simulated physiological salt concentrations (20 mM Tris, pH 7.5 containing the following cations, K^+^: 140 mM; Na^+^ 20 mM; Ca^2+^: 0.1 mM; Mg^2+^: 1 mM) with a FRET-based assay^47^ (see Figure S1 for a detailed molecular structure of PNAs). As shown in Figure 2, the 6-mer γ-PNA duplex containing no pseudo-complementary (pc) modification had a *K*_D_ of 13 and 22 nM at 25 and 37 °C, respectively (entry 1). Introduction of either pc-modification, the G-clamp (C^+^) on one side or N7-Me-G (G^+^) on the other side had little impact on the *K*_D_ of the PNA duplex (entry 2 and 3); however, the PNA duplex containing the pc-modifications on both sides showed a dramatic loss of affinity (>1000-fold) compared to the unmodified duplex (entry 1 vs 4). Comparing the affinities of the different PNAs to DNA, the G-clamp (C^+^) yielded a very important gain of affinity relative to unmodified PNA (>500 fold, entry 9 vs 6) while the N7-Me-G (G^+^) showed moderate loss affinity compared to the PNA without modified nucleobases (5–7 fold, entry 12 vs 8). Excellent sequence-specific hybridizations were maintained in the modified PNAs, with greater than 10-fold selectivity for perfect match oligomer vs single nucleotide mismatches (entries 6, 9, 12 vs 7, 10, 13, respectively). The most important consideration to achieve thermodynamically favorable dsDNA invasion is that the interaction of each pc-PNA strand is stronger for DNA than the complementary PNA (*K*_D4_ < *K*_D5_) or DNA:DNA duplex. The data clearly shows that this is not the case for PNA devoid of pseudo-complementarity, wherein the PNA homoduplex is far more stable than either PNA–DNA duplexes (entry 1 vs 6 and 8). However, it is clearly the case with the pc-bases reported herein (entry 4 vs 9 and 12). The data shown in Figure 2 contains two pc-modifications within a 6-mer PNA, but the same trend was observed for a single pc-modification in the same 6-mer sequence (Figure S2). However, a single modification was not sufficient to yield higher stability in the PNA–DNA duplex vs the PNA homoduplex. Collectively, the data support the fact that the N7-G:G-clamp is an effective pc-base pair.

**Figure 3:**
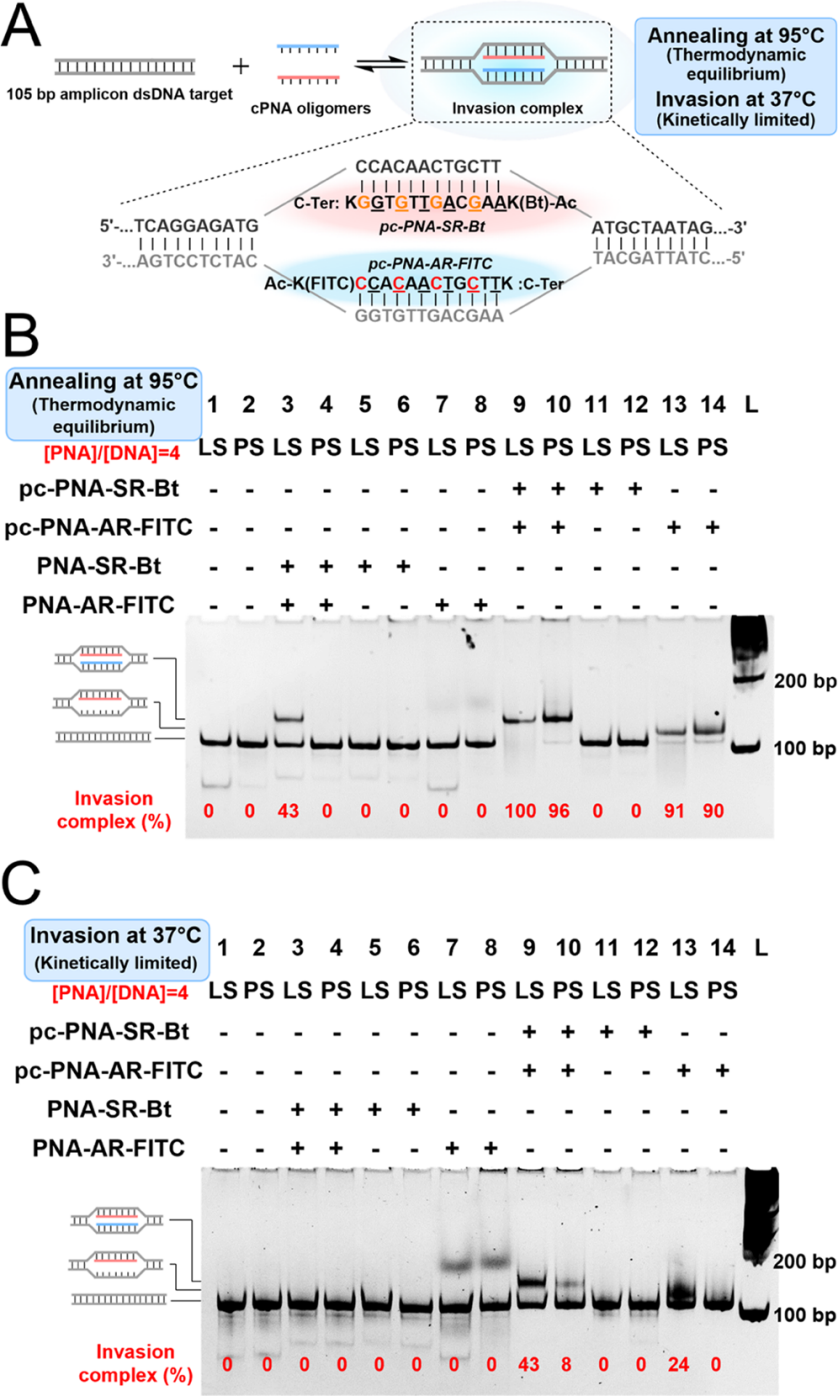
(A) Invasion scheme of pc-PNA oligomers into the 105 bp dsDNA target amplicon from SARS-CoV-2, forming the desired invasion complex. For PNA-SR-Bt-c and PNA-AR-FITC-c, C^+^ and G^+^ were replaced by native nucleobases C and G. (B) Invasion of regular c-PNAs vs pc-PNAs at low salt buffer conditions (LS) or simulated physiological salt conditions (PS) after annealing at 95 °C for 5 min. (C) Invasion of regular c-PNAs vs new pc-PNAs at low salt buffer conditions (LS - 20 mM Tris, pH 7.5, 0,02% Tween-20) or simulated physiological salt conditions (PS - 20 mM Tris, pH 7.5 containing KCl: 140 mM; NaCl_2_ 0 mM; CaCl_2_: 0.1 mM; MgSO_4_: 1 mM; 0,02% Tween-20) for 16 h at 37 °C. Quantification was performed by integration of the band signal using ImageJ software. Underlined residues denote a γ-modified backbone. Colored residues denote a PC nucleobase.

We next turned our attention to dsDNA invasion. To this end, we used a 105 nt amplicon^48^ from SARS-CoV-2, mindful that rapid and efficient dsDNA invasion could have applications in detecting such amplicon in rapid genetic tests (*vide infra*). While the invasion of dsDNA with PNA has been demonstrated, it typically requires low salt concentrations or large excess of the PNA probes. With the intention of performing the invasion at low target concentrations, we used longer PNA probes (12-mer) than the ones used in our *K*_D_ measurements (6-mer); however, maintaining the same ratio of modifications (one pc-modification out of three residues, one γ modification every other residue; see Figure S1 for explicit structures). We studied the invasion under two conditions: heating to 95 °C for 5 min and cooling to room temperature; at 37 °C, evaluating the invasion over a time course of 16 h using 1–4 equiv of PNAs (Figure 3A). The results from the annealing should reflect the thermodynamic equilibrium between dsDNA and the invasion complex, while the incubation at 37 °C will be limited by the kinetics of dsDNA invasion. As can be seen in Figure 3B, following the annealing cycle, the PNAs devoid of pc-modifications showed partial invasion (43%) under low salt (LS) conditions but no invasion under simulated physiological salt (PS) conditions (lanes 3 and 4, respectively), consistent with the *K*_D_ measurements showing a higher affinity for the PNA:PNA duplex vs PNA:DNA duplex. However, the pc-PNA showed excellent invasion under either condition (100% for LS and 96% for PS). The sequence selected positioned all G^+^ on one PNA strand targeting the sense strand of the amplicon, whereas all of the C^+^ were positioned on the antisense strand. Comparison between lanes 11 and 13 or 12 and 14 clearly shows that the C^+^-containing PNA contributes to a larger degree to dsDNA invasion. This observation is consistent with the *K*_D_ measurements shown in Figure 2. At 37 °C (Figure 3C), the PNA devoid of pc-modification showed no detectable invasion complex (lanes 3 and 4), while the pc-PNA showed partial invasion (lanes 9 and 10). As can be anticipated, the invasion was faster under LS conditions (43%, lane 9) than under PS conditions (8%, lane 10). Nonetheless, the fact that dsDNA invasion was observed under physiologically relevant temperature and salt concentrations is notable.

The gel shift assay reproducibly showed a fainter band running at nearly 200 bp (lanes 7 and 8) for PNA-AR, which was not observed with the equivalent PNA sequence containing pc-modifications, nor was it observed when this PNA-AR was used in conjunction with the complementary PNA (PNA-SR), leading to the speculation that it is the product of sequence-specific aggregation. Experiments with lower equivalents of pc-PNA still afforded dsDNA invasion (4 and 23% at 1 and 2 equiv of PNA, respectively, Figure S3A). Comparison of invasion at 30 min, 2 and 16 h shows a progression of dsDNA invasion from 19 to 36 and 58%, respectively, suggesting that the invasion progressed over time but is kinetically slow (Figure S3B). Increasing the concentrations of salts (MgSO_4_, KCl, CaCl_2_) slowed the kinetics of invasion, but invasion was still observed with 2 mM MgSO_4_, 140 mM KCl, 20 mM NaCl, and 0.1 mM CaCl_2_ (8% after 16 h at 37 °C, Figure S3B). The dsDNA invasion performances observed in PS conditions were also observed in high NaCl solutions (140 mM NaCl, 20 mM Tris, pH = 7.5, Figure S4). It should be noted that the quantification by SYBR gold staining of gel slightly underestimates the amount of the invasion complex since it will stain less intensely than dsDNA due to the lower intercalation of SYBR gold in the PNA:DNA stretch (see Figure S5 for comparison of quantification using a Cy3-labeled amplicon and quantification of Cy3 vs SYBR Gold). To drive the dsDNA invasion further, we tested higher equivalents of PNA (4, 7, 10 equiv) and higher temperatures (37 vs 50 °C). The results showed a progressively faster reaction at higher equivalence owing to the higher concentration of PNAs and faster reactions at 50 °C (Figure S6), consistent with the dsDNA invasion being kinetically limited. The invasion complex, once formed, was found to be kinetically stable for at least 12 h, even at simulated physiological salt concentrations. Using a pseudo-first-order approximation (10 equiv of PNAs), the half-life of the reaction was ca. 8 h at 37 °C and ca. 3 h 50 °C. While this is orders of magnitude lower than direct hybridization, it is consistent with strand-displacement kinetics.^49^

**Figure 4:**
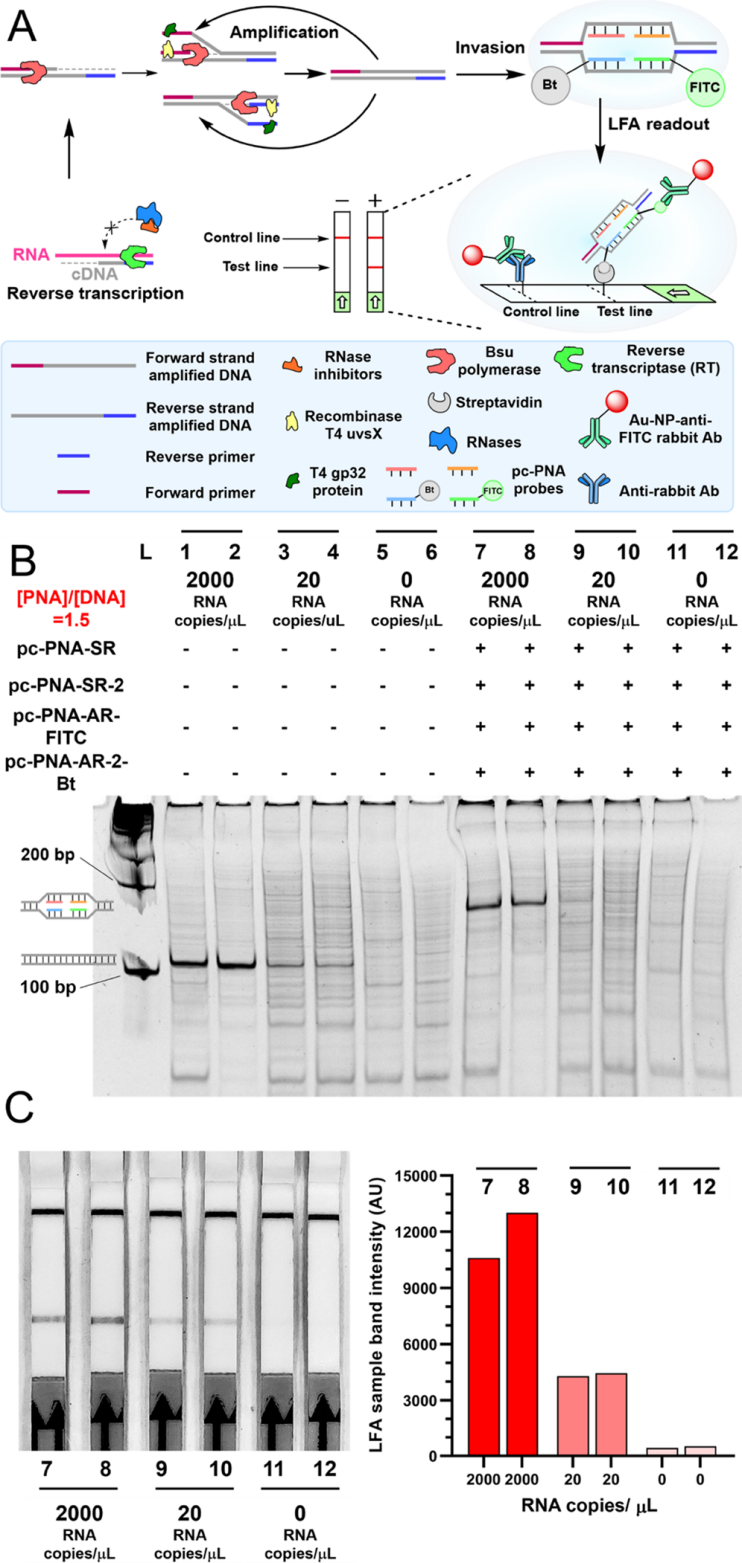
(A) Schematic protocol for the detection of desired RNA by RT-RPA amplification coupled to pc-PNAs invasion and LFA readout. (B) Native-PAGE gel analysis of analytical duplicates after RT-RPA amplification from the SARS-CoV-2 Wuhan-Hu-1 RNA template and invasion with pc-PNA probes. Lanes 1 to 6, products of RT-RPA as analytical duplicate pairs. Lanes 7 to 12, invasion after RT-RPA input from lanes 1 to 6. (C) LFA readout of analytical duplicates after RT-RPA amplification from the SARS-CoV-2 Wuhan-Hu-1 RNA template and invasion with pc-PNA probes. LFA test band intensity quantification. Lanes referred to samples at (4B). LFA band intensities were quantified using ImageJ software.

We next investigated the possibility of harnessing the high yield of dsDNA invasion following a simple annealing step for sequence-specific amplicon detection. We reasoned that the design could be further improved using two pairs of pc-probes. This would increase the genetic sequence being interrogated from 12nt to 24nt without compromising the penalty of mismatch hybridization (a single 24nt probe would be less sensitive to a single mismatch). Furthermore, it removes the risk of false positives from weak interactions from the pc-probes. Recombinase polymerase amplification (RPA), or RT-RPA, are well-established instrument-free isothermal amplification methods that conveniently proceed at 37–40 °C, which is desirable for point-of-care diagnostics.^50,51^ Accordingly, RPA has been embraced in efforts to develop rapid nucleic acid sensing technologies, particularly for SARS-CoV2.^48,52−57^ One challenge with RPA is the byproducts arising from amplification of transient interactions of primers or partially match contaminants yielding a ladder of random polymerization products. Thus, genetic tests using RPA greatly benefit from methods that provide a sequence-specific readout of the amplicon rather than simply quantifying the level of polymerization achieved, as is frequently done in RT-PCR. Indeed, the RPA-based test developed for SARS-CoV-2 use either a CRISPR-Cas system to provide a sequence-specific signal,^54−56^ a split DNAzyme that is turned on by sequence-specific hybridization^57,58^ or a templated reaction.^48^ We reasoned that the RPA amplicon could be captured and detected by invasion with a biotin-functionalized pc-PNA pair and a second FITC-labeled pc-PNA pair for detection by lateral flow without recourse to further manipulation (Figure 4A, see Figure S7 for sequences of PNA). We had previously reported the use of RPA with a templated reaction^48^ based on the sequence recommended by WHO for RT-PCR detection at the onset of the SARS-CoV2 outbreak.^59^ The RT-RPA afforded a clear 105-nt amplicon with 2000 copies of input RNA (lanes 1 and 2, Figure 4B); 20 copies of input RNA still yielded an amplicon detectable by gel (lanes 3 and 4, Figure 4B), but no single amplicon was observed in the control without input RNA (lane 5 and 6, Figure 4B). In the absence of input RNA, a ladder of unspecific amplicons was observed. The addition of the pairs of pc-PNA with a 5 min annealing using the crude RPA mixture produced a quantitative invasion complex (Figure 4B, lanes 7 and 8). The annealing step serves two purposes, it denatures the proteins involved in the RPA, thus stopping the amplification, and it provides the conditions for fast dsDNA invasion. The invasion complex could be observed in an RT-RPA sample prepared from 20 copies of viral RNA input, albeit the sample is highly contaminated by side products from the RPA (Figure 4B, lanes 9 and 10). The negative control with no viral RNA input yielded a ladder of amplification products, which highlights the fact that the RPA-based test greatly benefits from a sequence-selective readout relative to a simple quantification of the amplification products. Experiments performed with substoichiometric quantities of pc-PNA pairs provided a mixture of single invasion complexes and double invasion complexes (Figure S7B). Notably, the PNA containing the pc-modification significantly outperformed the control PNA, yielding >10-fold more invasion complexes (Figure S7, lane 3 vs 10 or lane 6 vs 13). During the optimization of the lateral flow assay (LFA) readout, it was found to be important to include short random DNA to avoid the small level of false positive signals by LFA. We speculate that the cationic pc-PNAs form charge complexes with the random DNA oligomers that are produced in the RPA and that these complexes are stable enough to give a faint false positive on the test band. This band was completely suppressed with the addition after the RPA of random 20-mer DNAs (N20), which compete and reduce the probability that PNAs containing the biotin or fluorescein associate with the same DNAs. Gratifyingly, the LFA yielded a response in the test band that was proportional to the input of viral RNA and clearly discernable by the naked eye, with as little as 20 copies of input RNA still yielding a visible readout (Figure 4C). Compared to our previous protocol using the templated reaction,^48^ the procedure reported herein is operationally simpler and faster. It can be performed in 30 min without any specialized equipment making it highly amenable to point-of-care diagnostic testing or even home testing.

**Figure 5:**
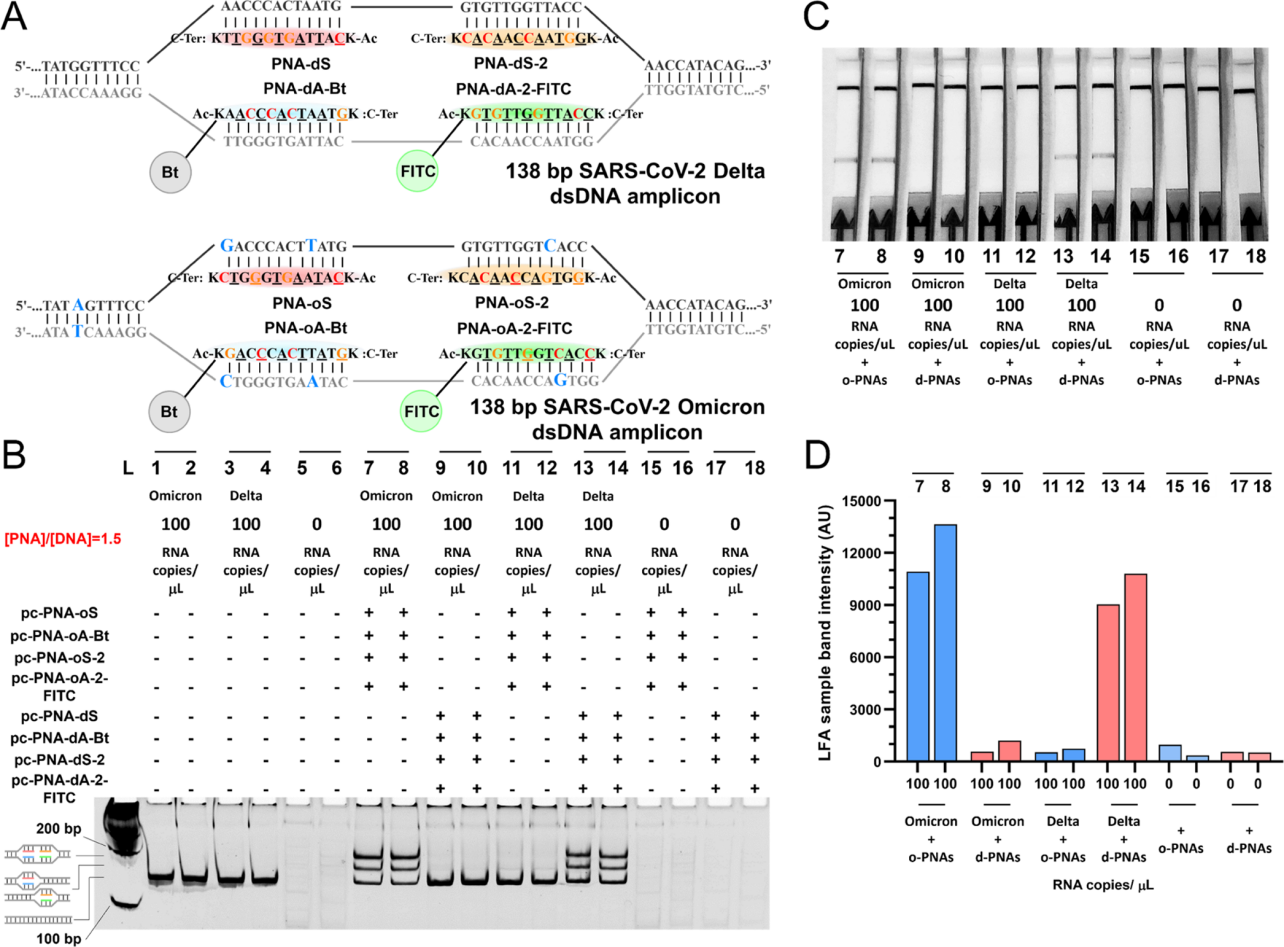
(A) Invasion scheme of pc-PNA oligomers into the138 bp dsDNA target amplicon from SARS-CoV-2 Delta/Omicron forming the desired invasion complex. (B) Native-PAGE gel analysis of analytical duplicates after RT-RPA amplification from SARS-CoV-2 Delta and Omicron variants RNA templates and invasion with Omicron and Delta pc-PNA probes. Lanes 1 to 6, products of RT-RPA as analytical duplicate pairs. Lanes 7 and 9, invasion after RT-RPA input from lane 1. Lanes 8 and 10, invasion after RT-RPA input from lane 2. Lanes 11 and 13, invasion after RT-RPA input from lane 3. Lanes 12 and 14, invasion after RT-RPA input from lane 4. Lanes 15 and 17, invasion after RT-RPA input from lane 5. Lanes 16 and 18, invasion after RT-RPA input from lane 6. (C) LFA readout of analytical duplicates after RT-RPA amplification from SARS-CoV-2 Delta and Omicron variants RNA templates and invasion with Omicron and Delta pc-PNA probes. Lanes referred to samples at (5C). (D) LFA test band intensity quantification. LFA band intensities were quantified with ImageJ software.

Having demonstrated that the dsDNA invasion complex could be used to detect the SARS-CoV2 amplicon with excellent sensitivity (20 copies), we next focused on the discrimination of different strains. Based on the emergence of the Delta (B.1.617.2) and Omicron variants (BA.1 and BA.2 lineages) as strains of concern, we sought to discriminate between these two strains. The primers used in the previous experiment amplify a region that does not contain mutations. Using a sequence corresponding to the spike protein identified in silico and validated experimentally by RT-PCR,^60^ we designed the RT-RPA primers to obtain a 138 bp amplicon with diagnostic mutations. It is interesting to note that the primers cover an area with a single nucleotide mismatch between delta and omicron but amplified the RNA template of both strains equally well (Figure 5B, lane 1 vs lane 3), consistent with the fact that a single mismatch in a central region of the primer is not sufficient for discrimination. As further evidence that the primer amplified both RNA oligomers, we performed a single-strand conformation polymorphism analysis^61^ and observed distinct bands for the two different templates following a snap annealing of the RPA product (Figure S8). As before, we designed two sets of pc-PNA functionalized with biotin and FITC, respectively, targeting the central region of the amplicon (Figure 5A, see Figure S1 for detailed structures of PNA). For one set of PNA (FITC), there is a single nucleotide mismatch between the omicron and delta amplicons, whereas the second set has a double mismatch. As shown by SDS-PAGE, the set of probes discriminated remarkably well between the two different amplicons (Figure 5, lane 7 vs 9 and 11 vs 13). It was found that increasing the Mg^2+^ concentration from 1 mM to 10 mM suppressed invasion even for the single nucleotide mismatched sequence, albeit at the detriment of quantitative invasion as had been previously observed. Elution of the mixture by the LFA assay provided a detectable signal only for the pc-PNA probes matching the genetic information of the strain, concurring the results observed by gel and demonstrating that quantitative invasion is not necessary for detection (Figure 5C,D). Quantification of the LFA signal showed that mismatch probes afford a signal comparable to the background (no RNA input), whereas the cognate pair affords a signal 8–10 times over the background. Taken together, the results indicate that the pc-PNA probes can discriminate a single nucleotide mismatch.

## Conclusions

In conclusion, we report a pseudo-complementary G–C base pair that leads to stable dsDNA invasion with PNA at physiological salt concentrations. This pc-set enables the design of PNA that have a stronger affinity for DNA than complementary PNA. We illustrate the potential of this with an application in diagnostics; we show that dsDNA invasion provides a fast and simple method to analyze amplicons of RPA. The test can be performed in less than 1 h (RT-RPA, dsDNA invasion, LFA) without specialized equipment making it accessible to point-of-care, field, or home settings. Given that RPA is carried out in high salt concentrations, the ability of these PNA to form stable invasion is quintessential. We show that the dsDNA invasion can proceed with single nucleotide resolution, enabling the discrimination of two strains of SARS-CoV-2 (Delta vs Omicron). The demonstration of dsDNA invasion at high salt concentrations may also find applications in gene editing or in PANDA.^42^ The pc-nucleobases also reduce the probability of folded structures and may also find applications in DNA nanotechnology. Indeed, pc-PNA using the U^s^ and A pseudo pair were used to actuate a DNA rotaxane architecture.^62^ We anticipate that the general design of using the G-clamp as a pseudo-complementary nucleobase for cationic forms of G is general and should be applicable to PreQ. 1.^63^ While the present work was performed with PNA, the findings should translate to other oligonucleotide backbones such as aTNA or SNA.^64,65^ Finally, the demonstration that a G-clamp leads to a significant reduction in hybridization to N7-G but a higher affinity to unmodified G suggests that probes could be designed to detect this natural post-transcriptional modification by competitive binding of oligomers bearing or not a G-clamp.

## Methods

The PNA oligomers were synthesized as previously reported.^66,67^ For full synthetic sequence and physical characterization, see the Supporting Information.

### Dissociation Constant (*K*_D_) Determination by Steady-State FRET Measurements

The donor fluorophore DNA/PNA conjugate was prepared as a solution at a constant concentration and mixed with the donor fluorophore DNA/PNA conjugates, prepared as a series of solutions with increasing concentrations in a buffer to obtain a final solution at simulated physiological salt conditions (PS - 20 mM Tris, pH 7.5 containing KCl: 140 mM; NaCl 20 mM; CaCl_2_: 0.1 mM; MgSO_4_: 1 mM; 0,02% Tween-20). The mixtures were transferred into black 96-well plates, 250 μL per well, before measuring the fluorescence at 25 or 37 °C. The fluorescence emission measurements were performed in an automated manner with temperature control using a SpectraMax^i3^ fluorescence multiwell plate reader. Each FRET mixture was excited with two different excitation wavelengths depending on the FRET fluorophore pair used and the fluorescence emission spectra were recorded. Two FRET pairs were used: FITC (ex. 468 nm, em. 520 nm) and Cy3 (e. 528 nm, em. 562 nm) or Cy3 and Atto647N (ex. 525 nm, em. 620 nm). The raw fluorescence emission signals were background-corrected and averaged between three experimental replicates for each condition. Each data point represents the mean and the associated RMSD of the three experimental replicates. The *K*_D_ values were obtained by fitting the data sets following previously reported protocols.^68,69^

Additional details are provided in Supporting Information Section 7.

### PCR Amplification and Purification of the 105 bp dsDNA Amplicon

PCR amplification was carried out in a 96 PCR well plate with a final volume of 50 μL per reaction. The amplification was initiated as follows: 5.0 μL of the standard Taq reaction buffer, 40.5 μL of water, 1.0 μL of the dNTPs 10 mM solution mix, 1.0 μL of the 10 μM Cy3-labeled forward primer (5′-Cy3-GTGGCGGTTCACTATATGTT-AAACCAGGTGGAA-3′), 1.0 μL of the 10 μM phosphorylated reverse primer (5′P-ATTGGCCGTGACAGCTTGACAAATGTTAAAAAC-3′), 0.5 μL of Taq DNA polymerase (5 U/μL), and 1.0 μL of a 4 pM ssDNA template solution (4.8 × 10^4^ copies DNA/μL, final volume). After 25 PCR cycles, the 96 reactions were combined, and ethanol precipitated and was purified by QIAquick PCR Purification. The purified 105nb dsDNA amplicon was eluted in water and used directly for the invasion experiments.

The 135nb ssDNA template used for amplification corresponds to the nucleotides 15 418-15 554 of the SARS-CoV-2 genome (Wuhan-Hu-1 /NCBI reference: NC_045512.2), the ORF1 region (135nb DNA template, 5′-GCTCAAGTATTGAGTG-AAATGGTCATGTGTGGCGGTTCACTATATGTTAAACCAGGTGGAACCTCATCAGGAGATGCCACAACTGCTTATGCTAATAGTGTTTTTAACATTTGTCAAGCTGTCACGGCCAATGTT-3′). The designed primer set amplifies the following 105nb DNA sequence (5′- GTGGCGGTTCACTATATGTTAAACCAGGTGGAACCTCATCAGGAGATGCCACAACTGCTTATGCTAATAGTGTTTTTAACATTTGTCAAGCTGTCACGGCCAATG-3′).

Additional details are provided in Supporting Information Section 8.1.

### Invasion Studies of the Purified 105 bp dsDNA Amplicon

Unless otherwise specified, invasion reactions were carried out in PCR tubes in 40 to 100 μL total volume: 150 nM purified 105nb dsDNA PCR amplicon, PNA concentration indicated by the [PNA]/[DNA] ratio and 1× buffer (LS or PS). Samples were incubated at the indicated temperature (37, 50, 60, 95 °C). For annealing conditions, samples were heated at 95 °C for 5 min before cooling down to the indicated temperature. Aliquots were taken at the indicated times and either analyzed directly by gel or snap-frozen until analysis. PNAs were used from 5–10 μM stocks and buffers prepared at 10×: low-salt buffer (LS - 20 mM Tris, pH 7.5, 0,02% Tween-20, final concentration); simulated physiological salt buffer (PS - 20 mM Tris, pH 7.5 containing KCl: 140 mM; NaCl 20 mM; CaCl_2_: 0.1 mM; MgSO_4_: 1 mM; 0,02% Tween-20, final concentration). The formation of the desired PNA–DNA invasion complex was followed by gel analysis with 15% Native-PAGE at 4 °C and 20 V/cm. The DNA was visualized by SYBR Gold Nucleic acid staining. Invasion quantification was performed by integration of the band signal using ImageJ.

Additional details are provided in Supporting Information Section 8.

### Detection of Viral RNA Sequences by RT-RPA Coupled to dsDNA Invasion by pc-PNAs and LFA Readout

Following the general procedure of the RPA TwistAmp Basic kit (TwistDx: TABAS03KIT), RPA was carried out in a PCR tube in 50 μL final volume and 500 nM primers. The amplification was initiated as follows: 30 μL of the primer-free rehydration buffer mixed with 9.5 μL of water, 2.5 μL of the 10 μM Cy3-labeled forward primer and 2.5 μL of the 10 μM reverse primer, 1.0 μL of RevertAid Reverse Transcriptase (200 U/μL, Thermo Fisher Scientific, Ref: EP0), 1.0 μL of the Recombinant RNasin Ribonuclease Inhibitor (40 U/μL, Promega, Ref: N251A). This was followed by the addition of either 1.0 μL of viral RNA at the indicated final copies per microliter or 1.0 μL of water and 2.5 μL of Mg(OAc)_2_ 280 mM. The RT-RPA reaction was carried out for a total of 30 min at 41 °C. Next, 20 μL of the RT-RPA mixture was combined with 130 μL of the PNA invasion mixture (115 nM PNAs, 46 ng/μL 20-mer ssDNA (N20), 1.15× PS buffer). The ratio of PNA/DNA was calculated assuming complete amplification of the primers. The resulting mixture was heated to 95 °C for 5 min and either filtered through a handmade Kimtech plug (see Supporting Information Sections 8.1 and 10) or centrifuged for 5 min at 14 k rpm. Then, 15 μL of the resulting solution was diluted up to 100 μL in the LFA buffer before addition to the LFA strip (Milenia GenLine HybriDetect Ref: MGHD 1). LFA strips were imaged after 10 min. The formation of the desired PNA–DNA invasion complex was also followed by gel analysis with 15% Native-PAGE at 4 °C and 20 V/cm. DNA was visualized by SYBR Gold Nucleic acid staining.

Additional details including primer sequences, amplified sequences, sequence alignments, and further experimental details are provided in Supporting Information Sections 8 and 10.

## Supporting Information Available

General chemical structures and nomenclature used for PNA oligomers; *K*_D_ of different PNA/DNA-fluorophore conjugates measured by FRET steady-state experiments; gel analysis showing the invasion G^+^–C^+^ pc-PNAs for the purified 105 bp amplicon at different concentrations and different time points at 37 °C; comparison between invasion complex quantification with SYBR gold vs Cy3; kinetic experiments involving the invasion of the G^+^–C^+^ pc-PNAs (pc-PNA-SR-Bt and pc-PNA-AR-FITC) into the purified 105 bp dsDNA PCR amplicon; scheme of 4-PNAs probes into target 105 bp dsDNA amplicon; single-strand conformation polymorphism (SSCP) analysis; detailed procedures; and physical characterization of compounds reported (PDF)

## Notes

The raw data have been deposited on Zenodo (https://zenodo.org, DOI: 10.5281/zenodo.7575983).
