# Supplementary material for: Pseudo-Complementary G:C Base Pair for Mixed Sequence dsDNA Invasion and Its Applications in Diagnostics (SARS-CoV-2 Detection)
Source: JACS Au. 2023 Feb 1;3(2):449–58. doi: 10.1021/jacsau.2c00588 (PMC9975836; doi:10.1021/jacsau.2c00588)
Supplement: Supplementary file 1 — au2c00588_si_001.pdf [file au2c00588_si_001.pdf]

Supporting Information

Pseudo-Complementary G:C Base Pair for Mixed Sequence dsDNA Invasion and its Applications in Diagnostics (SARS-CoV-2 Detection)

Miguel López-Tena, Lluc Farrera-Soler, Sofia Barluenga, Nicolas Winssinger*

Department of Organic Chemistry, NCCR Chemical Biology, Faculty of Science, University of Geneva, 1211 Geneva, Switzerland

Nicolas.Winssinger@unige.ch

Table of contents

1. Supplementary Figures S1 to S9 2
2. Abbreviations 10
3. General methods 11
4. Synthesis of PNA oligomers 12
5. PNA monomer synthesis 15
6. Characterization of PNA oligomers 26
7. Dissociation constant (K_D) determination by FRET measurements 57
   7.1. Parameter α & β of PNA and DNA conjugates with FITC/Cy3/Atto647N 60
   7.2. K_D of PNA and DNA conjugates with FITC/Cy3/Atto647N by FRET measurements 66
   7.3. K_d of PNA and PNA conjugates with Cy3/Atto647N by FRET measurements 76
8. Protocols 81
   8.1. Protocols for amplification and detection of SARS-CoV-2 viral RNA at the ORF1 region 81
   8.2. Protocols for amplification and detection of SARS-CoV-2 viral RNA of Omicron versus Delta variant 84
9. Raw copies of acrylamide and agarose gels 87
10. Raw copies of the LFA strips 94
11. Copies of the ^{1}H and ^{13}C NMR spectra for compounds *1*, *4*, *7*, *8*, *9*, *11*, *13*, *16*, *21* 97
12. Copies of the LC-MS traces for compounds *1*, *4*, *7*, *8*, *9*, *11*, *13*, *16*, *21* 104
13. References 110

1. Supplementary Figures S1 to S9

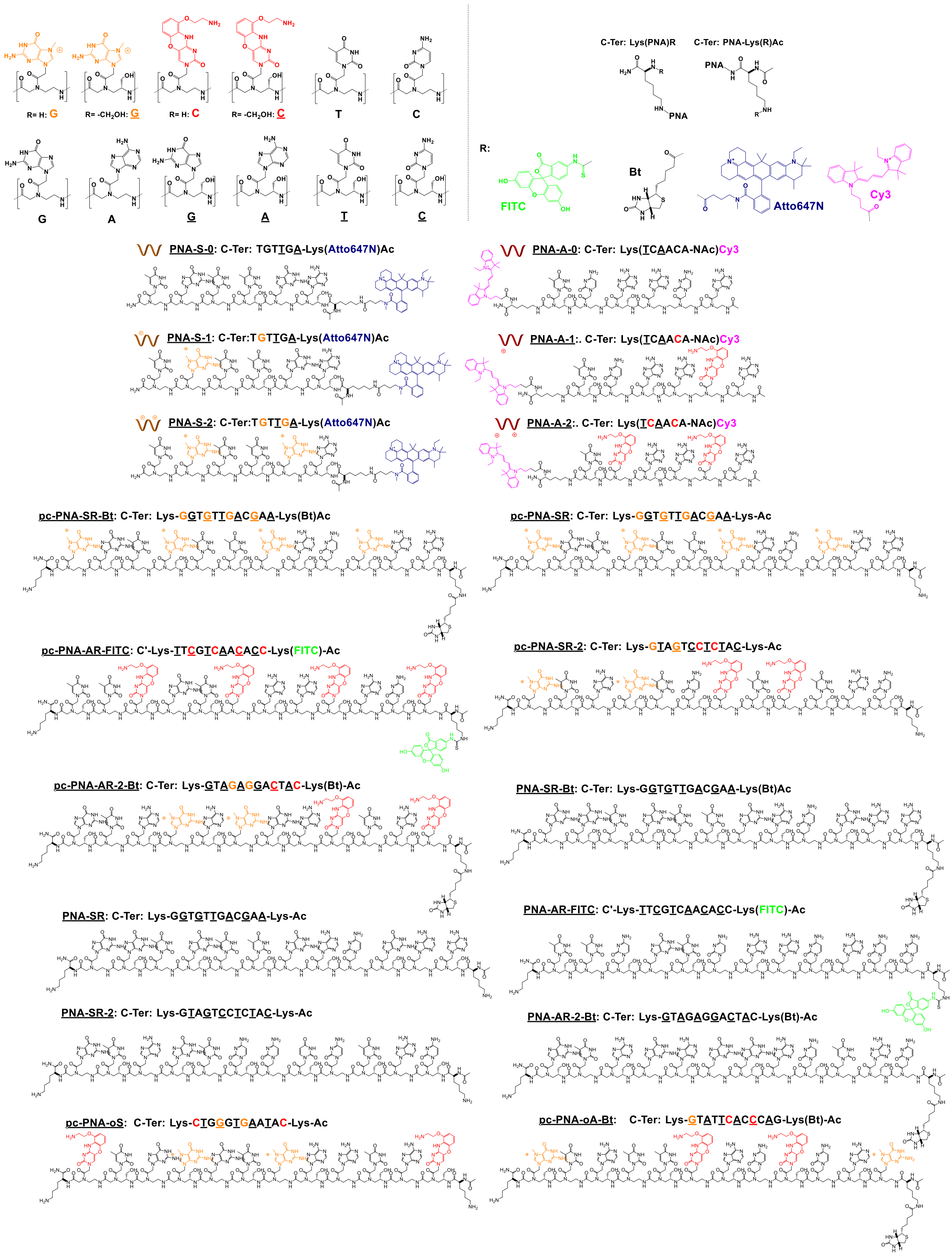

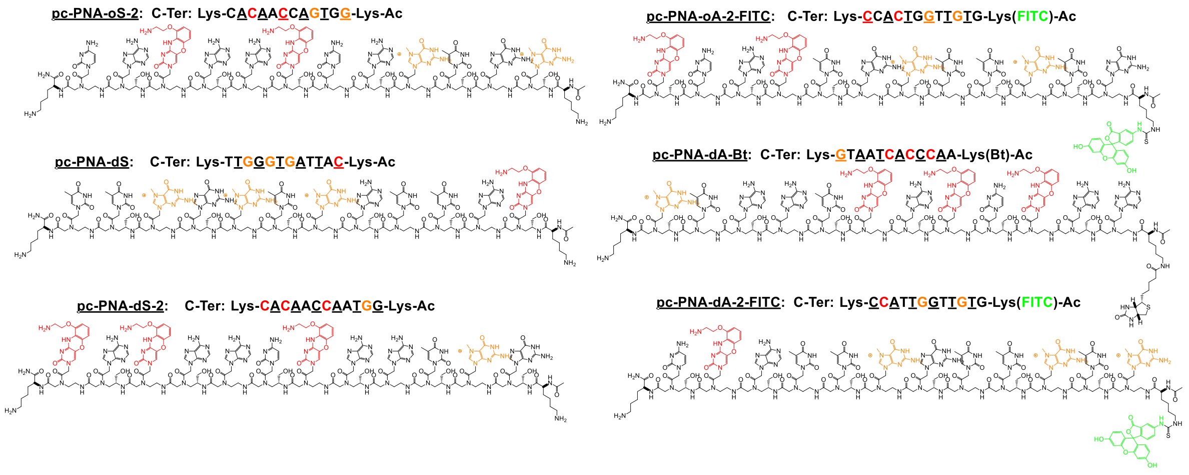

Figure S1. General chemical structures and nomenclature used for PNA oligomers.

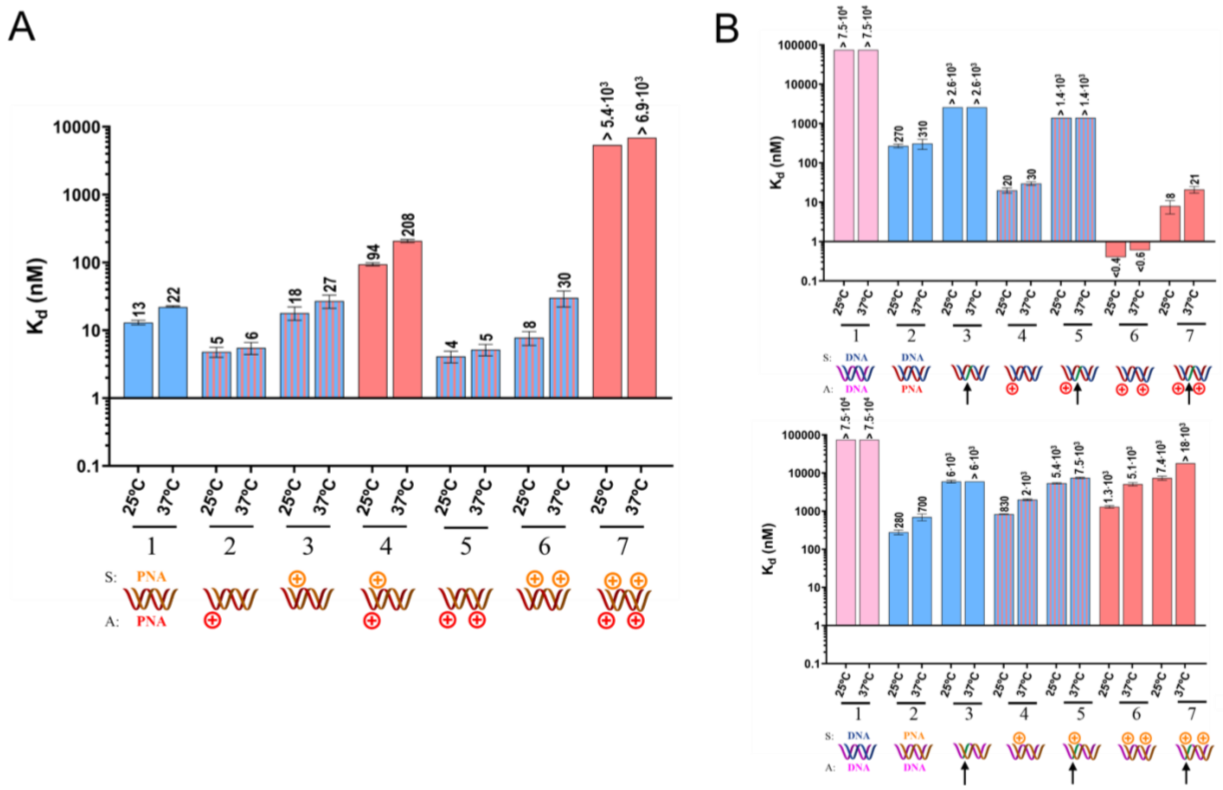

Figure S2. K_D of different PNA/DNA-fluorophore conjugates measured by FRET steady-state experiments. A) K_D PNA-PNA conjugates B) K_D PNA/DNA or DNA/DNA conjugates.

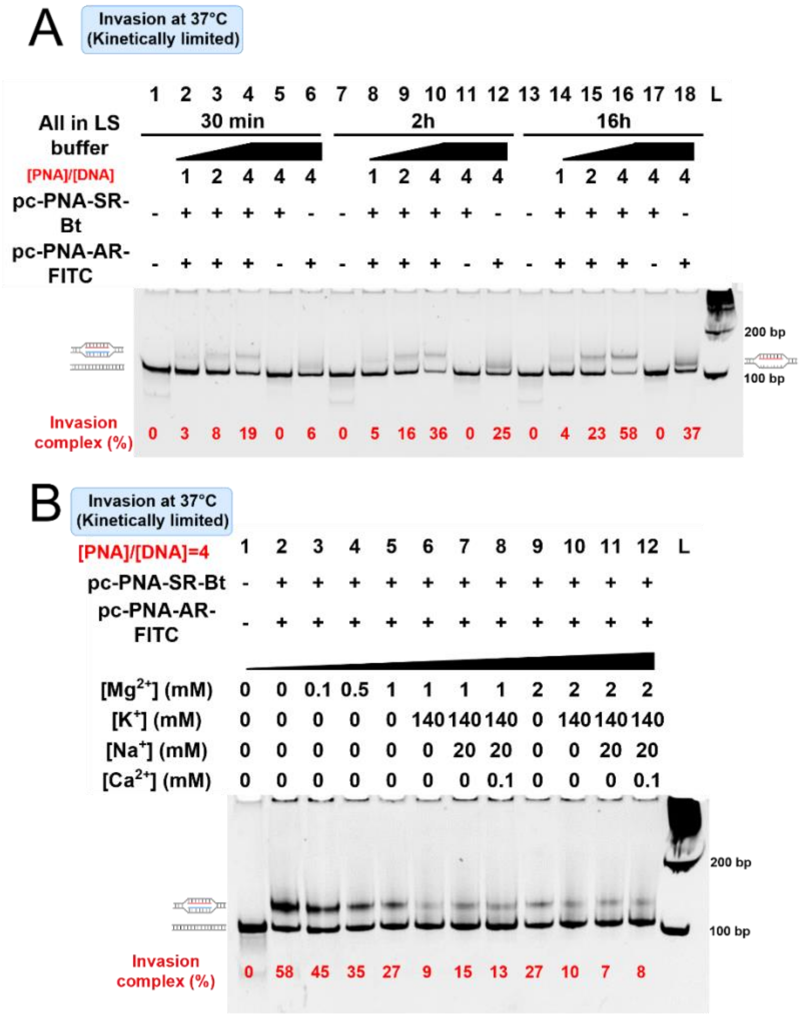

Figure S3. A) Gel analysis showing the invasion G^+-C^+ pc-PNAs for the purified 105bp amplicon at different concentrations and different time points at 37°C. B) Gel analysis showing the invasion G^+-C^+ pc-PNAs for the purified 105bp amplicon at different salt concentrations after 16h at 37°C. For experimental details please see section S8.1.

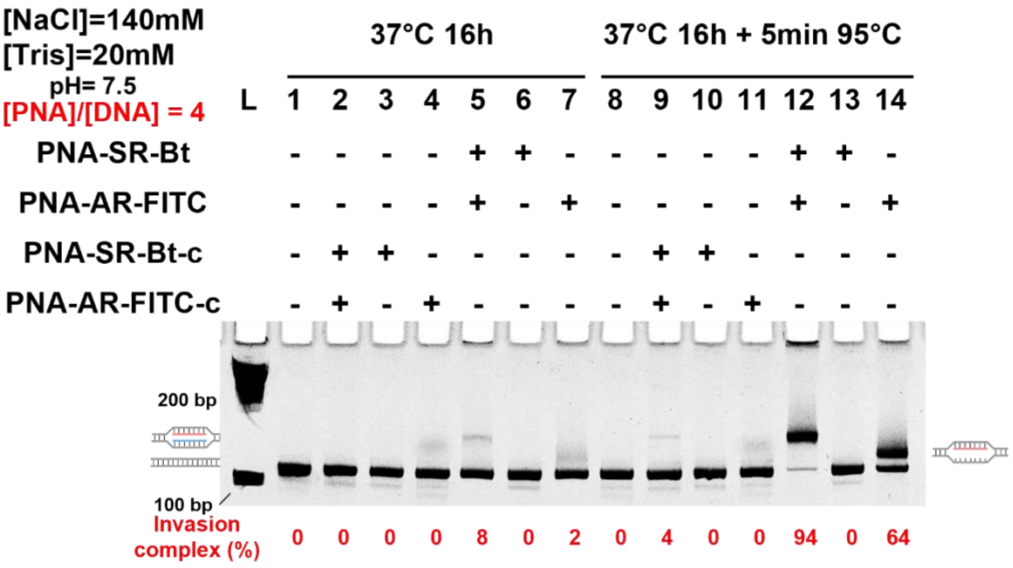

Figure S4. Gel analysis showing the invasion G+-C+ pc-PNAs for the purified 105bp amplicon at high NaCl salt buffer (20mM Tris, pH=7.5, 140mM NaCl, 0.02% tween-20) after 16h at 37°C and the same samples after 16h at 37°C + 95°C annealing. Same protocol as described in section 8.1. for the "invasion optimization at the purified 105bp amplicon".

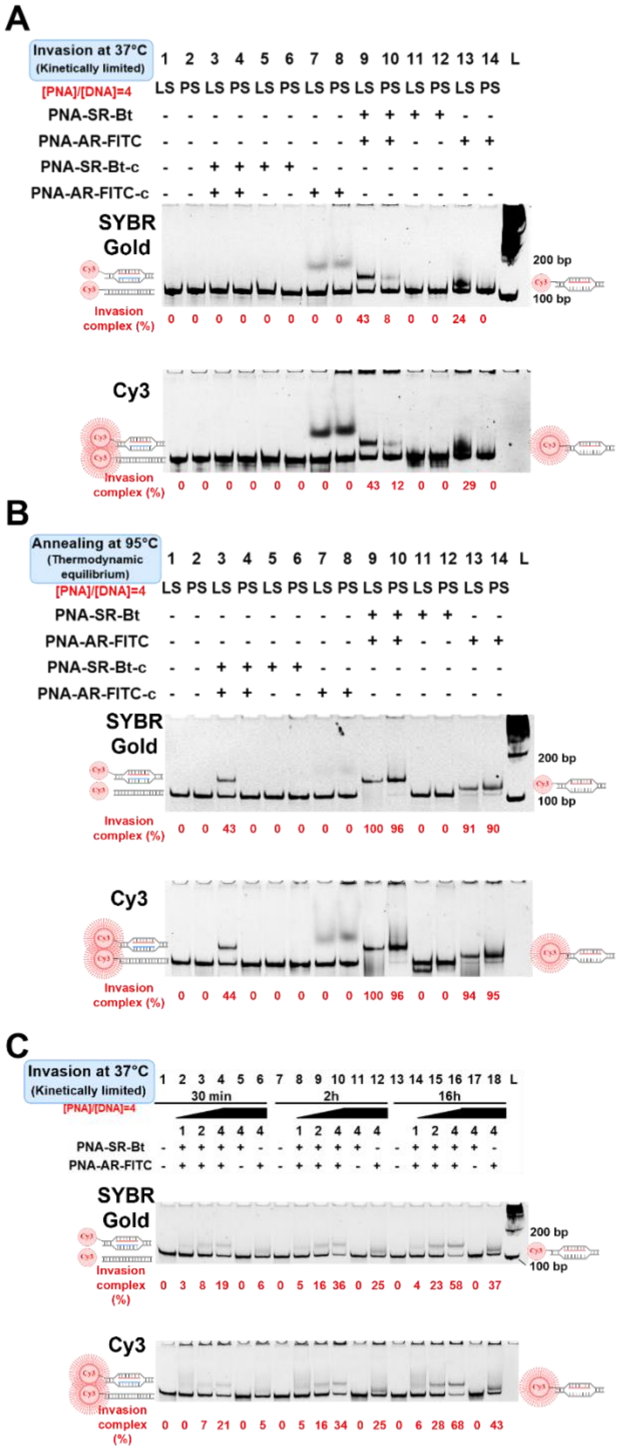

Figure S5. A) Figure 3C comparison between invasion complex quantification with SYBR gold vs Cy3. B) Figure 3B comparison between invasion complex quantification with SYBR gold vs Cy3. C) Figure S3A comparison between invasion complex quantification with SYBR gold vs Cy3. Invasion complex (%) was quantified with ImageJ. Cy3 was incorporated into the dsDNA amplicon by a Cy3 forward primer.

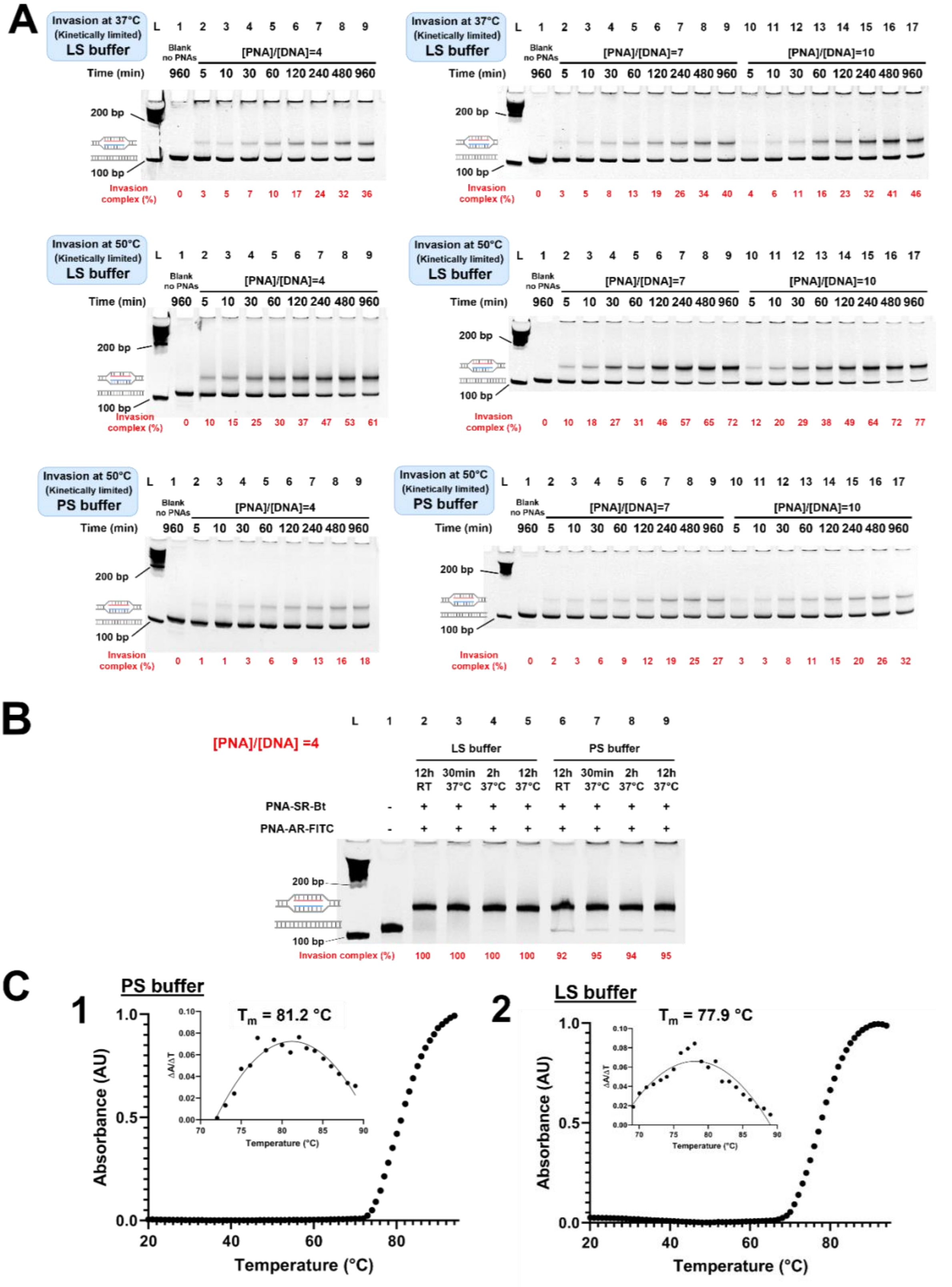

Figure S6. A) Kinetic experiments involving the invasion of the G^+-C^+ pc-PNAs (pc-PNA-SR-Bt and pc-PNA-AR-FITC) into the purified 105bp dsDNA PCR amplicon at different time points for different PNA/DNA ratios, temperatures and salt buffer conditions, either simulated physiological salt conditions (PS - 20 mM Tris, pH 7.5 containing KCl: 140 mM; NaCl 20 mM; $CaCl_2$: 0.1 mM; $MgSO_4$: 1 mM; 0,02% Tween-20) or low salt buffer conditions (LS - 20 mM Tris, pH 7.5, 0,02% Tween-20). All kinetic experiments were performed in PCR Eppendorf tubes at a final concentration of 150nM of dsDNA, aliquots were taken at the times indicated and quenched

over ice-cold stop-buffer (2x PS buffer). Samples were either frozen or kept at 0°C before loading into the native-PAGE. B) Gel analysis for the stability check of the invasion complex between G^+-C^+ pc-PNAs (pc-PNA-SR-Bt and pc-PNA-AR-FITC) and the purified 105bp dsDNA PCR amplicon at either simulated physiological salt conditions (PS) or low salt buffer conditions (LS). Samples were prepared in PCR Eppendorf tubes at a 150nM dsDNA final concentration, heated to 95°C for 3 minutes and quickly placing the sample to equilibrate at the indicated temperature for the indicated time. No clear sign of dissociation of the invasion complex observed during the experimental time. C) Melting temperature (T_m) curves for the 105bp purified dsDNA PCR amplicon. After initial heating to 95°C, the sample was left to cool to 20°C at a rate of 1°C per minute. Then after equilibration at 18°C for 5 min, sample was heated to 95°C at a rate of 1°C per minute with monitoring at 260 nm. The T_m for the duplex was determined using the maximum of the first derivative of the heating curves, then taken as an average of three runs. Measured at 150nM dsDNA in simulated physiological salt conditions (PS) for Figure S6C1 or in low salt buffer conditions (LS) for Figure S6C2.

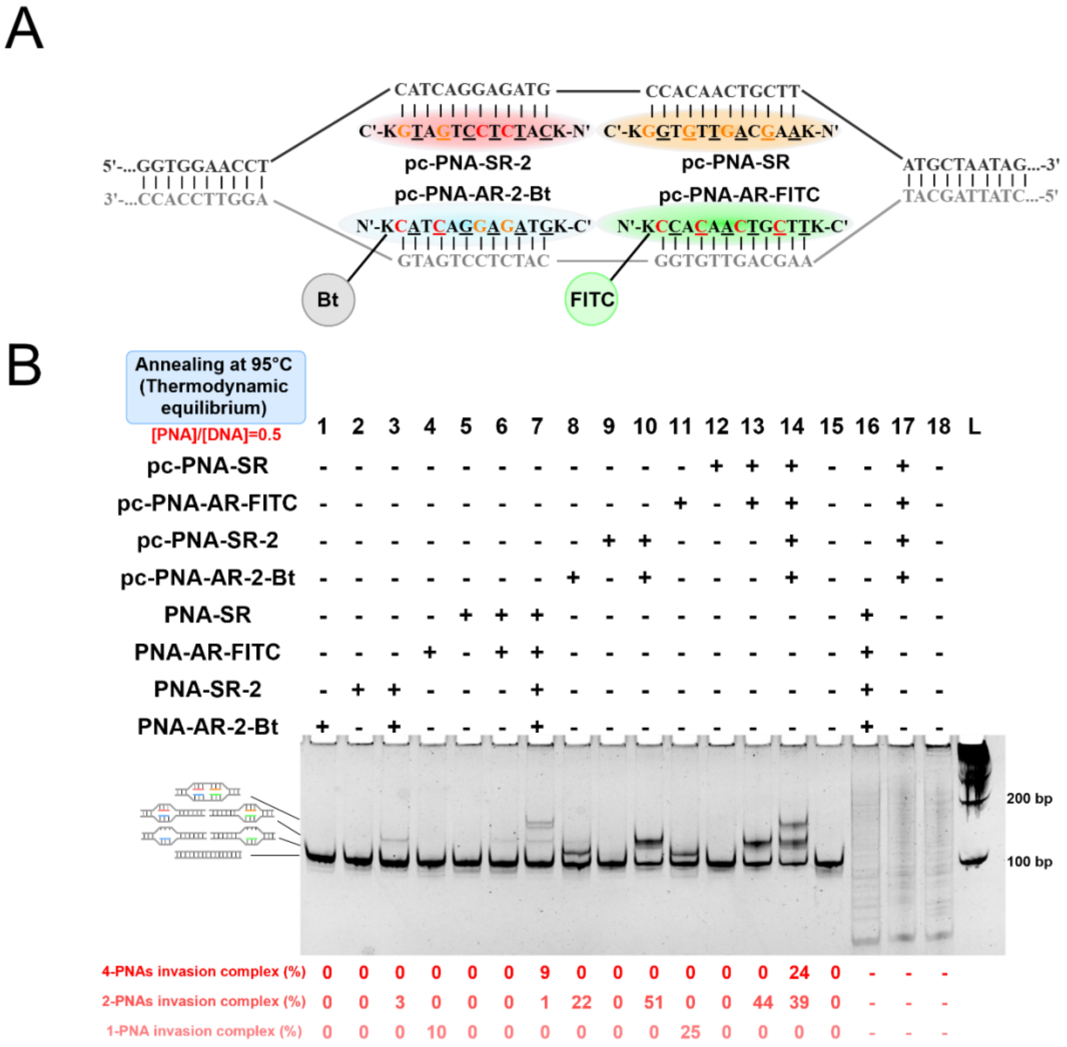

Figure S7. A) Scheme of 4-PNAs probes into target 105 bp dsDNA amplicon B) Sequences of 4-PNAs probes into target 105 bp dsDNA amplicon C) Gel analysis showing the invasion G^+-C^+ pc-PNAs versus c-PNAs at RPA mixtures for the 105bp amplicon. For experimental details please see section S8.1.

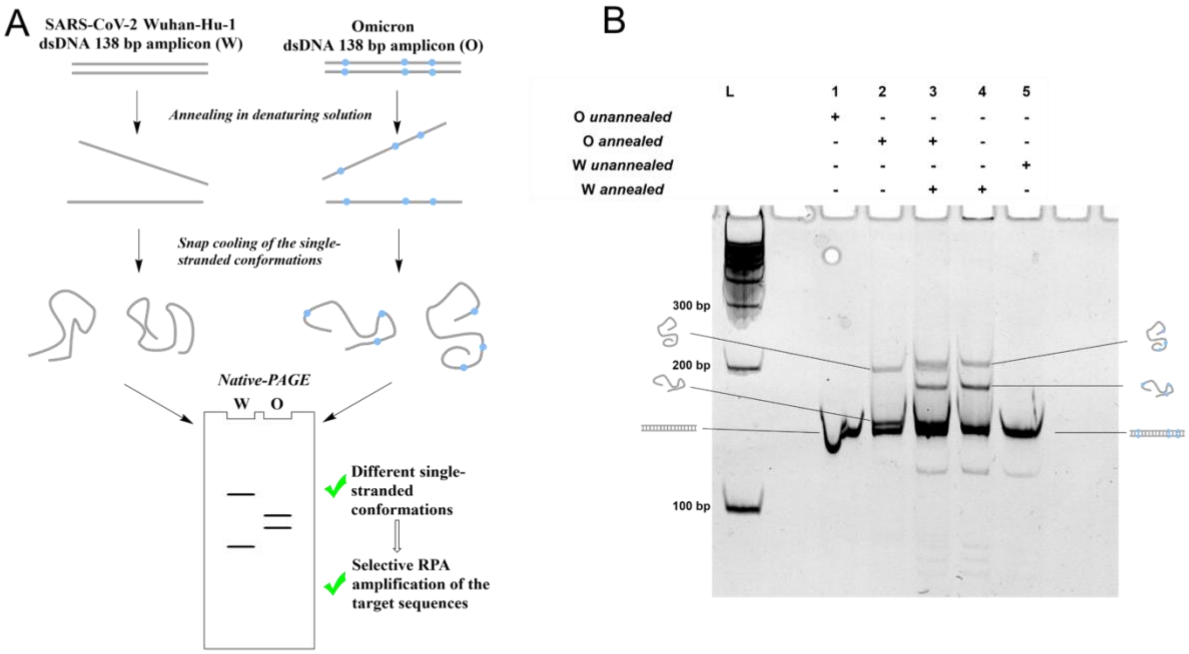

Figure S8. A) Single-Strand Conformation Polymorphism (SSCP) analysis scheme for the 138bp amplicons B) SSCP analysis of purified RPA 138bp amplicons. For experimental details please see section S8.2.

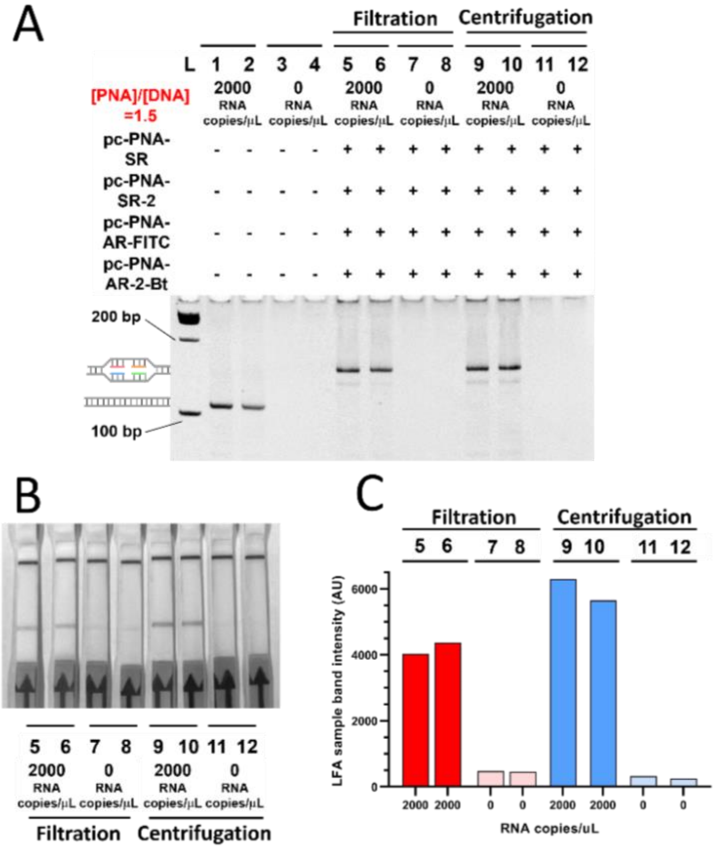

Figure S9. A) Gel analysis after RT-RPA amplification from SARS-CoV-2 Wuhan-Hu-1 RNA template, invasion G^{+}-C^{+} pc-PNAs, comparing between filtration vs centrifugation before LFA read-out. B) LFA readout of analytical duplicates of the samples at Figure S9A. C) LFA test band intensity quantification. Lanes referred to samples at Figure 9A. LFA band intensities were quantified using ImageJ. For experimental details please see section S8.1. and for raw images and filter set-up please see section S10.

2. Abbreviations

Boc	Tert-butyloxycarbonyl
DCE	1,2-dichloroethane
DHB	2,5-Dihydroxybenzoic acid
DIPEA	N,N-Diisopropylethylamine
DMF	Dimethylformamide
DMSO	Dimethyl sulfoxide
ESI	Electrospray ionization
EtOAc	Ethyl acetate
FITC	Fluorescein isothiocyanate
Fmoc	Fluorenylmethoxycarbonyl
HATU	Hexafluorophosphate Azabenzotriazole Tetramethyl Uronium
HIFP	Hexafluoroisopropanol
HOBt	Hydroxybenzotriazole
HPLC	High performance liquid chromatography
LC-MS	Liquid chromatography-mass spectrometry
MALDI	Matrix-assisted laser desorption/ionization
MS	Mass spectrometry
NMP	N-Methyl-2-pyrrolidone
PEG	Polyethylene glycol
PG	Protecting group
PNA	Peptide Nucleic Acid
TFA	Trifluoroacetic acid
THF	Tetrahydrofuran

3. General methods

All reagents and solvents for the organic synthesis were purchased from commercial sources and were used without further purification. NovaPEG Rink amide resin for peptide synthesis was obtained from EMD Millipore. HPLC purification was performed with an Agilent Technologies 1260 infinity HPLC using a ZORBAX 300SB-C18 column (9.4 x 250 mm). LC-MS spectra were recorded on a DIONEX Ultimate 3000 UHPLC (condition for elution gradient: 0 min, A:B =100:0; 4 min, A:B = 10:90; solution A: 0.01% aqueous TFA solution; solution B, 0.01% TFA in HPLC grade acetonitrile; flow rate: 0.750 mL/min) with a Thermo LCQ Fleet Mass Spectrometer System using PINNACLE DB C18 column (1.9 µm, 50 x 2.1 mm) operated in positive mode. All the LC-MS spectra were measured by electrospray ionization (ESI), linear gradient 0 to 100%. MALDI-TOF Mass spectra were measured using a Bruker Daltonics Autoflex spectrometer operated in positive mode. The samples were analyzed using 2,5-dihydroxybenzoic acid (DHB) matrix. SFC analyses were performed on a Waters Acquity UPC2 with OD-H column. Retention times (RT) are given in minutes. Thin layer chromatography (TLC) was performed on plates of silica precoated with 0.25 mm Kieselgel 60 F254 from Merck. Flash chromatography was performed using silica gel SiliaFlash® P60 (230-400 mesh) from Silicycle. Automated solid-phase synthesis was carried out on an Intavis AG Multipep RS instrument. Concentration of the PNA or DNA stocks was measured by NanoDrop^{RM} 2000c at 260nm wavelength. All DNA sequences were purchased from Eurogentec. The DNAs used as template for PCR/RPA were received from the supplier on the CPG resin, cleaved and purified by denaturing 4M Urea 18% PAGE before use. The rest of DNAs were used as received. All gels were imaged at a FUSION FX Imaging system. All quantifications for invasion complex formation or LFA strips band intensity were done with ImageJ. Thermal melting curves were obtained on a Jasco v-650 UV-visible spectrophotometer equipped with a water-circulated temperature-controlled cell holder monitoring at 260nm wavelength.

4. Synthesis of PNA oligomers

5.0 mg of Nova PEG® Rink amide resin (0.44mmol/g, NovaBiochem) were swollen in CH_2Cl_2 for 10 minutes and washed twice with DMF. Iterative cycles of amide coupling (**Procedure 1**), capping of the resin (**Procedure 4**), and deprotection of the protecting group (**Procedure 2 or 3**) were done to synthesize the PNA probes. The compounds were deprotected and cleaved from the resin using TFA (**Procedure 5**) and finally purified using HPLC.

Procedure 1 (P1): Amide coupling.

The corresponding Fmoc protected PNA monomer, fluorophore acid (Cy3/Atto647N) or Fmoc protected amino acid (4.0 equiv., 0.2M in NMP) was incubated for 5 minutes with HATU (3.5 equiv., 0.5M in NMP) and base solution [DIPEA, 1.2M (4.0 equiv.) and 2,6-lutidine 1.8M (6.0 equiv. in NMP]. The mixture was then added to the corresponding resin. After 20 minutes, the mixture was filtered, the resin was washed with DMF, and a new premixed reaction solution was added to the resin and let react for another 20 minutes. Finally, the resin was washed with 2x DMF, 2x CH_2Cl_2, and 2x DMF.

Procedure 2 (P2): Fmoc deprotection.

A solution of 20% piperidine in DMF was added to the resin and allowed to react for 5 minutes. The mixture was then filtered, the resin washed with DMF, and the sequence repeated a second time for another 5 minutes. Finally, the resin was washed with 2x DMF, 2x CH_2Cl_2, and 2x DMF.

Procedure 3 (P3): Mtt deprotection.

A solution (made from 244 mg of HOBt in 10 mL of HFIP and 10 mL of DCE) was added to the prewashed resin to reach a volume of 10 mL/g of resin and allowed to react for 5 minutes. The solution was flushed, the resin washed with CH_2Cl_2, and the sequence repeated a second time for another 5 minutes. Finally, the resin was washed with 2x CH_2Cl_2, and 2x DMF.

Procedure 4 (P4): Capping.

The resin was treated with a capping mixture (0.92 mL of acetic anhydride and 1.3 mL of 2,6 lutidine in 18 mL of DMF: 10 mL of solution/g of resin) for 5 minutes. After flushing the solution, the resin was washed with 2x DMF, 2x CH_2Cl_2, and 2x DMF.

Procedure 5 (P5): Cleavage from the resin and final deprotection.

Resin (5.0 mg, 1.0 μmol) was treated with 125 μL of a mixture of TFA and scavengers (440 μL of TFA + 25 mg phenol + 25 μL water + 10 μL triisopropylsilane) for 2 hours. The resin was filtered, washed with TFA (50 μL), and the collected fractions of cleavage product precipitated in cold ether (1.5 mL). After centrifugation, the pellet was vortexed again with cold Et_2O (1.5 mL) and centrifuged (14K rpm). The pellet was dissolved in H_2O/CH_3CN (3/1, 1.5 mL) and lyophilized to obtain a white powder.

Procedure 6 (P6): Microcleavage for quality control.

The minimum number of beads were picked up with a pipette plastic tip and transferred to 50 μL of TFA. The solution was left for 1 hour and transferred to 1.0 mL of ether. The ether solution was kept for 5 minutes at -20 ^oC and then centrifuged for 5 minutes at 14K rpm. The ether supernatant was removed, and the pellet dissolved in 20 μL 1:1 acetonitrile/water, which was then analyzed by MALDI and/or LC-MS.

Procedure 7 (P7): Coupling of Fluorescein isothiocyanate (FITC).

To 5.0 mg of resin (0.0022 mmol), a solution of 1.7 mg of FITC (0.0044 mmol, 2 equiv.) and 1.5 μL of DIPEA (0.0088 mmol, 8 equiv.) in 50 μL of NMP was added. After 20 minutes reaction at room temperature, the mixture was filtered, the resin was washed with DMF, and a new premixed reaction solution was added to the resin and let react for another 20 minutes. Finally, the resin was washed with 2x DMF, 2x CH_2Cl_2, and 2x DMF.

Characterization of PNA-peptide conjugates.

Characterization of the PNA-peptide conjugates was done by MALDI (Bruker Daltonics Autoflex spectrometer with Flex control 3.4 software and analysis with FlexAnalysis 3.4) and/or LC-MS (DIONEX Ultimate 3000 UHPLC with a Thermo LCQ Fleet Mass Spectrometer System using PINNACLE DB C18 column (1.9 μm, 50 x 2.1 mm) with Thermo Xcalibur 2.2.SP1.48 software and analysis with Thermo Xcalibur Qual Browser 2.2.Sp1.48). For MALDI analysis, 1.0 μL of the sample (in either water or water/acetonitrile 1:1) was mixed with 1.0 μL of DHB matrix solution (30 mg of DHB in 1.0 mL of 70:30:0.01 water/acetonitrile/TFA), and the mixture spotted on a MALDI plate. The measurements were done in a positive linear

mode. Calibration Standard Peptide II (Bruker LabScape – Daltonics 8222570) used prior to any measurement. For LC-MS analysis, 20 µL of sample in water or water/acetonitrile 1:1 was injected on the LC and further analyzed by MS on a positive mode.

Scheme for the synthesis of the PNA oligomers.

Synthesis of PNA oligomers containing Cy3 for FRET measurements

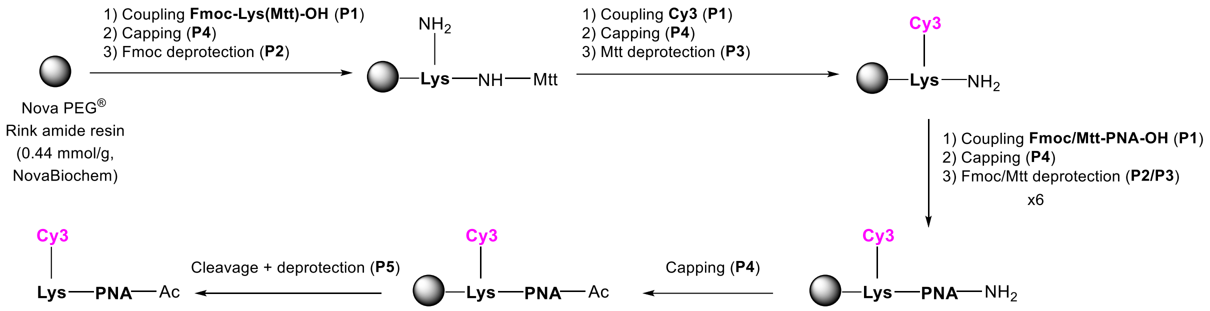

Synthesis of PNA oligomers containing Atto647N for FRET measurements

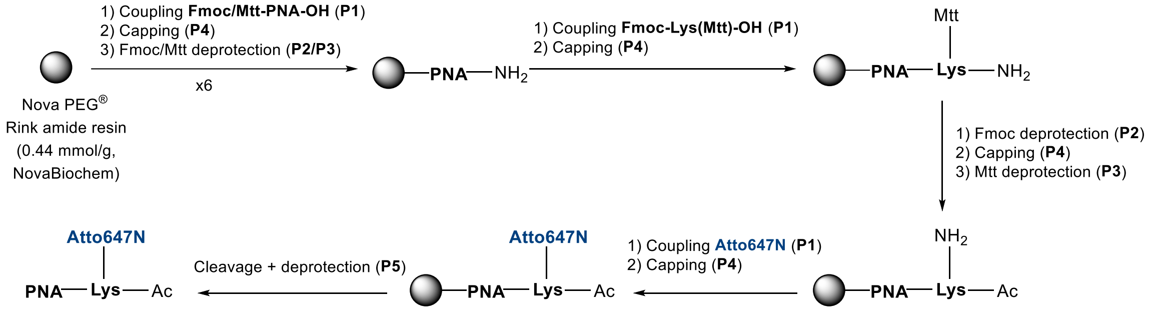

Synthesis of 12-mer PNA probes

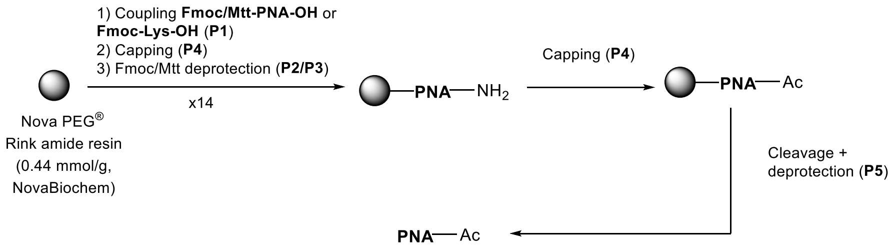

Synthesis of 12-mer PNA probes containing Biotin and FITC

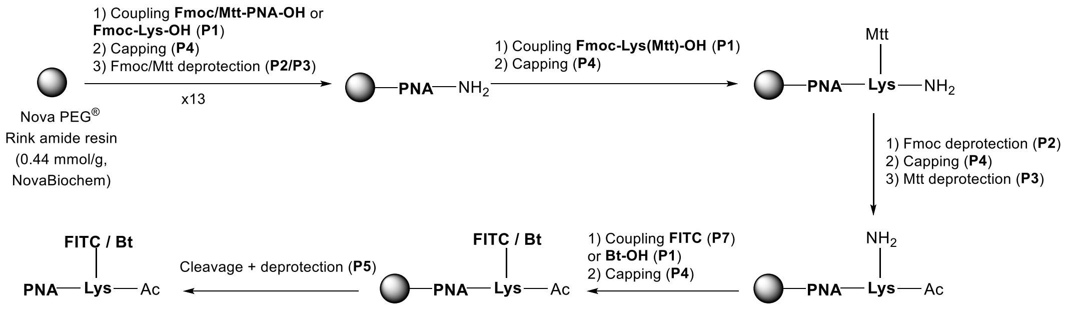

5. PNA monomer synthesis

5.1. Synthesis of PNA monomer Fmoc-Gclamp(Boc)-OH and Fmoc-γ-L-Ser(tBu)-Gclamp-OH

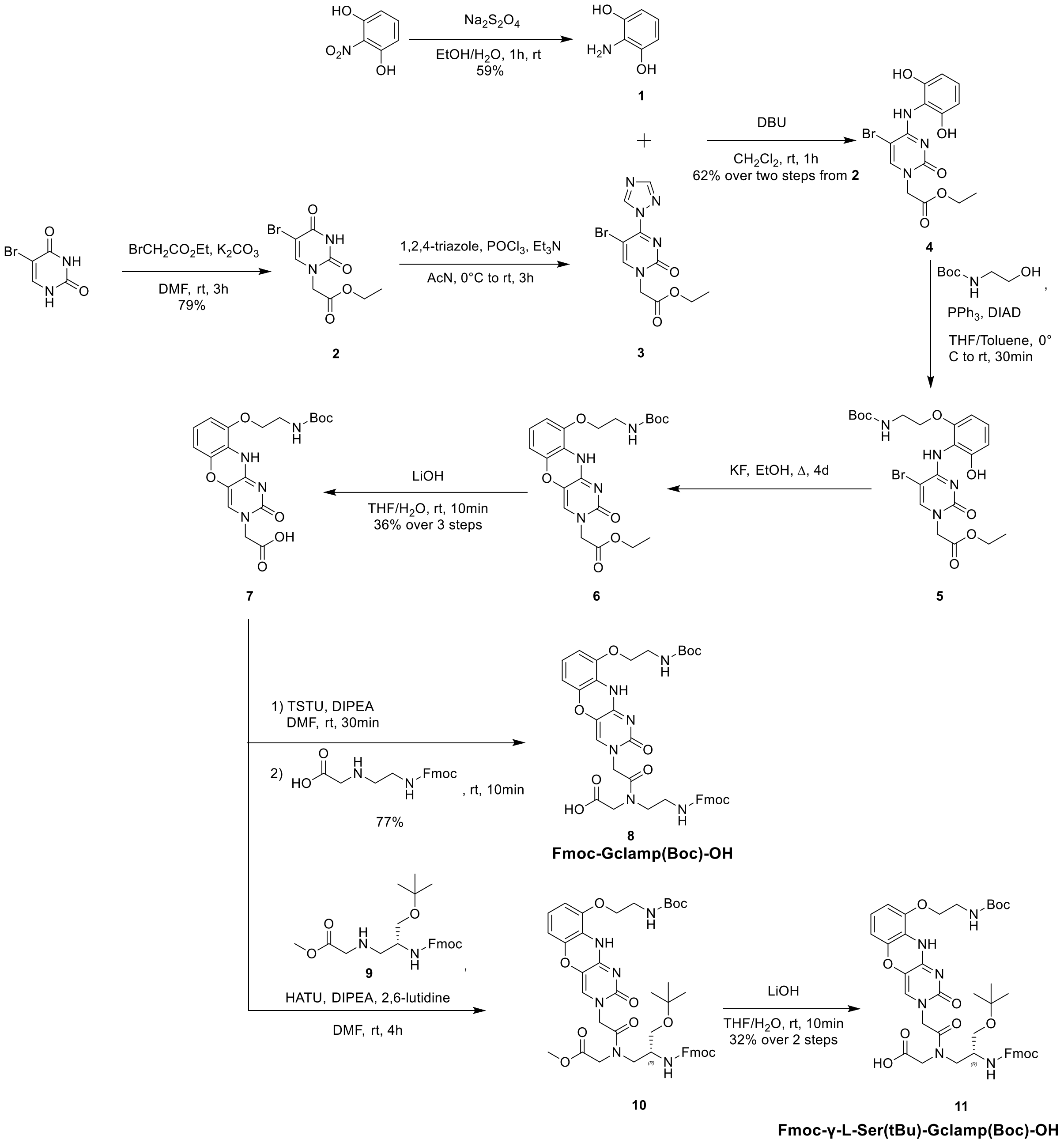

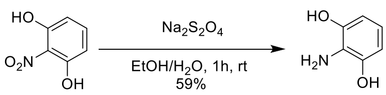

1

2-aminobenzene-1,3-diol (1). The compound was synthesized with slight modification of the previously reported procedure.[1] To a stirred solution of 2-nitroresorcinol (15 g, 91.87 mmol) in EtOH (920 mL) was added a solution of sodium dithionite (74.4 g, 367.48 mmol) in water

(300 mL) in one portion. The orange reaction mixture gradually turned into a white suspension. After 1h, the reaction mixture was filtered and concentrated in vacuo to dryness. The resulting solid was triturated overnight in EtOH (300 mL) before filtering it through a short silica path, washing with some ethyl acetate. The filtrate was concentrated in vacuo to give **1**.

Yield: 6.80 g (59%). Isolated as a beige solid. >95% pure by NMR and a single spot by TLC

R*f*: 0.72 in EtOAc. Stains yellow with $KMnO_4$ stain.

LC-MS-ESI (m/z): Calcd for $C_6H_7NO_2$ $[M + H]^+$: 126.05, Found 125.89

^{1}H NMR (300 MHz, D_2O): δ 6.66 (t, 1H), 6.50 (d, 2H).

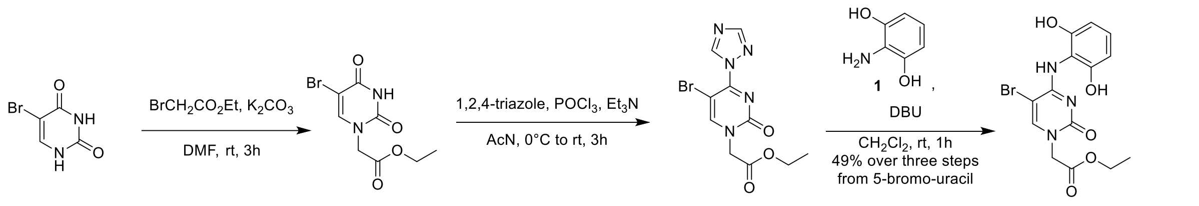

Ethyl 2-(5-bromo-4-((2,6-dihydroxyphenyl)amino)-2-oxopyrimidin-1(2H)-yl)acetate (4). The compound was synthesized with slight modification of the previously reported procedure.[2] To a stirred solution of 5-bromo-uracil (9.55 g, 50 mmol) in DMF (185 mL), freshly grounded potassium carbonate (6.91g, 50 mmol) and ethyl bromoacetate (5.55 mL, 50 mmol) were added in one portion. After 3h, the reaction mixture was partitioned between 10% citric acid aq. solution (250 mL) and EtOAc (400 mL). The organic layer was separated, and the aqueous layer was extracted once more with EtOAc (400 mL). Combined organic extracts were washed with water (4x100 mL), brine, dried over anhydrous Na_2SO_4, filtered, and concentrated in vacuo. The resulting crude white powder (12.86 g) was dissolved in CH_3CN (600 mL) together with 1,2,4-triazole (25 g, 355 mmol) and Et_3N (83 mL, 592 mmol). The mixture was cooled to 0°C and $POCl_3$ (7.5mL, 79 mmol) was quickly added. The reaction mixture was left to warm to room temperature, it gradually turned orange. After 3 h, the reaction mixture was concentrated to dryness in vacuo. The resulting orange solid was partitioned between CH_2Cl_2 (1.5 L) and a saturated aq. $NaHCO_3$ (400 mL) solution. The organic layer was separated and washed with water (2x400 mL), brine, dried over anhydrous Na_2SO_4, filtered, and concentrated in vacuo. The resulting crude orange solid (13.99g) was dissolved in CH_2Cl_2 (310 mL) and **1** (5.60g, 44 mmol) was added before quickly adding 1,8-diazabicyclo[5.4.0]undec-7-ene (7.1 mL, 48mmol). The orange reaction mixture quickly turned into a dark brown solution.

After 30 min, the reaction mixture was dropwise added into a vigorously stirred 10% citric acid aq. solution (750mL). Next, pentane (400 mL) was added into the mixture, a brown precipitate was observed. The mixture was filtered, the solid was washed with water and CH_2Cl_2, redissolved in EtOH and concentrated in vacuo to give **4**.

Yield: 8.49 g (49% over three steps). Isolated as a brown solid. >95% pure by NMR and a single spot by TLC

R$_f$: 0.60 in EtOAc. Stains yellow with $KMnO_4$ stain.

LC-MS-ESI (m/z): Calcd for $C_{14}H_{14}BrN_3O_5$ $[M + H]^+$: 384.01, Found 384.15

^{1}H NMR (400 MHz, DMSO): δ 9.76 (s, 1H), 8.19 (s, 1H), 8.12 (s, 1H), 6.92 (t, *J* = 8.2 Hz, 1H), 6.37 (d, *J* = 8.2 Hz, 2H), 4.49 (s, 2H), 4.14 (q, *J* = 7.1 Hz, 2H), 1.20 (t, *J* = 7.1 Hz, 3H).

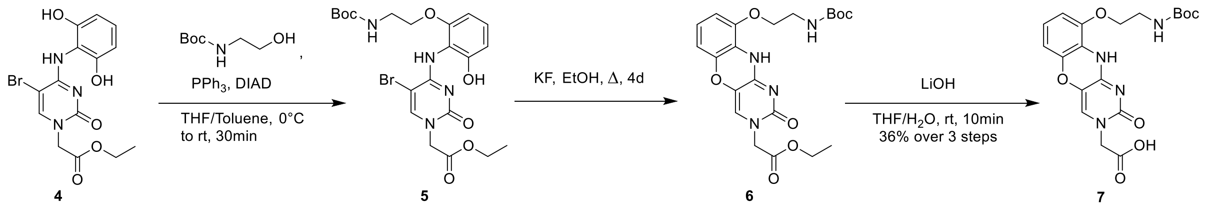

2-(9-(2-((tert-butoxycarbonyl)amino)ethoxy)-2-oxo-2,10-dihydro-3H-benzo[b]pyrimido[4,5-e][1,4]oxazin-3-yl)acetic acid (7). The compound was synthesized with slight modification of the previously reported procedure.[2] To a stirred solution of **4** (8.48 g, 21.0 mmol), N-Boc-ethanolamine (3.76 g, 23.1 mmol) and triphenyl phosphine (8.26 g, 31.5 mmol) in THF (156mL), 54 mL of toluene were added, and the mixture was sonicated for 15min. At 0°C, diisopropyl azodicarboxylate (6.25 mL, 31.5 mmol) was quickly added and the reaction was left to warm to room temperature. After 30min, the reaction mixture was partitioned between 10% citric acid aq. solution (250 mL) and EtOAc (250mL). The organic layer was separated, and the aqueous layer was extracted twice more with EtOAc (2x250 mL). Combined organic extracts were dried over anhydrous $MgSO_4$, filtered, and concentrated in vacuo. The resulting crude residue was purified by column chromatography (50-100% EtOAc/pentane) to give a mixture of **5** and O=PPh3, due to coelution, as a brown oil. The resulting oil (16.0g) was dissolved in EtOH (1 L), potassium fluoride (12.32 g, 210 mmol) was added, and the mixture was heated to reflux for 4 days. Then, the mixture was filtered through a short silica path, washing with some EtOAc and the filtrate was concentrated in vacuo. The resulting brown oil (15.5g) were dissolved in THF (47 mL) and a solution of LiOH (1

g, 42 mmol) in water (23 mL) was dropwise added. After 5 min, the reaction mixture was divided evenly into 8 x 50 mL FALCON centrifuge tubes before adding 10% citric acid aq. solution (40 mL) into each of them. A yellow precipitate was observed, and the tubes were centrifuged at 4°C, x11000 rpm, 10min. The supernatant was discarded, the yellow pellets were washed 3 times with diethyl ether (3x40 mL each tube) using the same centrifugation and supernatant dispose-off strategy. The pellets were dissolved in MeOH, combined, and concentrated in vacuo to give **7**.

Yield: 3.16 g (36% over three steps). Isolated as a yellow solid. >95% pure by NMR.

LC-MS-ESI (m/z): Calcd for $C_{19}H_{22}N_4O_7$ $[M + H]^+$: 419.15, Found 419.02

^{1}H NMR (400 MHz, DMSO): δ 9.87 (br. s, 1H), 7.49 (s, 1H), 7.37 (m, 1H), 6.80 (t, *J* = 8.4 Hz, 1H), 6.61 (dd, *J* = 8.4, 1.1 Hz, 1H), 6.46 (dd, *J* = 8.4, 1.1 Hz, 1H), 4.33 (s, 2H), 3.89 (t, *J* = 4.9 Hz, 2H), 3.47 – 3.13 (m, water + 2H ethanolamine chain), 1.40 (s, 9H).

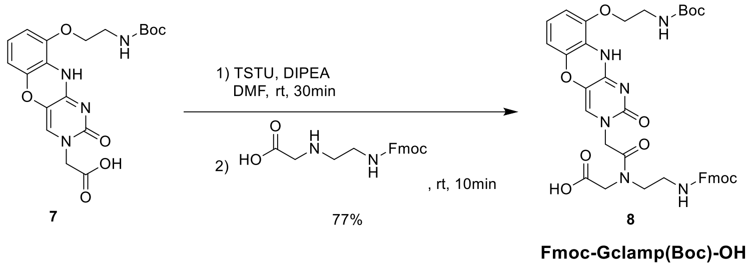

Fmoc-Gclamp(Boc)-OH (8). The compound was synthesized with slight modification of the previously reported procedure.[2] To a stirred solution of acid **7** (1 g, 2.32 mmol) together with DIPEA (526 µL, 3.02 mmol) in DMF (7 mL), TSTU (720 mg, 2.32 mmol) was added. After 30 min at room temperature, N[N'-fluorenylmethyloxycarbonyl-(2'-aminoethyl)]glycine (720 mg, 2.32 mmol) followed by DIPEA (1.151 mL, 6.61 mmol) and the mixture was sonicated for 10 min until a clear brown solution was obtained. Then, the reaction mixture was divided evenly into 4 x 50 mL FALCON centrifuge tubes before adding 10% citric acid aq. solution (40 mL) into each of them. A yellow precipitate was observed, and the tubes were centrifuged at 24°C, x11000 rpm, 10min. The supernatant was discarded, the yellow pellets were washed 2 times with diethyl ether (2x 40 mL each tube) and 2 times with THF/H_2O 1:3 (2x 40 mL each tube) using the same centrifugation and supernatant dispose-off strategy. The pellets were dissolved in 1:1 CH_3CN/H_2O, combined, and lyophilized to give **8**.

LC-MS-ESI (m/z): Calcd for $C_{38}H_{40}N_6O_{10}$ $[M + H]^+$: 741.28, Found 741.13

Yield: 1.51 g (77%). Isolated as a yellow lyophilized powder. >90% pure by LC-MS.

^{1}H NMR (500 MHz, DMSO): δ 9.83 (br. s, 1H), 7.88 (dd, 2H), 7.68 (q, 2H), 7.43 – 7.29 (m, 6H), 6.80 (t, 1H), 6.60 (dd, 1H), 6.43 (m, 1H), 4.71 – 4.14 (m, 6H), 3.99 (s, 1H), 3.89 (t, 2H), 3.43 – 3.04(m, water + *n*H), 1.39 (s, *J* = 3.2 Hz, 9H).

^{13}C NMR (126 MHz, DMSO): δ 171.7, 171.3, 171.0, 170.3, 168.5, 168.0, 156.8, 156.6, 155.9, 154.6, 146.7, 144.4, 142.8, 141.2, 141.2, 128.1, 127.5, 126.2, 125.6, 125.6, 123.6, 120.6, 108.3, 108.3, 107.4, 78.4, 68.7, 66.0, 65.9, 49.6, 49.3, 48.2, 47.4, 47.2, 40.6, 40.5, 40.4, 40.3, 40.2, 40.1, 40.1, 40.0, 39.9, 39.8, 39.6, 39.5, 39.1, 38.4, 28.7.

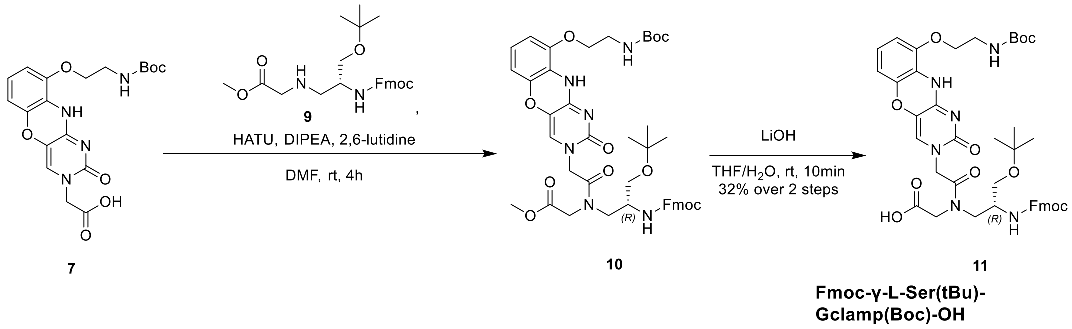

Fmoc-γ-L-Ser(tBu)-Gclamp(Boc)-OH (11). To a stirred solution of acid **7** (2.16 g, 5.0 mmol), 2,6-lutidine (580 µL, 5.0 mmol) and DIPEA (1.045 mL, 6.0 mmol) in DMF (15 mL), HATU (1.96g, 5.0 mmol) were added. After 30 min, a solution of amine **9** (2.45g, 5.5 mmol) and DIPEA (1.132 mL, 6.5 mmol) in DMF (10mL) was added in one portion. After 4 h, the reaction mixture was partitioned between 10% citric acid aq. solution (250 mL) and CH_2Cl_2 (250 mL). The organic layer was separated, and the aqueous layer was extracted once more with CH_2Cl_2 (250 mL). Combined organic extracts were washed with brine, dried over anhydrous Na_2SO_4, filtered, and concentrated in vacuo. The resulting crude residue was purified by column chromatography (0-10% EtOH/EtOAc) to give a mixture of **10** and impurities, due to coelution, as a brown oil. The resulting oil (5.1g) was dissolved in THF (17 mL) and a solution of LiOH (240 mg, 10 mmol) in water (8 mL) was dropwise added. After 15 min, the reaction mixture was divided evenly into 4 x 50 mL FALCON centrifuge tubes before adding 10% citric acid aq. solution (40 mL) into each of them. A yellow precipitate was observed, and the tubes were centrifuged at 4°C, x11000 rpm, 10min. The supernatant was discarded, the yellow pellets were washed with diethyl ether (1x40 mL each tube) and 3 times with THF/H_2O 1:3 (3x 40 mL

each tube) using the same centrifugation and supernatant dispose-off strategy. The pellets were dissolved in MeOH/water, combined, and lyophilized to give **11**.

Yield: 1.39 g (32% over two steps). Isolated as a yellow lyophilized powder. >95% pure by LC-MS.

LC-MS-ESI (m/z): Calcd for $C_{43}H_{50}N_6O_{11}$ $[M + H]^+$: 827.35, Found 827.21

^{1}H NMR (500 MHz, DMSO): Rotameric mixture. δ 9.82 (br. s, 1H), 7.91 – 7.86 (d, 2H), 7.70 (t, *J* = 6.3 Hz, 2H), 7.45 – 7.25 (m, 6H), 6.78 (m, 1H), 6.64 – 6.57 (d, 1H), 6.41 (m, 1H), 4.63 (m, 1H), 4.48 – 4.15 (m, 4H), 4.11 – 3.67 (m, 5H), 3.44-3.06 (m, water + nH_2O), 1.40 (s, 9H), 1.17 – 1.07 (d, 9H).

^{13}C NMR (126 MHz, DMSO): Rotameric mixture. δ 170.7, 170.3, 168.3, 167.8, 155.9, 155.8, 155.7, 155.5, 154.1, 146.2, 144.0, 143.9, 143.8, 143.8, 142.3, 140.7, 128.9, 127.6, 127.6, 127.3, 127.1, 125.2, 125.2, 123.1, 121.4, 120.1, 120.1, 120.0, 115.6, 109.8, 107.8, 107.0, 77.9, 72.8, 72.5, 68.3, 65.5, 65.4, 61.5, 61.1, 50.9, 50.2, 49.5, 48.9, 48.5, 48.1, 48.0, 46.8, 46.7, 28.3, 27.3, 27.2.

5.2. Synthesis of PNA monomer Fmoc-*N*-7-Me-G(Boc)-OH and Fmoc-γ-L-Ser(tBu)-*N*-7-Me-G(Boc)-OH

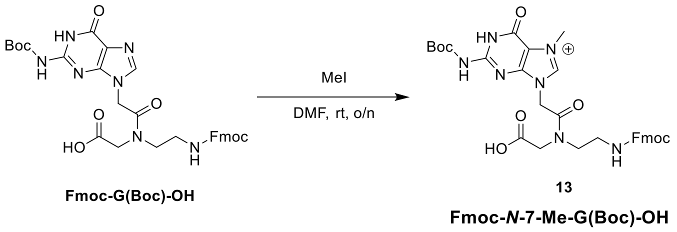

Fmoc- *N*-7-Me-G(Boc)-OH (13). The compound was synthesized with slight modification of the previously reported procedure.[3] To a stirred solution of PNA monomer Fmoc-G(Boc)-OH (1g, 1.58 mmol) in DMF (11.7 mL), iodomethane was added dropwise (2 mL, 31.60 mmol) and left stirring overnight. Then, the reaction mixture was divided evenly into 4 x 50 mL FALCON centrifuge tubes before adding diethyl ether (40 mL) into each of them. A white precipitate was observed, and the tubes were centrifuged at 4°C, x11000 rpm, 10min. The supernatant was discarded, the orange oil was sonicated with water (5 mL) and scratched with a spatula for 10min until a beige solid was formed. The tubes were again centrifuged at 4°C, x11000 rpm, 10min. The supernatant was discarded, the pellets were dissolved in CH_3CN/water, combined, and lyophilized to give **13**.

Yield: 956mg (93%). Isolated as a beige lyophilized powder. >95% purity by LC-MS. Methylation at *N*-7 position of the guanine moiety confirmed by the ^{1}H-NMR shift of the H at the C_8 position which showed a deshielding shift up to 9.38 ppm in accordance with previous reported data.[3]

LC-MS-ESI (m/z): Calcd for $C_{32}H_{36}N_7O_8$ $[M]^+$: 646.26, Found 646.01

^{1}H NMR (400 MHz, DMSO): Rotameric mixture. δ 9.38 (s, 1H), 7.86 (d, 2H), 7.63 (dd, 2H), 7.45 (m, 1H), 7.39 (t, 2H), 7.30 (t, 2H), 5.33 (s, 1H), 5.13 (s, 1H), 4.38 (d, 1H), 4.30 (s, 1H), 4.23 (t, 2H), 4.13 (s, 3H), 4.05 (m, 3H), 3.95 – 2.94 (br. m, water an *n*H), 1.46 (d, 9H).

^{13}C NMR (101 MHz, DMSO): Rotameric mixture. δ 170.7, 170.2, 165.4, 164.8, 156.5, 156.2, 153.3, 153.2, 152.5, 152.2, 151.2, 151.1, 147.8, 143.8, 143.8, 140.8, 140.7, 140.7, 140.6, 127.6, 127.1, 127.0, 125.1, 125.0, 120.1, 109.9, 109.7, 83.2, 83.1, 65.5, 53.6, 49.5, 48.2, 47.3, 47.0, 46.8, 46.7, 45.5, 45.4, 37.8, 35.9, 27.7.

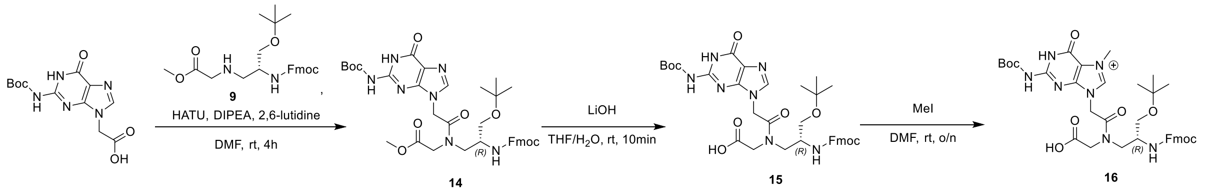

Fmoc-γ-L-Ser(tBu)-*N*-7-Me-G(Boc)-OH

Fmoc-γ-L-Ser(tBu)-*N*-7-Me-G(Boc)-OH (16). To a stirred solution of N2-Boc-guanine-9-acetic acid (1.37 g, 4.38 mmol), 2,6-lutidine (507 µL, 4.38 mmol) and DIPEA (881 µL, 5.06 mmol) in DMF (14 mL), HATU (1.72 g, 4.38 mmol) was added. After 1h, a solution of 9 (1.50 g, 3.37 mmol) with DIPEA (939 µL, 5.39 mmol) in DMF (20 mL) was added. After 4h, the reaction mixture was partitioned between 10% citric acid aq. solution (150 mL) and CH_2Cl_2 (150 mL). The organic layer was separated, and the aqueous layer was extracted twice more with CH_2Cl_2 (2x 150 mL). Combined organic extracts were washed with brine, dried over anhydrous Na_2SO_4, filtered, and concentrated in vacuo. The resulting crude residue was purified by column chromatography (0-25% EtOH/EtOAc) to give a mixture of **14** and impurities, due to coelution, as a brown oil. The resulting oil (6.2 g) was dissolved in THF (7 mL) and a and solution of LiOH (161 mg, 6.74 mmol) in water (4 mL) was dropwise added. After 15 min, the reaction mixture was divided evenly into 4 x 50 mL FALCON centrifuge tubes before adding 10% citric acid aq. solution (40 mL) into each of them. A white precipitate was observed, and the tubes were centrifuged at 4°C, x11000 rpm, 10min. The supernatant was discarded, the pellets were dissolved in MeOH/water, combined, and concentrated in vacuo. The resulting crude white

foam of **15** was directly dissolved in DMF (10 mL) and iodomethane (1.64 mL, 26.3 mmol) was dropwise added, stirring the mixture overnight. Then, the reaction mixture was divided evenly into 4 x 50 mL FALCON centrifuge tubes before adding diethyl ether (40 mL) into each of them. A white precipitate was observed, and the tubes were centrifuged at 4°C, x11000 rpm, 10min. The supernatant was discarded, the orange oil was sonicated with water (5 mL) and scratched with a spatula for 10min until a beige solid was formed. The tubes were again centrifuged at 4°C, x11000 rpm, 10min. The supernatant was discarded, the pellets were dissolved in CH_3CN/water, combined, and lyophilized to give **16**.

Yield: 571 mg (23%). Isolated as a beige lyophilized powder. >95% pure by NMR and LC-MS. Methylation at *N*-7 position of the guanine moiety confirmed by the ^{1}H-NMR shift of the H at the C_8 position which showed a deshielding shift up to 9.37 ppm in accordance with previous reported data.[3]

LC-MS-ESI (m/z): Calcd for $C_{37}H_{46}N_7O_9^+$ $[M]^+$: 732.34, Found 732.16

^{1}H NMR (500 MHz, DMSO): Rotameric mixture. δ 9.37 (d, 0.5H), 9.14 (d, 0.5H), 7.92 – 7.02 (m, 11H), 5.47 – 4.97 (m, 2H), 4.48 – 3.97 (m, 11H), 3.82– 3.05 (m, water + *n*H), 1.19 – 1.05 (m, 18H).

^{13}C NMR (126 MHz, DMSO): Rotameric mixture. δ 171.0, 170.4, 166.2, 166.0, 165.8, 165.6, 156.7, 156.4, 156.3, 156.3, 156.1, 156.1, 153.9, 153.8, 153.6, 153.4, 150.3, 150.3, 148.2, 144.4, 144.3, 144.3, 144.2, 144.0, 144.0, 143.0, 141.2, 141.2, 141.2, 141.1, 139.9, 139.6, 139.5, 137.9, 129.4, 128.1, 128.1, 128.1, 128.0, 127.8, 127.5, 127.4, 127.4, 127.3, 125.7, 125.7, 125.6, 125.6, 125.5, 121.9, 120.7, 120.7, 120.6, 120.6, 120.5, 110.4, 110.3, 110.1, 107.2, 107.1, 73.4, 73.0, 66.0, 66.0, 65.9, 65.8, 62.1, 61.9, 61.7, 51.9, 51.7, 50.8, 50.6, 50.2, 49.4, 49.3, 49.1, 48.9, 48.8, 48.7, 47.2, 47.1, 45.9, 45.8, 45.7, 45.4, 36.3, 36.2, 36.1, 36.0, 31.8, 28.2, 28.0, 27.7.

5.3. Synthesis of chiral PNA backbones

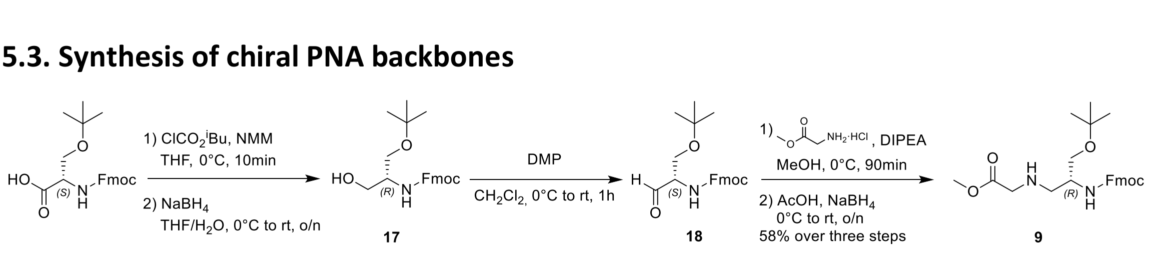

Methyl (R)-(2-((((9H-fluoren-9-yl)methoxy)carbonyl)amino)-3-(tert-butoxy)propyl)glycinate (9). The compound was synthesized with slight modification of the previously reported procedures.[4] To a stirred solution of Fmoc-L-Ser(tBu)-OH (15.49 g, 40 mmol) and NMM (4.4

mL, 40 mmol) in THF (235 mL) at 0°C, isobutyl chloroformate (6 mL, 44 mmol) was added in one portion. The formation of a white precipitate was observed. After 10 min, the reaction mixture was cold filtered and quickly a solution sodium borohydride (3.0 g, 80 mmol) in water (25 mL) was added at 0°C. The reaction mixture was left to warm to room temperature and was stirred overnight. Then, the reaction mixture was partitioned between sat. NH_4Cl aq. solution (300 mL) and EtOAc (300 mL). The organic layer was separated, and the aqueous layer was extracted twice more with EtOAc (2x300 mL). Combined organic extracts were washed with brine, dried over anhydrous Na_2SO_4, filtered, and concentrated in vacuo. To give a crude white foam of **17** (16.02 g), which was dissolved in CH_2Cl_2 (267 mL) and DMP (25.45 g, 60 mmol) was added in one portion at 0°C. After 1h, the reaction mixture was quenched by the addition of a sat. $Na_2S_2O_3$ aq. solution (40 mL) and sat. $NaHCO_3$ aq. solution (40 mL). Then, the mixture was partitioned between CH_2Cl_2 (500 mL) and water (500 mL). The organic layer was separated, and the aqueous layer was extracted twice more with CH_2Cl_2 (2x250mL). Combined organic extracts were washed with brine, dried over anhydrous Na_2SO_4, filtered, and concentrated in vacuo (not to dryness, keeping the water bath at 25°C to avoid racemization). To give a crude oil of **18** (32 g) was dissolved in MeOH (490 mL) and, a solution of glycine methyl ester hydrochloride (10.04g, 80 mmol) and DIPEA (13 mL, 75 mmol) in MeOH (60 mL) was added at 0°C. After 90 min, acetic acid (7.39 mL, 129 mmol) and sodium cyanoborohydride (4.02 g, 64 mmol) were added at 0°C, letting the reaction mixture to warm to room temperature. The mixture was left stirring overnight, then the reaction mixture was partitioned between sat. $NaHCO_3$ aq. solution (500 mL) and EtOAc (1 L). The organic layer was separated, and the aqueous layer was extracted once more with EtOAc (1 L). Combined organic extracts were washed with brine, dried over anhydrous Na_2SO_4, filtered, and concentrated in vacuo. The resulting crude residue was purified by column chromatography (50-100% EtOAc/pentane) to give **9**.

Yield: 10.28 g (58% over three steps). Isolated as a yellow gum. >95% pure by NMR and a single spot by TLC. >99% ee by SFC-OD-H 2% MeOH with R_t=8.36min.

R_f: 0.66 in EtOAc. Stains yellow with $KMnO_4$ stain.

LC-MS-ESI (m/z): Calcd for $C_{25}H_{32}N_2O_5$ $[M+H]^+$: 441.23, Found 441.14

^{1}H NMR (400 MHz, $CDCl_3$): δ 7.76 (d, 2H), 7.61 (d, 2H), 7.39 (t, 2H), 7.36 – 7.25 (m, 2H), 5.57 (br. d, 1H), 4.38 (br. m, 2H), 4.24 (t, 1H), 3.86 (br. s, 1H), 3.73 (s, 3H), 3.57 – 3.19 (m, 5H), 2.88 (br. m, 2H), 1.18 (s, 9H).

^{13}C NMR (101 MHz, $CDCl_3$): δ 172.3, 156.5, 144.2, 144.1, 141.4, 127.8, 127.2, 125.3, 120.1, 73.4, 66.9, 62.3, 52.1, 50.8, 50.5, 50.4, 47.4, 27.5.

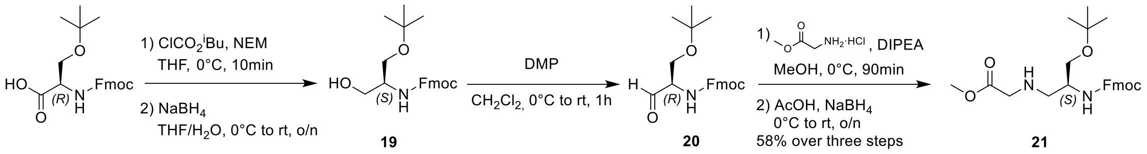

Methyl (S)-(2-((((9H-fluoren-9-yl)methoxy)carbonyl)amino)-3-(tert-butoxy)propyl)glycinate (21). The compound was synthesized with slight modification of the previously reported procedures.[4] To a stirred solution of Fmoc-D-Ser(tBu)-OH (500 mg, 1.30 mmol) and NEM (186 µL, 1.30 mmol) in THF (7.7 mL) at 0°C, isobutyl chloroformate (186 µL, 44 mmol) was added in one portion. The formation of a white precipitate was observed. After 10 min, the reaction mixture was cold filtered and quickly a solution sodium borohydride (99 mg, 2.60 mmol) in water (1 mL) was added at 0°C. The reaction mixture was left to warm to room temperature and was stirred overnight. Then, the reaction mixture was partitioned between sat. NH_4Cl aq. solution (25 mL) and EtOAc (25 mL). The organic layer was separated, and the aqueous layer was extracted twice more with EtOAc (2x25 mL). Combined organic extracts were washed with brine, dried over anhydrous Na_2SO_4, filtered, and concentrated in vacuo. To give a crude white foam of 1**9** (16.02 g), which was dissolved in CH_2Cl_2 (8.7 mL) and DMP (827 mg, 1.95 mmol) was added in one portion at 0°C. After 1h, the reaction mixture was quenched by the addition of a sat. $Na_2S_2O_3$ aq. solution (4 mL) and sat. $NaHCO_3$ aq. solution (4 mL). Then, the mixture was partitioned between CH_2Cl_2 (25 mL) and water (25 mL). The organic layer was separated, and the aqueous layer was extracted twice more with CH_2Cl_2 (2x25 mL). Combined organic extracts were washed with brine, dried over anhydrous Na_2SO_4, filtered, and concentrated in vacuo (not to dryness, keeping the water bath at 25°C to avoid racemization). To give a crude oil of **20** (684 mg) was dissolved in MeOH (16 mL) and, a solution of glycine methyl ester hydrochloride (326 mg, 2.60 mmol) and DIPEA (420 µL, 2.41 mmol) in MeOH (2 mL) was added at 0°C. After 90 min, acetic acid (240 µL, 4.20 mmol) and sodium cyanoborohydride (176 mg, 2.08 mmol) were added at 0°C, letting the reaction mixture to warm to room temperature. The mixture was left stirring overnight, then the reaction mixture was partitioned between sat. $NaHCO_3$ aq. solution (50 mL) and EtOAc (100 mL). The organic layer was separated, and the aqueous layer was extracted once more with EtOAc (100 mL). Combined organic extracts were washed with brine, dried over anhydrous Na_2SO_4, filtered,

and concentrated in vacuo. The resulting crude residue was purified by column chromatography (50-100% EtOAc/pentane) to give **21**.

Yield: 294 mg (51% over three steps). Isolated as a yellow gum. >95% pure by NMR and a single spot by TLC. >99% ee by SFC-OD-H 2% MeOH with R_t=9.05min.

R_f: 0.66 in EtOAc. Stains yellow with $KMnO_4$ stain.

LC-MS-ESI (m/z): Calcd for $C_{25}H_{32}N_2O_5$ $[M+H]^+$: 441.23, Found 441.09

^{1}H NMR (400 MHz, $CDCl_3$): δ 7.80 – 7.72 (m, 2H), 7.65 – 7.55 (m, 2H), 7.39 (m, 2H), 7.31 (m, 2H), 5.74 (br. d, 1H), 4.37 (br. m, 2H), 4.24 (t, 1H), 3.97 – 3.87 (br. s, 1H), 3.74 (s, 3H), 3.69 – 3.32 (m, 4H), 2.98 (br. m, 2H), 1.19 (s, 9H).

^{13}C NMR (101 MHz, $CDCl_3$): δ 171.2, 156.6, 144.2, 144.1, 141.4, 127.8, 127.2, 125.4, 120.1, 73.7, 67.0, 62.5, 52.4, 51.0, 50.0, 49.9, 47.3, 27.5.

6. Characterization of PNA oligomers

PNA-S-0: C-Ter: TGT<u>T</u>G<u>A</u>-Lys(Atto647N)-Ac

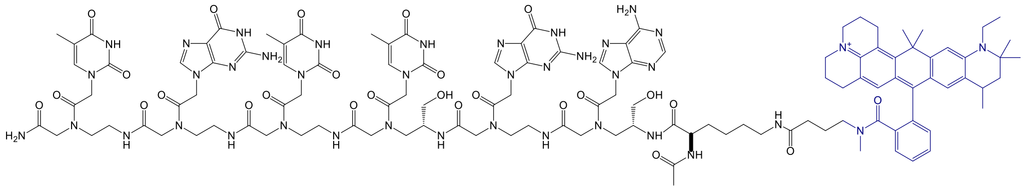

Chemical Formula: $C_{118}H_{152}N_{39}O_{26}^{+}$ **Exact Mass:** 2531.18, **Molecular Weight:** 2532.76

LC-MS (ESI): RT=2.18min. Absorbance at λ=260 nm. *m/z* expected for $[M+2H]^{3+}$: 844.73, *m/z* observed: 844.67.

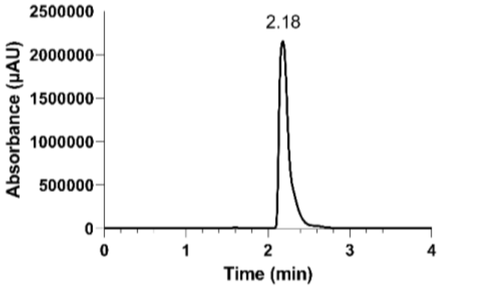

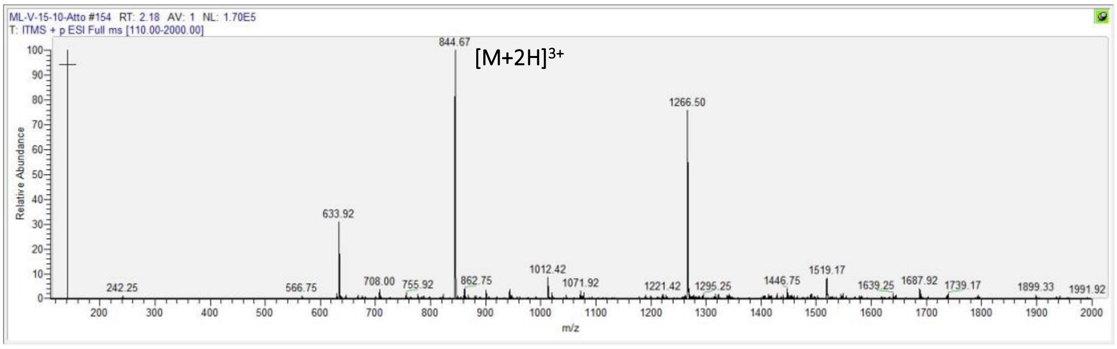

Chemical Formula: $C_{118}H_{152}N_{39}O_{26}^{+}$

MALDI-TOF: *m/z* calc: 2532.18 $[M]^{+}$ *m/z* found: 2532.52

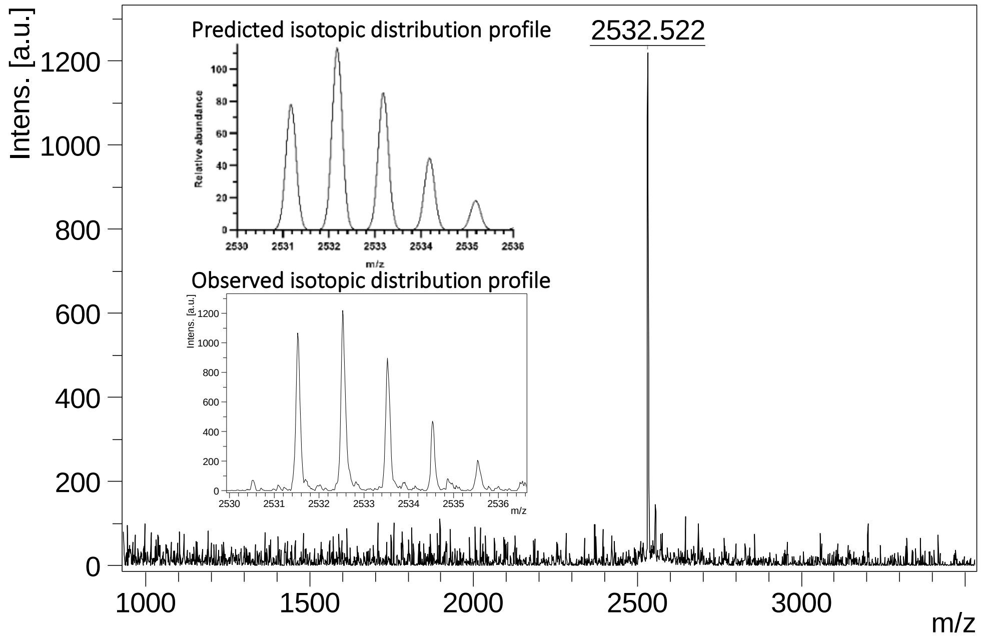

PNA-A-0: C-Ter: Lys(<u>T</u>C<u>A</u>ACA-Ac)-Cy3

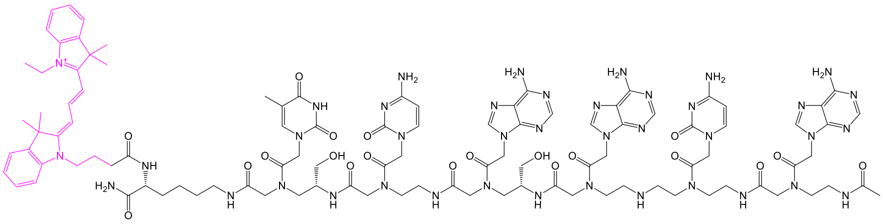

Chemical Formula: $C_{104}H_{137}N_{40}O_{20}^{+}$ **Exact Mass:** 2266.09 **Molecular Weight:** 2267.50

LC-MS (ESI): RT=1.84min. Absorbance at λ=260 nm. *m/z* expected for $[M+2H]^{3+}$: 756.37, *m/z* observed: 756.33.

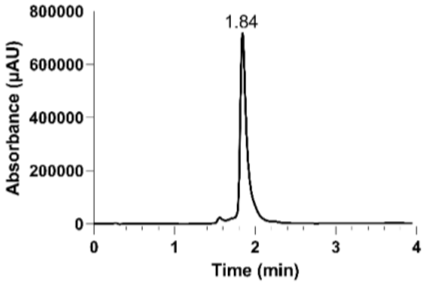

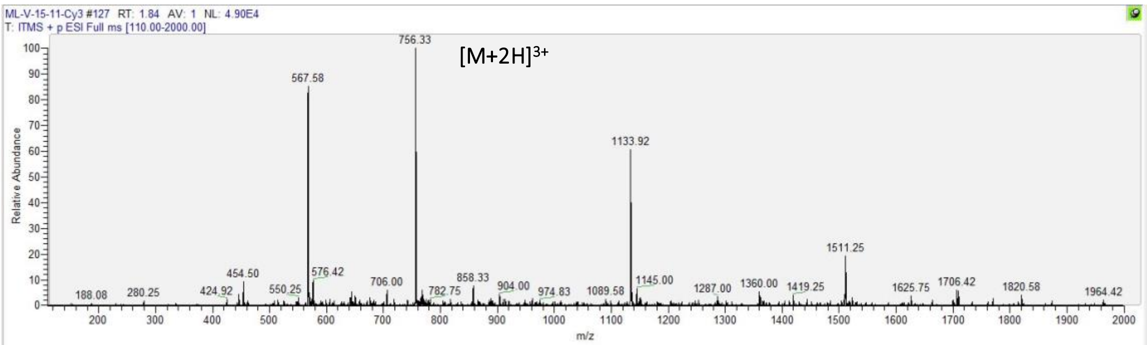

Chemical Formula: $C_{104}H_{137}N_{40}O_{20}^{+}$

MALDI-TOF: *m/z* calc: 2267.10 [M]$^+$ *m/z* found: 2266.80

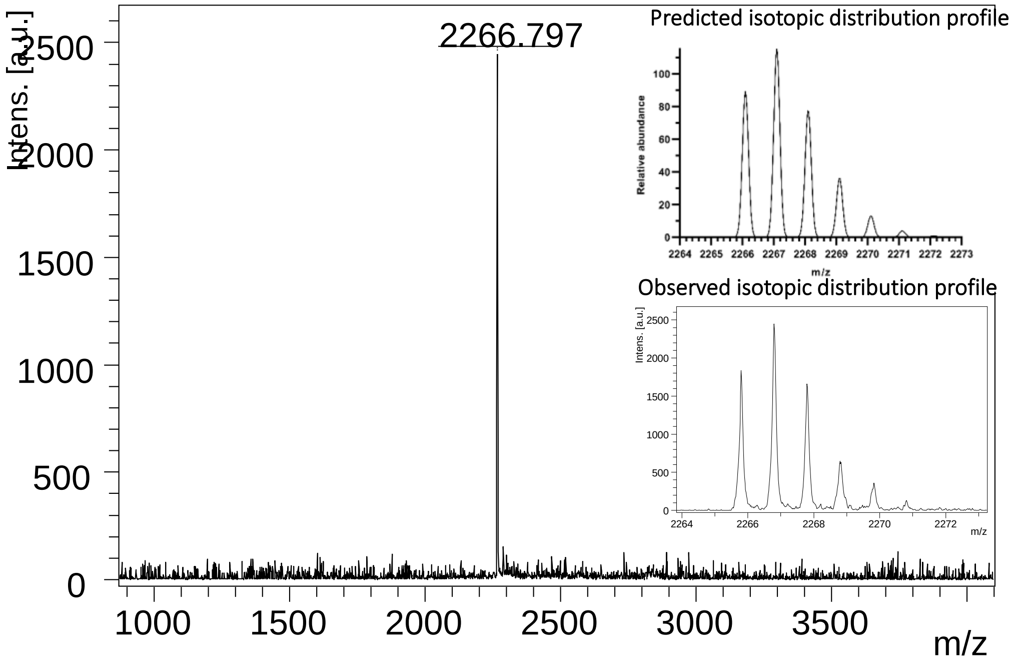

PNA-S-1: C-Ter: TGT̲TGA̲-Lys(Atto647N)-Ac (Orange=N-7-MeG)

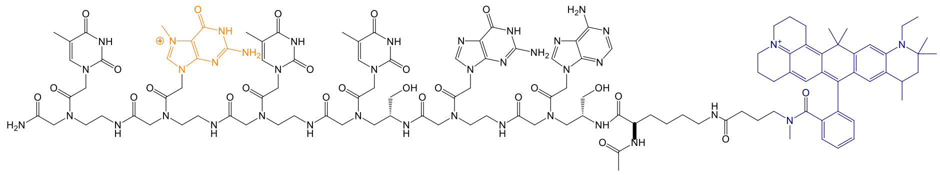

Chemical Formula: $C_{119}H_{155}N_{39}O_{26}^{2+}$ **Exact Mass:** 2546.20 **Molecular Weight:** 2547.80

LC-MS (ESI): RT=2.16min. Absorbance at λ=260 nm. *m/z* expected for $[M+H]^{3+}$: 849.40, *m/z* observed: 849.50.

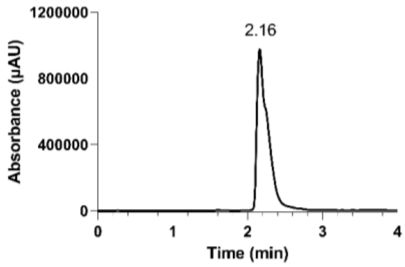

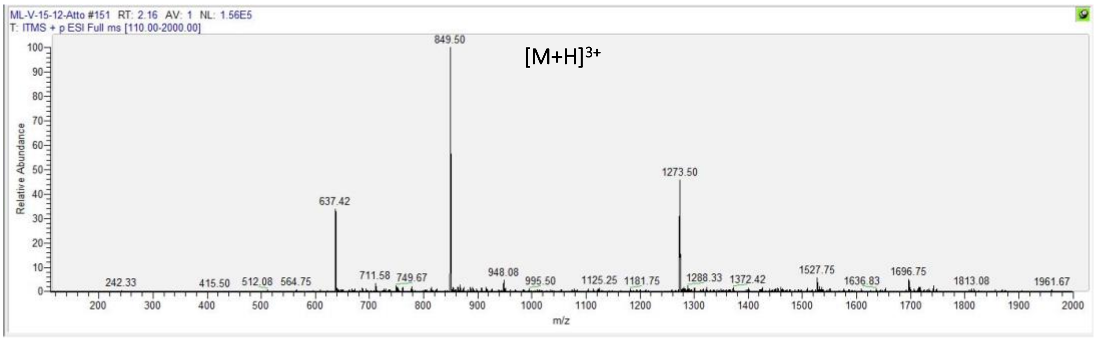

Chemical Formula: $C_{119}H_{155}N_{39}O_{26}^{2+}$

MALDI-TOF: *m/z* calc: 2546.21 $[M-H]^{+}$ *m/z* found: 2546.60

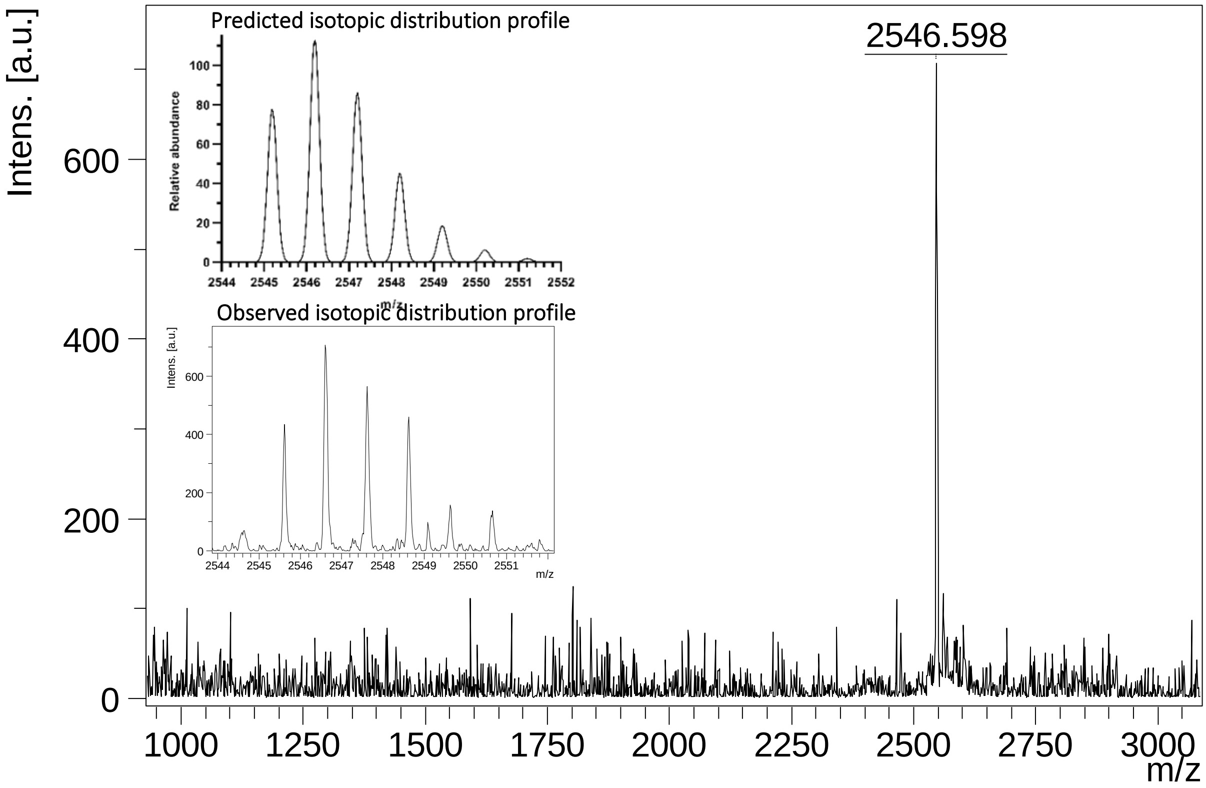

PNA-A-1: C-Ter: Lys(TCAACA-Ac)-Cy3 (Red=G-clamp)

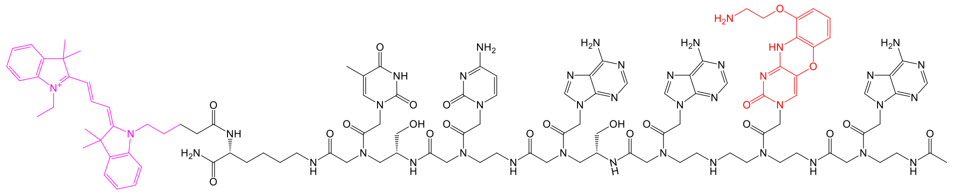

Chemical Formula: $C_{112}H_{144}N_{41}O_{22}^{+}$ **Exact Mass:** 2415.14 **Molecular Weight:** 2416.65

LC-MS (ESI): RT=1.83min. Absorbance at λ=260 nm. *m/z* expected for $[M+3H]^{4+}$: 604.29, *m/z* observed: 604.75.

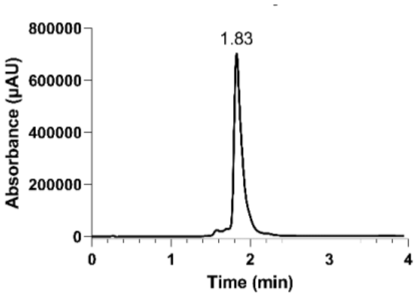

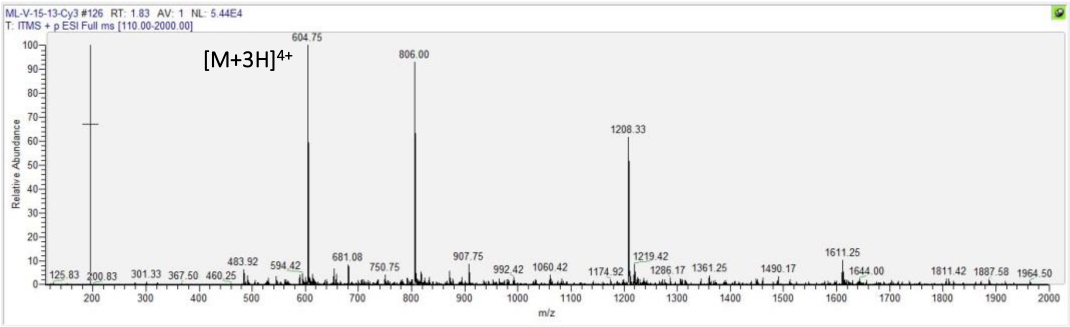

Chemical Formula: $C_{112}H_{144}N_{41}O_{22}^{+}$

MALDI-TOF: *m/z* calc: 2416.14 $[M]^{+}$ *m/z* found: 2416.00

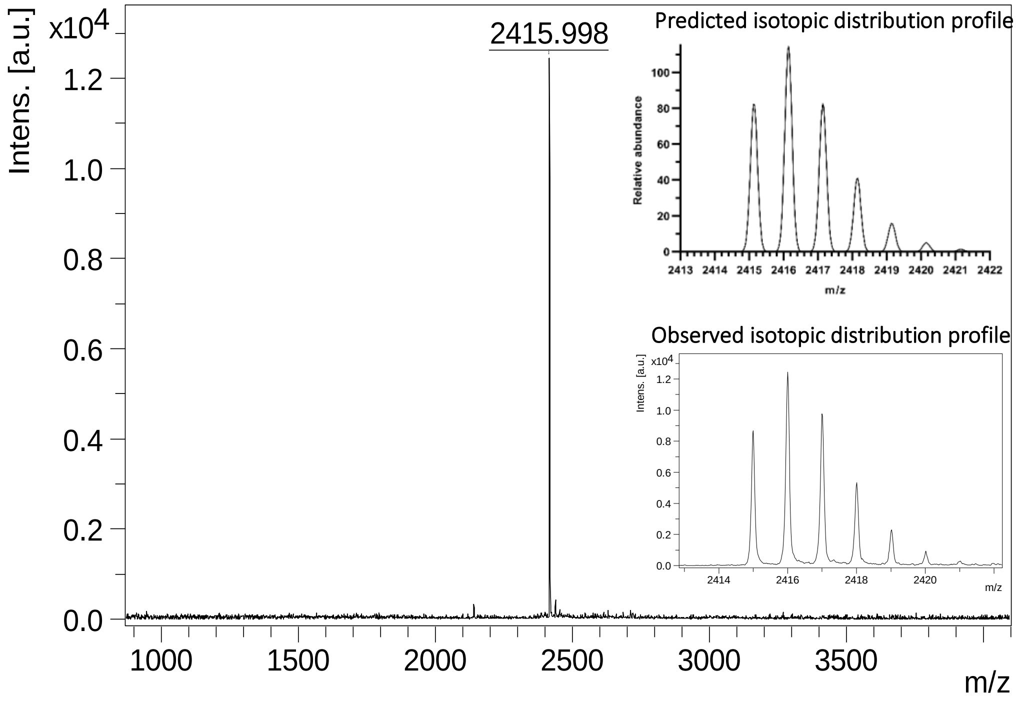

PNA-S-2: C-Ter: TGT<u>T</u>G<u>A</u>-Lys(Atto647N)-Ac (Orange=N-7-MeG)

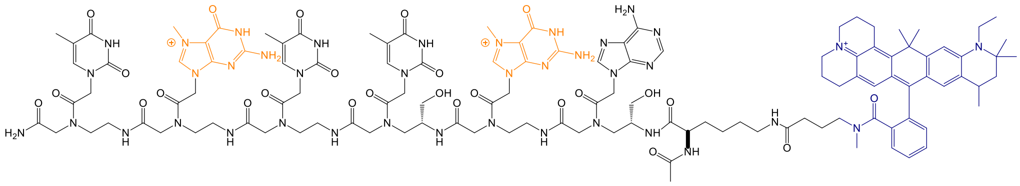

Chemical Formula: $C_{120}H_{158}N_{39}O_{26}^{3+}$ **Exact Mass:** 2561.22 **Molecular Weight:** 2562.83

LC-MS (ESI): RT=2.16min. Absorbance at λ=260 nm. *m/z* expected for $[M]^{3+}$: 854.08, *m/z* observed: 854.08.

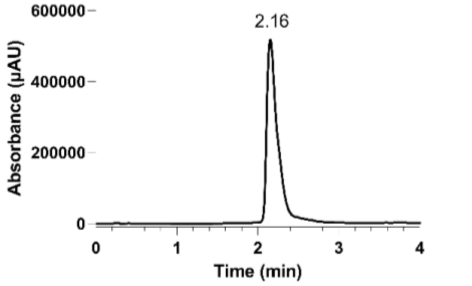

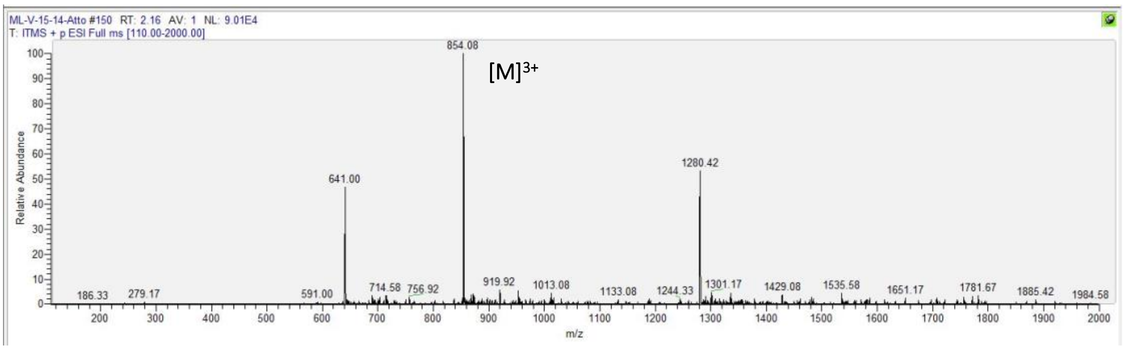

Chemical Formula: $C_{120}H_{158}N_{39}O_{26}^{3+}$

MALDI-TOF: *m/z* calc: 2560.21 $[M-2H]^{+}$ *m/z* found: 2560.68

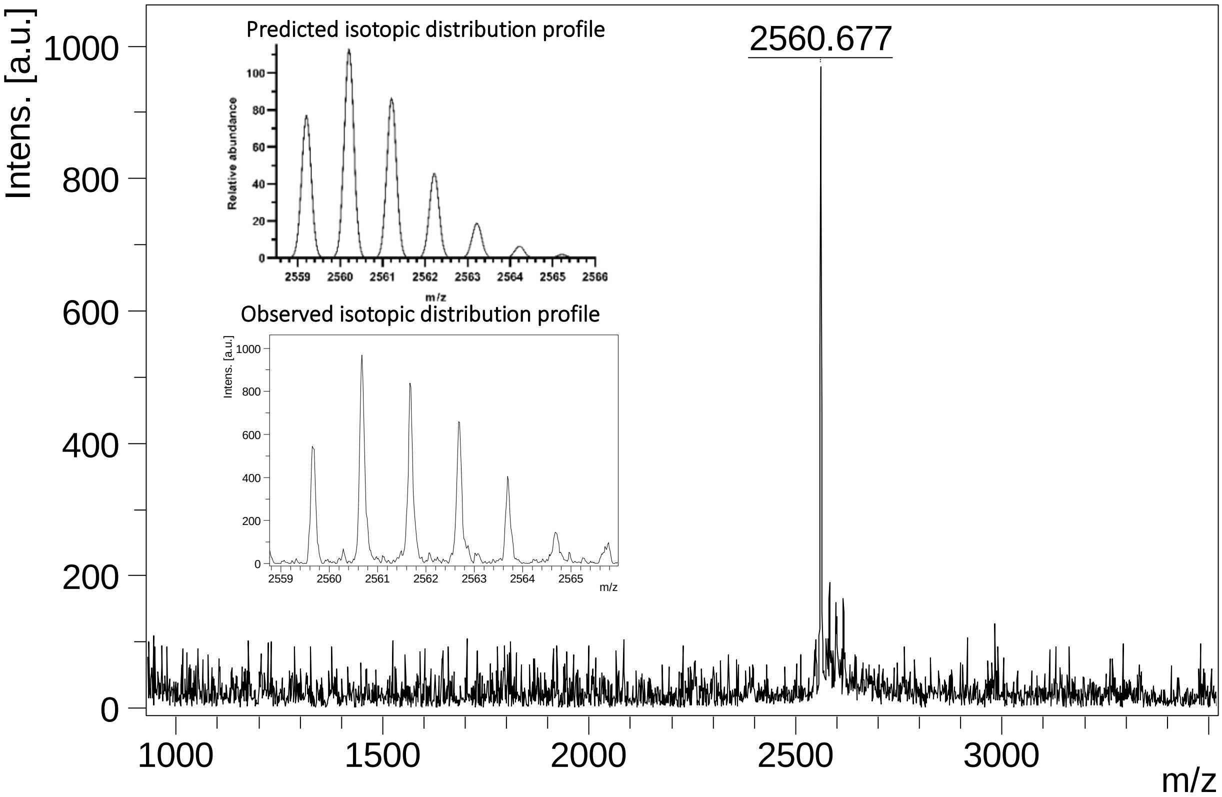

PNA-A-2: C-Ter: Lys(TCAACA-Ac)-Cy3 (Red=G-clamp)

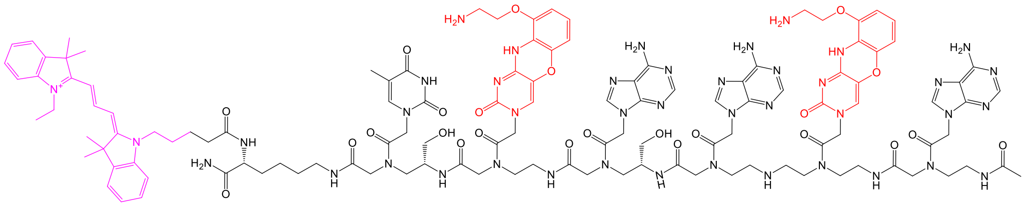

Chemical Formula: $C_{120}H_{151}N_{42}O_{24}^{+}$ **Exact Mass:** 2564.19 **Molecular Weight:** 2565.80

LC-MS (ESI): RT=1.81min. Absorbance at λ=260 nm. *m/z* expected for $[M+3H]^{4+}$: 642.05, *m/z* observed: 642.00.

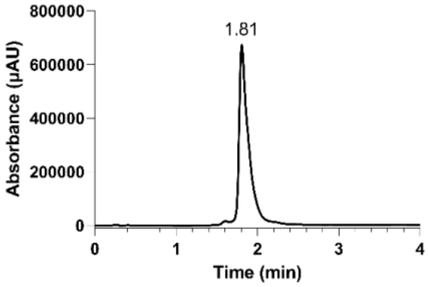

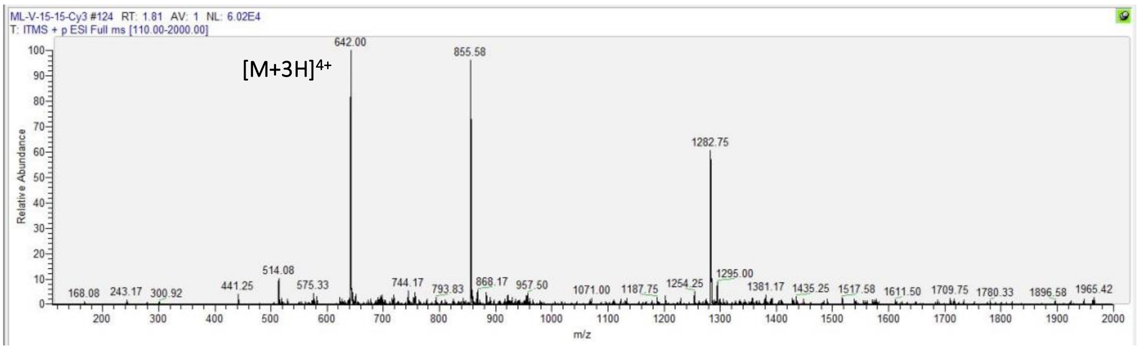

Chemical Formula: $C_{120}H_{151}N_{42}O_{24}^{+}$

MALDI-TOF: *m/z* calc: 2565.19 $[M]^{+}$ *m/z* found: 2565.12

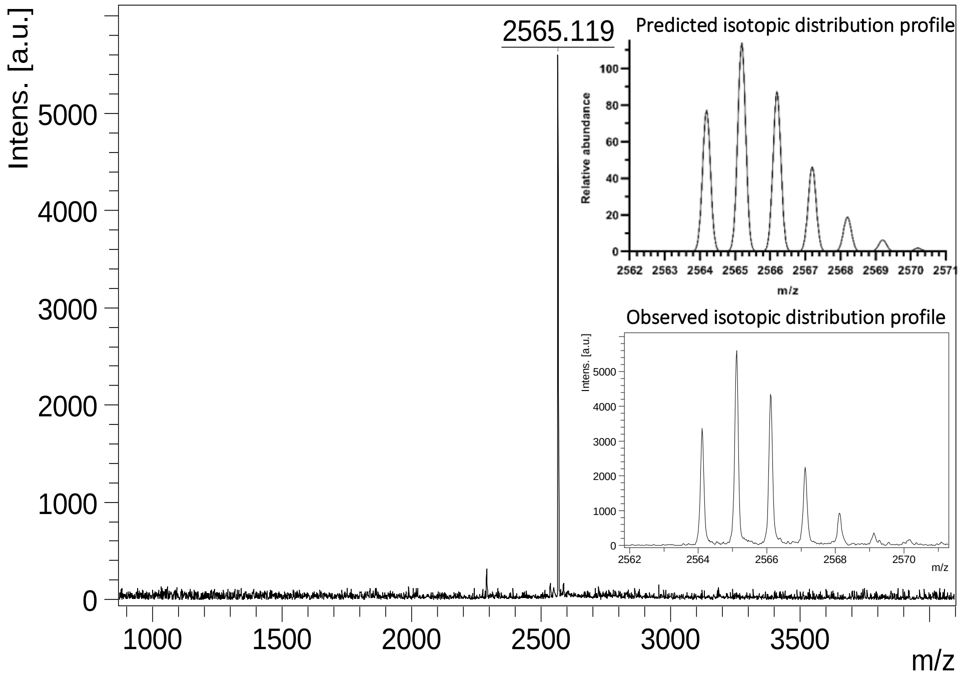

pc-PNA-SR-Bt: C-Ter: Lys-GGTGTTGACGAA-Lys(**Bt**)-Ac (Orange=N-7-MeG)

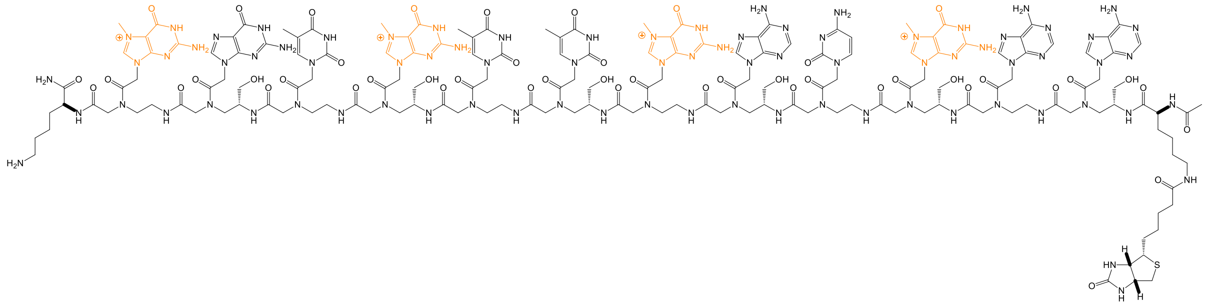

Chemical Formula: $C_{165}H_{226}N_{80}O_{47}S^{4+}$ **Exact Mass:** 4111.75 **Molecular Weight:** 4114.19

LC-MS (ESI): RT=1.26min. Absorbance at λ=260 nm. *m/z* expected for $[M]^{4+}$: 1028.44, *m/z* observed: 1028.75.

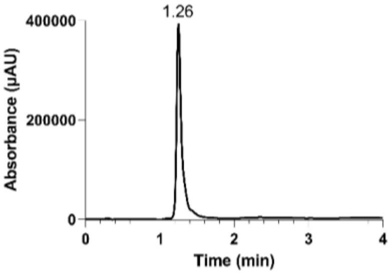

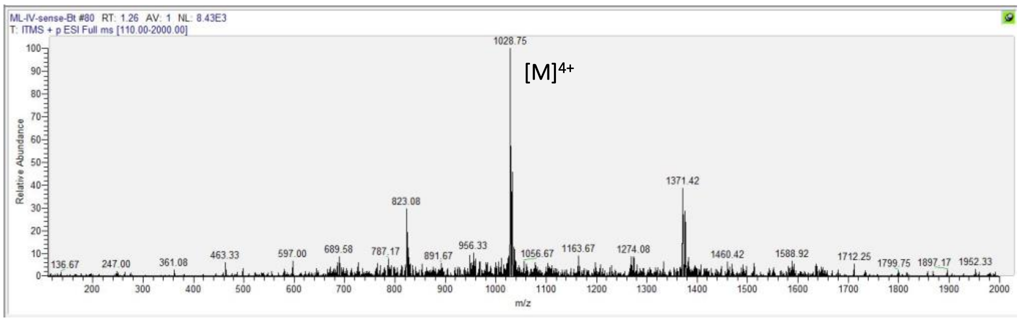

Chemical Formula: $C_{165}H_{226}N_{80}O_{47}S^{4+}$

MALDI-TOF: *m/z* cal: 4110.73 $[M\text{-}3H]^{+}$ *m/z* found: 4111.40

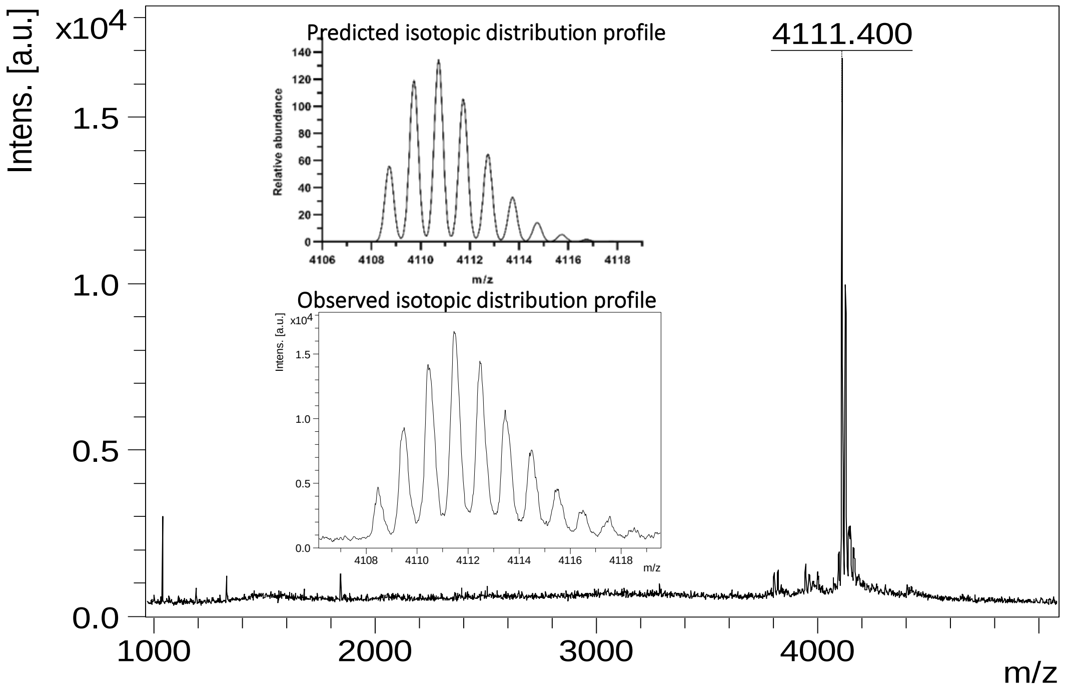

pc-PNA-SR: C-Ter: Lys-GGTGTTGACGAA-Lys-Ac (Orange=N-7-MeG)

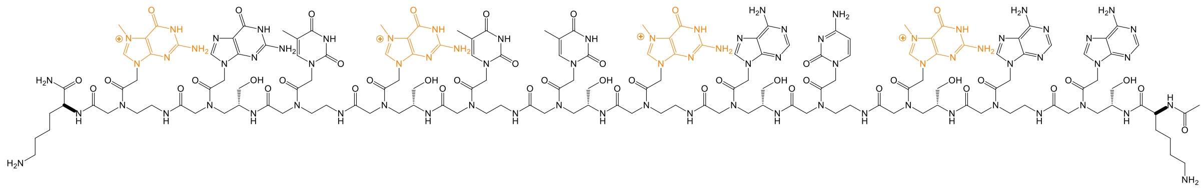

Chemical Formula: $C_{155}H_{212}N_{78}O_{45}^{4+}$ **Exact Mass:** 3885.67 **Molecular Weight:** 3887.90

LC-MS (ESI): RT=1.09min. Absorbance at λ=260 nm. *m/z* expected for $[M\text{-}H]^{3+}$: 1295.56, *m/z* observed: 1295.42.

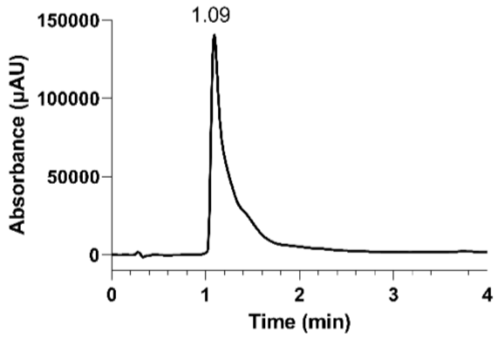

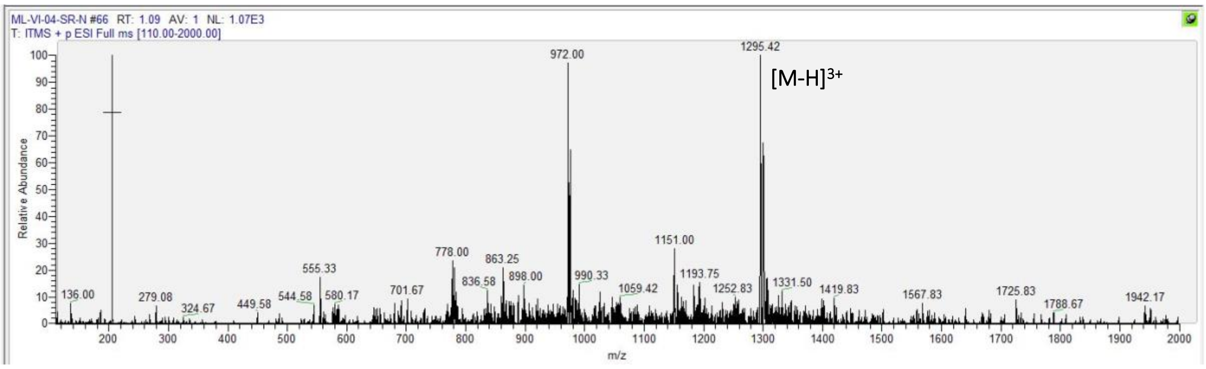

Chemical Formula: $C_{155}H_{212}N_{78}O_{45}{}^{4+}$

MALDI-TOF: *m/z* cal: 3884.65 [M-3H]$^{+}$ *m/z* found: 3884.81

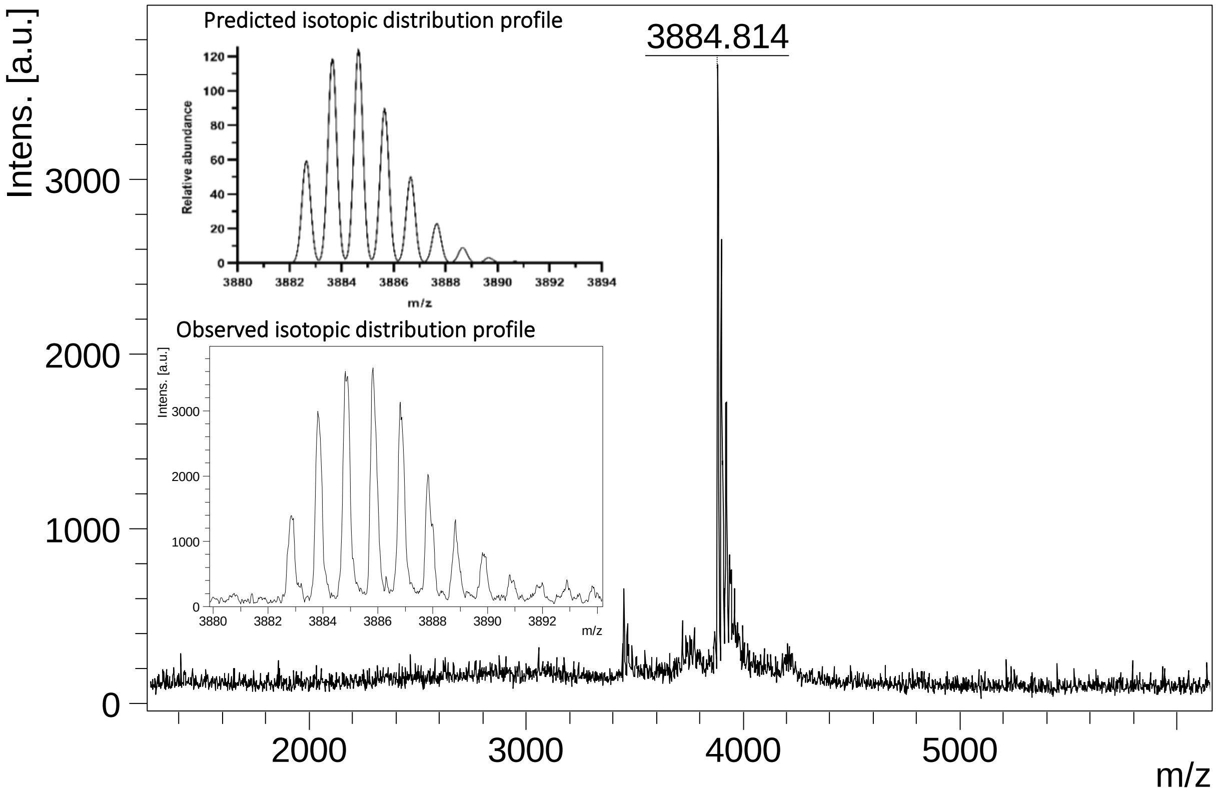

pc-PNA-AR-FITC: C'-Lys-TTCGTCAACACC-Lys(FITC)-Ac (Red=G-clamp)

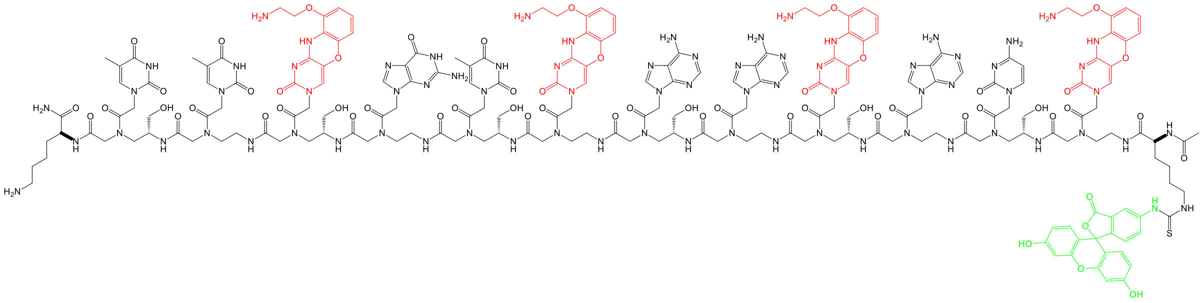

Chemical Formula: $C_{200}H_{239}N_{75}O_{58}S$ **Exact Mass:** 4650.78 **Molecular Weight:** 4653.64

LC-MS (ESI): RT=1.42min. Absorbance at λ=260 nm. *m/z* expected for $[M+5H]^{5+}$: 931.56, *m/z* observed: 931.58; *m/z* expected for $[M+4H]^{4+}$: 1164.20; *m/z* observed: 1164.25. *m/z* expected for $[M+3H]^{3+}$: 1551.94, *m/z* observed: 1551.67.

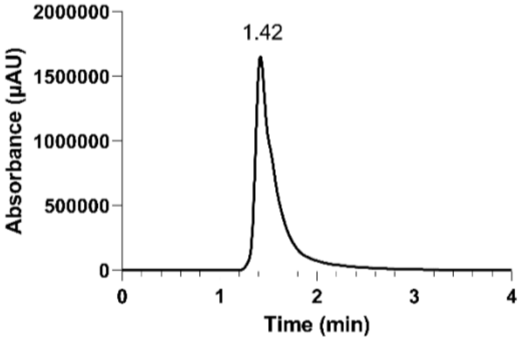

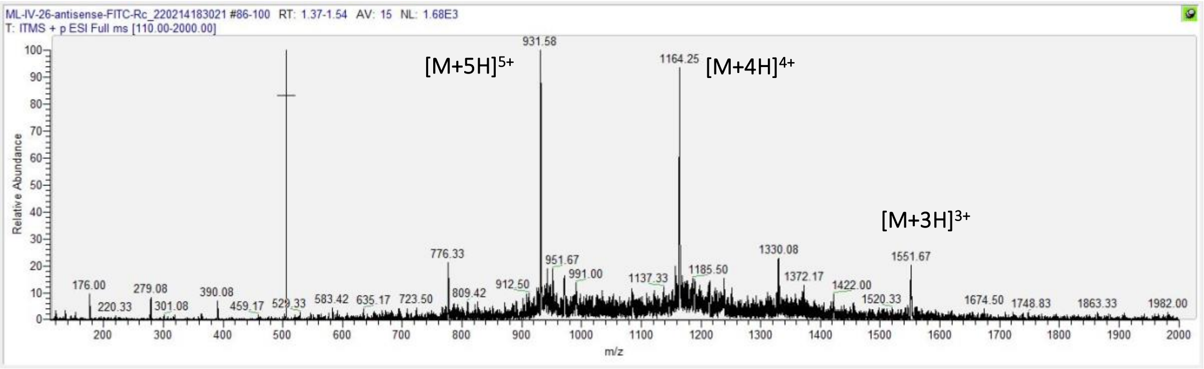

Chemical Formula: $C_{200}H_{239}N_{75}O_{58}S$

MALDI-TOF: *m/z* cal: 4652.79 $[M+H]^+$ *m/z* found: 4650.18

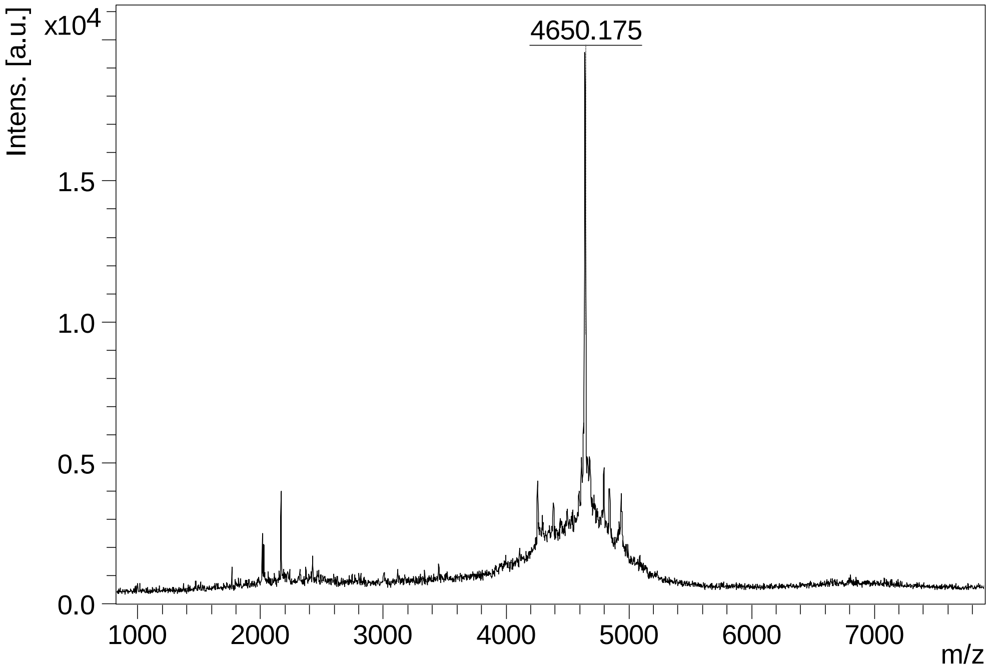

pc-PNA-SR-2: C-Ter: Lys-GTAGTCCTCTAC-Lys-Ac (Orange=N-7-MeG) (Red=G-clamp)

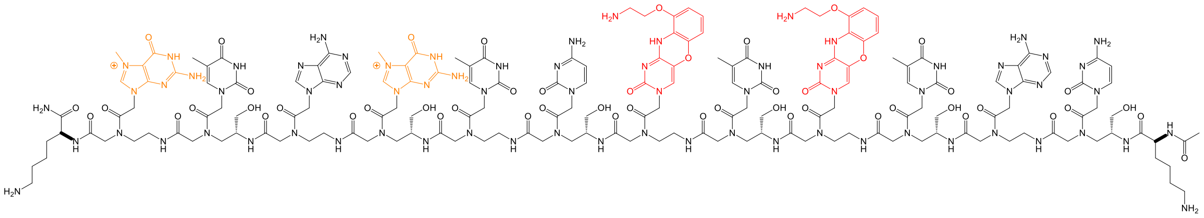

Chemical Formula: $C_{166}H_{221}N_{71}O_{51}^{2+}$ **Exact Mass:** 4024.69 **Molecular Weight:** 4027.04

LC-MS (ESI): RT=1.25min. Absorbance at λ=260 nm. *m/z* expected for $[M+2H]^{4+}$: 1007.18, *m/z* observed: 1006.92.

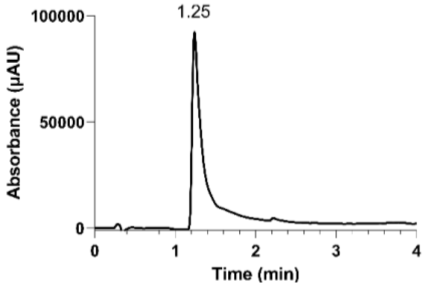

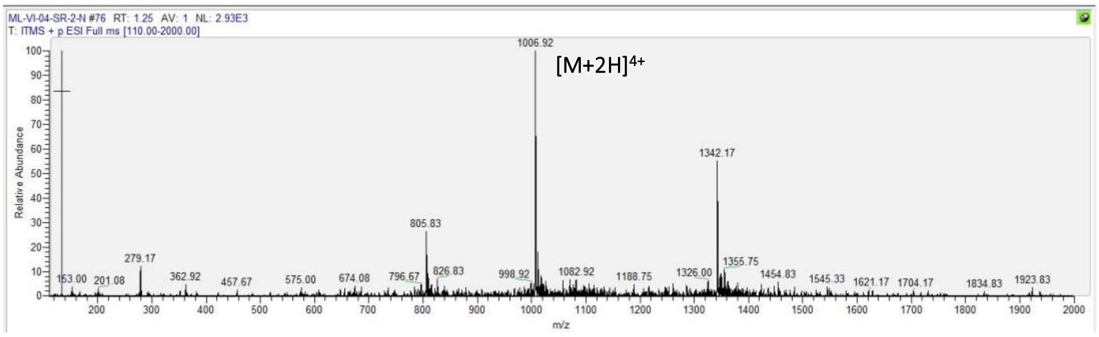

Chemical Formula: $C_{166}H_{221}N_{71}O_{51}^{2+}$

MALDI-TOF: *m/z* cal: 4025.69 $[M-H]^{+}$ *m/z* found: 4026.04

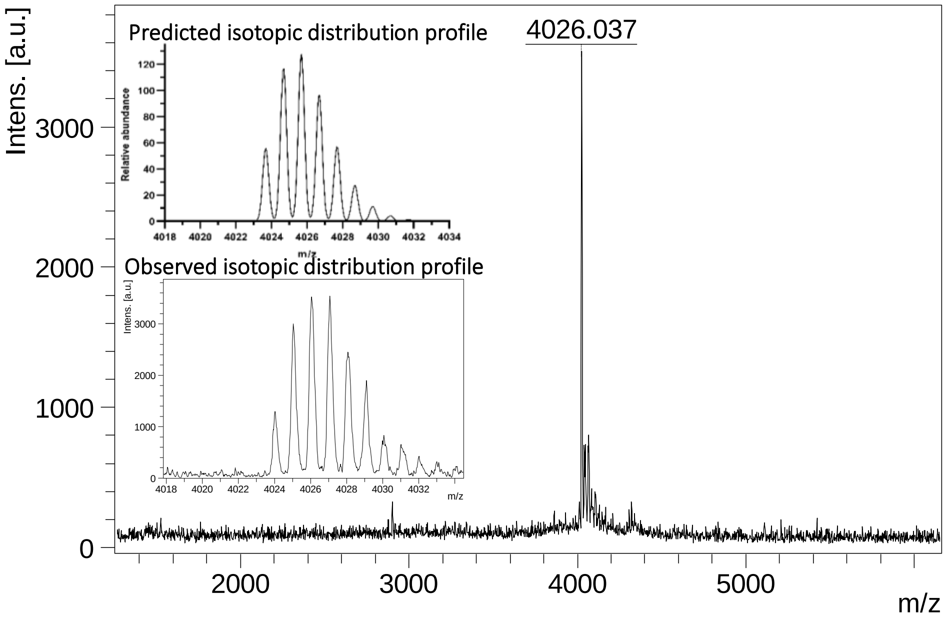

pc-PNA-AR-2-Bt: C-Ter: Lys-GTAGAGGACTAC-Lys(**Bt**)-Ac (Orange=N-7-MeG) (Red=G-clamp)

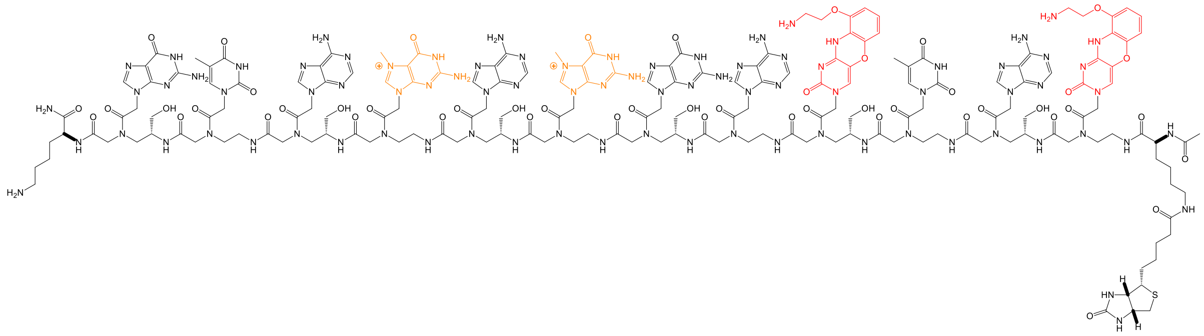

Chemical Formula: $C_{178}H_{233}N_{83}O_{49}S^{2+}$ **Exact Mass:** 4348.80 **Molecular Weight:** 4351.41

LC-MS (ESI): RT=1.39min. Absorbance at λ=260 nm. *m/z* expected for $[M+2H]^{4+}$: 1088.21, *m/z* observed: 1088.17.

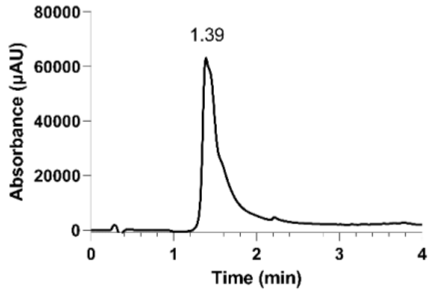

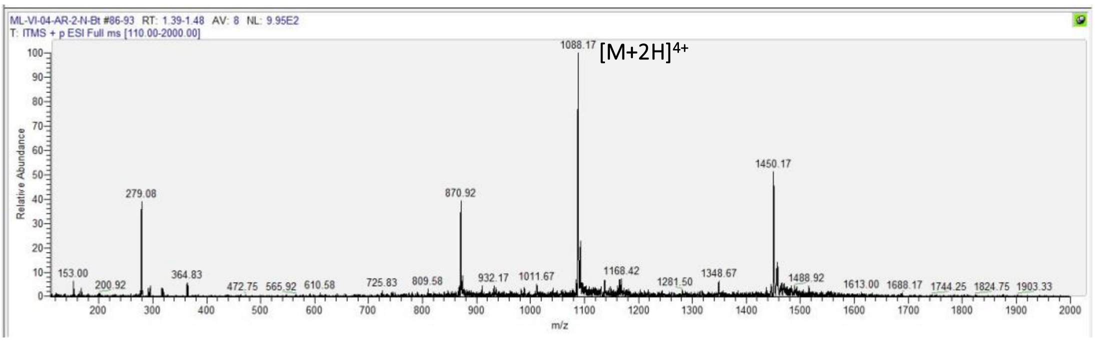

Chemical Formula: $C_{178}H_{233}N_{83}O_{49}S^{2+}$

MALDI-TOF: *m/z* cal: 4349.81 $[M-H]^{+}$ *m/z* found: 4350.38

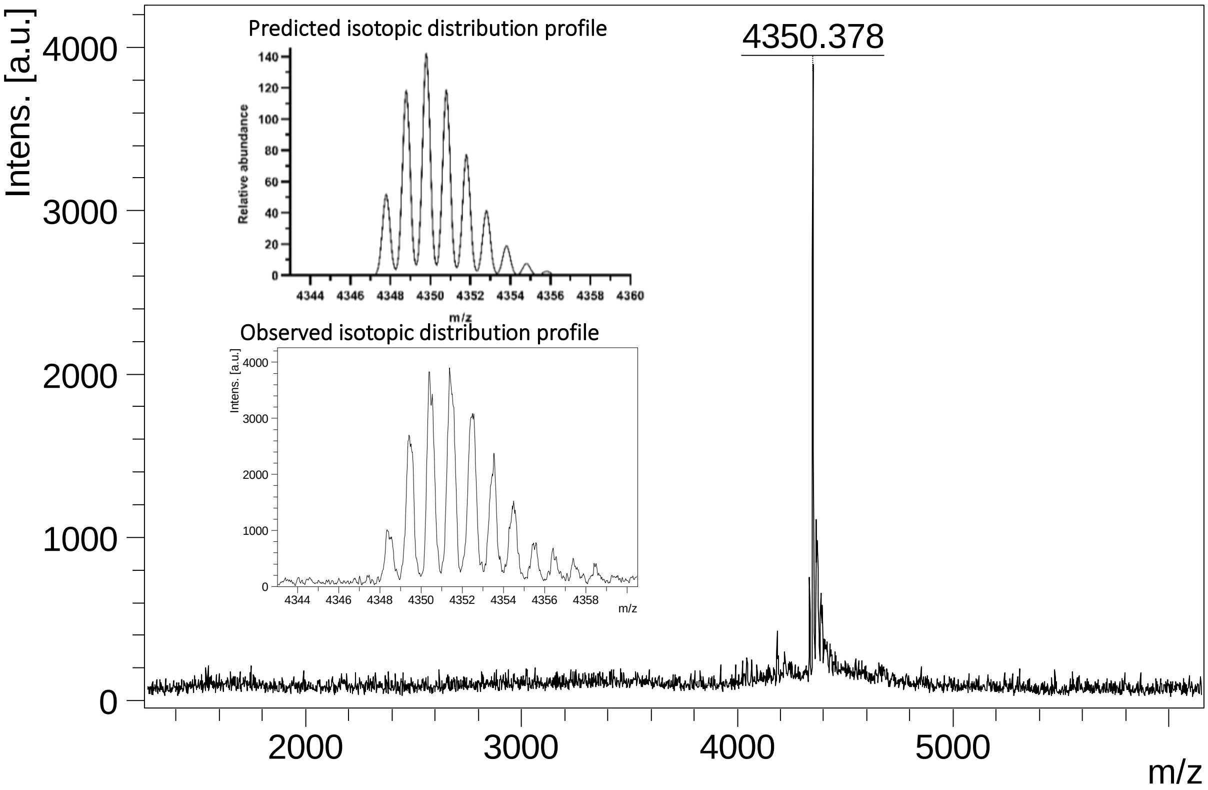

PNA-SR-Bt: C-Ter: Lys-GGTGTTGACGAA-Lys(**Bt**)-Ac

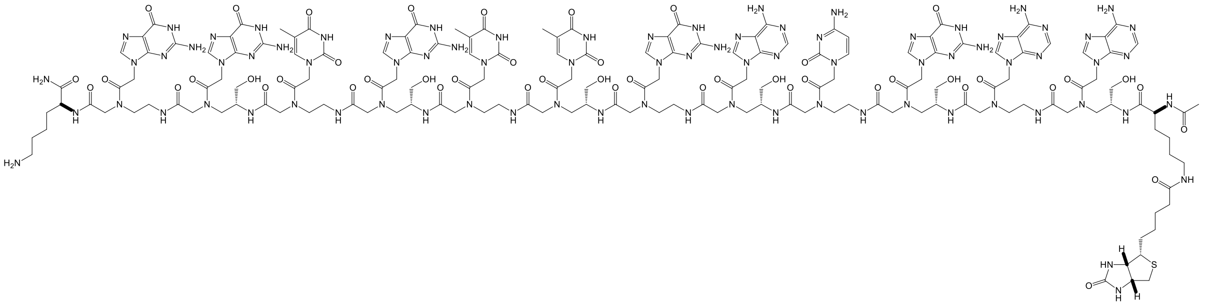

Chemical Formula: $C_{161}H_{214}N_{80}O_{47}S$ **Exact Mass:** 4051.65 **Molecular Weight:** 4054.06

LC-MS (ESI): RT=1.23min. Absorbance at λ=260 nm. *m/z* expected for $[M+3H]^{3+}$: 1352.34, *m/z* observed: 1352.00.

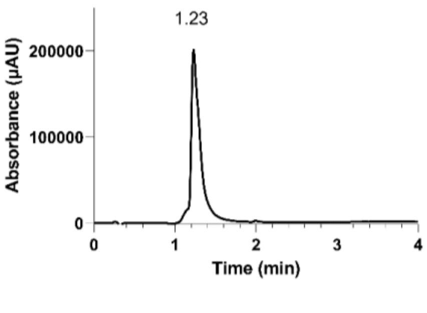

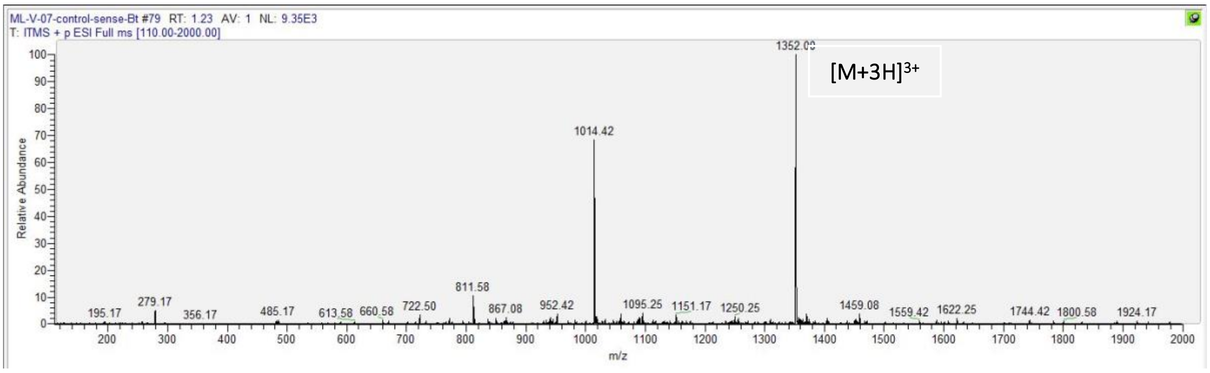

Chemical Formula: $C_{161}H_{214}N_{80}O_{47}S$

MALDI-TOF: *m/z* cal: 4054.66 [M+H]$^+$ *m/z* found: 4054.76

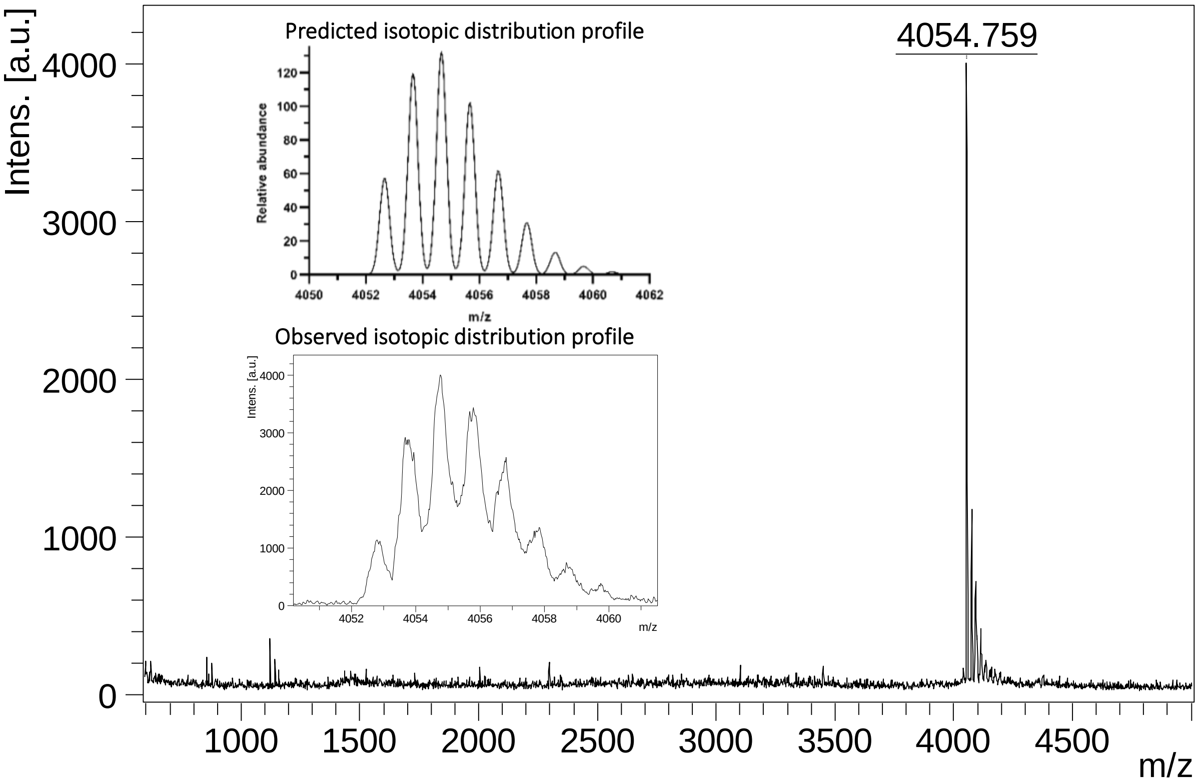

PNA-SR: C-Ter: Lys-GGTGTTGACGAA-Lys-Ac

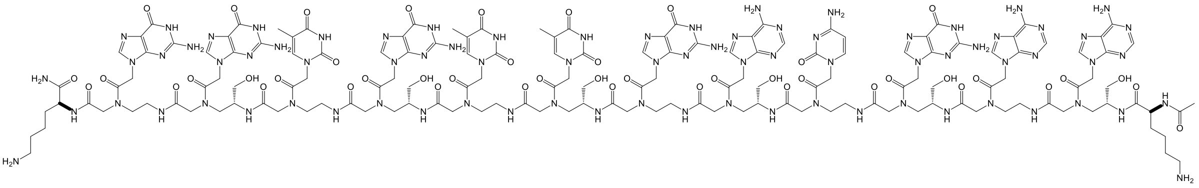

Chemical Formula: $C_{151}H_{200}N_{78}O_{45}$ **Exact Mass:** 3825.58 **Molecular Weight:** 3827.76

LC-MS (ESI): RT=1.15min. Absorbance at λ=260 nm. *m/z* expected for $[M+3H]^{3+}$: 1276.53, *m/z* observed: 1276.50.

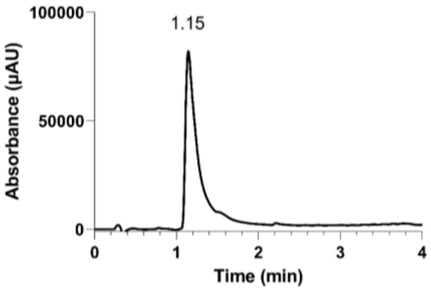

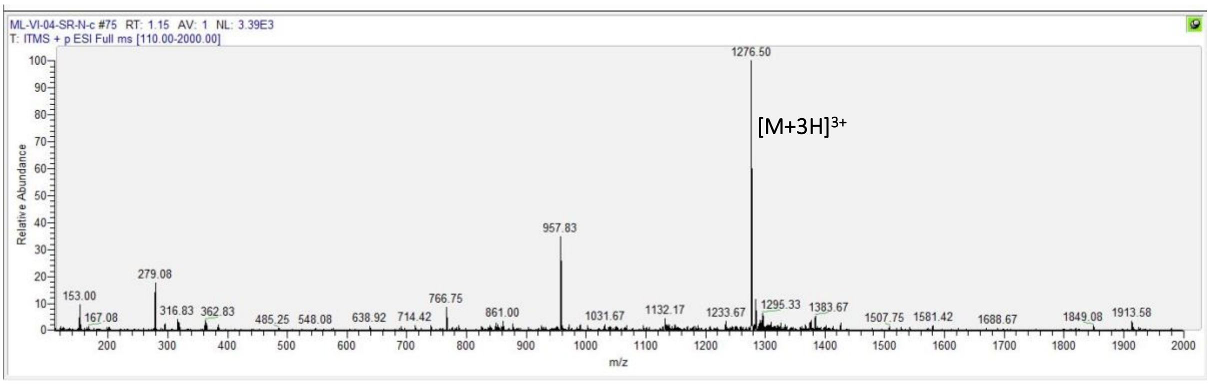

Chemical Formula: $C_{151}H_{200}N_{78}O_{45}$

MALDI-TOF: *m/z* cal: 3828.57 $[M+H]^+$ *m/z* found: 3829.27

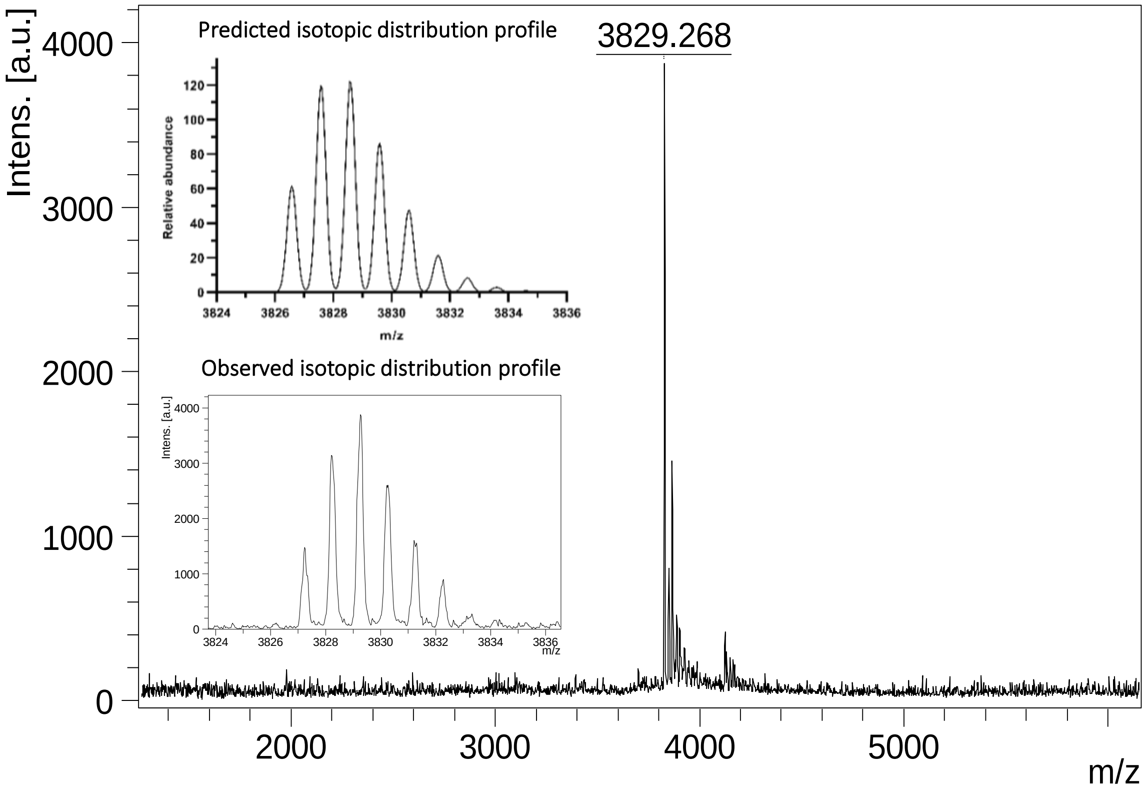

PNA-AR-FITC: C-Ter: Lys-TTCGTCAACACC-Lys(**FITC**)-Ac

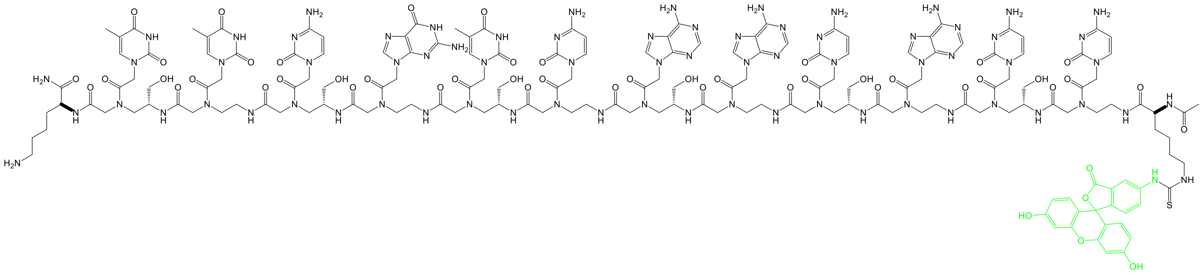

Chemical Formula: $C_{168}H_{211}N_{71}O_{50}S$ **Exact Mass:** 4054.59 **Molecular Weight:** 4057.04

LC-MS (ESI): RT=1.39min. Absorbance at λ=260 nm. *m/z* expected for $[M+5H]^{5+}$: 812.33, *m/z* observed: 812.25; *m/z* expected for $[M+4H]^{4+}$: 1015.15, *m/z* observed: 1015.08; *m/z* expected for $[M+3H]^{3+}$: 1353.20, *m/z* observed: 1352.83.

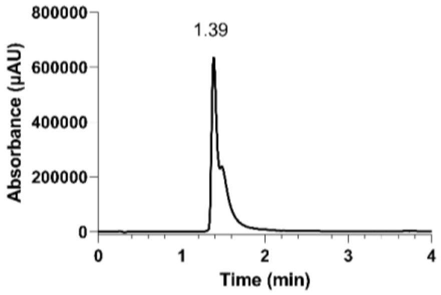

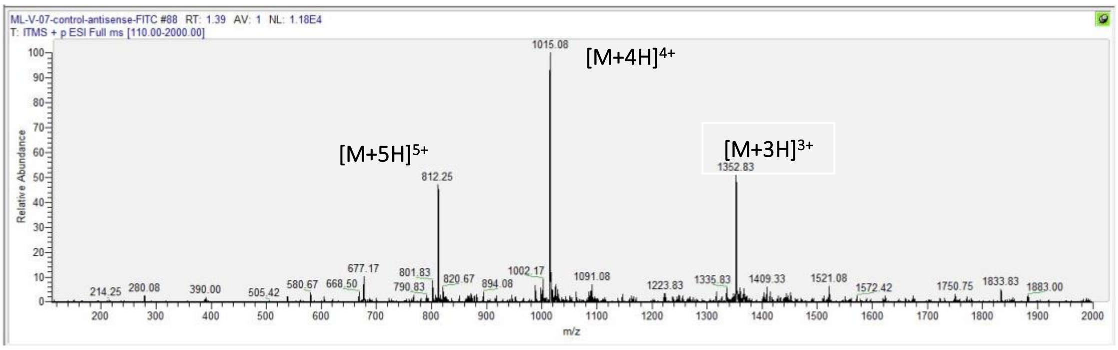

Chemical Formula: $C_{168}H_{211}N_{71}O_{50}S$

MALDI-TOF: *m/z* cal: 4056.60 $[M+H]^+$ *m/z* found: 4055.82

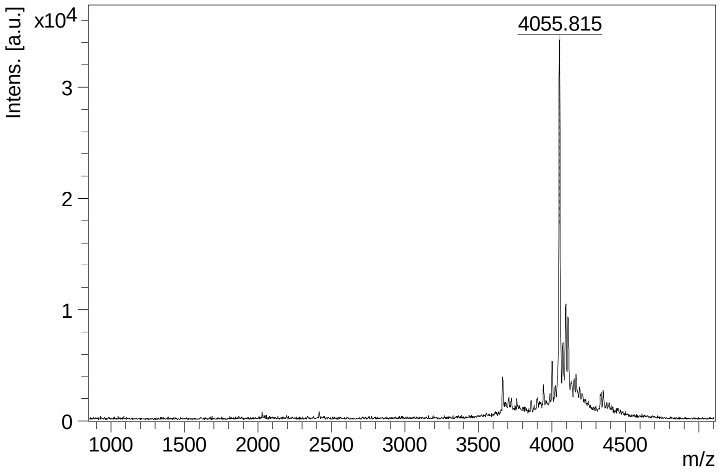

PNA-SR-2: C-Ter: Lys-GTAGTCCTCTAC-Lys-Ac

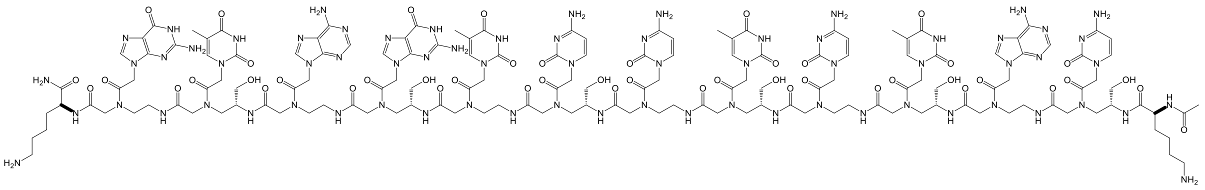

Chemical Formula: $C_{148}H_{201}N_{69}O_{47}$ **Exact Mass:** 3696.55 **Molecular Weight:** 3698.67

LC-MS (ESI): RT=1.10min. Absorbance at λ=260 nm. *m/z* expected for $[M+4H]^{4+}$: 925.39, *m/z* observed: 925.50.

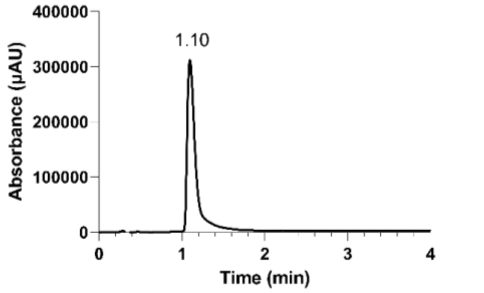

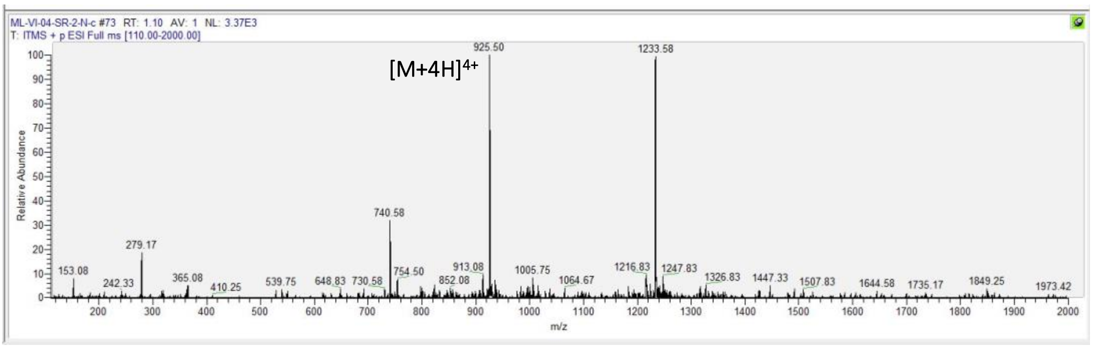

Chemical Formula: $C_{148}H_{201}N_{69}O_{47}$

MALDI-TOF: m/z cal: 3698.55 $[M+H]^+$ m/z found: 3697.86

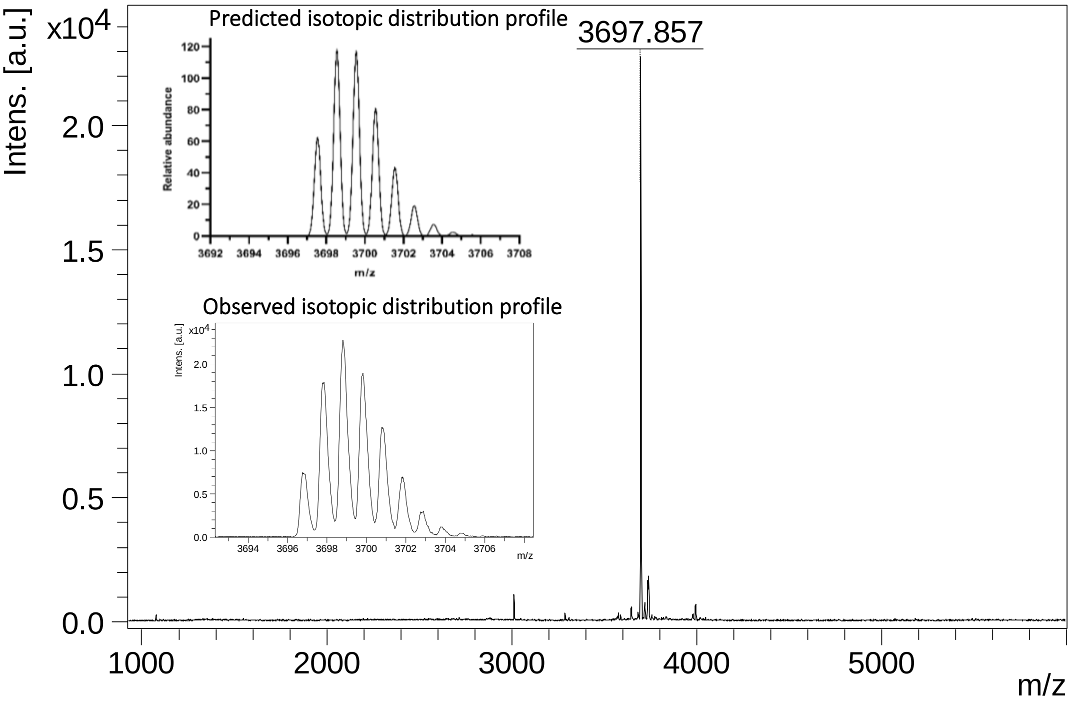

PNA-AR-2-Bt: C-Ter: Lys-<u>G</u>T<u>A</u>G<u>A</u>G<u>G</u>A<u>C</u>T<u>A</u>C-Lys(**Bt**)-Ac

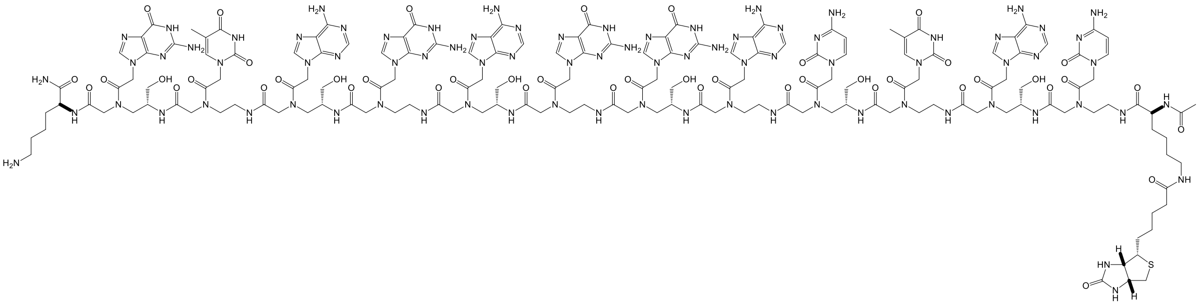

Chemical Formula: $C_{160}H_{213}N_{81}O_{45}S$ **Exact Mass:** 4020.66 **Molecular Weight:** 4023.05

LC-MS (ESI): RT=1.25min. Absorbance at λ=260 nm. *m/z* expected for $[M+4H]^{4+}$: 1006.67, *m/z* observed: 1006.75.

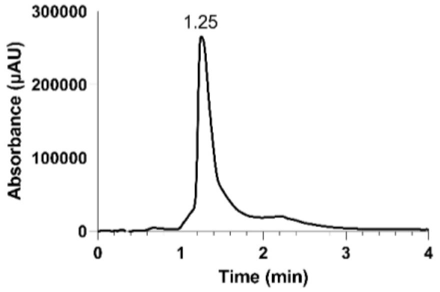

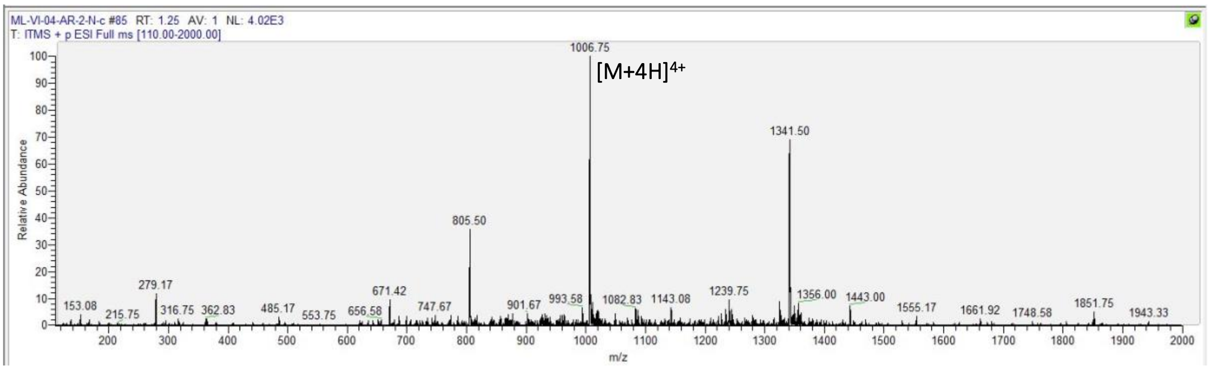

Chemical Formula: $C_{160}H_{213}N_{81}O_{45}S$

MALDI-TOF: *m/z* cal: 4023.65 $[M+H]^+$ *m/z* found: 4023.10

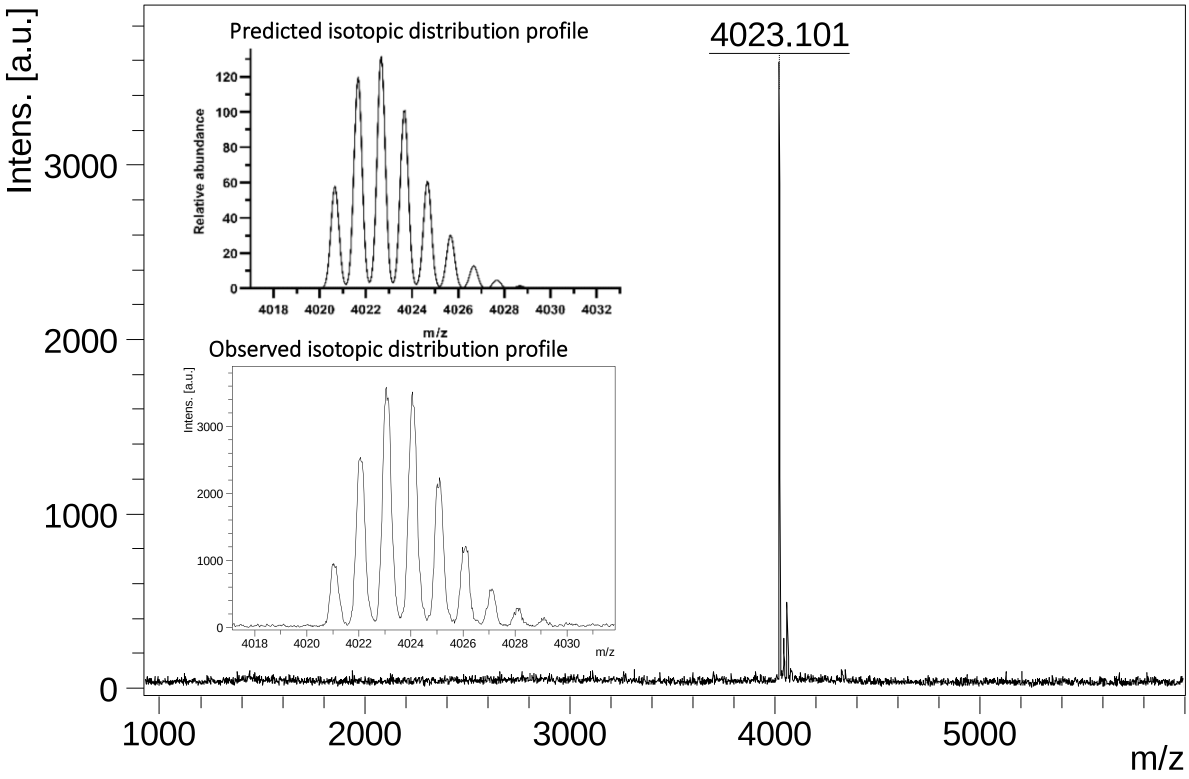

pc-PNA-oS: C-Ter: Lys-CTGGGTGAATAC-Lys-Ac (Orange=N-7-MeG) (Red=G-clamp)

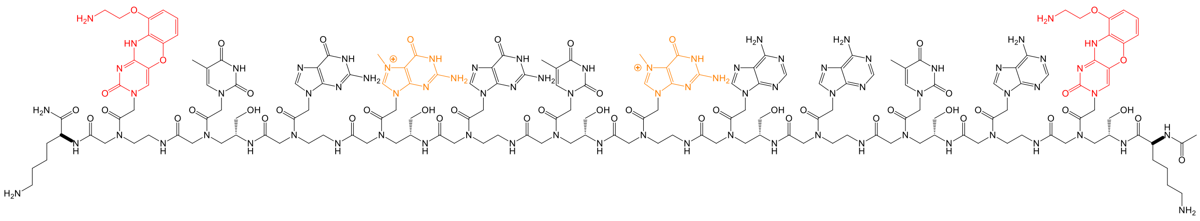

Chemical Formula: $C_{168}H_{220}N_{78}O_{49}^{2+}$ **Exact Mass:** 4113.71 **Molecular Weight:** 4116.10

LC-MS (ESI): RT=1.23min. Absorbance at λ=260 nm. *m/z* expected for $[M+2H]^{4+}$: 1029.44, *m/z* observed: 1029.42.

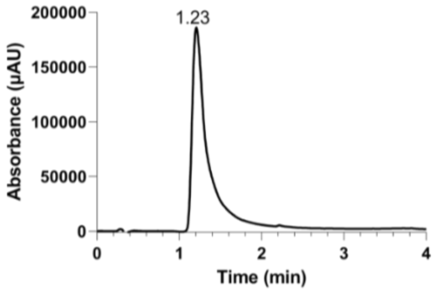

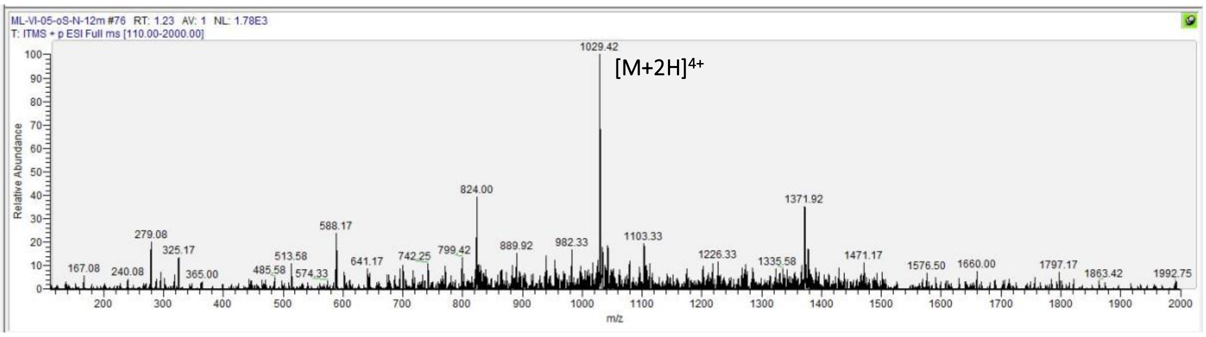

Chemical Formula: $C_{168}H_{220}N_{78}O_{49}^{2+}$

MALDI-TOF: *m/z* cal: 4114.71 $[M-H]^+$ *m/z* found: 4113.71

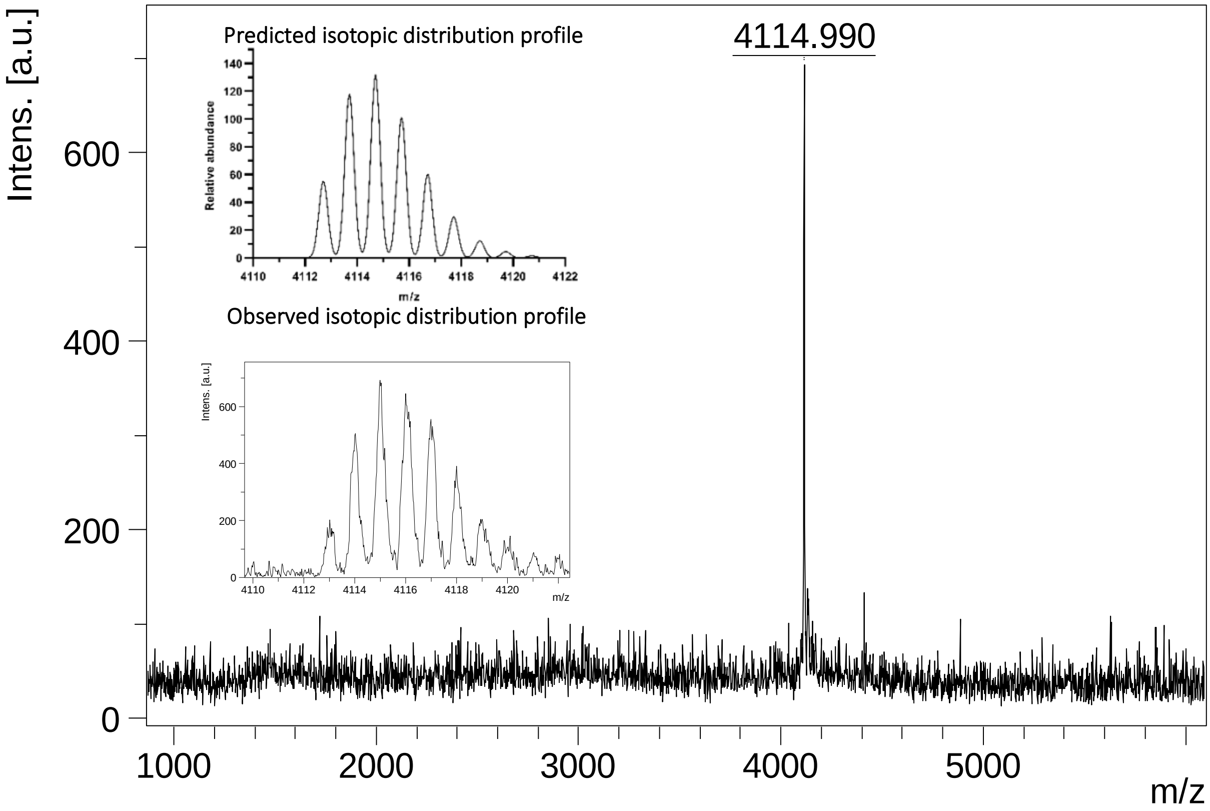

pc-PNA-oA-Bt: C-Ter: Lys-GTATTCACCCAG-Lys(**Bt**)-Ac (Orange=N-7-MeG) (Red=G-clamp)

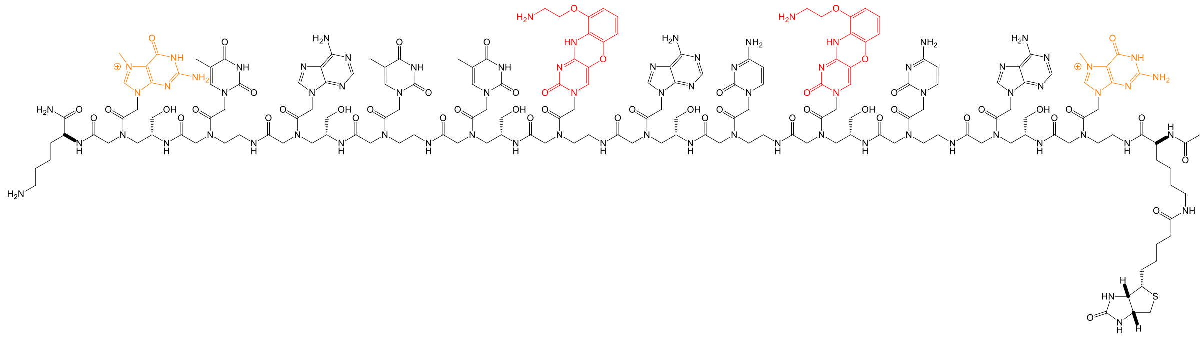

Chemical Formula: $C_{176}H_{234}N_{76}O_{51}S^{2+}$ **Exact Mass:** 4259.78 **Molecular Weight:** 4262.35

LC-MS (ESI): RT=1.32min. Absorbance at λ=260 nm. *m/z* expected for $[M+2H]^{4+}$: 1065.94, *m/z* observed: 1065.75.

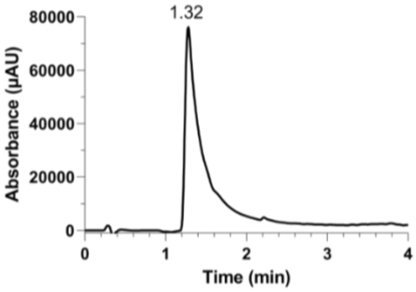

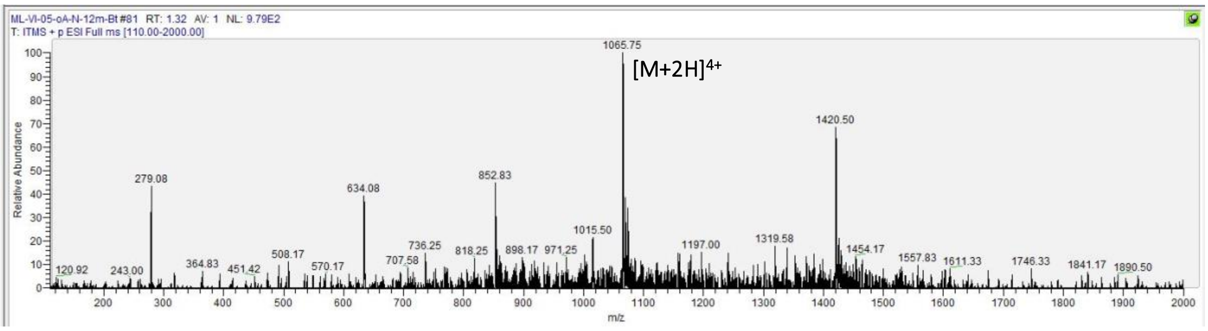

Chemical Formula: $C_{176}H_{234}N_{76}O_{51}S^{2+}$

MALDI-TOF: m/z cal: 4260.71 [M-H]$^+$ m/z found: 4529.81

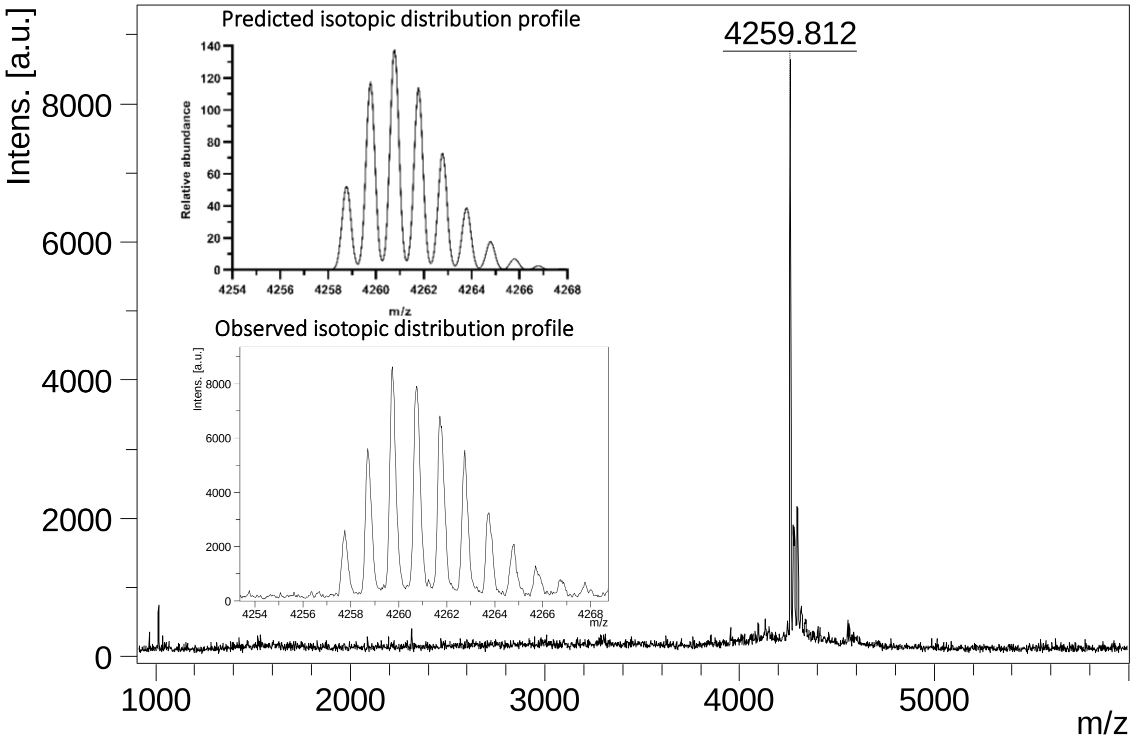

pc-PNA-oS-2: C-Ter: Lys-CACAACCAGTGG-Lys-Ac (Orange=N-7-MeG) (Red=G-clamp)

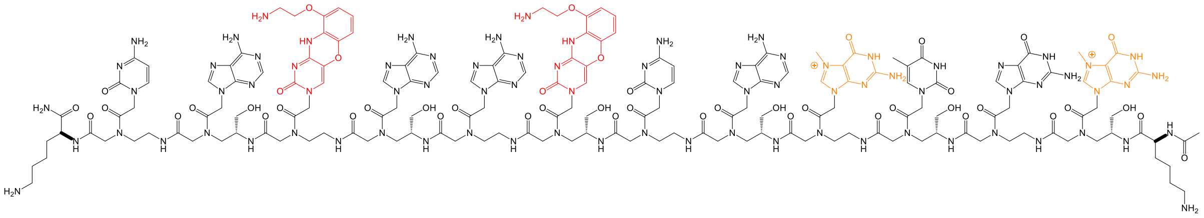

Chemical Formula: $C_{166}H_{218}N_{80}O_{46}^{2+}$ **Exact Mass:** 4067.72 **Molecular Weight:** 4070.08

LC-MS (ESI): RT=1.15min. Absorbance at λ=260 nm. *m/z* expected for [M+2H]$^{4+}$: 1017.93, *m/z* observed: 1017.83.

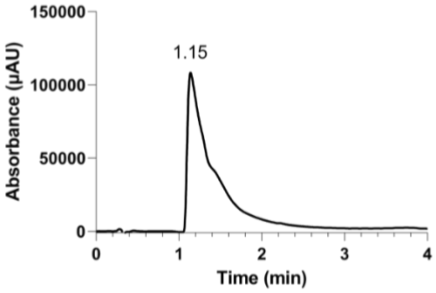

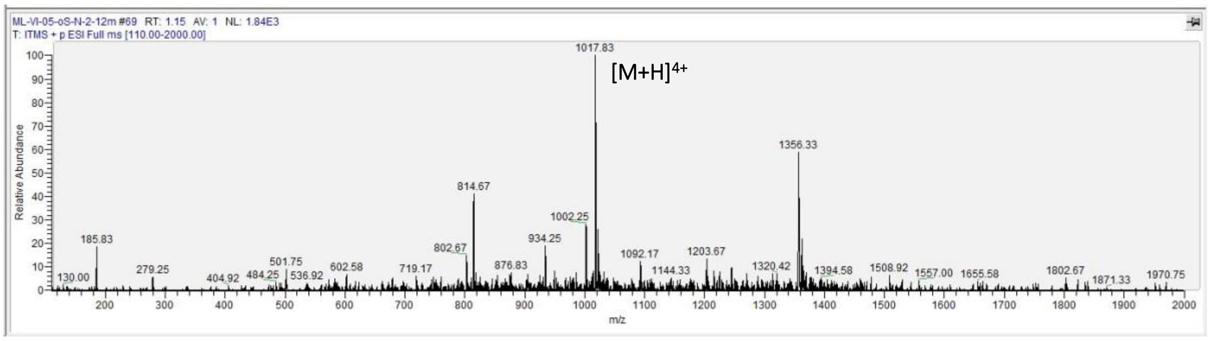

Chemical Formula: $C_{166}H_{218}N_{80}O_{46}^{2+}$

MALDI-TOF: *m/z* cal: 4068.72 $[M-H]^+$ *m/z* found: 4069.12

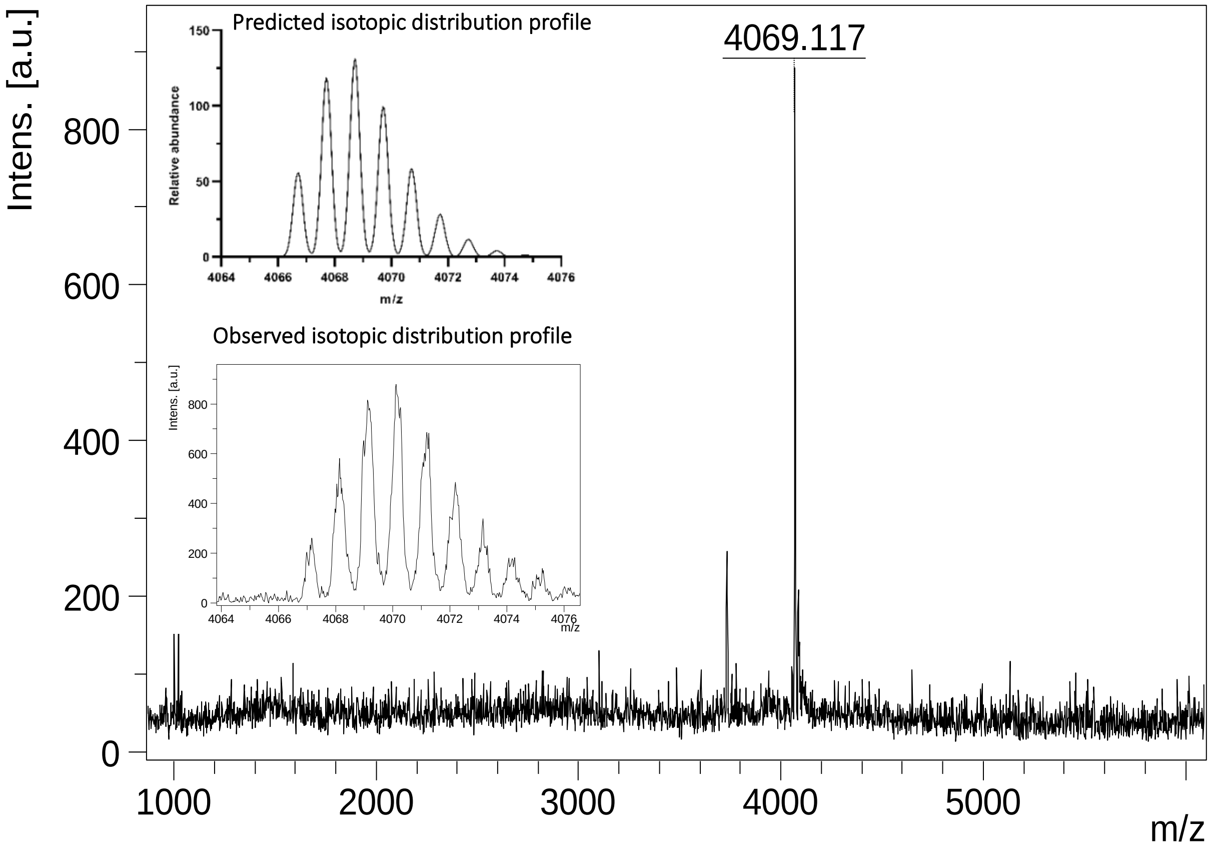

pc-PNA-oA-2-FITC: C-Ter: Lys-CCACTGGTTGTG-Lys(**FITC**)-Ac (Orange=N-7-MeG) (Red=G-clamp)

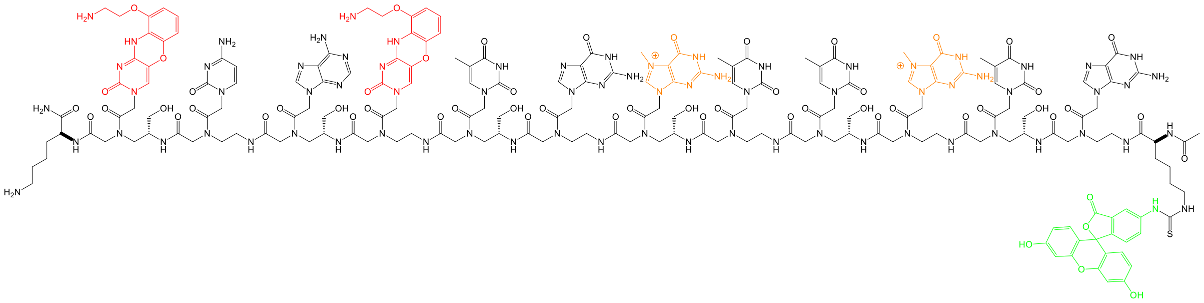

Chemical Formula: $C_{188}H_{232}N_{74}O_{57}S^{2+}$ **Exact Mass:** 4469.72 **Molecular Weight:** 4472.44

LC-MS (ESI): RT=1.50min. Absorbance at λ=260 nm. *m/z* expected for $[M+2H]^{4+}$: 1118.43, *m/z* observed: 1118.42.

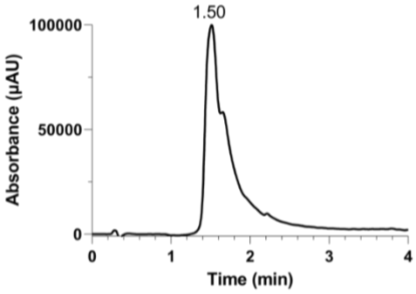

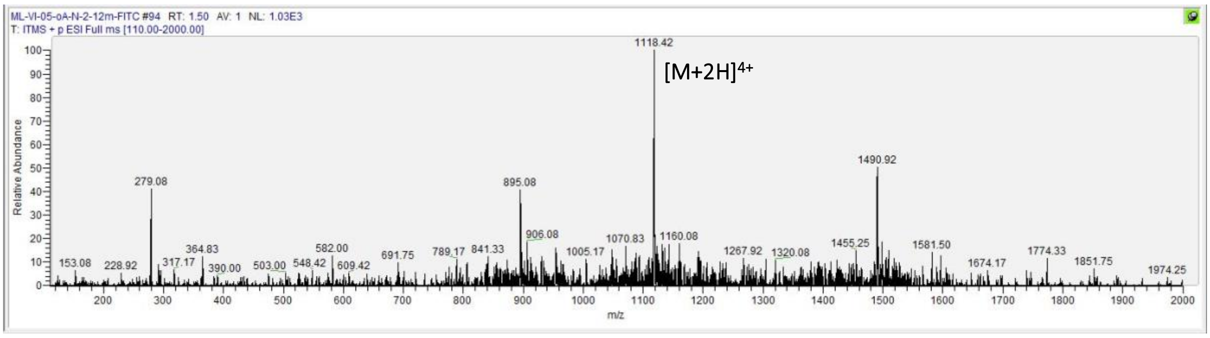

Chemical Formula: $C_{188}H_{232}N_{74}O_{57}S^{2+}$

MALDI-TOF: *m/z* cal: 4470.72 $[M-H]^{+}$ *m/z* found: 4471.22

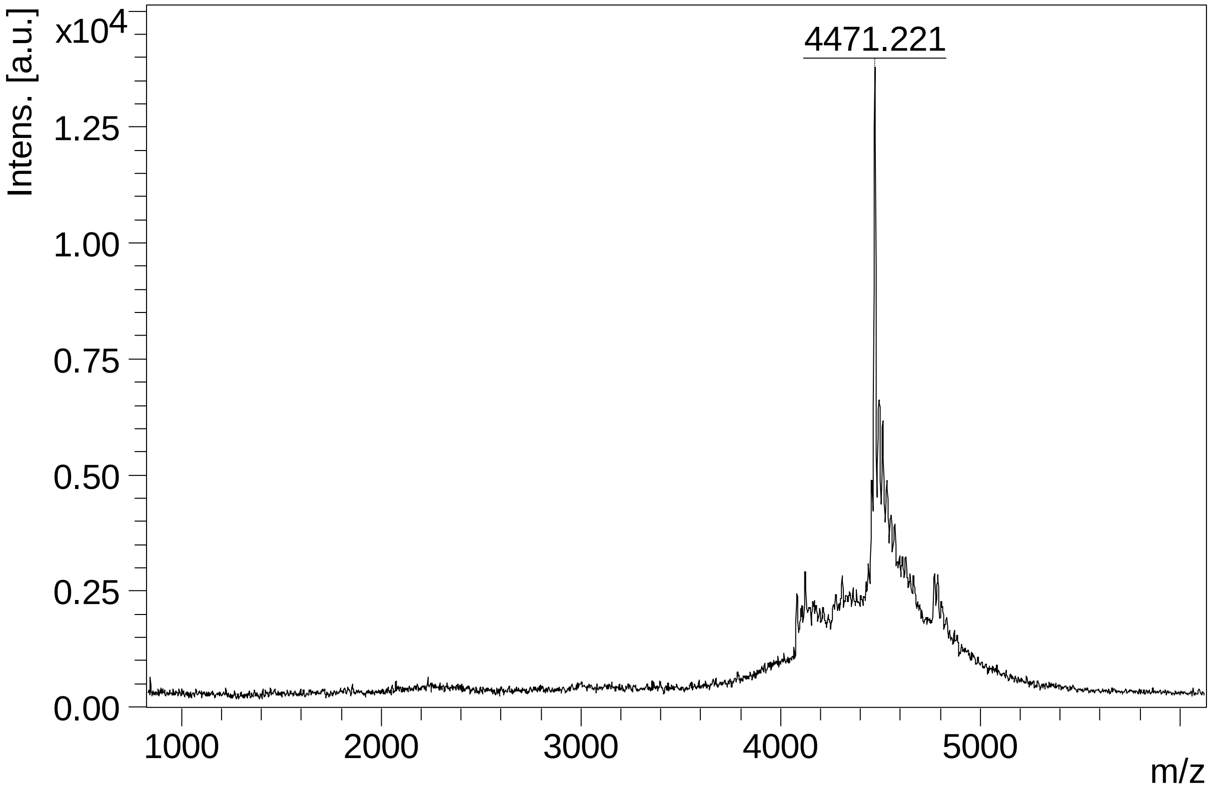

pc-PNA-dS: C-Ter: Lys-TTGGGTGATTAC-Lys-Ac (Orange=N-7-MeG) (Red=G-clamp)

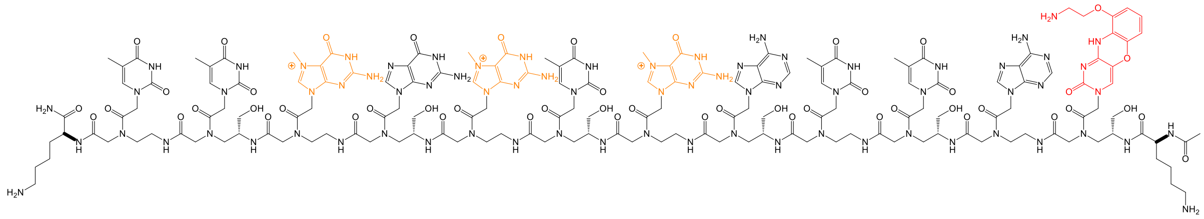

Chemical Formula: $C_{162}H_{218}N_{73}O_{50}^{3+}$ **Exact Mass:** 3985.67 **Molecular Weight:** 3987.99

LC-MS (ESI): RT=2.22min. Absorbance at λ=260 nm. *m/z* expected for $[M]^{3+}$: 1329.22, *m/z* observed: 1329.00.

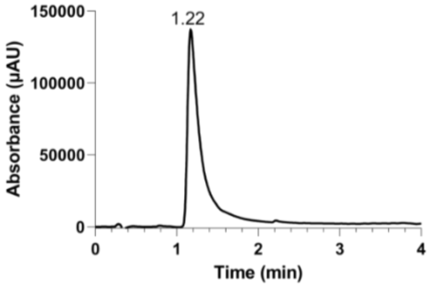

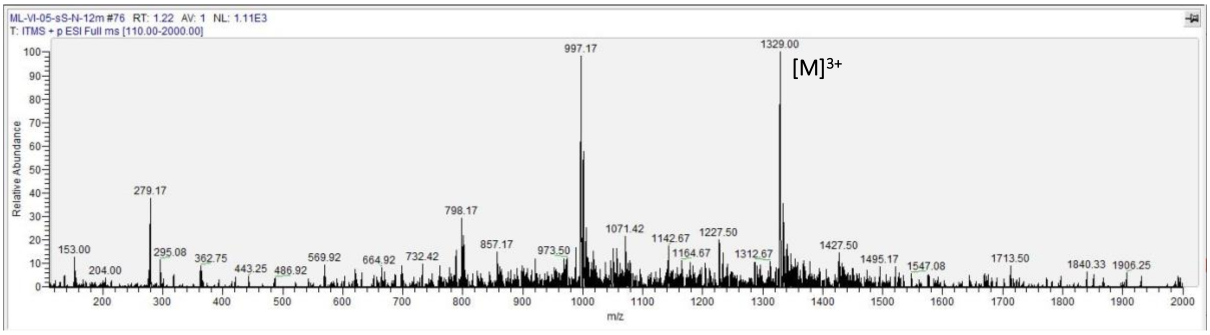

Chemical Formula: $C_{162}H_{218}N_{73}O_{50}{}^{3+}$

MALDI-TOF: *m/z* cal: 3985.61 $[M-2H]^+$ *m/z* found: 3984.78

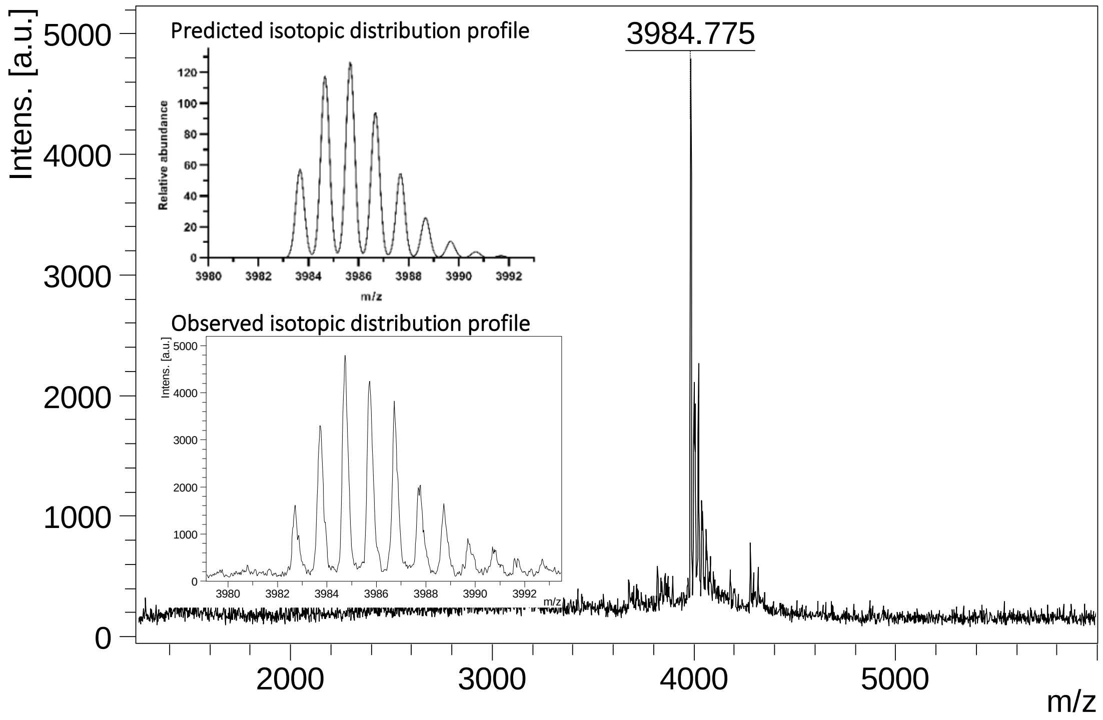

pc-PNA-dA-Bt: C-Ter: Lys-GTAATCACCCAA-Lys(**Bt**)-Ac (Orange=N-7-MeG) (Red=G-clamp)

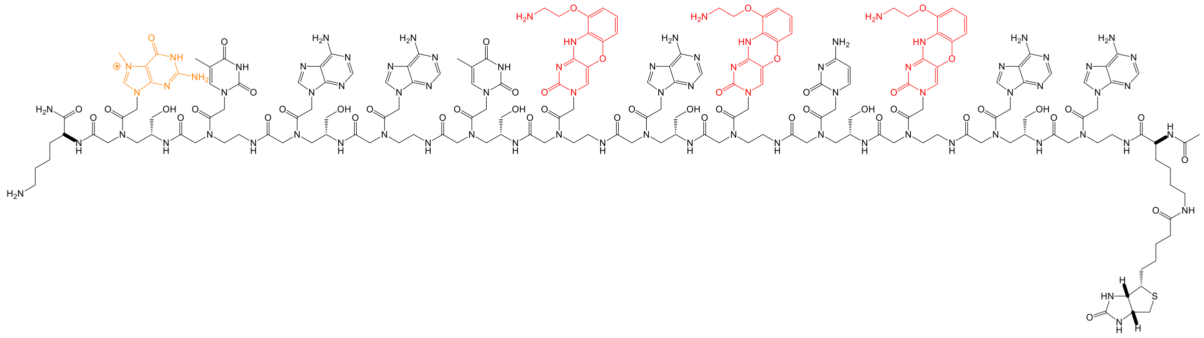

Chemical Formula: $C_{183}H_{237}N_{80}O_{50}S^+$ **Exact Mass:** 4386.82 **Molecular Weight:** 4389.48

LC-MS (ESI): RT=1.27min. Absorbance at λ=260 nm. *m/z* expected for $[M+3H]^{4+}$: 1097.95, *m/z* observed: 1097.83.

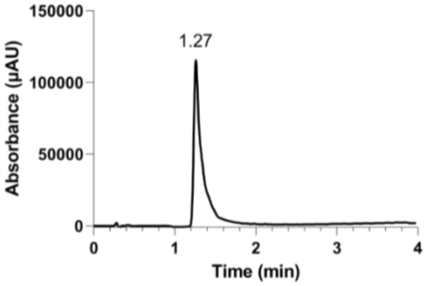

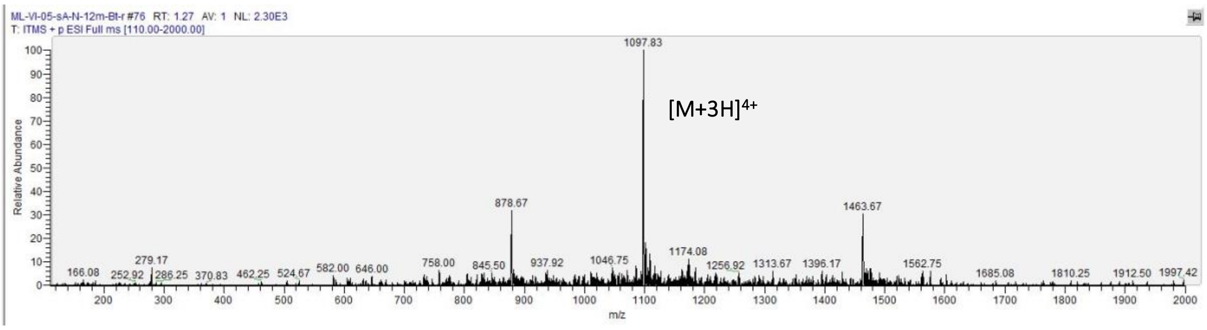

Chemical Formula: $C_{183}H_{237}N_{80}O_{50}S^{+}$

MALDI-TOF: *m/z* cal: 4388.76 $[M]^{+}$ *m/z* found: 4388.14

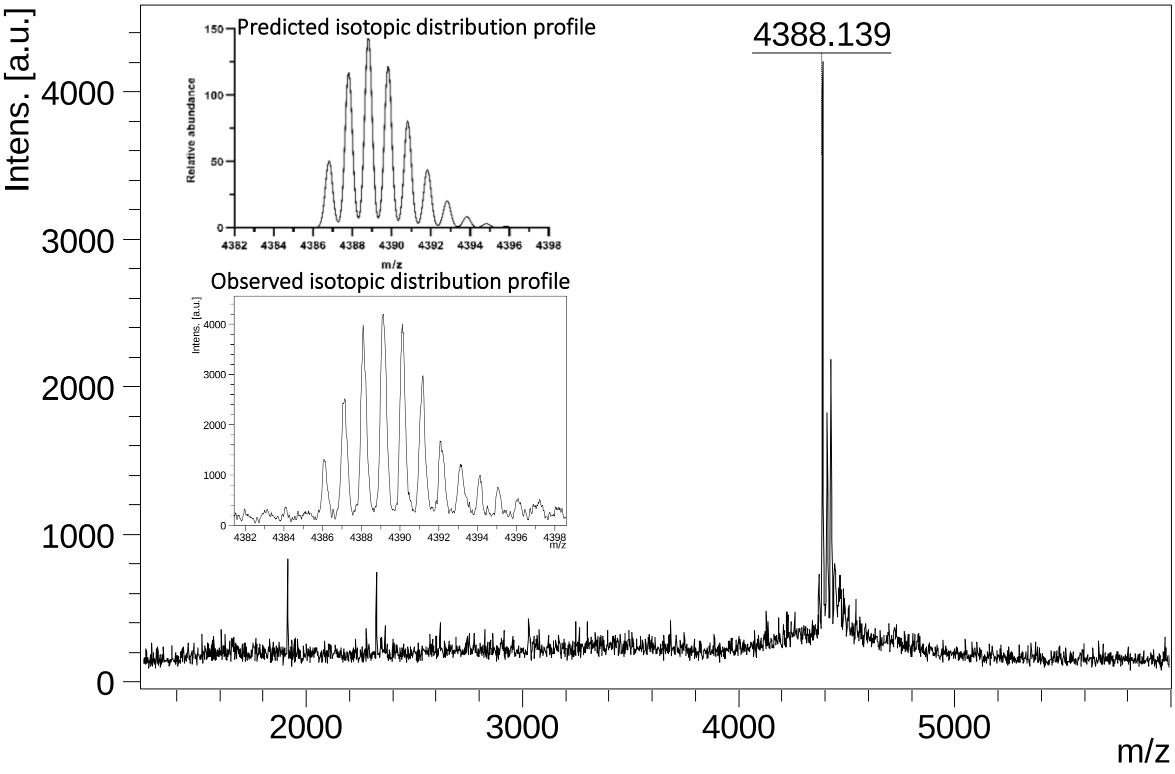

pc-PNA-dS-2: C-Ter: Lys-CACAACCAATGG-Lys-Ac (Orange=N-7-MeG) (Red=G-clamp)

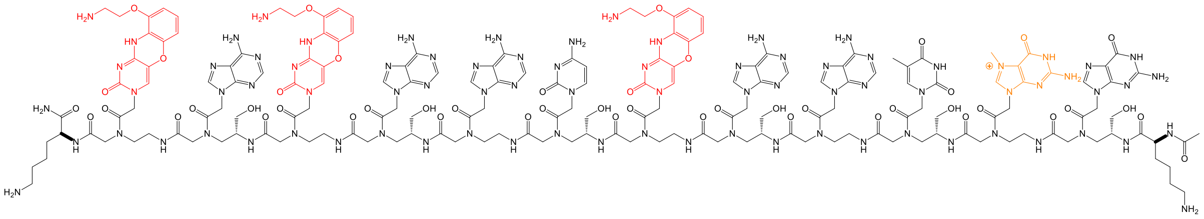

Chemical Formula: $C_{173}H_{222}N_{81}O_{47}^{+}$ **Exact Mass:** 4185.75 **Molecular Weight:** 4188.20

LC-MS (ESI): RT=1.23min. Absorbance at λ=260 nm. *m/z* expected for $[M+3H]^{4+}$: 1047.69, *m/z* observed: 1047.33.

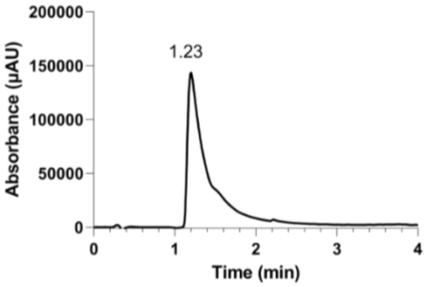

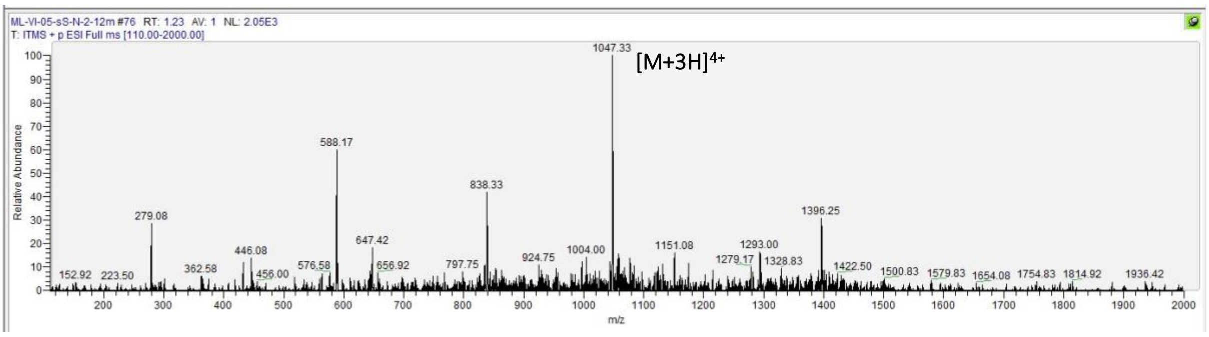

Chemical Formula: $C_{173}H_{222}N_{81}O_{47}^{+}$

MALDI-TOF: *m/z* cal: 4187.76 $[M]^{+}$ *m/z* found: 4187.65

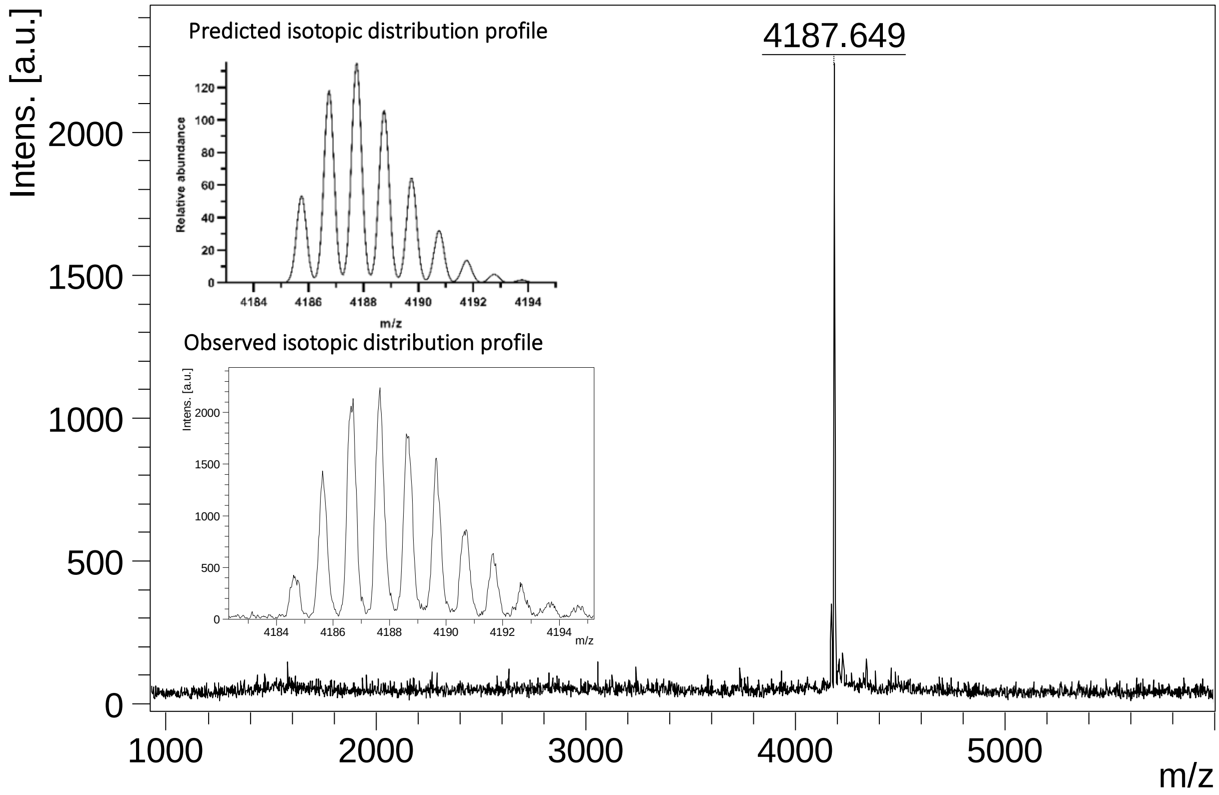

pc-PNA-dA-2-FITC: C-Ter: Lys-CCATTGGTTGTG-Lys(FITC)-Ac (Orange=N-7-MeG) (Red=G-clamp)

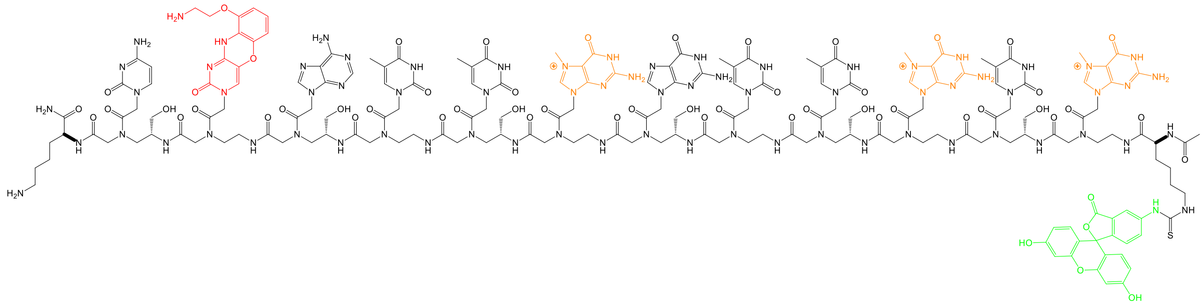

Chemical Formula: $C_{182}H_{229}N_{72}O_{56}S^{3+}$ **Exact Mass:** 4350.70 **Molecular Weight:** 4353.34

LC-MS (ESI): RT=1.44min. Absorbance at λ=260 nm. *m/z* expected for $[M+2H]^{5+}$: 870.95, *m/z* observed: 871.17; *m/z* expected for $[M+H]^{4+}$: 1088.43, *m/z* observed: 1088.33; *m/z* expected for $[M]^{3+}$: 1450.90, *m/z* observed: 1450.50.

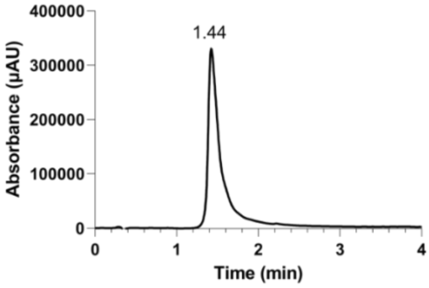

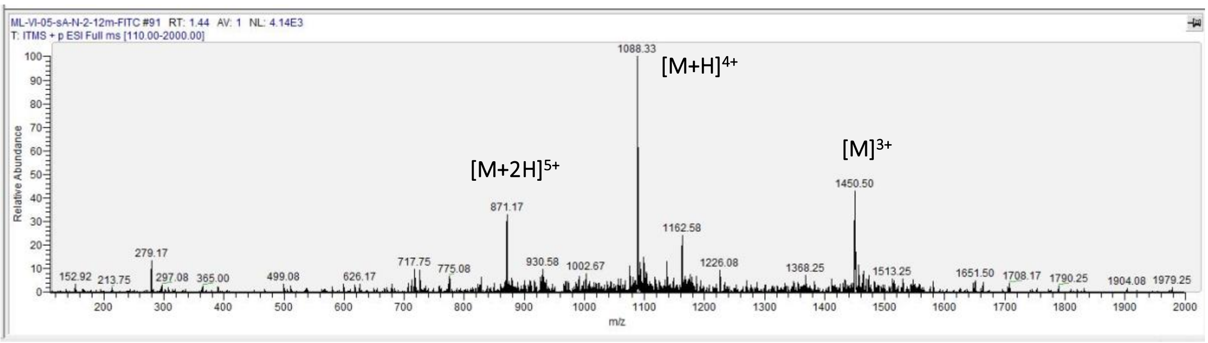

Chemical Formula: $C_{182}H_{229}N_{72}O_{56}S^{3+}$

MALDI-TOF: *m/z* cal: 4350.69 $[M-2H]^{+}$ *m/z* found: 4351.22

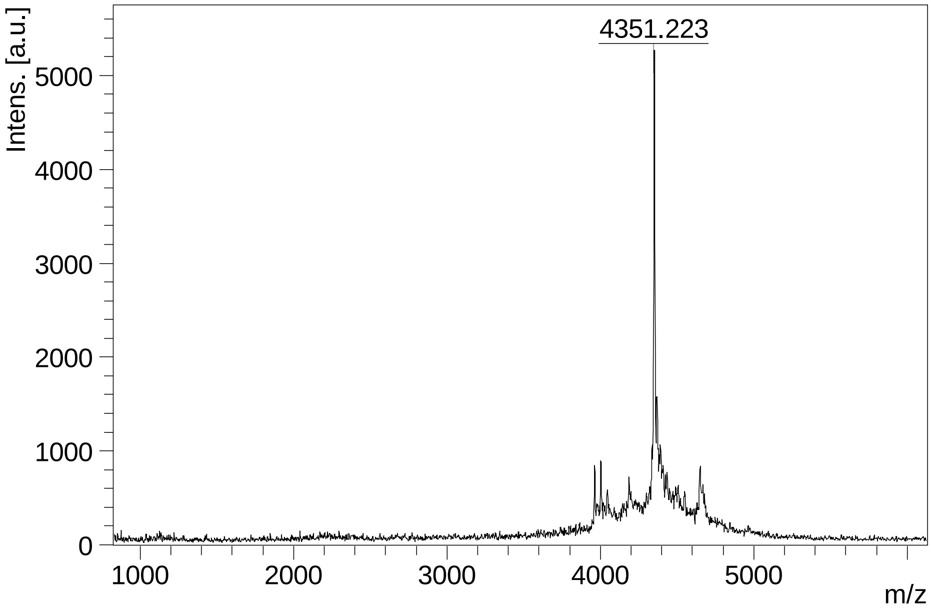

7. Dissociation constant (K_D) determination by FRET measurements

To obtain the dissociation constant (K_D) using steady-state FRET measurements, a series of FRET mixtures were prepared for each pair of donor/acceptor fluorophore-DNA/PNA conjugates. 30µL of x10 PS buffer pH 7.5 (200mM Tris·HCl, 10mM $MgSO_4$, 1.4M KCl, 200mM NaCl, 1mM $CaCl_2$, 0.2% tween-20) were mixed with both donor/acceptor fluorophore-DNA/PNA conjugates, volume added regarding the target final concentration of each from their corresponding stocks in MilliQ H_2O. The mixtures were brought up to 300 µL with MilliQ H_2O in Protein LoBind® eppendorfs, vortexed and 250 µL of the mixture were transferred into a black 96-well plate (ref.267342 – ThermoFisher). The concentration of the donor (FITC or Cy3) fluorophore-DNA/PNA conjugate was kept constant and, increasing concentrations of the acceptor (Cy3 or Atto647N) fluorophore-DNA/PNA conjugate were used to obtain the different data shown. The fluorescence emission experiments were performed in an automated manner at a fluorescence multi-well plate reader SpectraMax® i3.

As described below, each FRET mixture was excited with two different set of excitation wavelengths depending on the FRET fluorophore pair used and, the fluorescence emission spectra were recorded. The raw fluorescence emission signals were background-corrected and averaged between three experimental replicates for each condition. Each data point represents the mean and the associated RMSD of the three experimental replicates.

The reported methodology to measure the dissociation constant (K_D) by the steady-state FRET measurements by Song et al.[5] and its subsequent successful application by Chakraborty et al.[6] were followed for the experimental design and data processing. It was assumed one-to-one interaction between the donor fluorophore-DNA/PNA and the acceptor fluorophore-DNA/PNA conjugates yielding the adequate orientation and distance at the hybridized complex for FRET to occur. Equation 1 can be derived from the previous assumption:

$$K_d = \frac{[DNA/PNA_{Donor}]_{free} * [DNA/PNA_{Acceptor}]_{free}}{[DNA/PNA_{Donor}\text{-}DNA/PNA_{Acceptor}]} \quad (1)$$

In our FRET experiments we used two pairs of fluorophores with different excitation/ emission wavelengths, FITC/Cy3 and Cy3/Atto647N. Each DNA or PNA is conjugated to one the previous fluorophores.

In the case of FRET between FITC/Cy3, two excitation wavelengths, 468nm and 528nm, were used to excite the FRET mixture. Upon excitation at 468nm, the emission spectra showed two different peaks at 520nm and 562nm. The peak at 520nm corresponds to the unquenched FITC (FL_{DD}) upon its direct excitation, meanwhile the one at 562nm (FL_{DA}) has several different contributions. These contributions are divided in the unquenched FITC emission at 562 nm (α*FL_{DD}), the direct emission of Cy3 at 562 nm when excited at 468 nm (β*FL_{AA}) and, the emission of Cy3 due to the non-radiative energy transfer or Förster resonance energy transfer (FL_{FRET}) from FITC. When the FRET mixture is excited at 528nm only one peak is observed at 562 nm as expected, the fluorescence emission of Cy3 upon its direct excitation (FL_{AA}), and not FITC. So, the corresponding FRET emission could be calculated from the following equation:

$$FL_{FRET} = FL_{DA} - \alpha(FL_{DD}) - \beta(FL_{AA}) \quad (2)$$

Both parameters α and β are the so-called ratio constants or factors, which were calculated in independent experiments, and are specific for each fluorophore-DNA/fluorophore-PNA conjugate at a given temperature. These radiometric parameters allow subtraction of the two background contributions of the FL_{DA}, so a FL_{FRET} value is obtained for each FRET mixture.
When the other FRET pair Cy3/Atto647N was used, the two excitation wavelengths used were 525nm and 620 nm. In analogy to the FITC/Cy3 pair, the Cy3/Atto647N pair in the FRET mixture showed two distinct peaks when excited at 525 nm. A peak at 562 nm corresponding to the unquenched Cy3 emission (FL_{DD}) upon direct excitation and, a second one at 660 nm (FL_{DA}) containing several contributions. As expected, upon excitation of the FRET mixture at 620nm, only one peak at 660 nm corresponding to the fluorescence emission of the acceptor (FL_{AA}) upon direct excitation, and not Cy3. As for the other pair, Cy3/Atto647N PNA or DNA conjugates have their specific α and β ratio factors, allowing with the above equation the quantification of the FRET fluorescence of each FRET mixture.

Fluorescence measurements at FRET mixtures

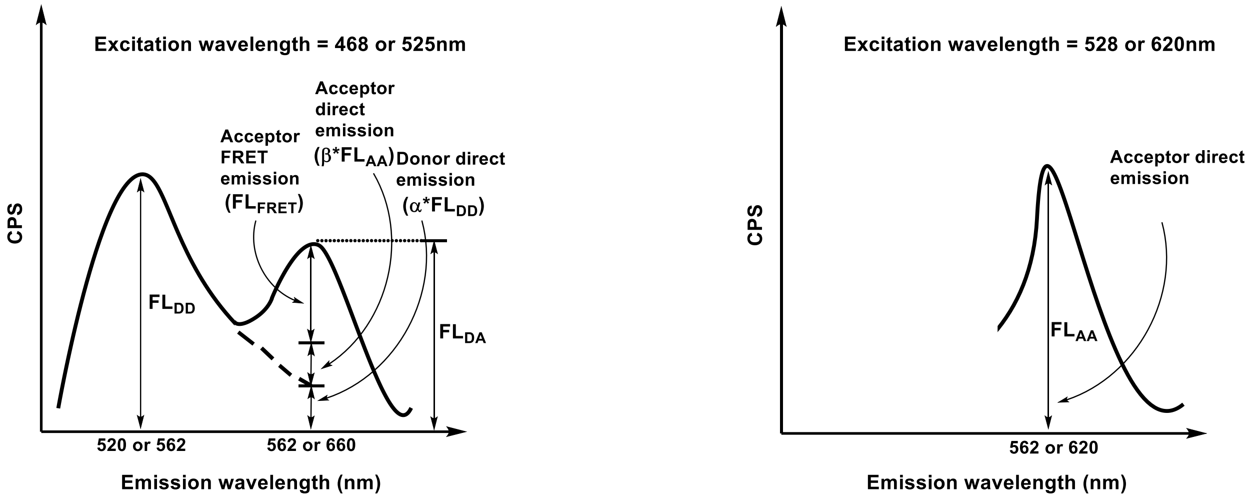

Figure SI7. Quantitative steady-state FRET analysis of the fluorescence emission on FRET mixtures. On the left, fluorescence emission pattern of a FRET mixtures when excited at the donor wavelenght, 468 or 525 nm (depending which FRET pairs used), with the three different contributions at the acceptor peak emission wavelength, 562 or 660 nm. On the right, fluorescence emission patter of a FRET mixture when excited at wavelength for direct excitation of the acceptor, 528 or 620nm, showing an emission peak, 562 or 620 nm, of major contribution by just the acceptor direct emission.

For each FRET mixture, after each of the three replicates was background corrected, a value of FL_{FRET} was calculated via equation 2 for all the different acceptor to donor ratios. A non-linear correlation was used to fit the datasets of FL_{FRET} vs acceptor concentrations (A) to obtain a maximum value of FRET emission ($FL_{FRETmax}$). Following deduced equation (3) by Song et al.[5] and previously applied by Chakraborty et al.[6], the same previous datasets were fitted thanks to the calculated $FL_{FRETmax}$ to obtain the K_D values from the steady-state FRET measurements:

$$FL_{FRET} = FL_{FRETmax}\left[\frac{A - D - K_d + \sqrt{(A - D - K_d)^2 + 4K_dA}}{A - D + K_d + \sqrt{(A - D - K_d)^2 + 4K_dA}}\right] \quad (3)$$

Datasets which were not suitable for obtaining an accurate value of $FL_{FRETmax}$ via non-linear correlation fitting, a value of $FL_{FRETmax}$ was empirically chosen to give the highest correlation when the datasets were fitted with equation 3. It is the case of the datasets which have not reached the FL_{FRET} plateau on the experimental range of donor/acceptor ratio.

7.1. Parameter α & β of PNA and DNA conjugates with FITC/Cy3/Atto647N

To measure the parameter α of each donor FITC-DNA conjugate, a series of concentrations of each donor FITC-DNA conjugate alone was prepared. Upon excitation at 468nm, the emission spectra were recorded from 498 to 640 nm. The emission at 562nm (FL_{DBA}) was divided by the emission at 520 nm (FL_{DD}). Then, the data was fitted by a non-linear regression to obtain a value of α for each donor FITC-DNA conjugate. Each sample was measured at 25°C and 37°C. The same principle was used to obtain the parameter α of each Cy3-PNA/DNA conjugate, a series of concentrations of each donor Cy3-PNA/DNA conjugate alone was prepared. Upon excitation at 525 nm, the emission spectra were recorded from 556 to 720 nm. The emission at 660 nm (FL_{DBA}) was divided by the emission at 562nm (FL_{DD}). Then, the data was fitted by a non-linear regression to obtain a value of α for each donor Cy3-PNA/DNA conjugate. Each sample was measured at 25°C and 37°C.

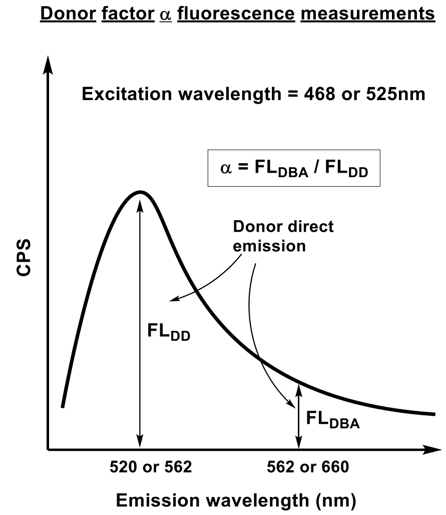

Figure S8. Quantification of the donor ratio parameter a by the fluorescence emission spectra when excited at the direct excitation donor wavelength, 468nm for FITC and 525 nm for Cy3, of the alone donor fluorophore-DNA/PNA conjugates

To measure the β parameter, in the same fashion as for parameter α, a series of concentration of each acceptor Cy3-PNA/DNA alone was prepared. The emission at 562 nm is recorded upon excitation at 468 nm and 528 nm. The emission at 562 nm (FL_{ABA}) when excited at 468 nm was divided by the emission at 562 nm when excited at 528 nm (FL_{AA}). Then, the data was fitted by a non-linear regression to obtain a value of β for each acceptor Cy3-PNA/DNA conjugate. Each sample was measured at 25°C and 37°C.

The same principle was used to obtain the parameter β of each Atto647N-PNA conjugate, a series of concentration of each acceptor Atto647N-PNA alone was prepared. The emission at 660 nm (FL_{ABA}) when excited at 525 nm was divided by the emission at 660 nm when excited at 620 nm (FL_{AA}). Then, the data was fitted by a non-linear regression to obtain a value of β for each acceptor Atto647N-PNA conjugate. Each sample was measured at 25°C and 37°C.

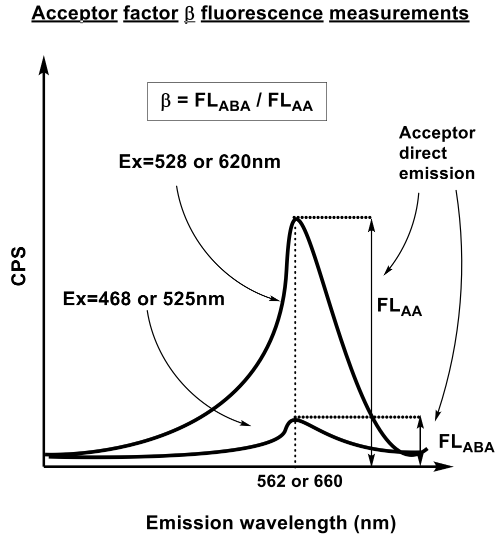

Figure S9. Quantification of the acceptor ratio parameter b of the alone acceptor fluorophore-DNA/PNA conjugates by the fluorescence emission spectra. Excited at the direct excitation donor wavelength, 468 nm for Cy3 and 525 nm for Atto647N, and at the direct excitation of the acceptor wavelenght, 528 nm for Cy3 and 620 for Atto647N, measuring the fluorescence emission intensity at the acceptor direct emission wavelength, 562 nm for Cy3 or 660 nm for Atto647N.

Results parameters α & β of PNA and DNA conjugates with FITC/Cy3/Atto647N

25°C	PNA-A-0	PNA-A-1	PNA-A-2	PNA-S-0	PNA-S-1	PNA-S-2	DNA-1	DNA-2	DNA-3	DNA-4
α Cy3-Atto647N	0,061 ± 0,001	0,060 ± 0,001	0,063 ± 0,001				0,064 ± 0,001	0,064 ± 0,001		
β Cy3-Atto647N				0,049 ± 0,001	0,055 ± 0,002	0,049 ± 0,001				
α FITC-Cy3									0,300 ± 0,003	0,308 ± 0,003
β FITC-Cy3	0,154 ± 0,001	0,146 ± 0,001	0,148 ± 0,001				0,141 ± 0,001	0,144 ± 0,002		

37°C	PNA-A-0	PNA-A-1	PNA-A-2	PNA-S-0	PNA-S-1	PNA-S-2	DNA-1	DNA-2	DNA-3	DNA-4
α Cy3-Atto647N	0,061 ± 0,001	0,061 ± 0,001	0,062 ± 0,001				0,063 ± 0,001	0,063 ± 0,001		
β Cy3-Atto647N				0,049 ± 0,001	0,052 ± 0,001	0,050 ± 0,001				
α FITC-Cy3									0,313 ± 0,002	0,320 ± 0,003
β FITC-Cy3	0,143 ± 0,001	0,142 ± 0,004	0,138 ± 0,002				0,142 ± 0,001	0,147 ± 0,001		

Parameters α & β of PNA-A-0

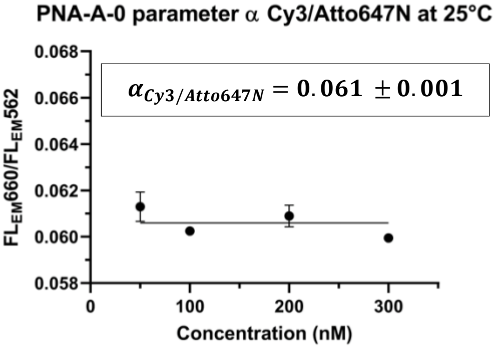

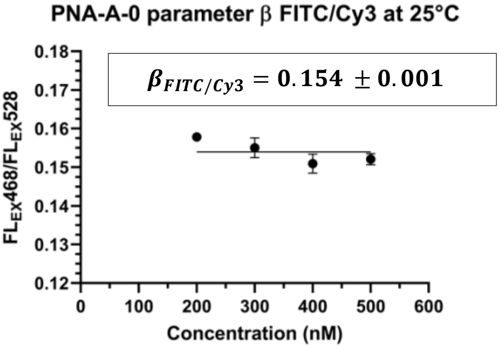

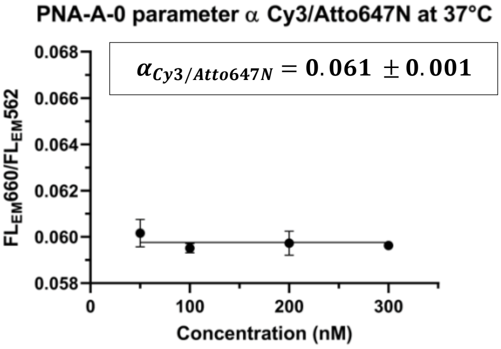

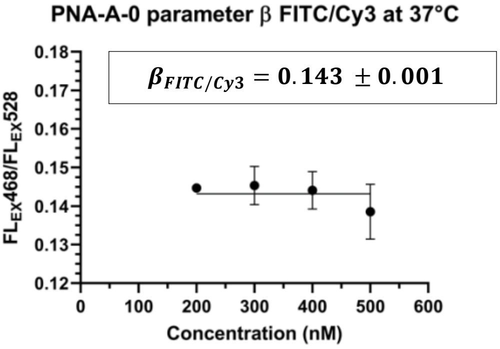

Parameters α & β of PNA-A-1

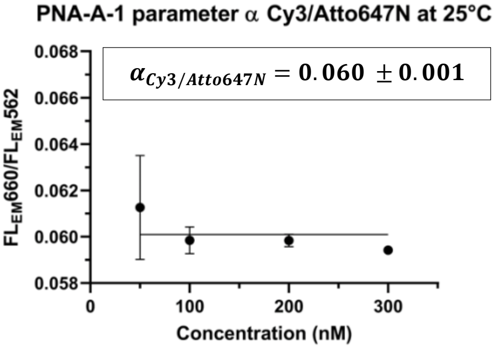

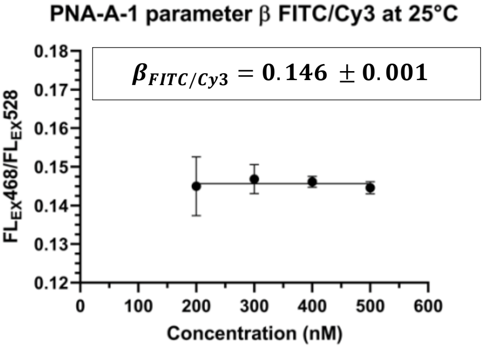

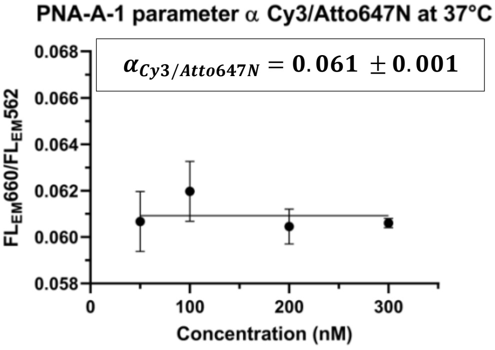

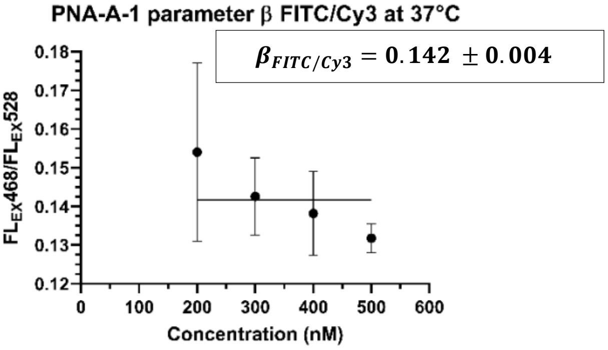

Parameters α & β of PNA-A-2

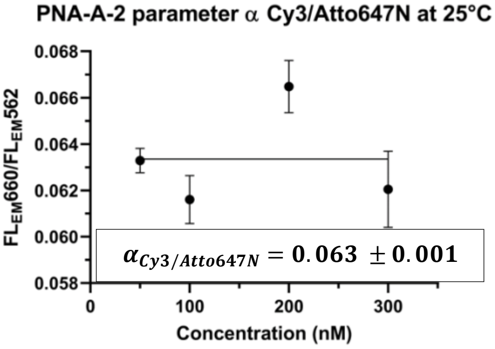

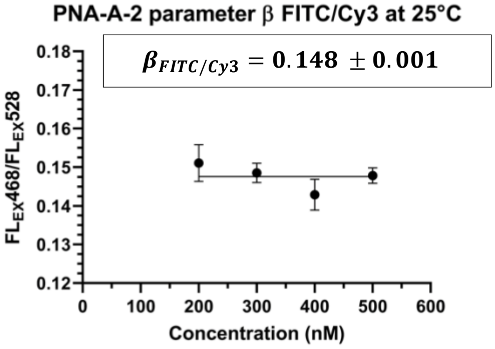

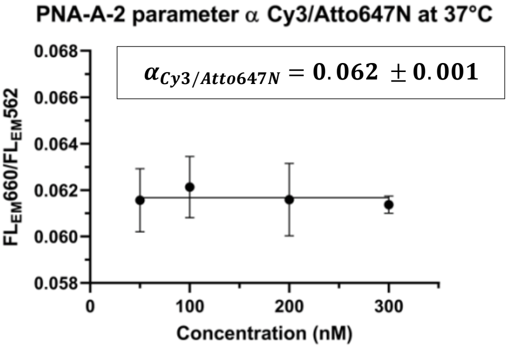

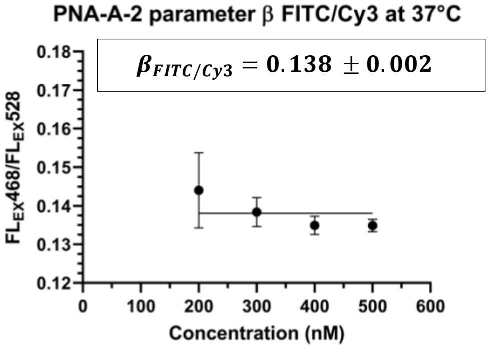

Parameters β of PNA-S-0

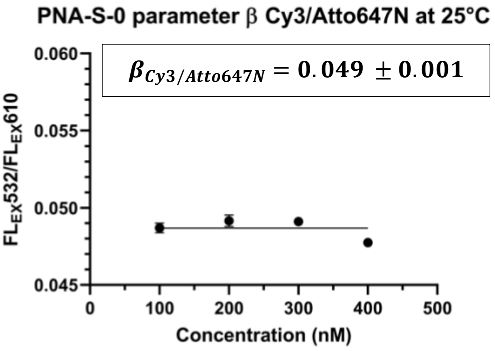

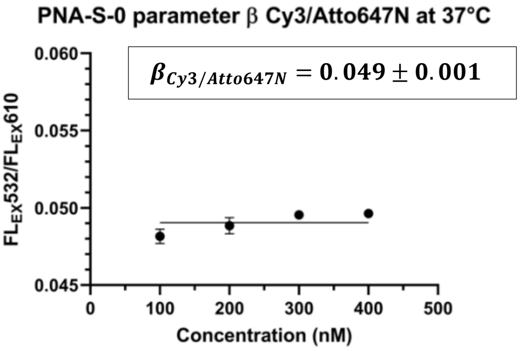

Parameters β of PNA-S-1

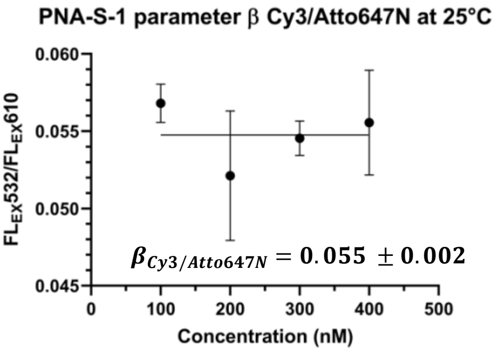

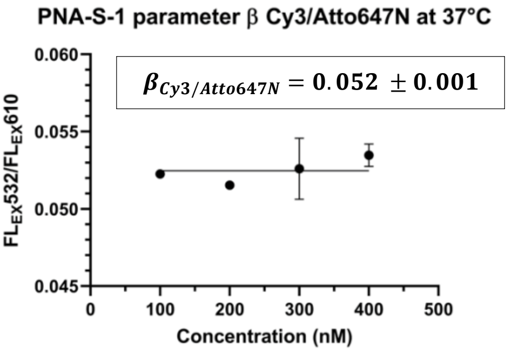

Parameters β of PNA-S-2

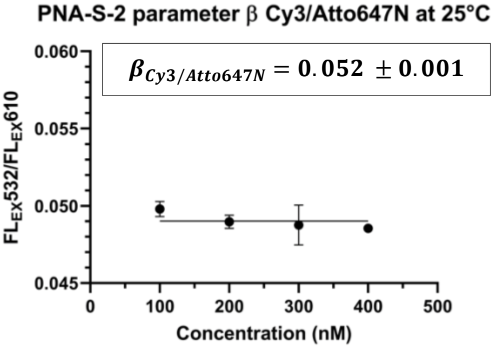

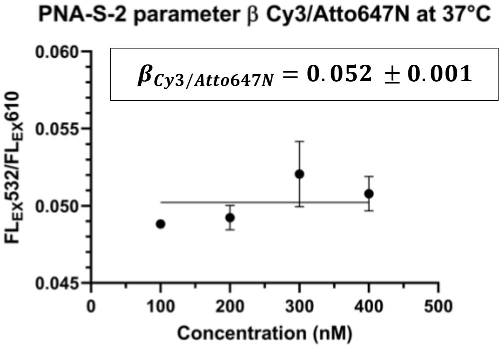

Parameters α & β of DNA-1

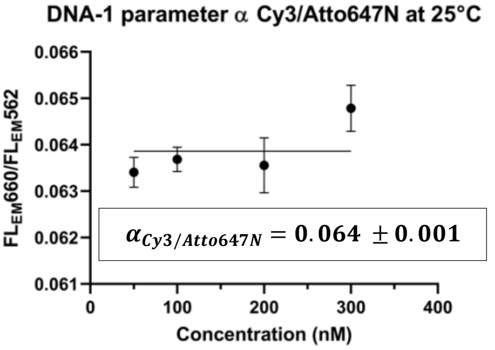

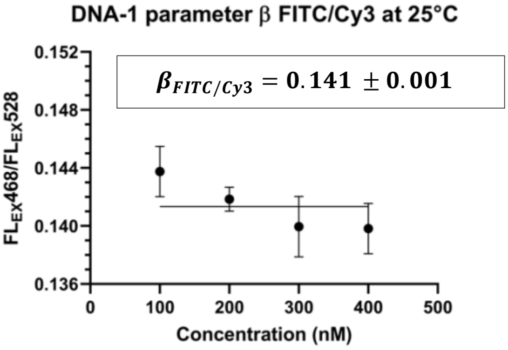

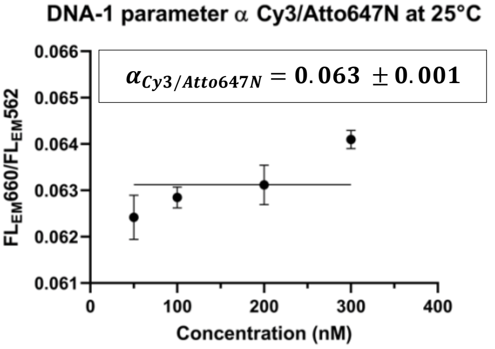

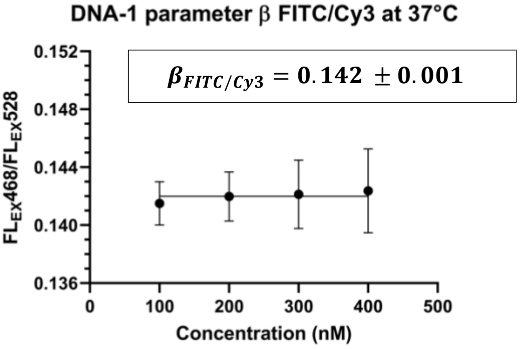

Parameters α & β of DNA-2

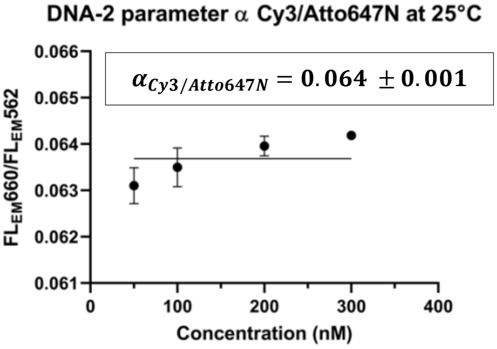

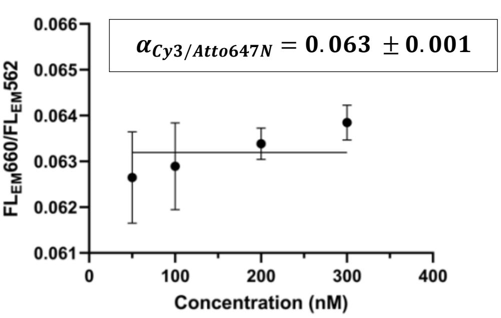

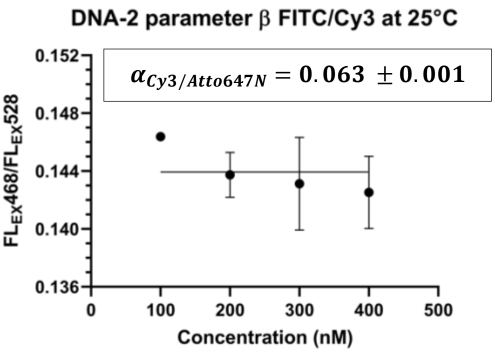

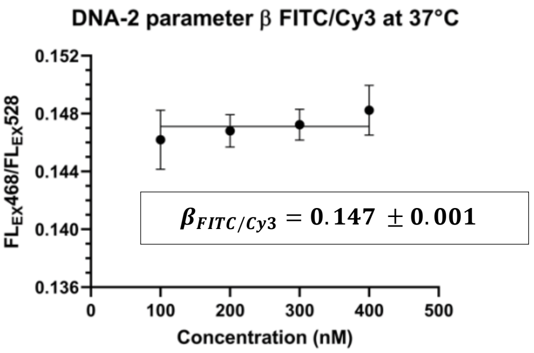

Parameters α of DNA-3

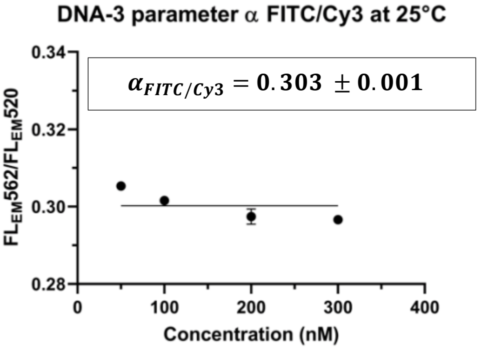

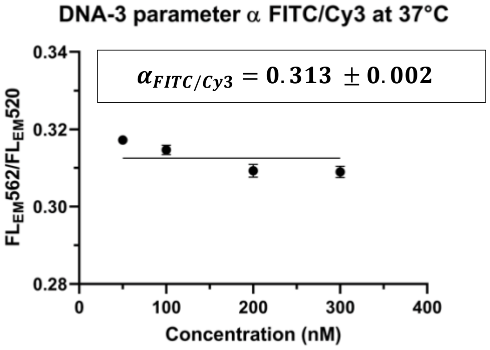

Parameters α of DNA-4

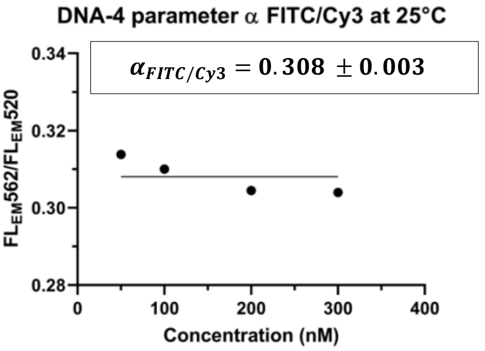

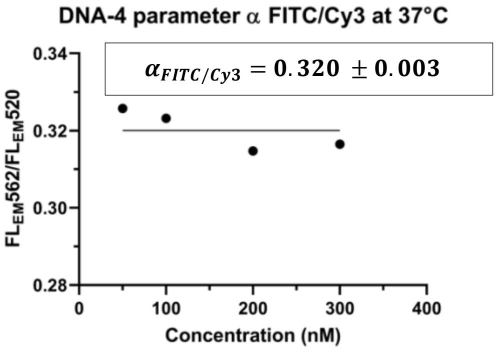

7.2. K_D of PNA and DNA conjugates with FITC/Cy3/Atto647N by FRET measurements

Results K_D of PNA/DNA conjugates with FITC/Cy3/Atto647N at 25°C and 37°C

25°C	PNA-S-0	PNA-S-1	PNA-S-2	DNA-3	DNA-4
PNA-A-0	13 ± 1 nM	18 ± 4 nM	7.8 ± 1,8 nM	270 ± 30 nM	> 2600 nM
PNA-A-1	4.8 ± 0.8 nM	94 ± 5 nM		20 ± 3 nM	> 1400 nM
PNA-A-2	4.1 ± 0.8 nM		> 5.4 µM	< 0,4 nM	8 ± 3 nM
DNA-1	280 ± 40 nM	830 ± 20 nM	1.3 ± 0.1 µM	> 75 µM	> 75 µM
DNA-2	6.0 ± 0.5 µM	5.4 ± 0.2 µM	7.4 ± 0.9 µM		

37°C	PNA-S-0	PNA-S-1	PNA-S-2	DNA-3	DNA-4
PNA-A-0	22 ± 1 nM	27 ± 6 nM	30 ± 8 nM	310 ± 90 nM	> 2600 nM
PNA-A-1	5.5 ± 1.1 nM	208 ± 11 nM		30 ± 3 nM	> 1400 nM
PNA-A-2	5.2 ± 1.0 nM		> 6.9 µM	< 0.6 nM	21 ± 4 nM
DNA-1	700 ± 140 nM	2.0 ± 0.1 µM	5.1 ± 0.5 µM	> 75 µM	> 75 µM
DNA-2	> 6 µM	7.5 ± 0.3 µM	>18 µM		

FRET measurements of DNA-1 and PNA-S-0

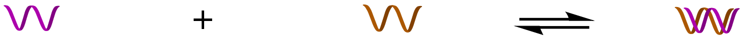

DNA-1: 5'-ACAACT-Cy3-3' PNA-S-0: C'-TGTTGA-Lys(Atto647N)Ac-N'

$$Concentration\ donor = 150\ nM$$

$$K_D = 280 \pm 40\ nM$$
$$R^2 = 0.97$$
$$FRET_{intensity\ MAX\ fitted} = 4.9 \pm 0.5 \times 10^6\ CPS$$

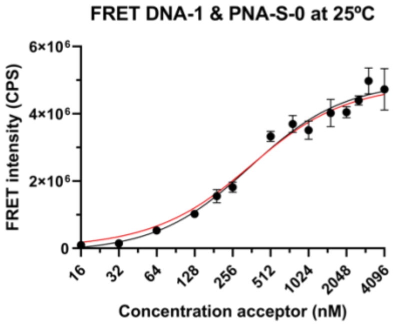

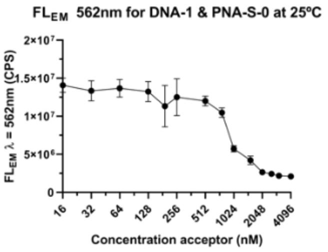

$$K_D = 700 \pm 140\ nM$$
$$R^2 = 0.90$$
$$FRET_{intensity\ MAX\ fitted} = 3.2 \pm 0.9 \times 10^6\ CPS$$

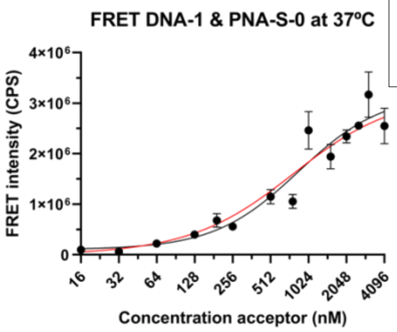

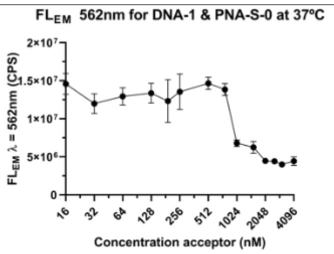

FRET measurements of DNA-2 and PNA-S-0

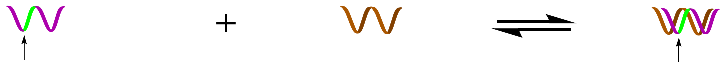

DNA-2: 5'-ATAACT- Cy3-3' PNA-S-0: C'-TGTTGA-Lys(Atto647N)Ac-N'

$$K_D = 6.0 \pm 0.5\ \mu M$$
$$R^2 = 0.95$$
$$FRET_{intensity\ MAX\ expected} = 4.9 \times 10^6\ CPS$$

$$Concentration\ donor = 150\ nM$$

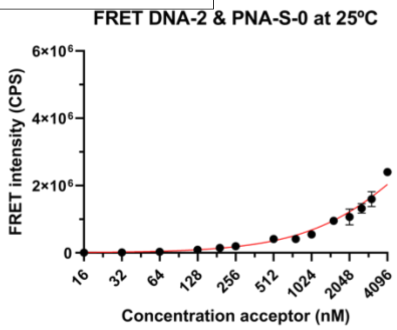

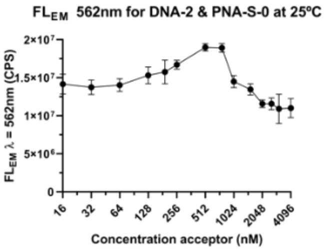

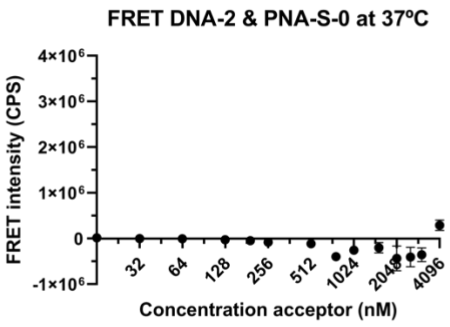

$K_D > 6\ \mu M$

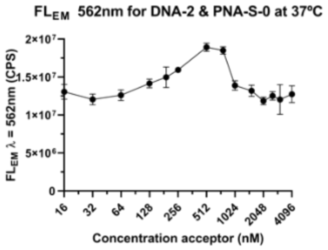

FRET measurements of DNA-3 and PNA-A-0

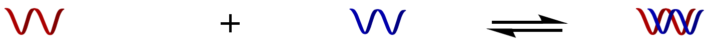

PNA-A-0: C'-Lys(TCAACA-NAc)Cy3-N'   DNA-3: 5'-FITC-AGTTGT-3'

$Concentration\ donor = 75\ nM$

$K_D = 270 \pm 30\ nM$
$R^2 = 0.95$
$FRET_{intensity\ MAX\ fitted} = 2.1 \pm 0.2 \times 10^6\ CPS$

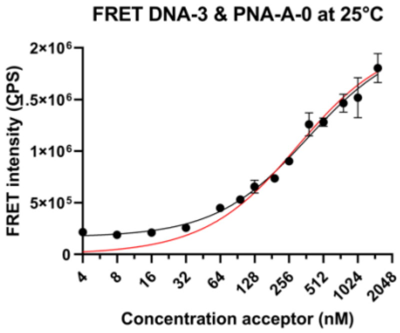

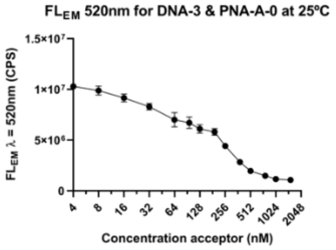

$K_D = 310 \pm 90\ nM$
$R^2 = 0.85$
$FRET_{intensity\ MAX\ expected} = 0.6 \times 10^6\ CPS$

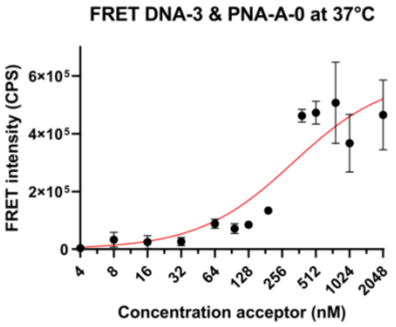

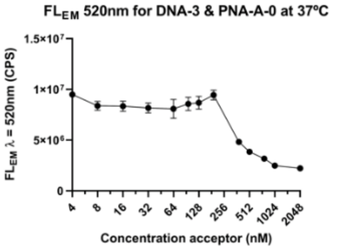

FRET measurements of DNA-4 and PNA-A-0

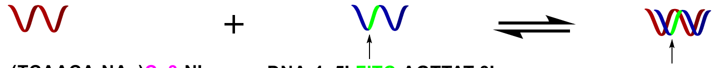

PNA-A-0: C'-Lys(TCAACA-NAc)Cy3-N'   DNA-4: 5'-FITC-AGTTAT-3'

$Concentration\ donor = 75\ nM$

$K_D > 2600\ nM$
$R^2 = 0.69$
$FRET_{intensity\ MAX\ expected} = 2.1 \times 10^6\ CPS$

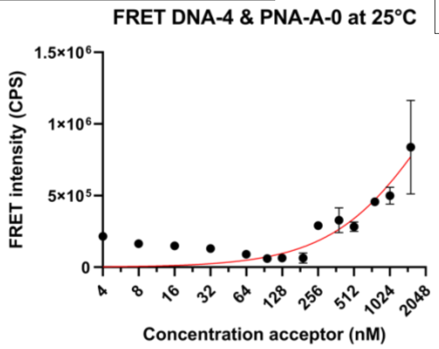

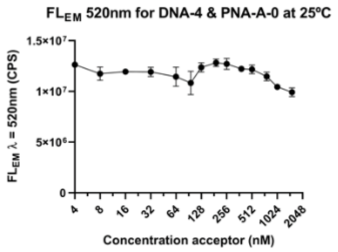

$K_d > 2600\ nM$

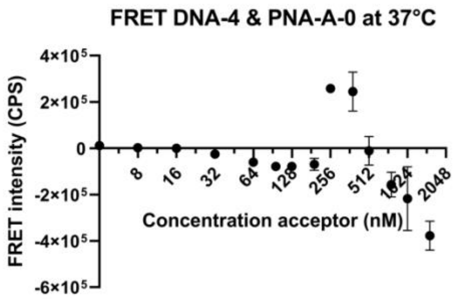

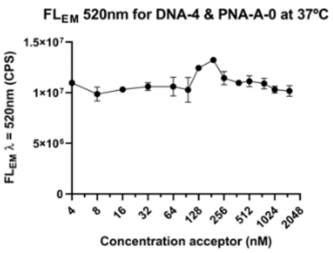

FRET measurements of DNA-1 and DNA-3

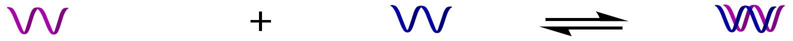

DNA-1: 5'-ACAACT-Cy3-3'   DNA-3: 5'-FITC-AGTTGT-3'

$Concentration\ donor = 300\ nM$

$K_D > 75\mu M$
$R^2 = 0.98$
$FRET_{intensity\ MAX\ expected} = 4 \times 10^8\ CPS$

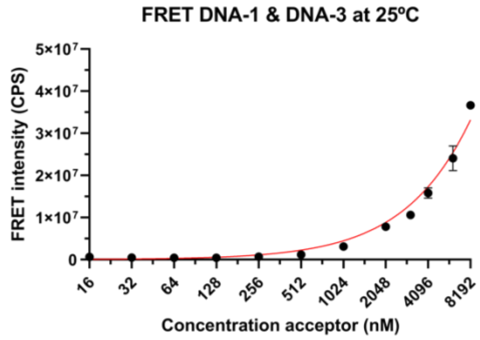

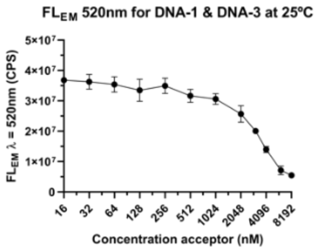

$$K_D > 75\mu M$$
$$R^2 = 0.86$$
$$FRET_{intensity\ MAX\ expected} = 4 \times 10^8\ CPS$$

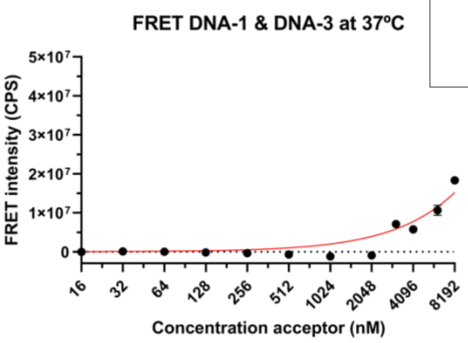

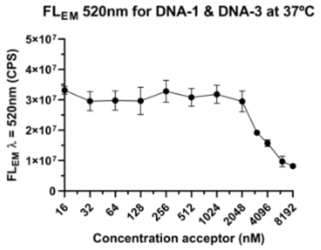

FRET measurements of DNA-1 and DNA-4

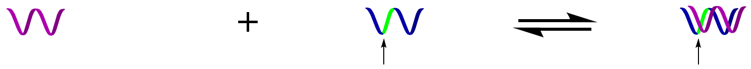

DNA-1: 5'-ACAACT-Cy3-3'

DNA-4: 5'-FITC-AGTTAT-3'

$$K_D > 75\mu M$$
$$R^2 = 0.98$$
$$FRET_{intensity\ MAX\ expected} = 4 \times 10^8\ CPS$$

$$Concentration\ donor = 300\ nM$$

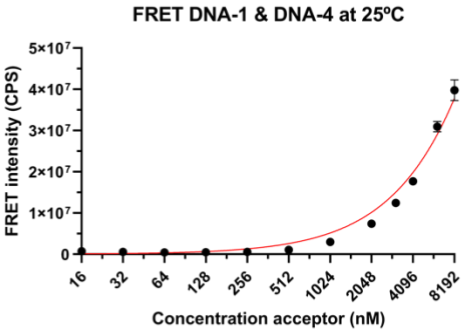

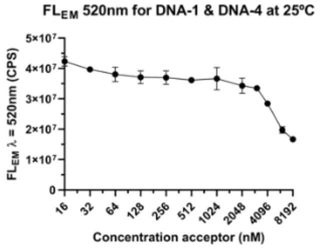

$$K_D > 75\mu M$$
$$R^2 = 0.87$$
$$FRET_{intensity\ MAX\ expected} = 4 \times 10^8\ CPS$$

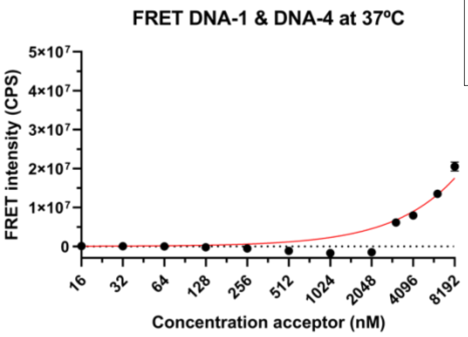

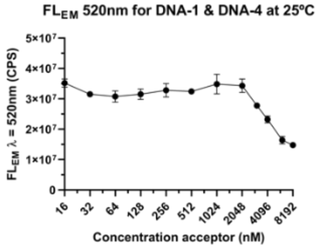

FRET measurements of DNA-1 and PNA-S-1

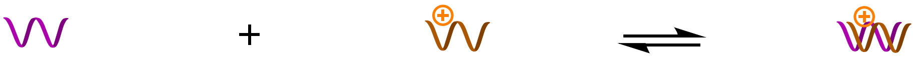

DNA-1: 5'-ACAACT-Cy3-3'   PNA-S-1: C'-TGTTGA-Lys(Atto647N)Ac-N' (G=N-7-MeG)

Concentration donor = 150 *nM*

$K_D = 830 \pm 20\ nM$
$R^2 = 0.99$
$FRET_{intensity\ MAX\ fitted} = 8.9 \pm 0.9 \times 10^6\ CPS$

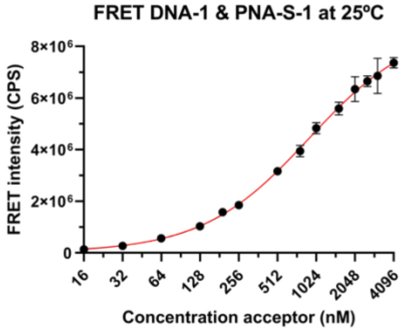

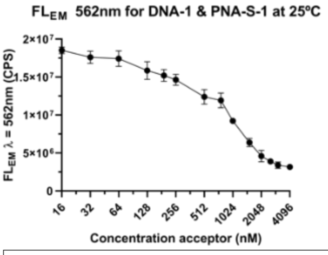

$K_D = 2.0 \pm 0.1\ \mu M$
$R^2 = 0.98$
$FRET_{intensity\ MAX\ fitted} = 8.0 \pm 2.9 \times 10^6\ CPS$

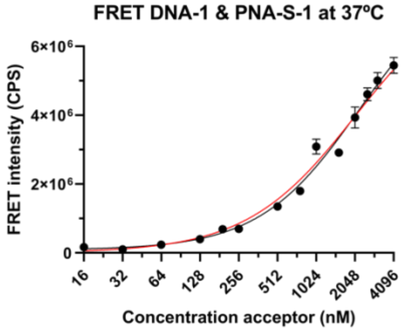

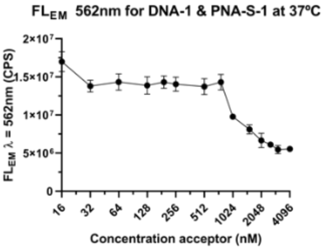

FRET measurements of DNA-2 and PNA-S-1

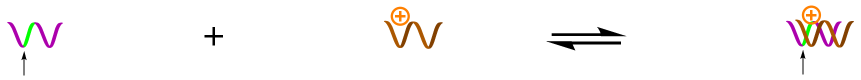

DNA-2: 5'-ATAACT-Cy 3-3'

PNA-S-1: C'-TGTTGA-Lys(Atto647N)Ac-N' (G=N-7-MeG)

Concentration donor = 150 *nM*

$K_D = 5.4 \pm 0.2\ \mu M$
$R^2 = 0.99$
$FRET_{intensity\ MAX\ expected} = 9 \times 10^6\ CPS$

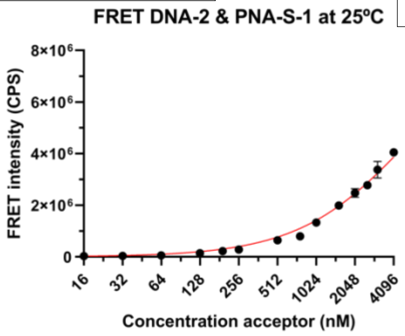

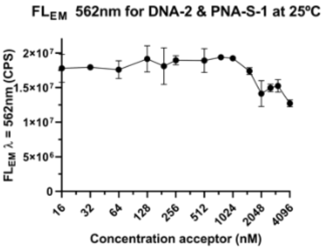

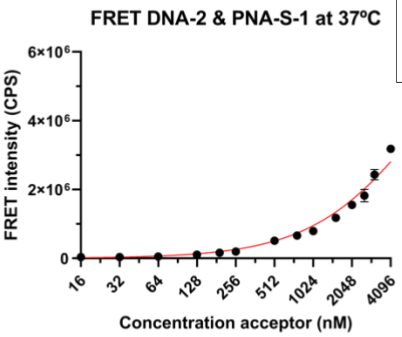

$K_D = 7.5 \pm 0.3\ \mu M$
$R^2 = 0.97$
$FRET_{intensity\ MAX\ expected} = 8 \times 10^6\ CPS$

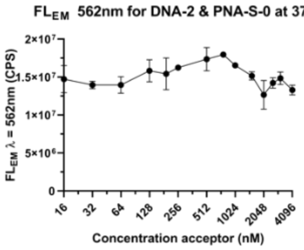

FRET measurements of DNA-3 and PNA-A-1

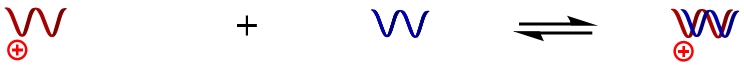

PNA-A-1: C'-Lys(TCAACA-NAc)Cy3-N' (C=G-clamp) DNA-3: 5'-FITC-AGTTGT-3'

$Concentration\ donor = 25\ nM$

$K_D = 20 \pm 3\ nM$
$R^2 = 0.98$
$FRET_{intensity\ MAX\ fitted} = 0.87 \pm 0.05 \times 10^6\ CPS$

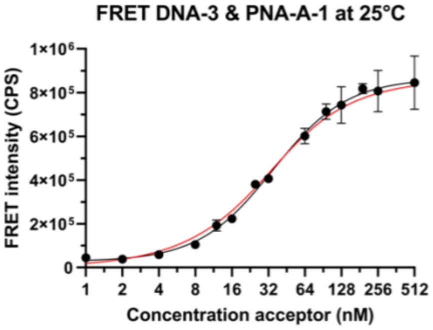

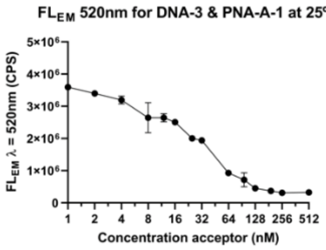

$K_D = 30 \pm 10\ nM$
$R^2 = 0.87$
$FRET_{intensity\ MAX\ expected} = 0.5 \times 10^6\ CPS$

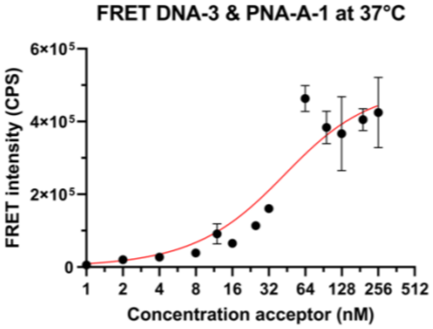

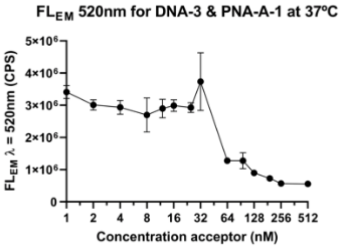

FRET measurements of DNA-4 and PNA-A-1

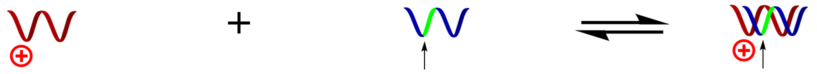

PNA-A-1: C'-Lys(TCAACA-NAc)Cy3-N' (C=G-clamp) DNA-4: 5'-FITC-AGTTAT-3'

$Concentration\ donor = 25\ nM$

$K_D > 1400\ nM$
$R^2 = 0.71$
$FRET_{intensity\ MAX\ expected} = 0.87 \times 10^6\ CPS$

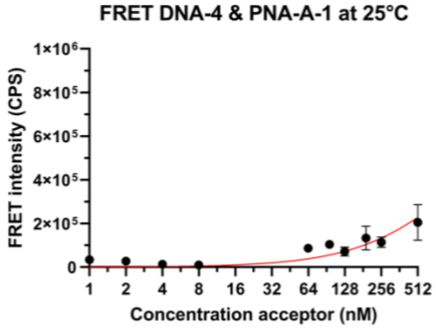

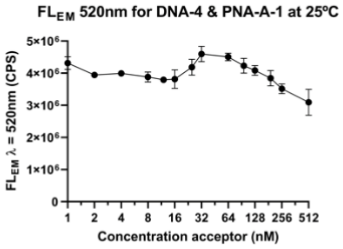

$K_D > 1400\ nM$

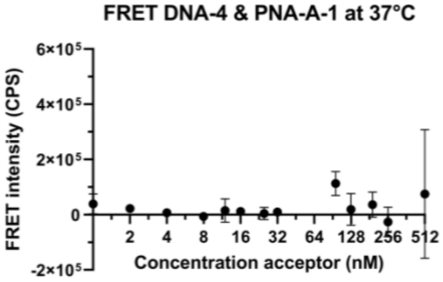

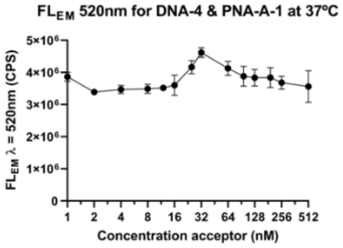

FRET measurements of DNA-1 and PNA-S-2

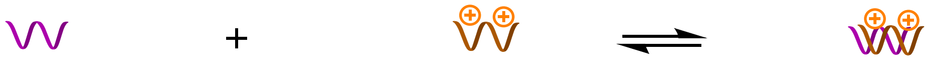

DNA-1: 5'-ACAACT-Cy3-3' PNA-S-2: C'-TGTTGA-Lys(Atto647N)Ac-N' (G=N-7-MeG)

$Concentration\ donor = 150\ nM$

$K_D = 1.3 \pm 0.1 \mu M$
$R^2 = 0.98$
$FRET_{intensity\ MAX\ expected} = 8 \times 10^6\ CPS$

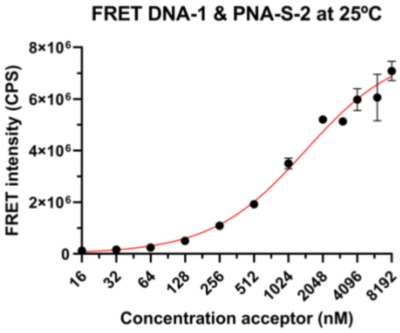

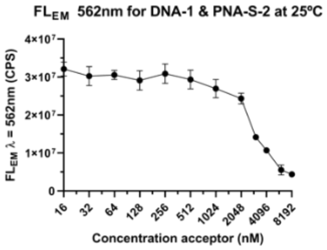

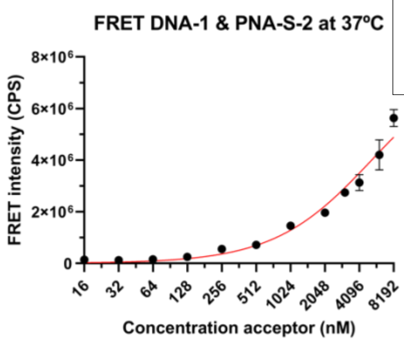

$K_D = 5.1 \pm 0.5 \mu M$
$R^2 = 0.97$
$FRET_{intensity\ MAX\ expected} = 8 \times 10^6\ CPS$

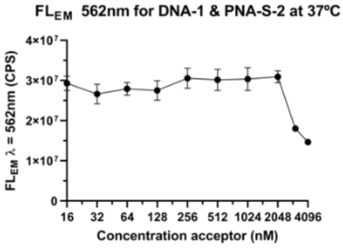

FRET measurements of DNA-2 and PNA-S-2

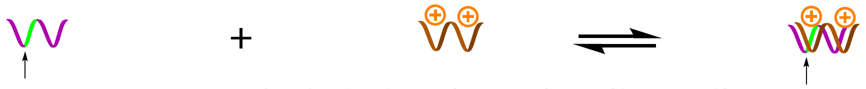

<u>DNA-2</u>: 5'-ATAACT- Cy3-3' <u>PNA-S-2</u>: C'-TGTTGA-Lys(Atto647N)Ac-N' (G=N-7-MeG)

$Concentration\ donor = 150\ nM$

$K_D = 7.4 \pm 0.9\ \mu M$
$R^2 = 0.94$
$FRET_{intensity\ MAX\ expected} = 8 \times 10^6\ CPS$

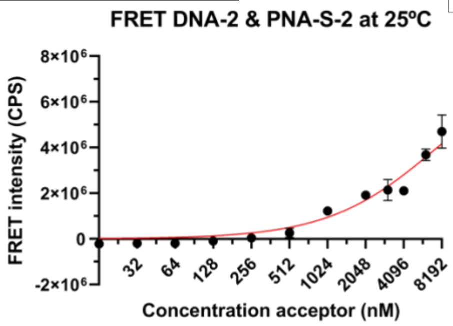

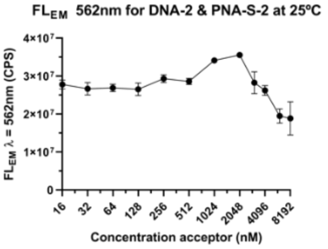

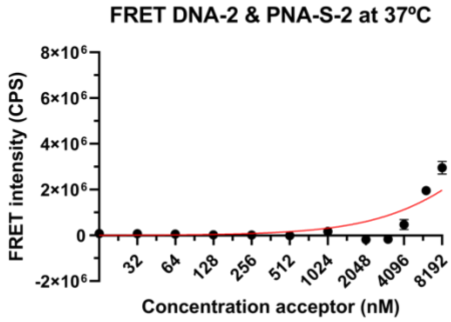

$K_D > 18\ \mu M$
$FRET_{intensity\ MAX\ expected} = 8 \times 10^6\ CPS$

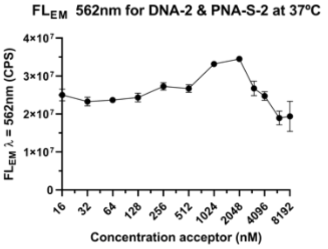

FRET measurements of DNA-3 and PNA-A-2

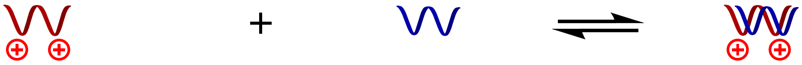

PNA-A-2:. C'-Lys(TCAACA-NAc)Cy3-N' (C=G-clamp) DNA-3: 5'-FITC-AGTTGT-3'

$Concentration\ donor = 25\ nM$

$K_D < 0.4\ nM$
$R^2 = 0.95$
$FRET_{intensity\ MAX\ fitted} = 0.47 \pm 0.02 \times 10^6\ CPS$

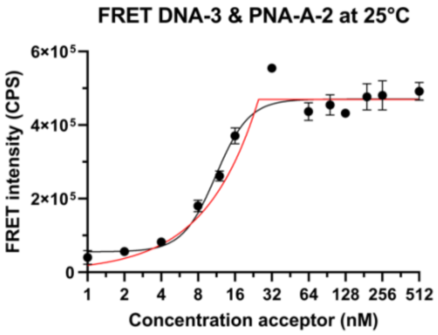

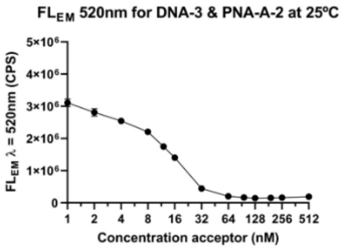

$K_D < 0.6\ nM$
$R^2 = 0.86$
$FRET_{intensity\ MAX\ fitted} = 0.41 \pm 0.02 \times 10^6\ CPS$

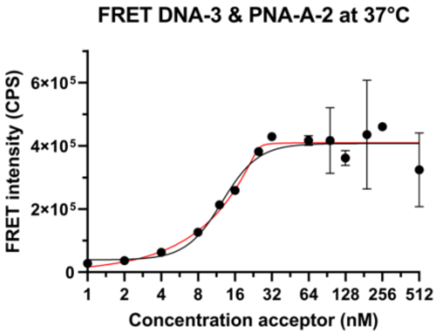

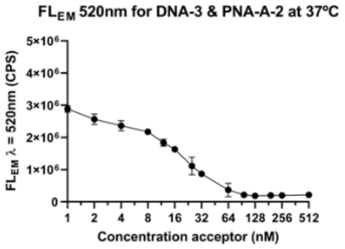

FRET measurements of DNA-4 and PNA-A-2

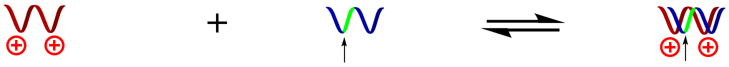

PNA-A-2:. C'-Lys(TCAACA-NAc)Cy3-N' (C=G-clamp) DNA-4: 5'-FITC-AGTTAT-3'

$Concentration\ donor = 25\ nM$

$K_D = 8 \pm 3\ nM$
$R^2 = 0.92$
$FRET_{intensity\ MAX\ fitted} = 0.63 \pm 0.07 \times 10^6\ CPS$

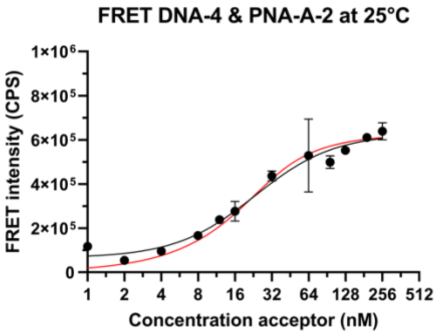

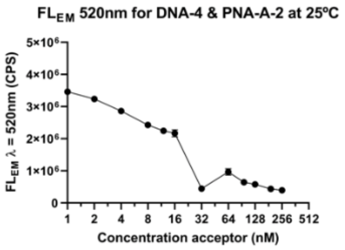

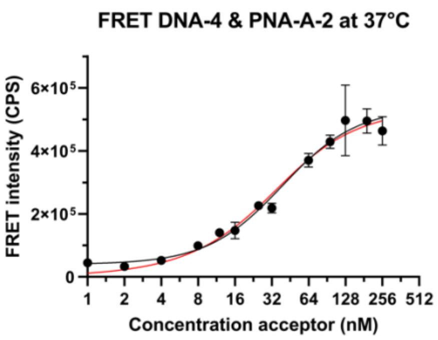

$$K_D = 21 \pm 4\ nM$$
$$R^2 = 0.95$$
$$FRET_{intensity\ MAX\ fitted} = 0.54 \pm 0.07 \times 10^6\ CPS$$

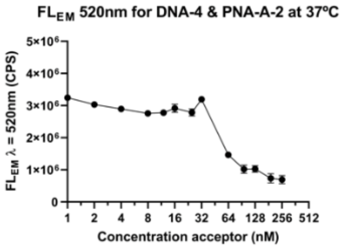

7.3. K_d of PNA and PNA conjugates with Cy3/Atto647N by FRET measurements

FRET measurements of PNA-A-0 and PNA-S-0

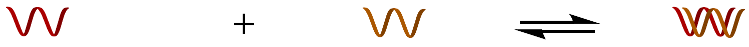

PNA-A-0: C'-Lys(TCAACA-NAc)Cy3-N' PNA-S-0: C'-TGTTGA-Lys(Atto647N)Ac-N'

$$Concentration\ donor = 5\ nM$$

$$K_D = 13 \pm 1\ nM$$
$$R^2 = 0.94$$
$$FRET_{intensity\ MAX\ expected} = 0.22 \times 10^6\ CPS$$

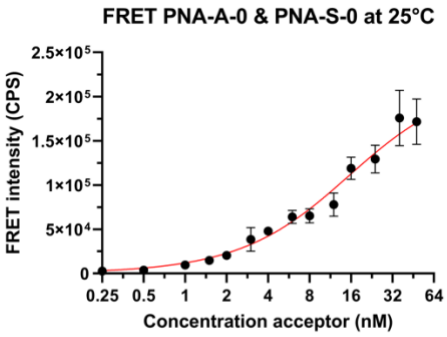

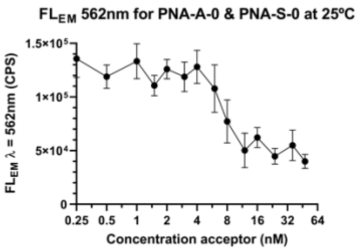

$$K_D = 22 \pm 1\ nM$$
$$R^2 = 0.95$$
$$FRET_{intensity\ MAX\ expected} = 0.26 \times 10^6\ CPS$$

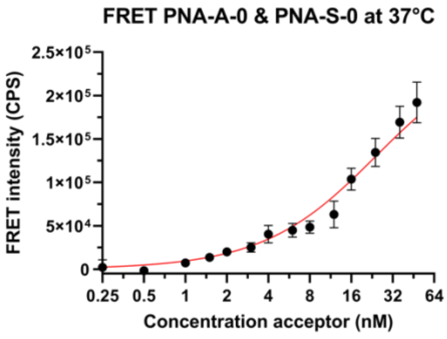

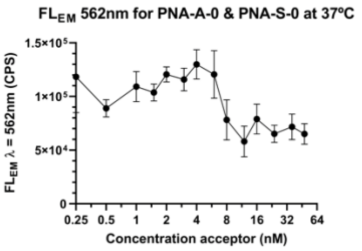

FRET measurements of PNA-A-1 and PNA-S-0

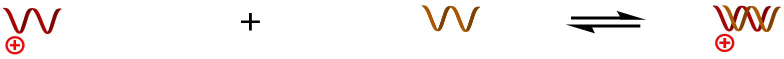

PNA-A-1: C'-Lys(TCAACA-NAc)Cy3-N' (C=G-clamp) PNA-S-0: C'-TGTTGA-Lys(Atto647N)Ac-N'

Concentration donor = 5 nM

$K_D = 4.8 \pm 0.8\ nM$

$R^2 = 0.89$

$FRET_{intensity\ MAX\ expected} = 0.23 \times 10^6\ CPS$

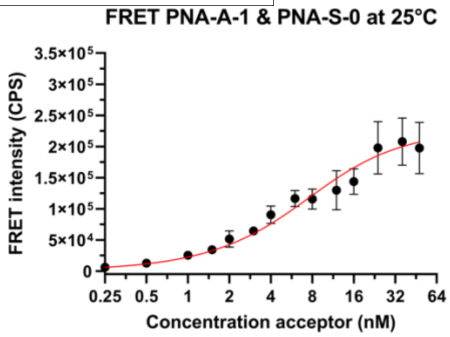

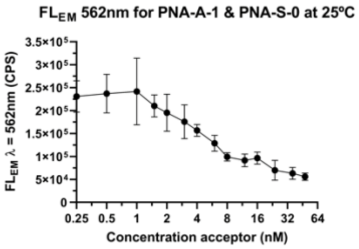

$K_D = 5.5 \pm 1.1\ nM$

$R^2 = 0.85$

$FRET_{intensity\ MAX\ expected} = 0.28 \times 10^6\ CPS$

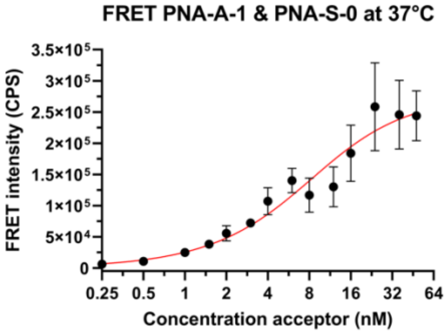

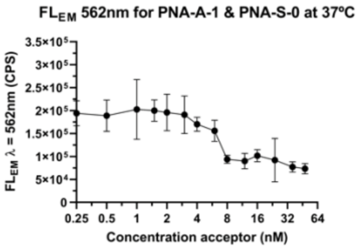

FRET measurements of PNA-A-0 and PNA-S-1

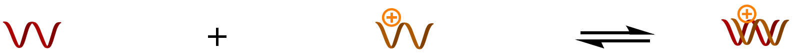

PNA-A-0: C'-Lys(TCAACA-NAc)Cy3-N' PNA-S-1: C'-TGTTGA-Lys(Atto647N)Ac-N' (G=N-7-MeG)

Concentration donor = 25 nM

$K_D = 18 \pm 4\ nM$

$R^2 = 0.93$

$FRET_{intensity\ MAX\ fitted} = 0.7 \pm 0.1 \times 10^6\ CPS$

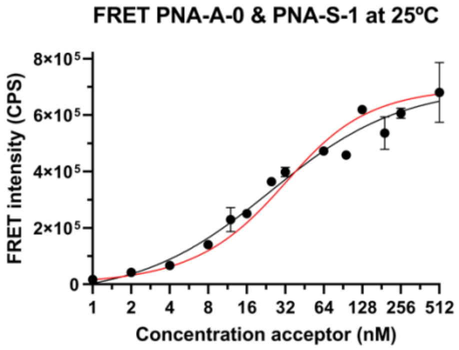

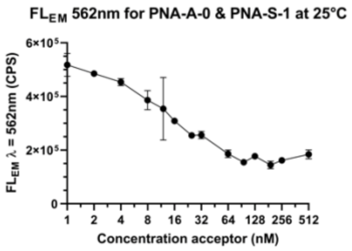

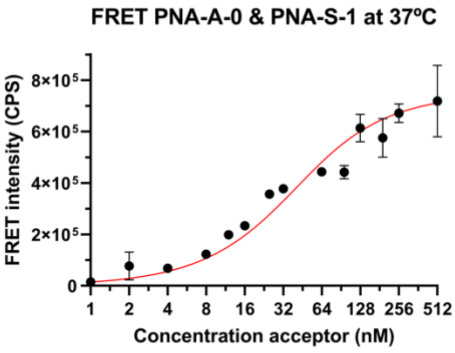

$$K_D = 27 \pm 6\ nM$$
$$R^2 = 0.92$$
$$FRET_{intensity\ MAX\ expected} = 0.75 \pm 0.1 \times 10^6\ CPS$$

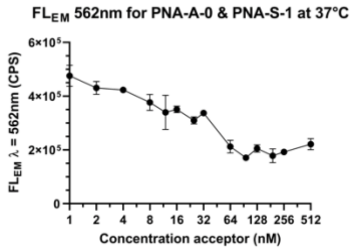

FRET measurements of PNA-A-1 and PNA-S-1

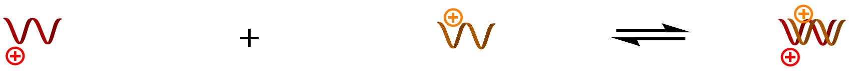

PNA-A-1: C'-Lys(TCAACA-NAc)Cy3-N' (C=G-clamp) PNA-S-1: C'-TGTTGA-Lys(Atto647N)Ac-N' (G=N-7-MeG)

$$Concentration\ donor = 25\ nM$$

$$K_D = 94 \pm 5\ nM$$
$$R^2 = 0.93$$
$$FRET_{intensity\ MAX\ fitted} = 1.58 \pm 0.15 \times 10^6\ CPS$$

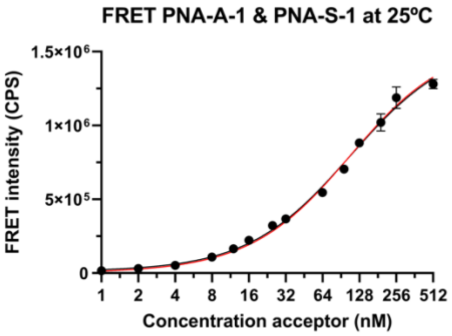

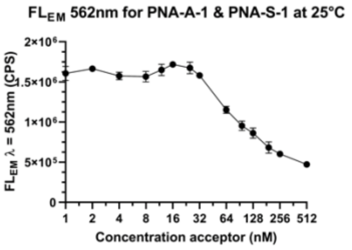

$$K_D = 208 \pm 11\ nM$$
$$R^2 = 0.99$$
$$FRET_{intensity\ MAX\ expected} = 1.8 \times 10^6\ CPS$$

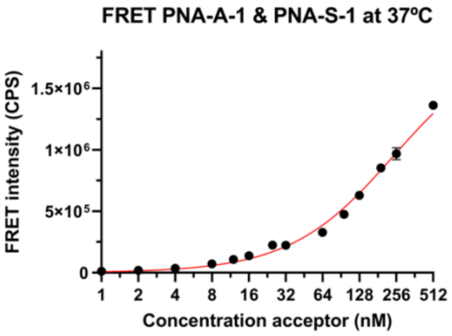

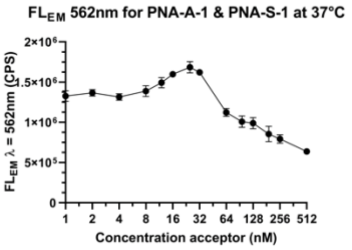

FRET measurements of PNA-A-2 and PNA-S-0

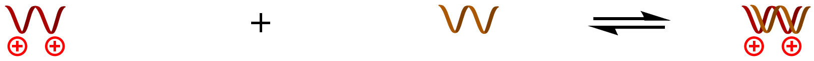

PNA-A-2:. C'-Lys(TCAACA-NAc)Cy3-N' (C=G-clamp) PNA-S-0: C'-TGTTGA-Lys(Atto647N)Ac-N'

$$Concentration\ donor = 5\ nM$$

$$K_D = 4.1 \pm 0.8\ nM$$
$$R^2 = 0.88$$
$$FRET_{intensity\ MAX\ fitted} = 0.31 \pm 0.04 \times 10^6\ CPS$$

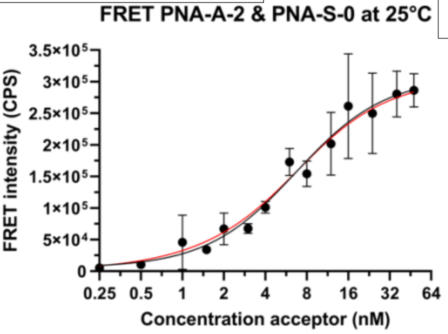

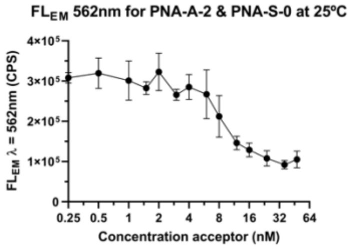

$$K_D = 5.2 \pm 1.0\ nM$$
$$R^2 = 0.90$$
$$FRET_{intensity\ MAX\ fitted} = 0.43 \pm 0.06 \times 10^6\ CPS$$

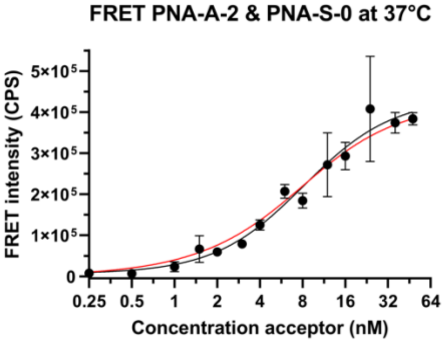

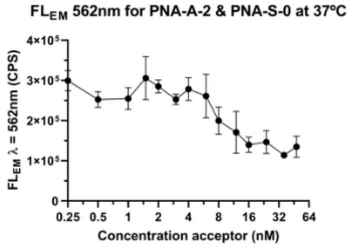

FRET measurements of PNA-A-0 and PNA-S-2

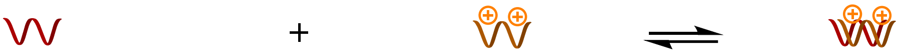

PNA-A-0: C'-Lys(TCAACA-NAc)Cy3-N' PNA-S-2: C'-TGTTGA-Lys(Atto647N)Ac-N' (G=N-7-MeG)

$$Concentration\ donor = 25\ nM$$

$$K_D = 7.8 \pm 1.8\ nM$$
$$R^2 = 0.97$$
$$FRET_{intensity\ MAX\ fitted} = 0.39 \pm 0.03 \times 10^6\ CPS$$

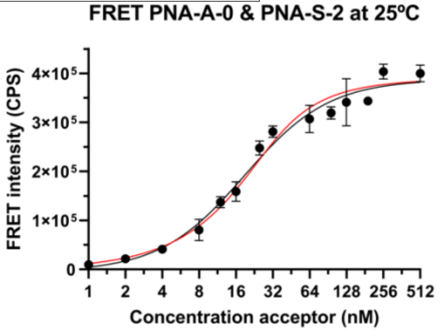

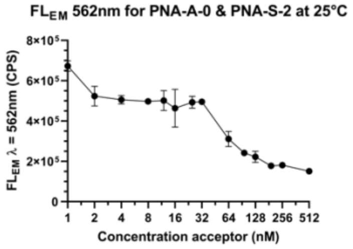

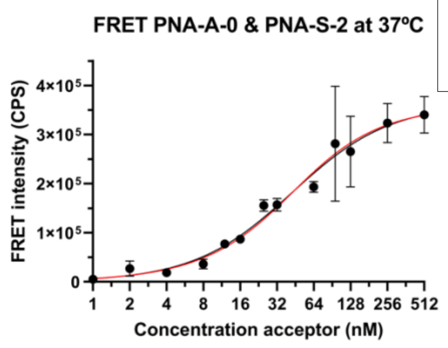

$$K_D = 30 \pm 8\ nM$$
$$R^2 = 0.90$$
$$FRET_{intensity\ MAX\ fitted} = 0.36 \pm 0.08 \times 10^6\ CPS$$

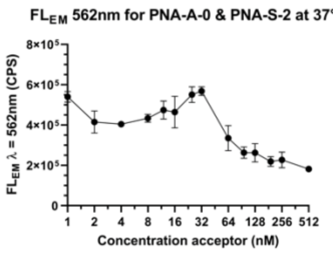

FRET measurements of PNA-A-2 and PNA-S-2

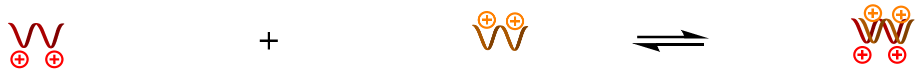

PNA-A-2:. C'-Lys(TCAACA-NAc)Cy3-N' (C=G-clamp) PNA-S-2: C'-TGTTGA-Lys(Atto647N)Ac-N' (G=N-7-MeG)

$$Concentration\ donor = 300\ nM$$

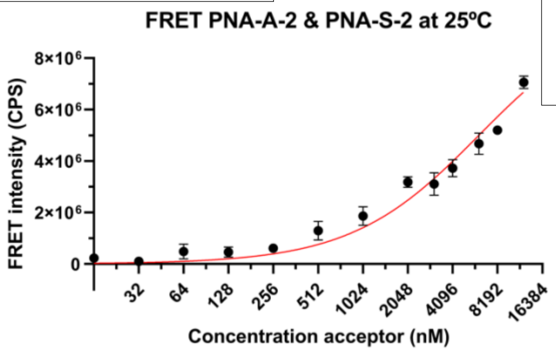

$$K_D > 5.4\ \mu M$$
$$R^2 = 0.96$$
$$FRET_{intensity\ MAX\ expected} = 1.0 \times 10^7\ CPS$$

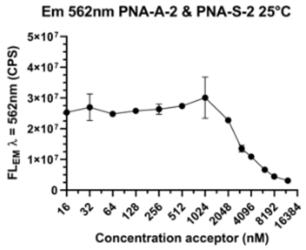

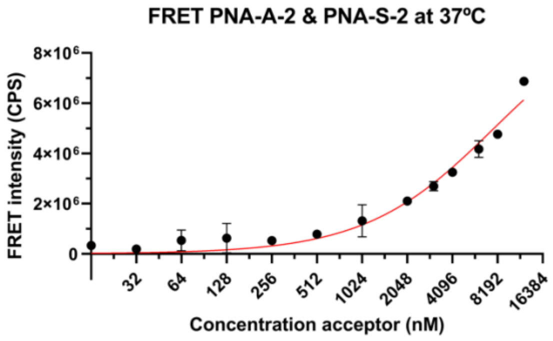

$$K_D > 6.9\ \mu M$$
$$R^2 = 0.96$$
$$FRET_{intensity\ MAX\ expected} = 1.0 \times 10^7\ CPS$$

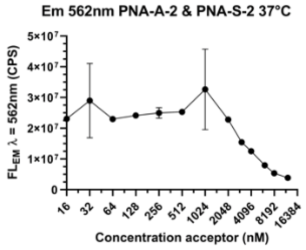

8. Protocols

8.1. Protocols for amplification and detection of SARS-CoV-2 viral RNA at the ORF1 region

RPA amplification SARS-CoV-2 viral RNA at the ORF1 region
Following the general procedure of the RPA TwistAmp Basic kit (TwistDx: TABAS03KIT), RPA was carried out in a PCR tube in a final volume of 50 µL and 500 nM of primers. The amplification was initiated as follows: 30 µL of primer-free rehydration buffer were mixed with 9.5 µL of water, 2.5 µL of 10 µM Cy3 labeled forward primer (5'-Cy3-GTGGCGGTTCAC-TATATGTTAAACCAGGTGGAA-3') and 2.5 µL of 10 µM phosphorylated reverse primer (5'P-ATTGGCCGTGACAGCTTGACAAATGTTAAAAAC-3'). This solution was transferred to a TwistAmp Basic reaction kit followed by adding 1 µL of analyte solution (DNA template at 4pM – 4.8e4 copies DNA/µL) or 1 µL water, and 2.5 µL of $Mg(OAc)_2$ 280 mM. The reaction was incubated at 41°C for 30 min.

The formation of the desired DNA was followed by gel analysis with either 15% Native PAGE or 2.5% agarose gel. The DNA was visualized by SYBR Gold Nucleic acid staining.

The 135nb ssDNA template used for RPA amplification corresponds to the nucleotides 15 418-15 554 of the SARS-CoV-2 genome (Wuhan-Hu-1 /NCBI reference: NC_045512.2) placed at the ORF1 region (135nbDNA template, 5'-GCTCAAGTATTGAGTGAAATGGTCATGTGTGG-CGGTTCACTATATGTTAAACCAGGTGGAACCTCATCAGGAGATGCCACAACTGCTTATGCTAATAGT GTTTTTAACATTTGTCAAGCTGTCACGGCCAATGTT-3'). The designed primer set amplifies the following 105nb DNA sequence (5'- GTGGCGGTTCACTATATGTTAAACCAGGTGGAACCTCATC-AGGAGATGCCACAACTGCTTATGCTAATAGTGTTTTTAACATTTGTCAAGCTGTCACGGCCAATG-3').

For RT-RPA, the same procedure was used starting with 1µL RNA analyte (Wuhan coro-navirus 2019 RdRP gene control obtained from Charité/EVAg – 1e5 copies/µL or 1e3 µL stocks) or 1 µL water, with 1 µL of RevertAid Reverse Transcriptase (Thermo Fisher Scientific, 200 U/ µL, Ref: EP0441), 1 µL of Recombinant RNasin Ribonuclease Inhibitor (Promega, 40 U/µL,Ref: N251A) and the RT-RPA reaction was carried out for a total of 30 min at 41°C.

Invasion and detection of the viral RNA sequence SARS-CoV-2 viral RNA at the ORF1 region

For the invasion experiments coupled with LFA read out, 20 µL of the RT-RPA mixture (500 nM primers/dsDNA amplicon) after 30 min at 41°C was transferred into a Protein LoBind® Eppendorf containing 130µL of the PNA invasion MIX (115 nM of each indicated PNA, 2.30 µM 22-nt ssDNA scavengers: 5'-TCTGCAGGTCGACTCTAGAAAA-3' and 5'-TAGCTTATC AGACTGATGTTGA-3', 1.15x PS buffer). The resulting 150 µL mixture [66.7 nM primers/dsDNA amplicon (1 eq), 100nM each PNA (1.5 eq), 2 µM 22nt-ssDNA scavengers (30eq), 1x PS buffer] was heated at 95°C for 5 min. Ratio of PNA/DNA calculated assuming complete amplification of the primers. The RPA invasion mixture was then centrifuged for 5 min at 14k rpm.

Then, 15 µL of the supernatant were diluted up to 100 µL with LFA buffer (Milenia GenLine HybriDetectRef:MGHD 1) in a Protein LoBind® Eppendorf before adding the LFA strip (Milenia GenLine HybriDetectRef:MGHD 1). All the LFA strips were imaged after 10 min.

The formation of the desired PNA-DNA invasion complex was also followed by gel analysis with 15% Native PAGE at 4°C and 20V/cm. The DNA was visualized by SYBR Gold Nucleic acid staining.

PCR amplification and purification dsDNA amplicon protocols

PCR amplification was carried out in a x96 PCR well plate in a final volume of 50 µL and 500nM of primers. The amplification was initiated as follows: 5 µL of Standard Taq Reaction Buffer (NEB, Ref: B9014SVIAL) were mixed with 40.5 µL of water, 1 µL dNTPs 10mM solution Mix (NEB, Ref: N0447S), 1 µL of 10µM Cy3 labeled forward primer (5'-Cy3-GTGGCGGTTCAC-TATATGTTAAACCAGGTGGAA-3'), 1 µL of 10µM phosphorylated reverse primer (5'P-ATTGGCCGTGACAGCTTGACAAATGTTAAAAAC-3') and Taq DNA Polymerase 5 U/µL (NEB, Ref: M0273E). This solution was transferred into a well followed by adding 1 µL of analyte solution (DNA template at 4pM – 4.8e4 copies DNA/µL). The x96 plate was filled with the samples, sealed, and placed at the thermocycler for 25 PCR cycles (initial denaturation 30s at 95°C; cycle: 30s at 95°C, 50s at 50°C, 60s at 68°C).

The 96 reactions were pulled together, ethanol precipitated and purified by QIAquick PCR Purification Kit (QIAGEN, ref: 28104). The purified 105nb dsDNA amplicon was eluted in water and used directly for the invasion optimization experiments.

The formation of the desired DNA was followed by gel analysis with either 15% Native PAGE or 2.5% agarose gel. The DNA was visualized by SYBR Gold Nucleic acid staining.

Invasion G^{+}-C^{+} pcPNAs versus cPNAs at RPA mixtures for 105bp amplicon

For the invasion experiments, the previous RPA amplifications were performed using the same conditions described above but using double concentration of the primers. No effect on the outcome of the RPA amplification was observed.

For Figure S7, 10 µL of the RPA mixture (1 µM primers/dsDNA amplicon) after 30 min at 41°C was transferred into a Protein LoBind® Eppendorf containing 140 µL of the PNA invasion MIX (36 nM of each indicated PNA, 1.15x PS buffer). The resulting 150 µL mixture [66.7 nM primers/dsDNA amplicon (1 eq), 33.4nM each PNA (0.5 eq), 1x PS buffer] was heated at 95°C for 5 min.

The RPA invasion mixture was then centrifuged for 5 min at 14k rpm. The formation of the desired PNA-DNA invasion complex was followed by gel analysis of the supernatant with 15% Native PAGE at 4°C and 20V/cm. The DNA was visualized by SYBR Gold Nucleic acid staining.

Filtration vs centrifugation for invasion and detection of the SARS-CoV-2 viral RNA (ORF1 region)

RT-RPA was performed as described above (50 µL RT-RPA reaction volume). Each reaction was split into two by taking 20 µL of the RT-RPA mixture (500nM primers/dsDNA amplicon) after 30 min at 41°C into a Protein LoBind® Eppendorf containing 130 µL of the PNA invasion MIX (115 nM of each indicated PNA, 46 ng/µL 20-*N* ssDNA scavenger, 1.15x PS buffer). The resulting 150 µL mixture [66.7 nM primers/dsDNA amplicon (1 eq), 100 nM each PNA (1.5 eq), 40 ng/µL 20-*N* ssDNA scavenger, 1x PS buffer] was heated at 95°C for 5 min. Ratio of PNA/DNA calculated assuming complete amplification of the primers. RPA invasion mixtures were either filtrated through a handmade Kimtech plug (see section 10 for details) or centrifuged for 5 min at 14k rpm.

Then, 15 µL of the resulting solutions were diluted up to 100 µL with LFA buffer (Milenia GenLine HybriDetectRef:MGHD 1) in a Protein LoBind® Eppendorf before adding the LFA strip (Milenia GenLine HybriDetectRef:MGHD 1). All the LFA strips were imaged after 10 min.

The formation of the desired PNA-DNA invasion complex was also followed by gel analysis with 15% Native PAGE at 4°C and 20V/cm. The DNA was visualized by SYBR Gold Nucleic acid staining.

8.2. Protocols for amplification and detection of SARS-CoV-2 viral RNA of Omicron versus Delta variant

RPA amplification Omicron versus Delta variant

Sequence alignment of the sequences from the variants are the following ones, shown in **blue** the **nucleotides mutated** from the SARS-CoV-2 Wuhan-Hu-1 (NC_045512.2):

SARS-CoV-2 Wuhan-Hu-1 / NC_045512.2 (158mer – 22981-23138):

5’-TCAGGCCGGTAGCACACCTTGTAATGGTGTTGAAGGTTTTAATTGTTACTTTCCTTTACAATCAT-ATGGTTTCCAACCCACTAATGGTGTTGGTTACCAACCATACAGAGTAGTAGTACTTTCTTTTGAACTT CTACATGCACCAGCAACTGTTTGTG-3’

Delta (B.1.617.2) first detected in India sequence / EPI_ISL_1544014 (158mer – 22944-23101):

5’-TCAGGCCGGTAGCA**A**ACCTTGTAATGGTGTTGAAGGTTTTAATTGTTACTTTCCTTTACAATCAT-ATGGTTTCCAACCCACTAATGGTGTTGGTTACCAACCATACAGAGTAGTAGTACTTTCTTTTGAACTT CTACATGCACCAGCAACTGTTTGTG-3’

Omicron (B.1.1.529) / EPI_ISL_6841980 (158mer – 22981-23138):

5’-TCAGGCCGGTA**A**CA**A**ACCTTGTAATGGTGTTG**C**AGGTTTTAATTGTTACTTTCCTTTAC**G**ATCAT-AT**A**GTTTCC**G**ACCCACT**T**ATGGTGTTGGT**C**ACCAACCATACAGAGTAGTAGTACTTTCTTTTGAACTT CTACATGCACCAGCAACTGTTTGTG-3’

Following the general procedure of the RPA TwistAmp Basic kit (TwistDx: TABAS03KIT), RPA was carried out in a PCR tube in a final volume of 50 µL and 500 nM of primers. The amplification was initiated as follows: 30 µL of primer-free rehydration buffer were mixed with 9.5 µL of water, 2.5 µL of 10 µM Cy3 labeled forward primer (5’-Cy3- A**A**CA**A**ACCTTG-TAATGGTGTTG**C**AGGTTTTAAT-3’) and 2.5 µL of 10 µM reverse primer (5’- TGCTGGTGCATG-TAGAAGTTCAAAAGAAAGT-3’). This solution was transferred to a TwistAmp Basic reaction kit followed by adding 1 µL of analyte solution (158nb ssDNA template of either SARS-CoV-2 Wuhan-Hu-1 or SARS-CoV-2 Omicron variant (B.1.1.529) /EPI_ISL_6841980 at 4pM – 4.8e4 copies DNA/µL) or 1 µL water, and 2.5µL of $Mg(OAc)_2$ 280 mM. The reaction was incubated at 41°C for 30 min.

The formation of the desired DNA was followed by gel analysis with either 15% Native PAGE or 2.5% agarose gel. The DNA was visualized by SYBR Gold Nucleic acid staining.

The 158nb DNA templates used for RPA amplification correspond to:

・The nucleotides 22981-23138 of the SARS-CoV-2 genome (NC_045512.2) placed at the region (158nb ssDNA template, 5'- TCAGGCCGGTAGCACACCTTGTAATGGTGTTGAAGGTTT-TAATTGTTACTTTCCTTTACAATCATATGGTTTCCAACCCACTAATGGTGTTGGTTACCAACCATACAG AGTAGTAGTACTTTCTTTTGAACTTCTACATGCACCAGCAACTGTTTGTG -3'). The designed primer set amplifies the following 138nb DNA sequence (5'- AGCACACCTTGTAATGGTGTTGA-AGGTTTTAATTGTTACTTTCCTTTACAATCATATGGTTTCCAACCCACTAATGGTGTTGGTTACCAACC ATACAGAGTAGTAGTACTTTCTTTTGAACTTCTACATGCACCAGCA-3').

・The nucleotides 22981-23138 of the SARS-CoV-2 Omicron variant (B.1.1.529) / EPI_ISL_6841980 (158nb ssDNA template, 5'- TCAGGCCGGTA**A**CA**A**ACCTTGTAATGGTGTTG**C**-AGGTTTTAATTGTTACTTTCCTTTAC**G**ATCATAT**A**GTTTCC**G**ACCCACT**T**ATGGTGTTGGT**C**ACCAACC ATACAGAGTAGTAGTACTTTCTTTTGAACTTCTACATGCACCAGCAACTGTTTGTG-3').
The designed primer set amplifies the following 138nb DNA sequence (5'-A**A**CA**A**ACCTTGTAATGGTGTTG**C**AGGTTTTAATTGTTACTTTCCTTTAC**G**ATCATAT**A**GTTTCC**G**ACCC ACT**T**ATGGTGTTGGT**C**ACCAACCATACAGAGTAGTAGTACTTTCTTTTGAACTTCTACATGCACCAGC A -3').

・The nucleotides 22944-23101 of the SARS-CoV-2 Delta variant (B.1.617.2) first detected in India sequence / EPI_ISL_1544014 (5'-TCAGGCCGGTAGCA**A**ACCTTGTAATGGTGTTGAA-GGTTTTAATTGTTACTTTCCTTTACAATCATATGGTTTCCAACCCACTAATGGTGTTGGTTACCAACCA TACAGAGTAGTAGTACTTTCTTTTGAACTTCTACATGCACCAGCAACTGTTTGTG-3'). The designed primer set amplifies the following 138nb DNA sequence (5'- AGCA**A**ACCTTGTAATGGTGTTG-AAGGTTTTAATTGTTACTTTCCTTTACAATCATATGGTTTCCAACCCACTAATGGTGTTGGTTACCAAC CATACAGAGTAGTAGTACTTTCTTTTGAACTTCTACATGCACCAGCA -3').

For RT-RPA, the same procedure was used starting with 1 µL RNA analyte (Omicron variant (B.1.1.529)/ EPI_ISL_6841980 / "Synthetic control 23" - 5e3 copies/µL stock or Delta variant (B.1.617.2) first detected in India sequence / EPI_ISL_1544014 / "Synthetic control 48" - 5e3 copies/µL stock, obtained both synthetic control RNAs from TWIST Bioscience) or 1 µL water, with 1 µL of RevertAid Reverse Transcriptase (Thermo Fisher Scientific, Ref: EP0441), 1 µL of

Recombinant RNasin Ribonuclease Inhibitor (Promega, Ref: N251A) and the RT-RPA reaction was carried out for a total of 30 min at 41°C.

Invasion and detection of the viral RNA sequence Omicron versus Delta variant

For the invasion experiments coupled with LFA read out, 20 µL of the RT-RPA mixture (500nM primers/dsDNA amplicon) after 30 min at 41°C was transferred into a Protein LoBind® Eppendorf containing 130 µL of the PNA invasion MIX (115 nM of each indicated PNA, 46 ng/µL 20-*N* ssDNA scavenger, 1.15x PS buffer, 9.23 mM additional $MgSO_4$). The resulting 150µL mixture [66.7 nM primers/dsDNA amplicon (1 eq), 100 nM each PNA (1.5 eq), 40 ng/µL 20-*N* ssDNA scavenger, 1x PS buffer, 9 mM additional $MgSO_4$] was heated at 95°C for 5 min. The RPA invasion mixture was then centrifuged for 5 min at 14k rpm. Ratio of PNA/DNA calculated assuming complete amplification of the primers.

Then, 22.5 µL of the supernatant were diluted up to 100 µL with LFA buffer (Milenia GenLine HybriDetectRef:MGHD 1) in a Protein LoBind® Eppendorf before adding the LFA strip (Milenia GenLine HybriDetectRef:MGHD 1). All the LFA strips were imaged after 10 min.

The formation of the desired PNA-DNA invasion complex was also followed by gel analysis with 15% Native PAGE at 4°C and 20V/cm. The DNA was visualized by SYBR Gold Nucleic acid staining.

Single-Strand Conformation Polymorphism (SSCP) analysis of RPA amplicons from SARS-CoV-2 Wuhan-Hu-1(NC_045512.2) and Omicron (B.1.1.529)/EPI_ISL_6841980

The RPA amplification protocol above described for SARS-CoV-2 Wuhan-Hu-1(NC_045512.2) and Omicron (B.1.1.529)/EPI_ISL_6841980, having as a template the purified 158-mer ssDNAs, was followed. After amplification 30 min at 41°C, the samples were heated 5 min at 95°C. Then, the amplification products purified by QIAquick PCR Purification Kit (QIAGEN, ref: 28104). The purified 138nb dsDNA amplicons were eluted in water. The concentration of the eluted amplicons was measured and, 180 nM solutions of the amplicons in 1:1 water/formamide were annealed at 95°C for 5 min and snap cooled at 0°C for 10 min before gel analysis with 12% Native PAGE including 10% glycerol at 4°C, 20V/cm. The DNA was visualized by SYBR Gold Nucleic acid staining.

9. **Raw copies of acrylamide and agarose gels**

Raw native-PAGE Figure 3B

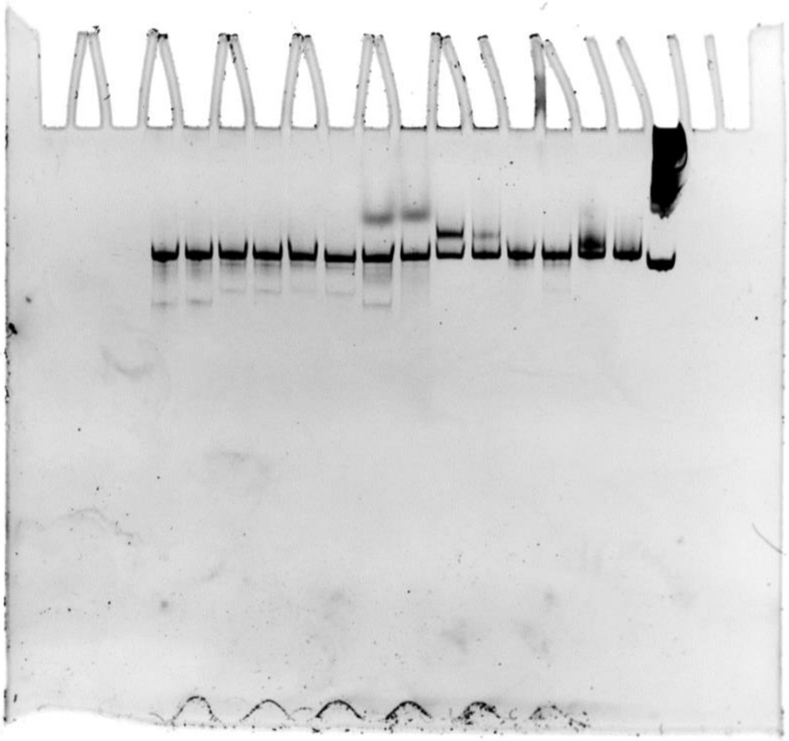

Raw native-PAGE Figure 3C

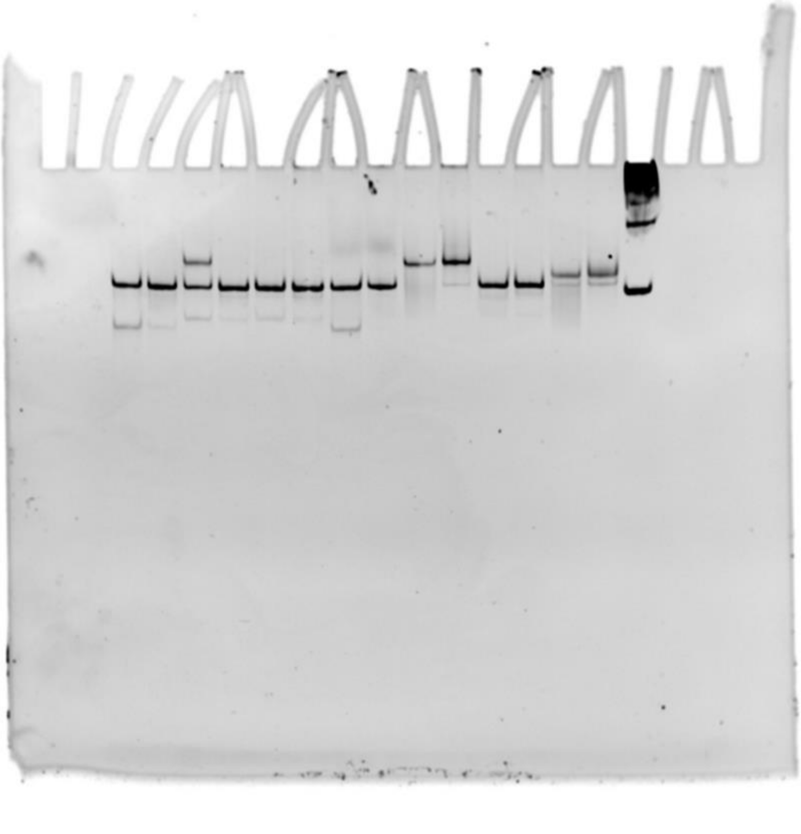

Raw native-PAGE Figure 4C

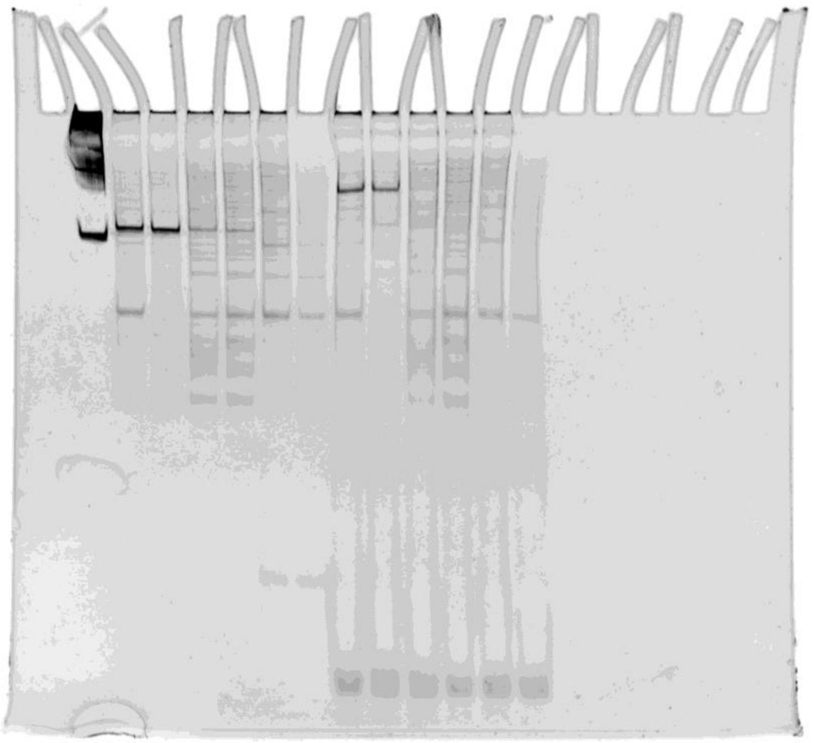

Raw native-PAGE Figure 5C

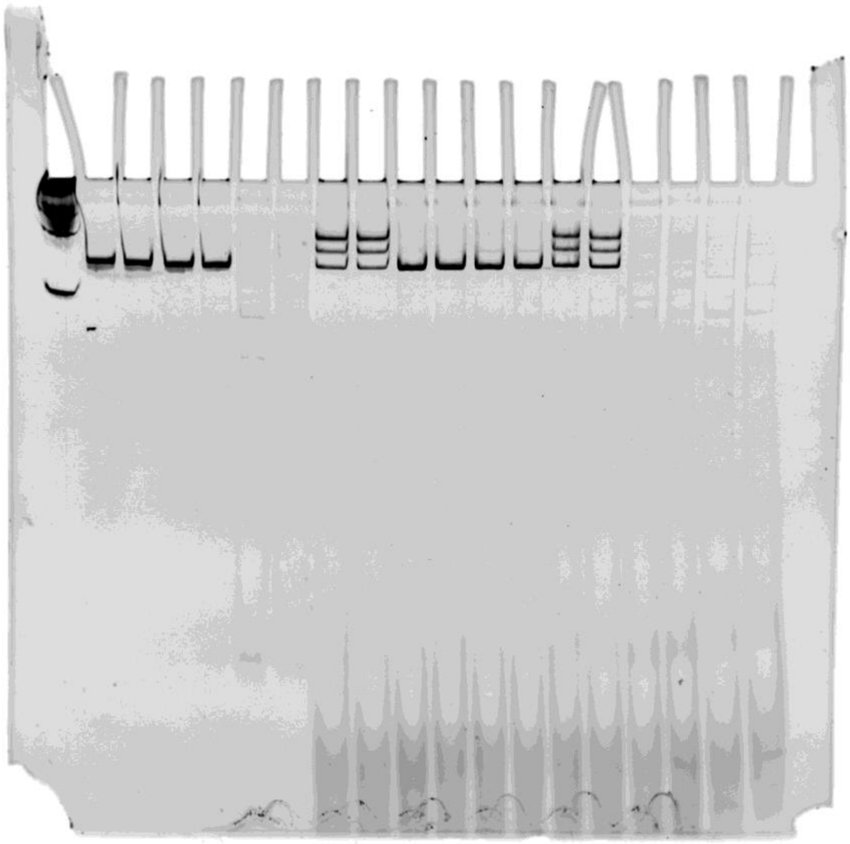

Raw native-PAGE Figure S3A

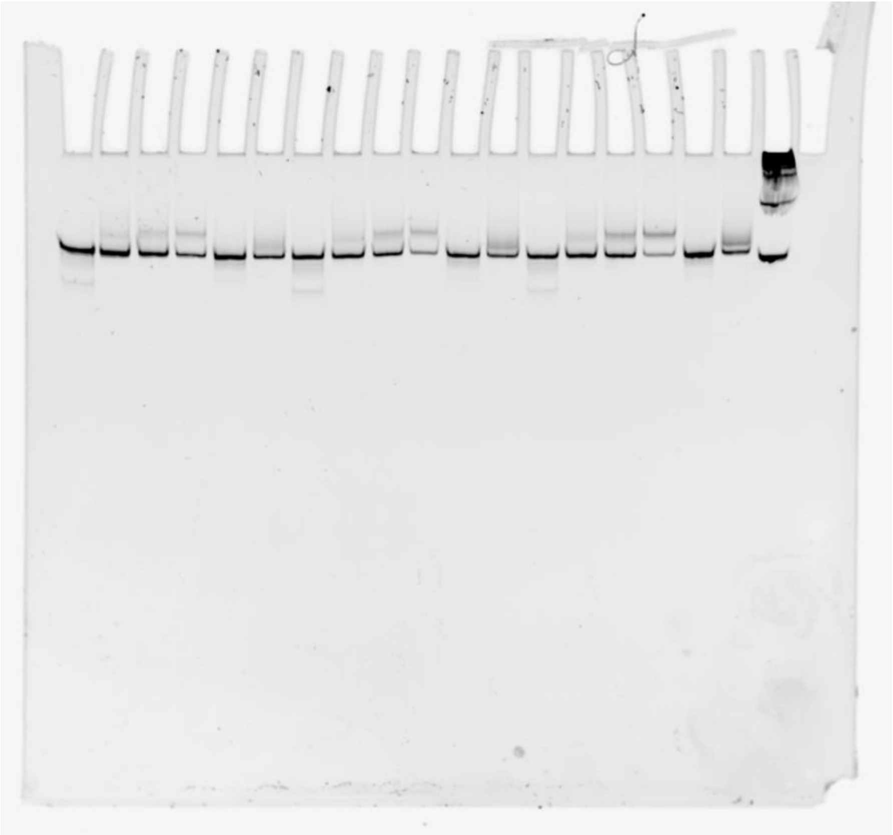

Raw native-PAGE Figure S3B

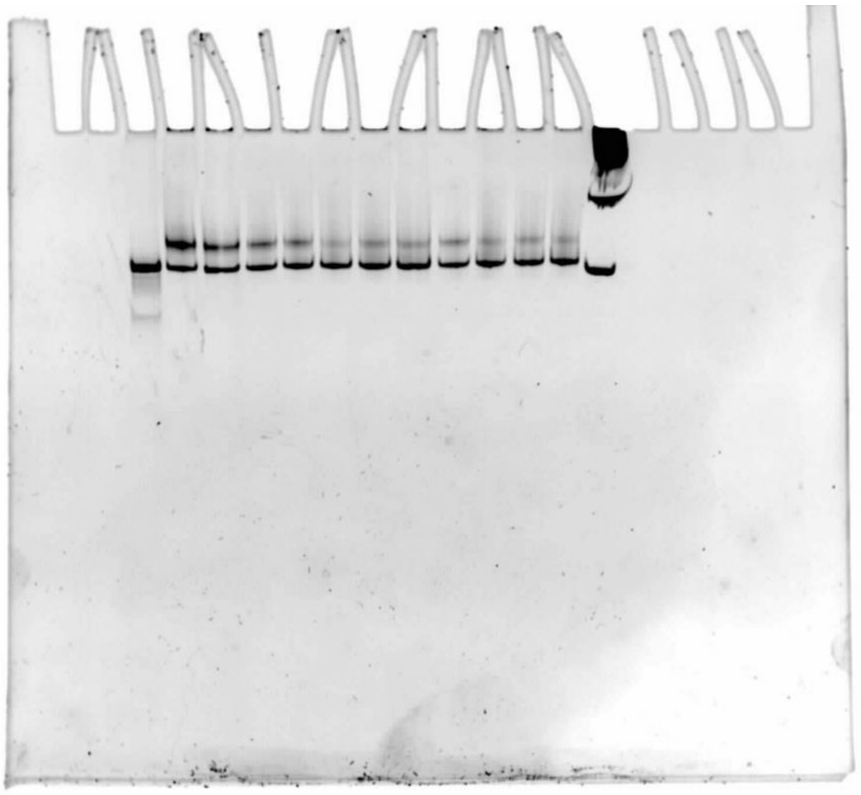

Raw native-PAGE Figure S5

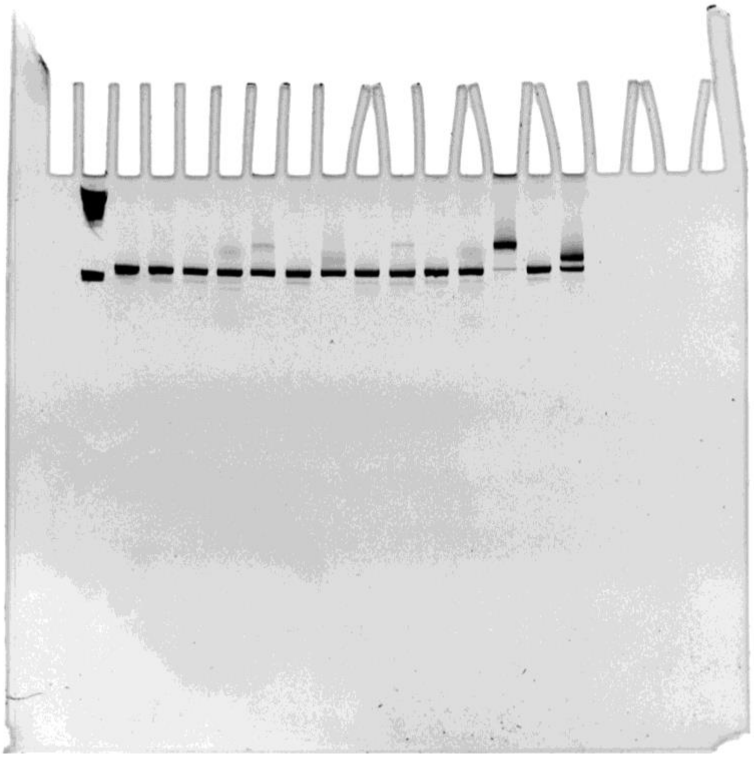

Raw native-PAGE Figure S6A

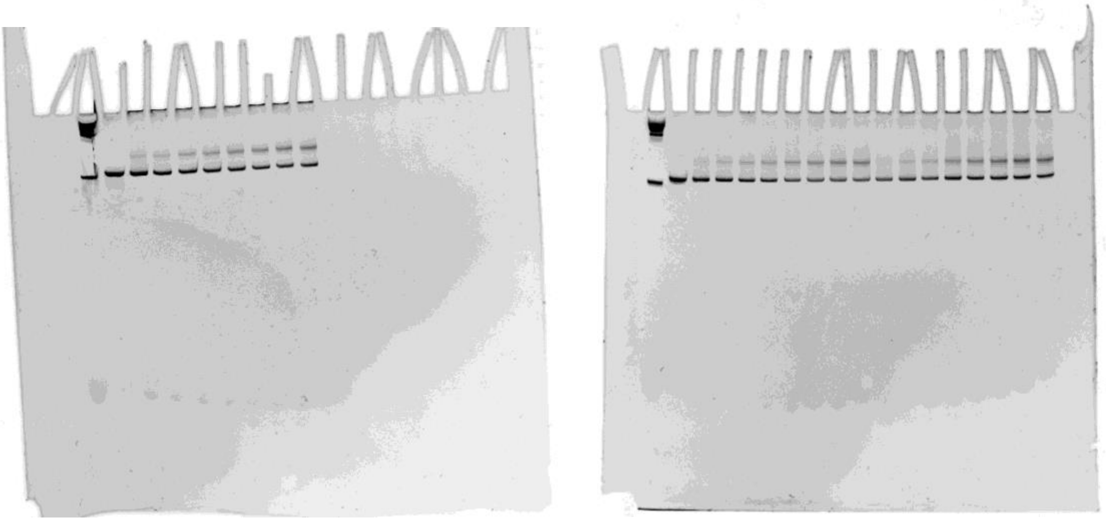

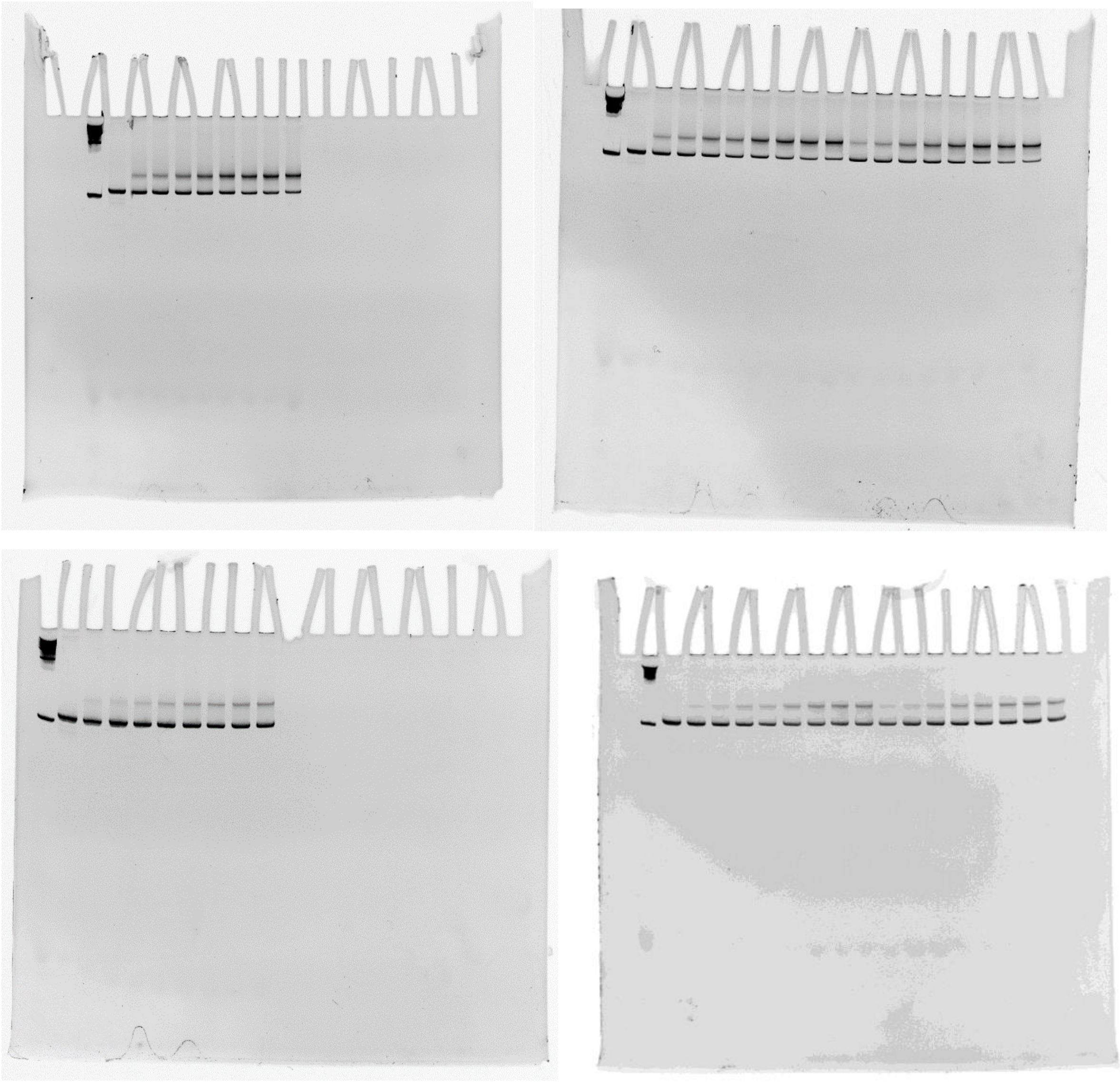

Raw native-PAGE Figure S6B

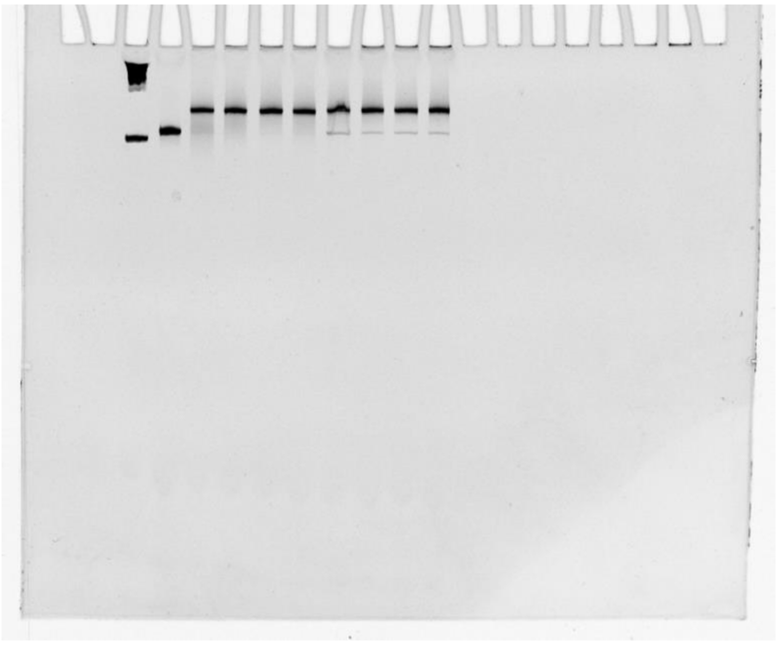

Raw native-PAGE Figure S7B

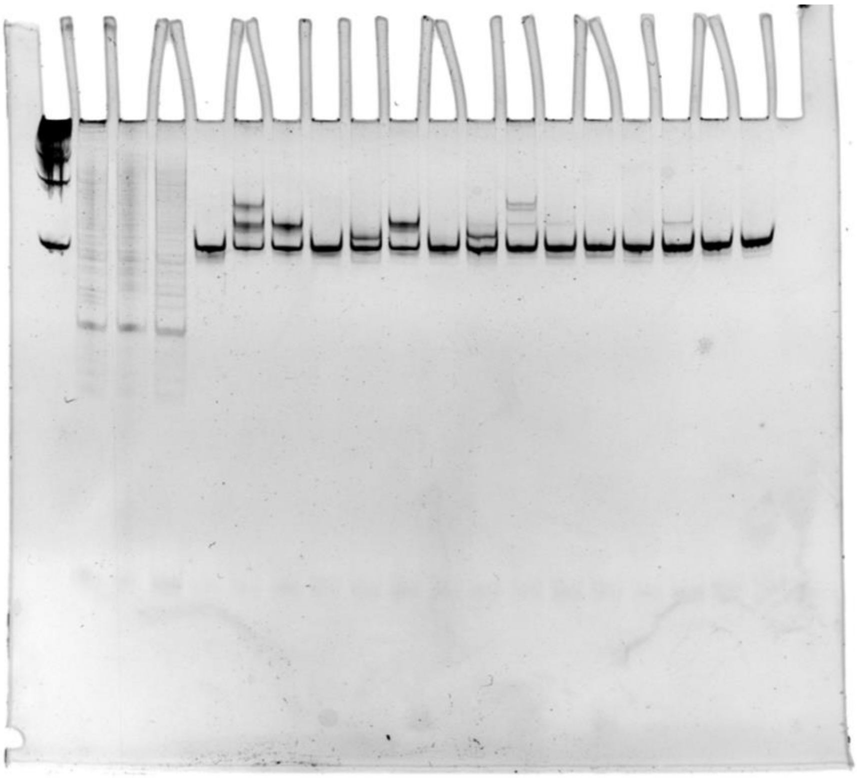

Raw native-PAGE Figure S8B

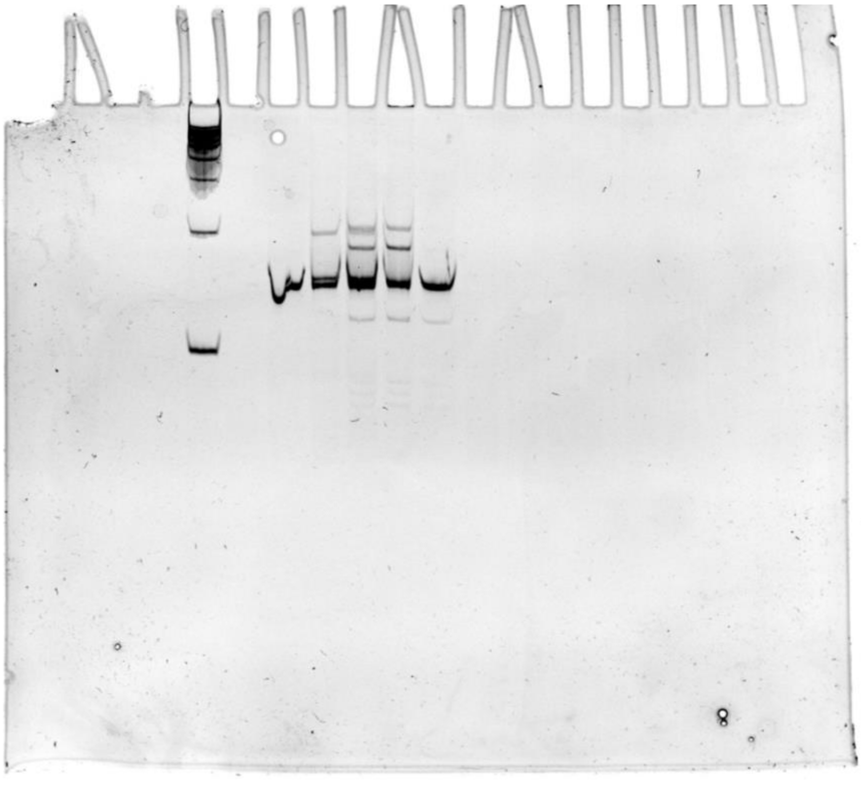

Raw native-PAGE Figure S9A

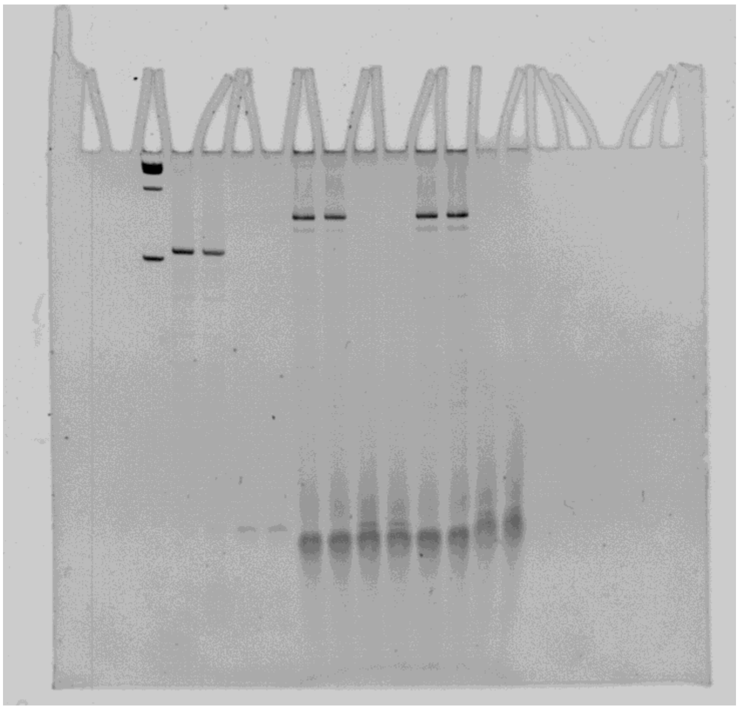

10.Raw copies of the LFA strips

Raw LFA strips Figure 4B

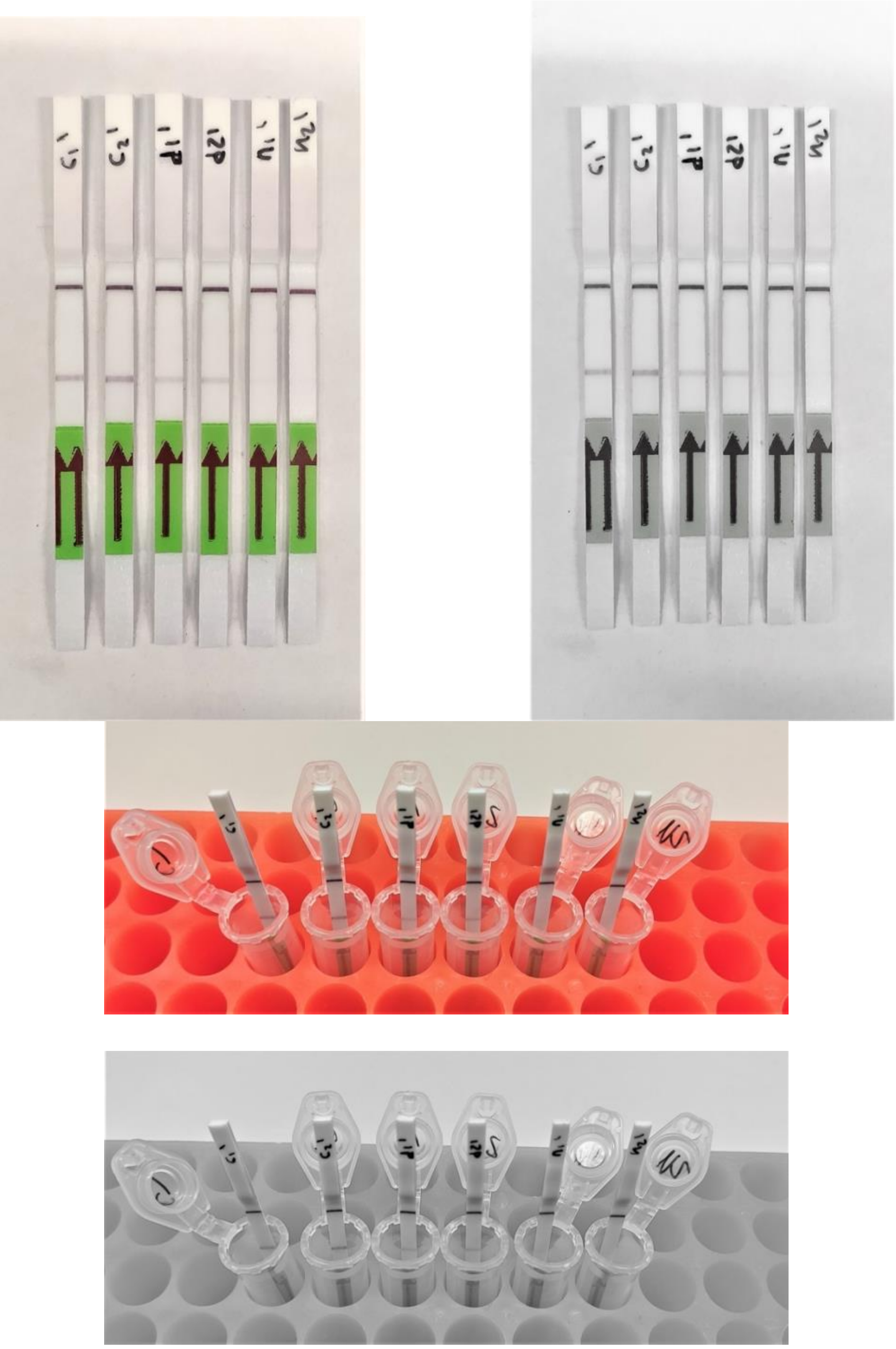

Raw LFA strips Figure 5A

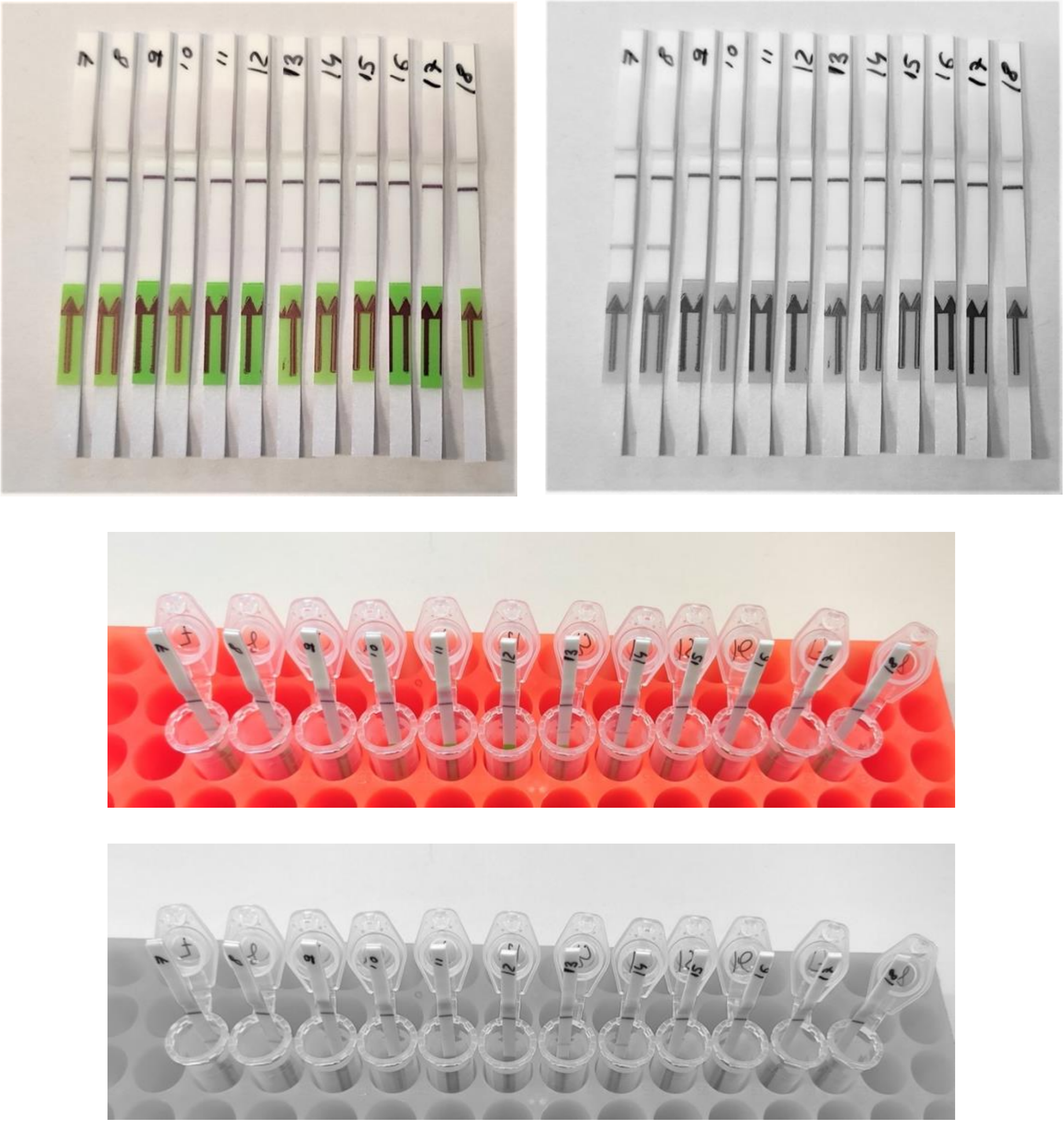

Raw LFA strips Figure S9B

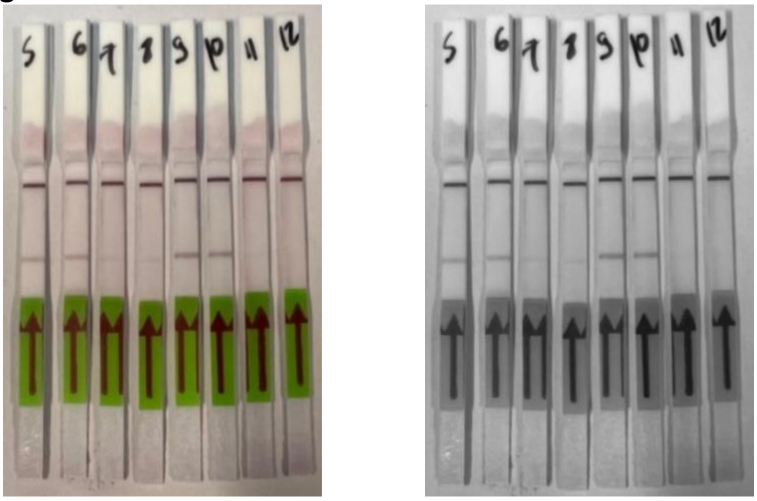

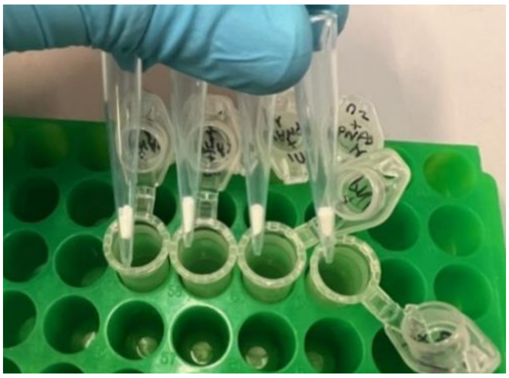

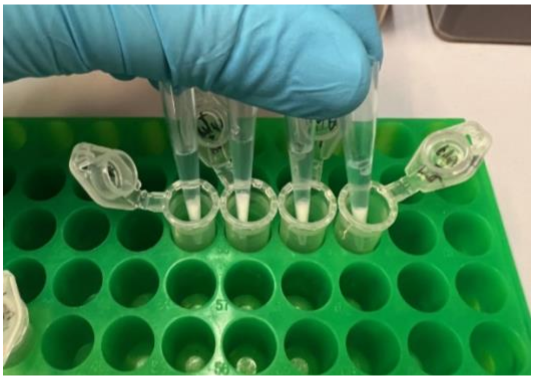

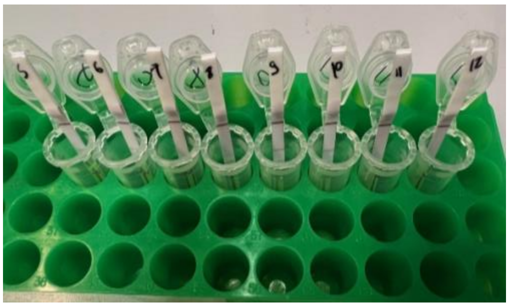

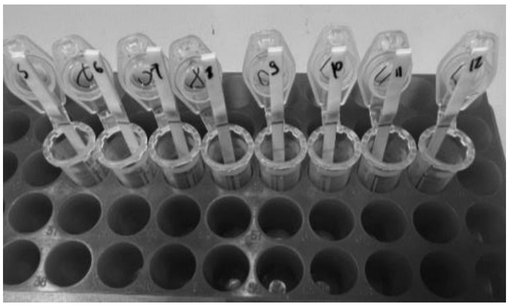

11. Copies of the ^{1}H and ^{13}C NMR spectra for compounds *1, 4, 7, 8, 9, 11, 13, 16, 21*

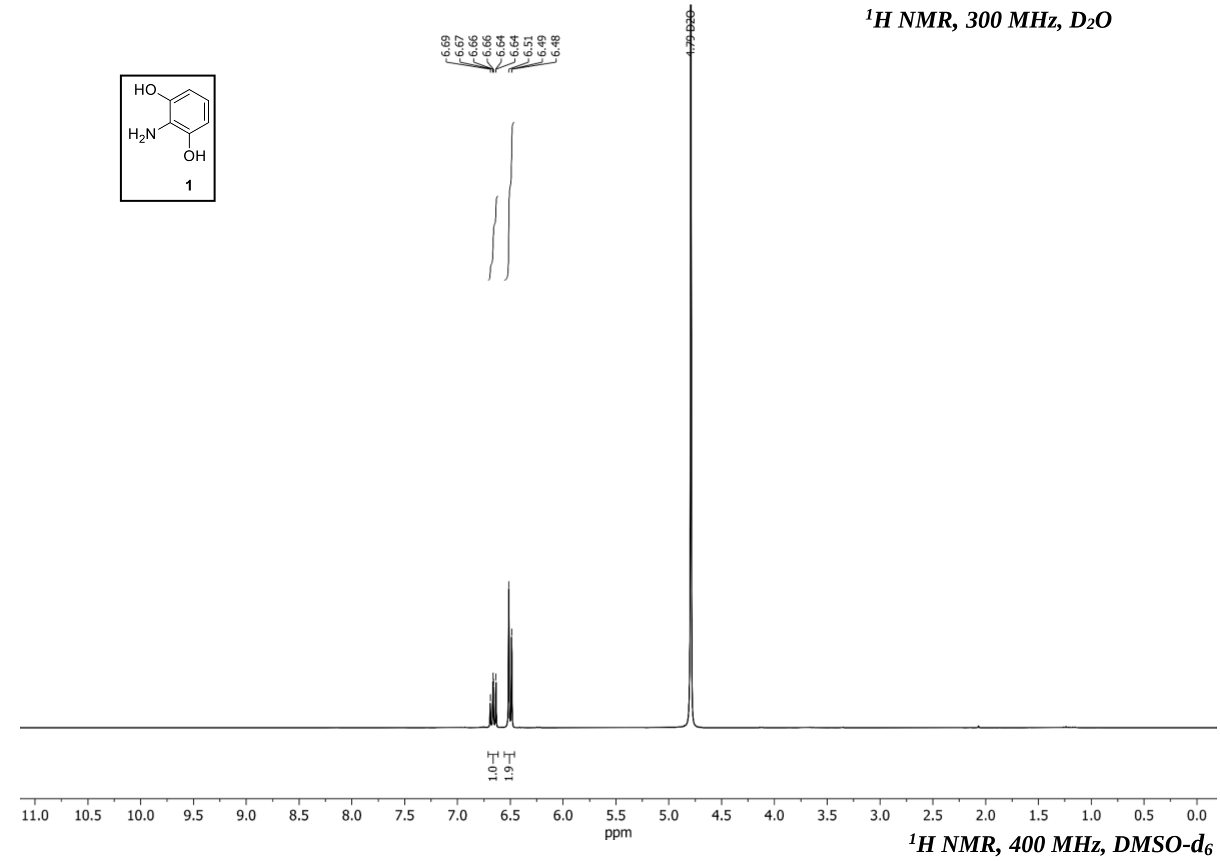

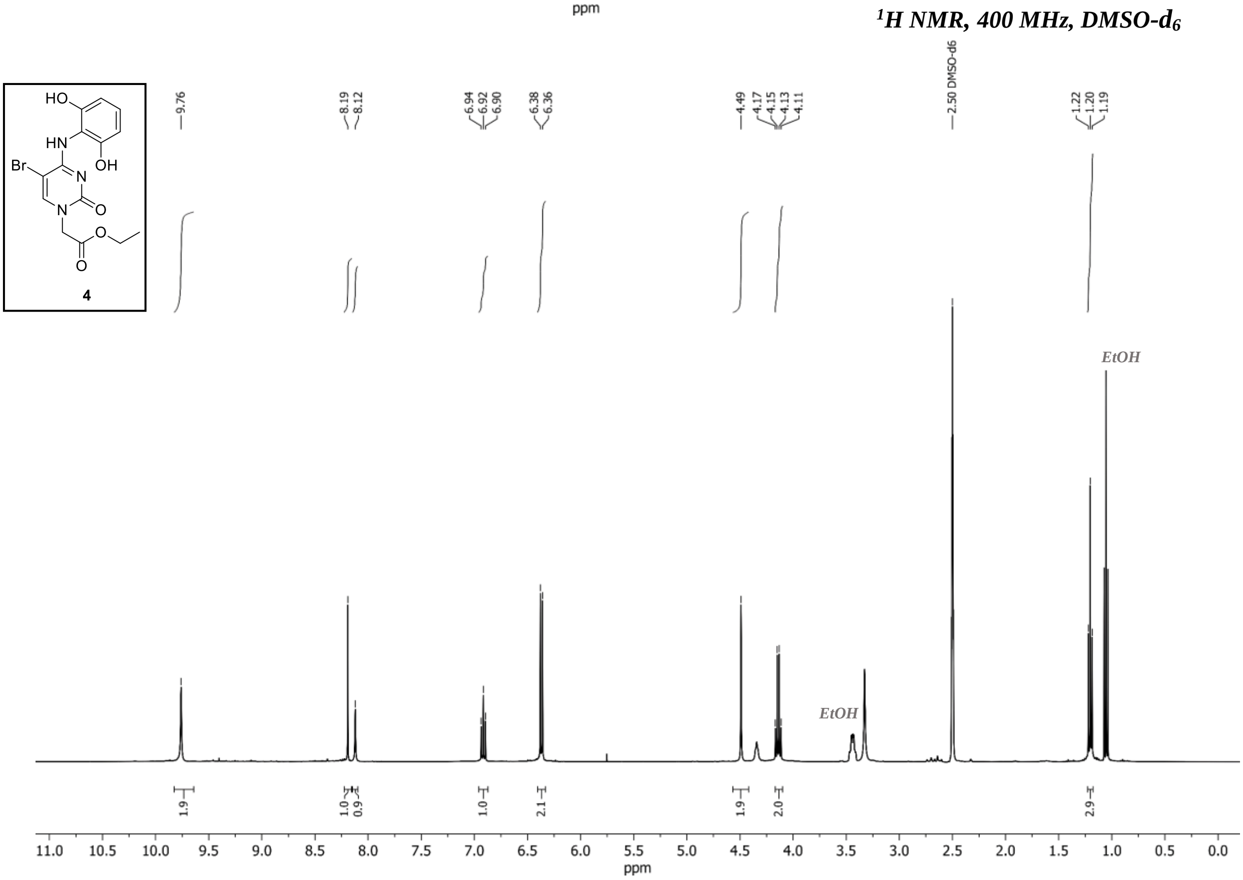

^{1}H NMR, 400 MHz, DMSO-d_6

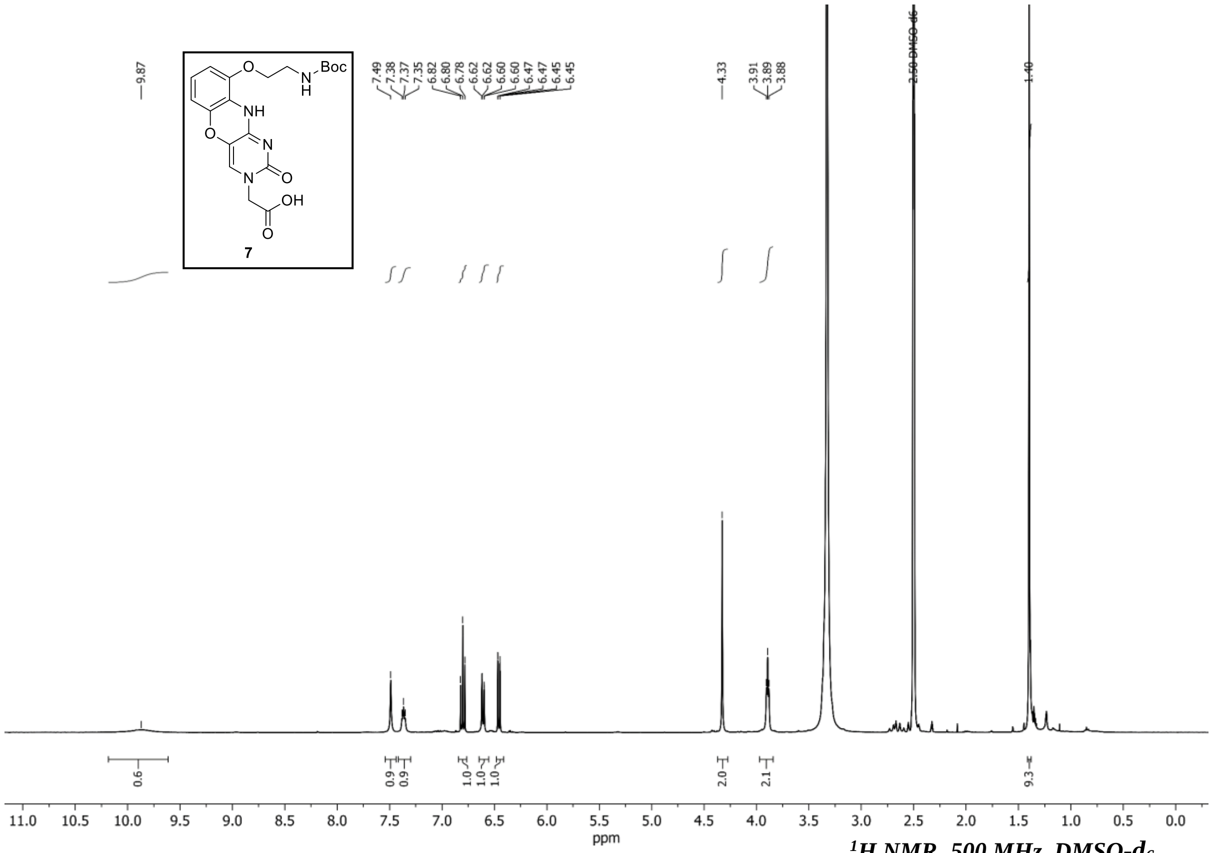

^{1}H NMR, 500 MHz, DMSO-d_6

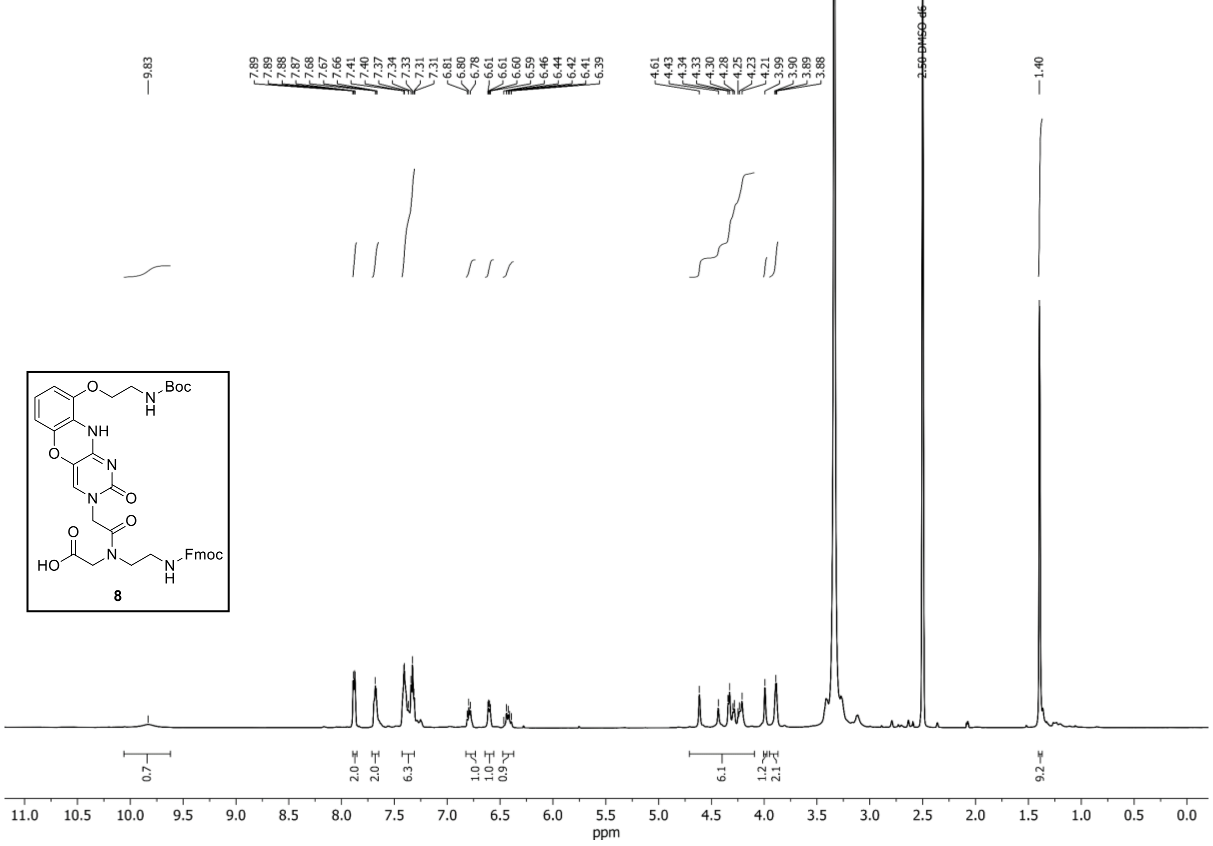

^{13}C NMR, 500 MHz, DMSO-d_6

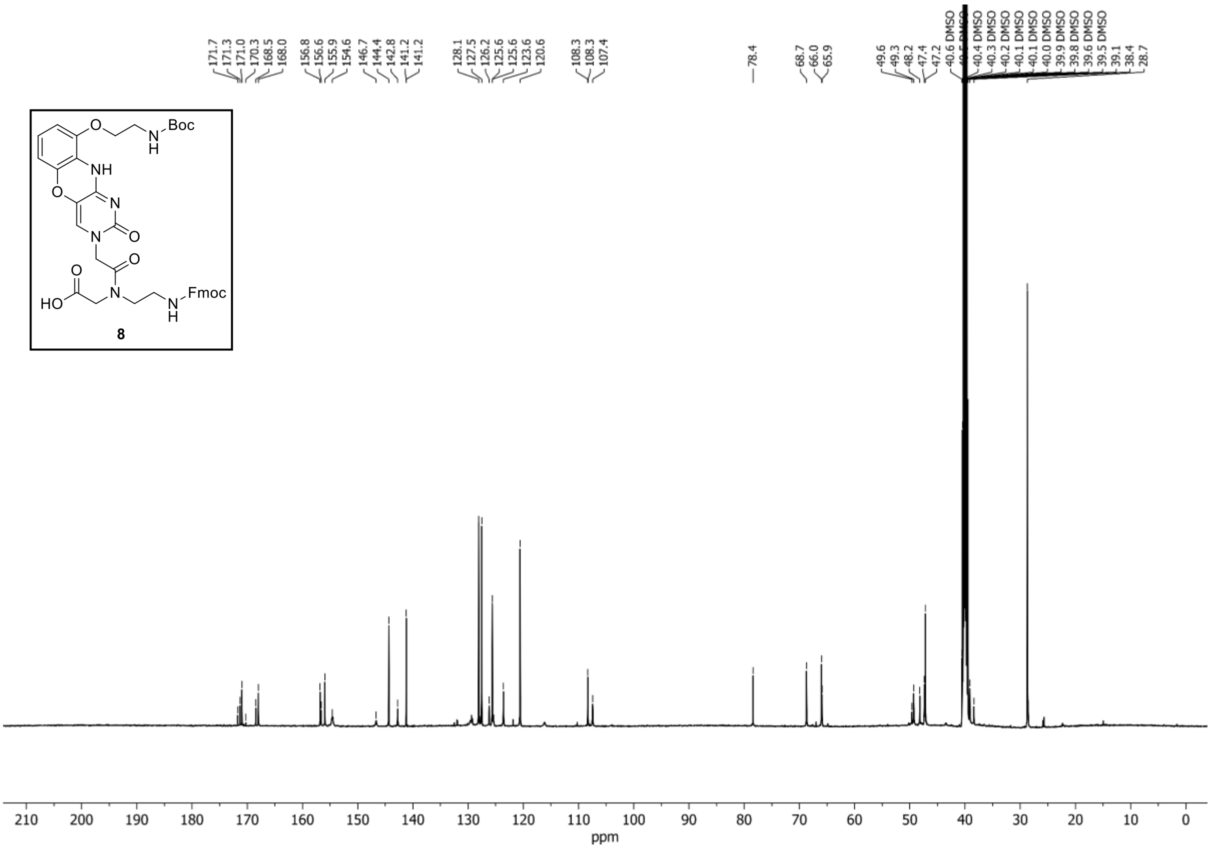

^{1}H NMR, 400 MHz, DMSO-d_6

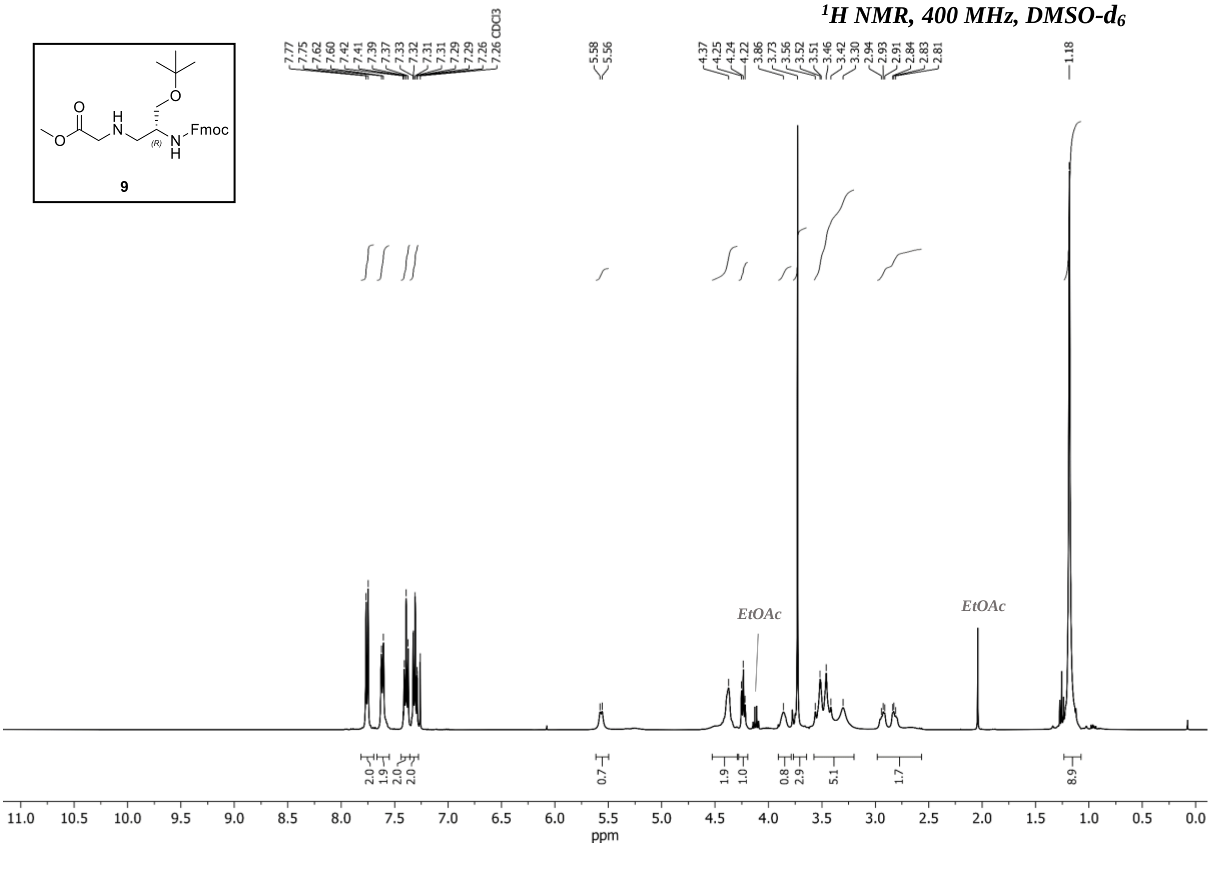

^{13}C NMR, 400 MHz, DMSO-d_6

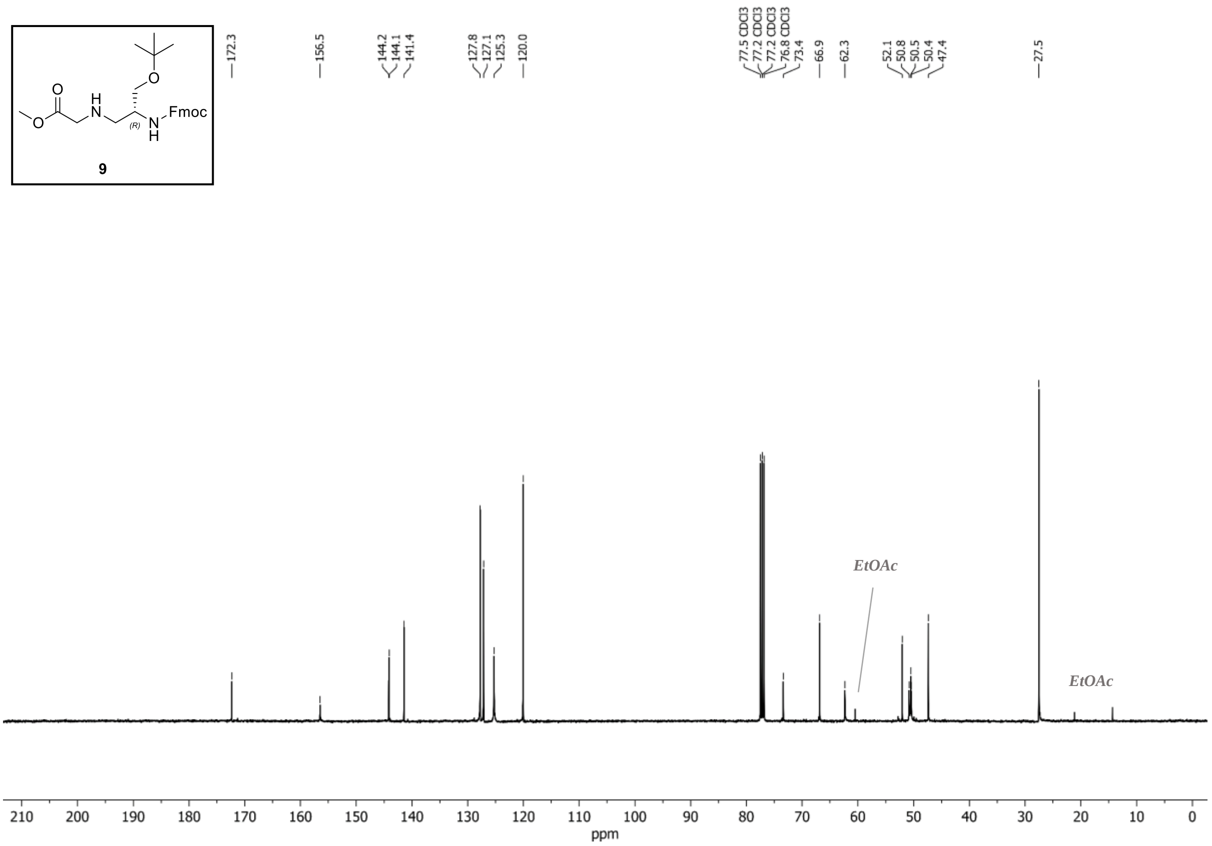

^{1}H NMR, 500 MHz, DMSO-d_6

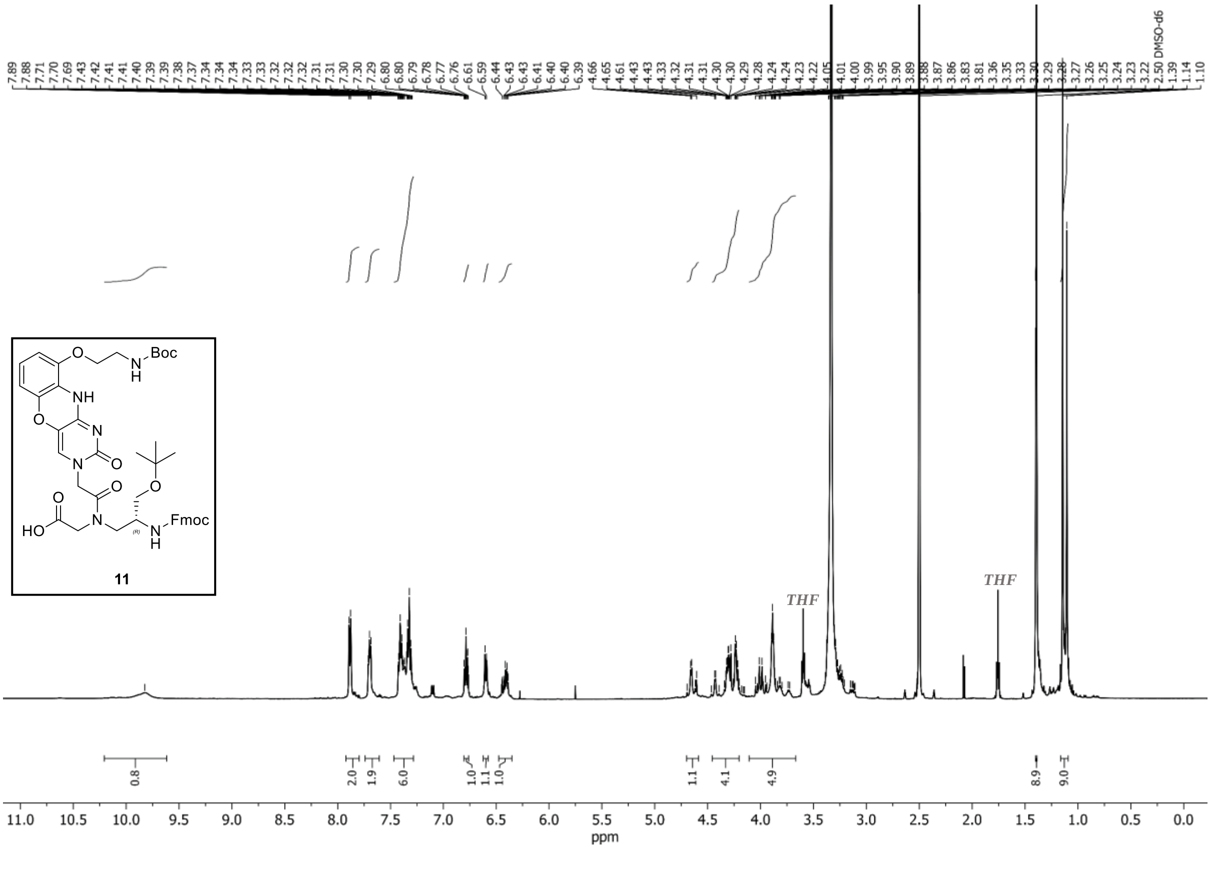

^{13}C NMR, 500 MHz, DMSO-d_6

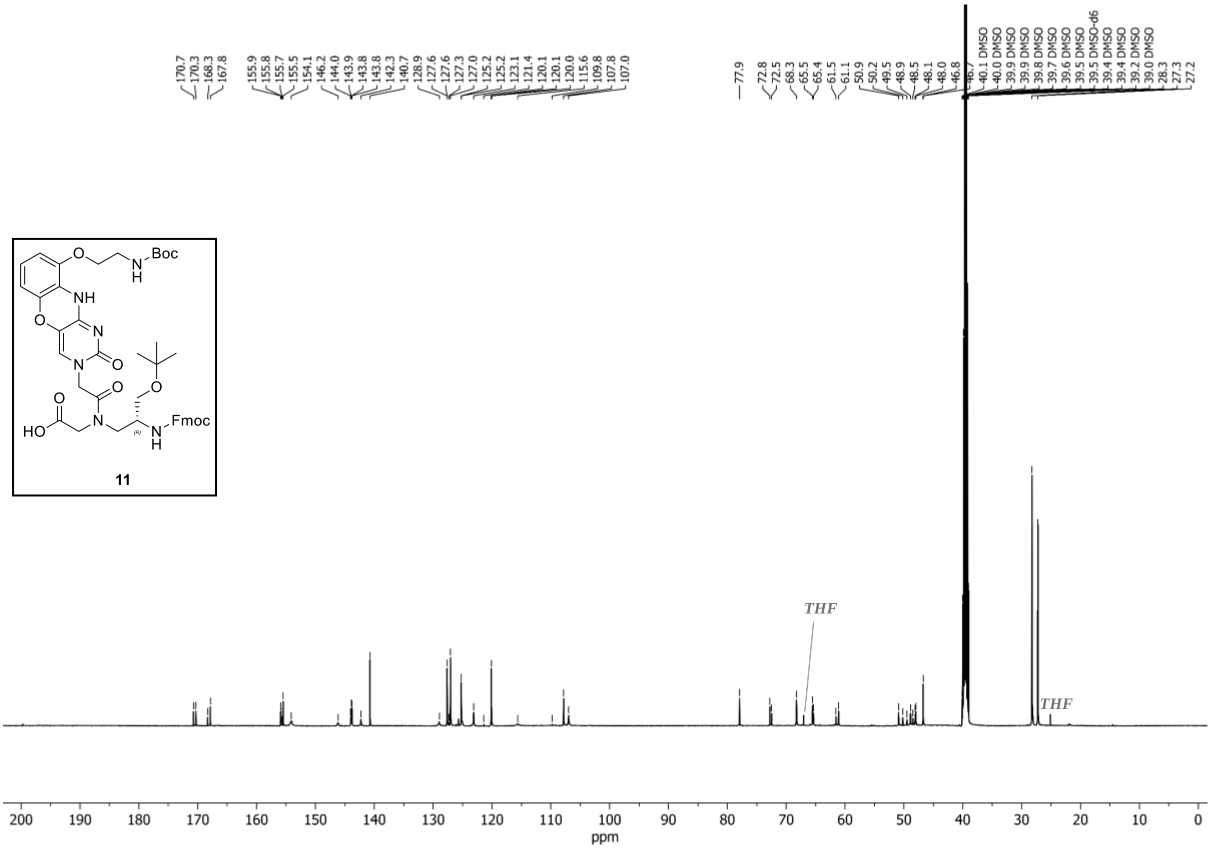

^{1}H NMR, 500 MHz, DMSO-d_6

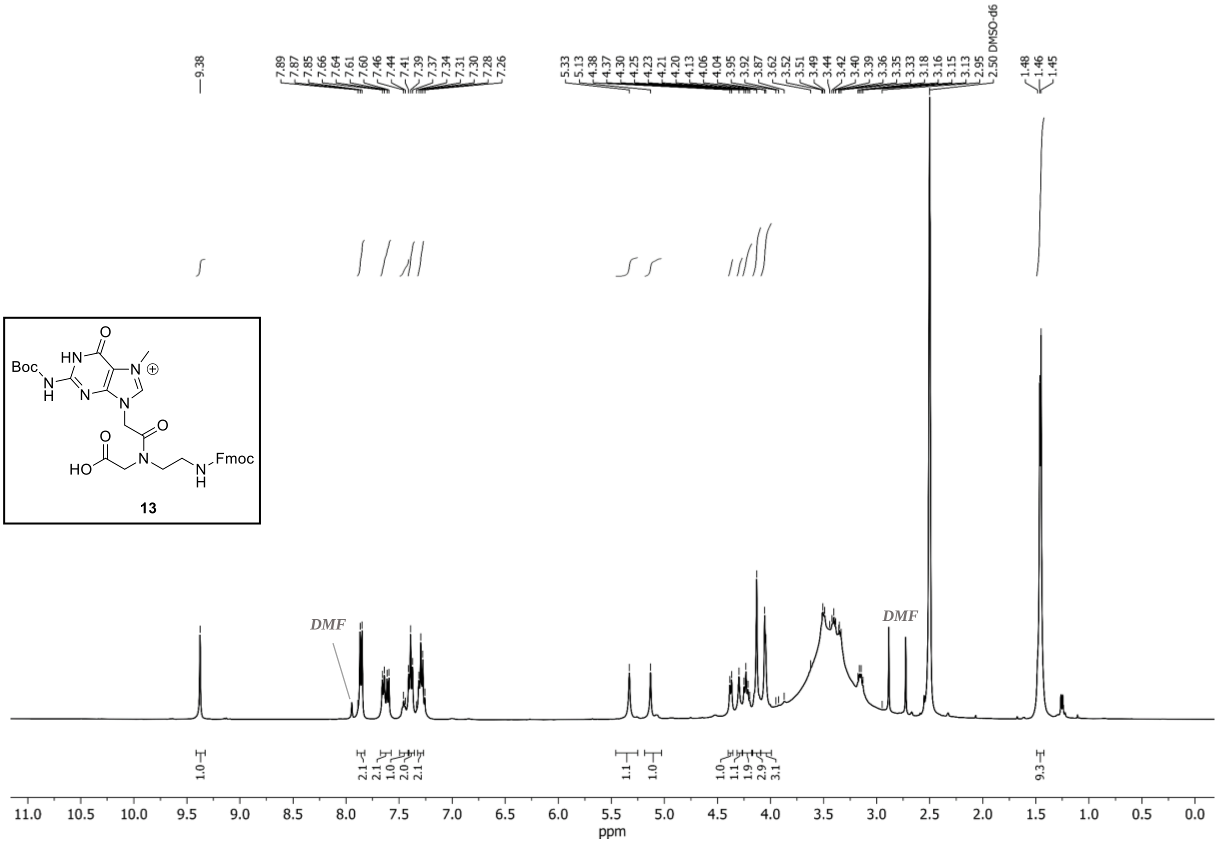

^{13}C NMR, 500 MHz, DMSO-d_6

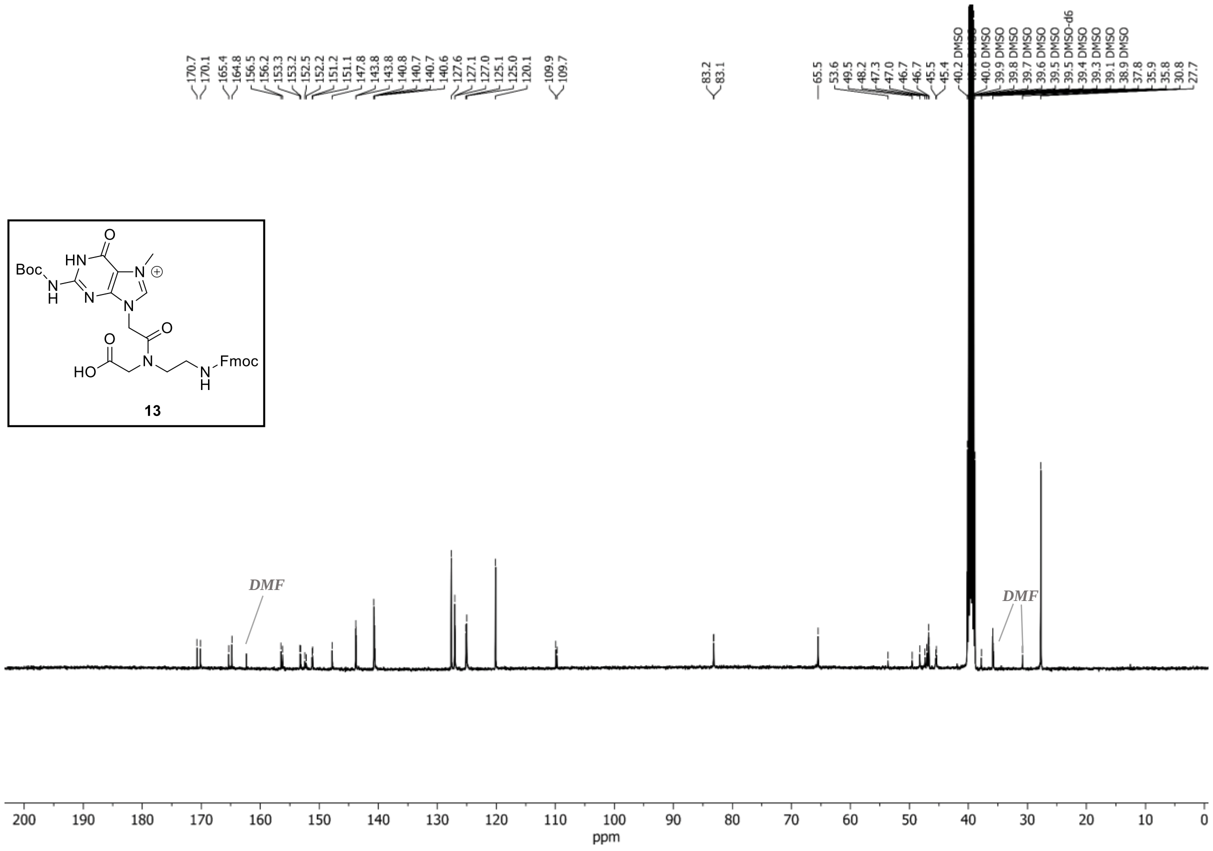

^{1}H NMR, 500 MHz, DMSO-d_6

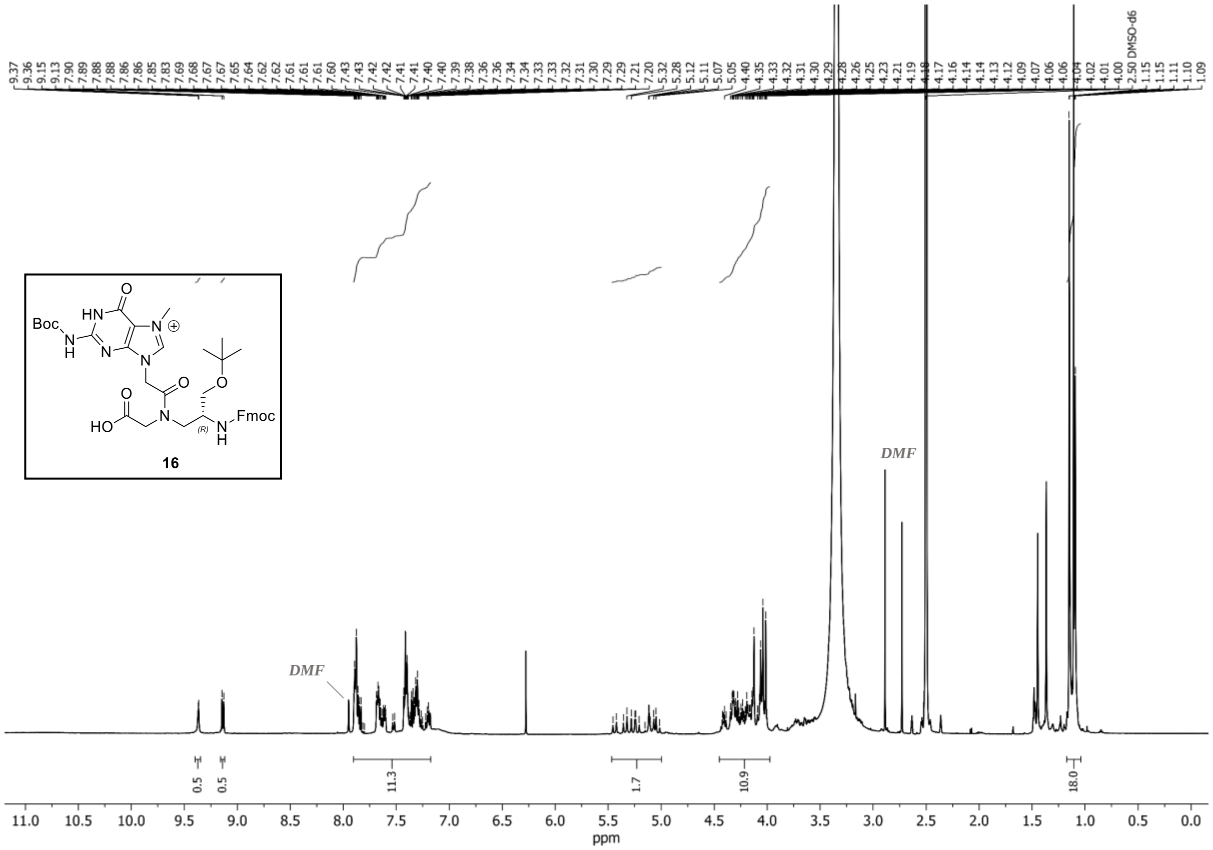

^{13}C NMR, 500 MHz, DMSO-d_6

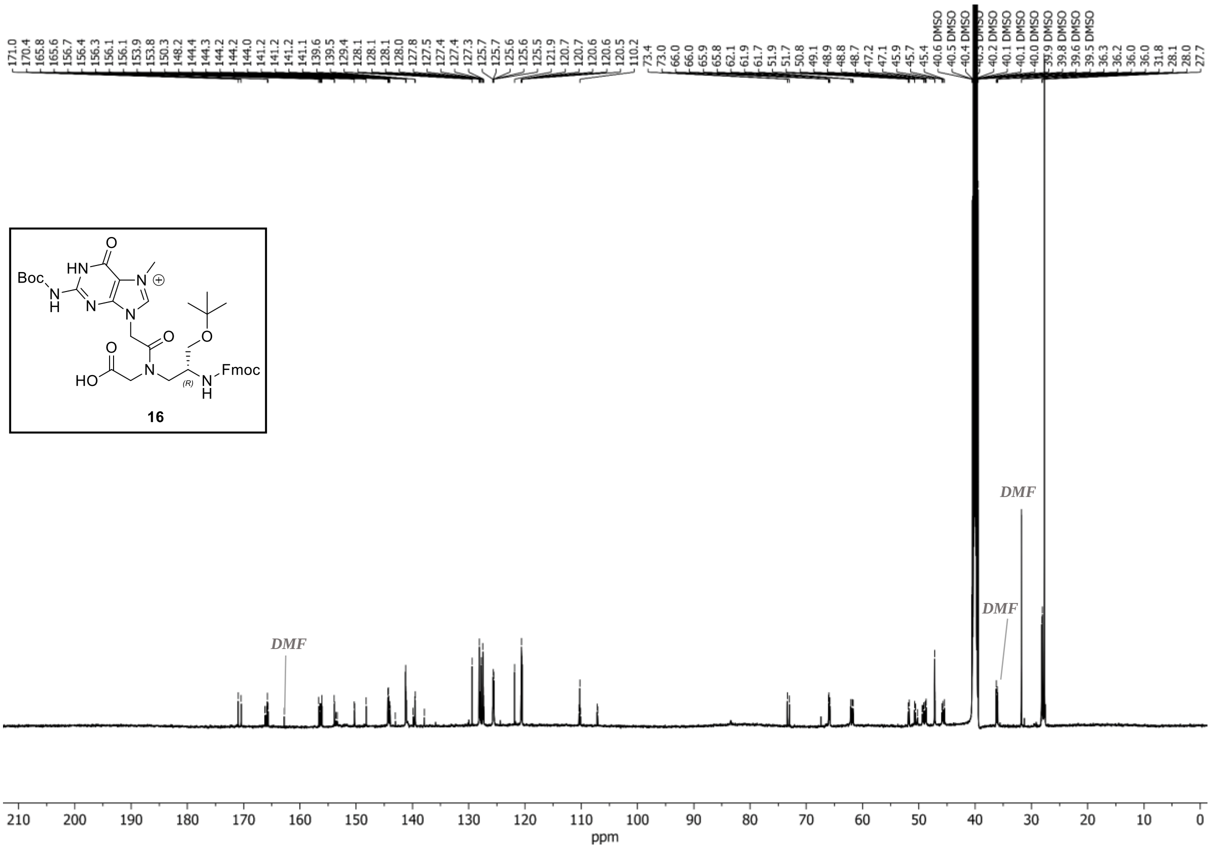

^{1}H NMR, 400 MHz, DMSO-d_6

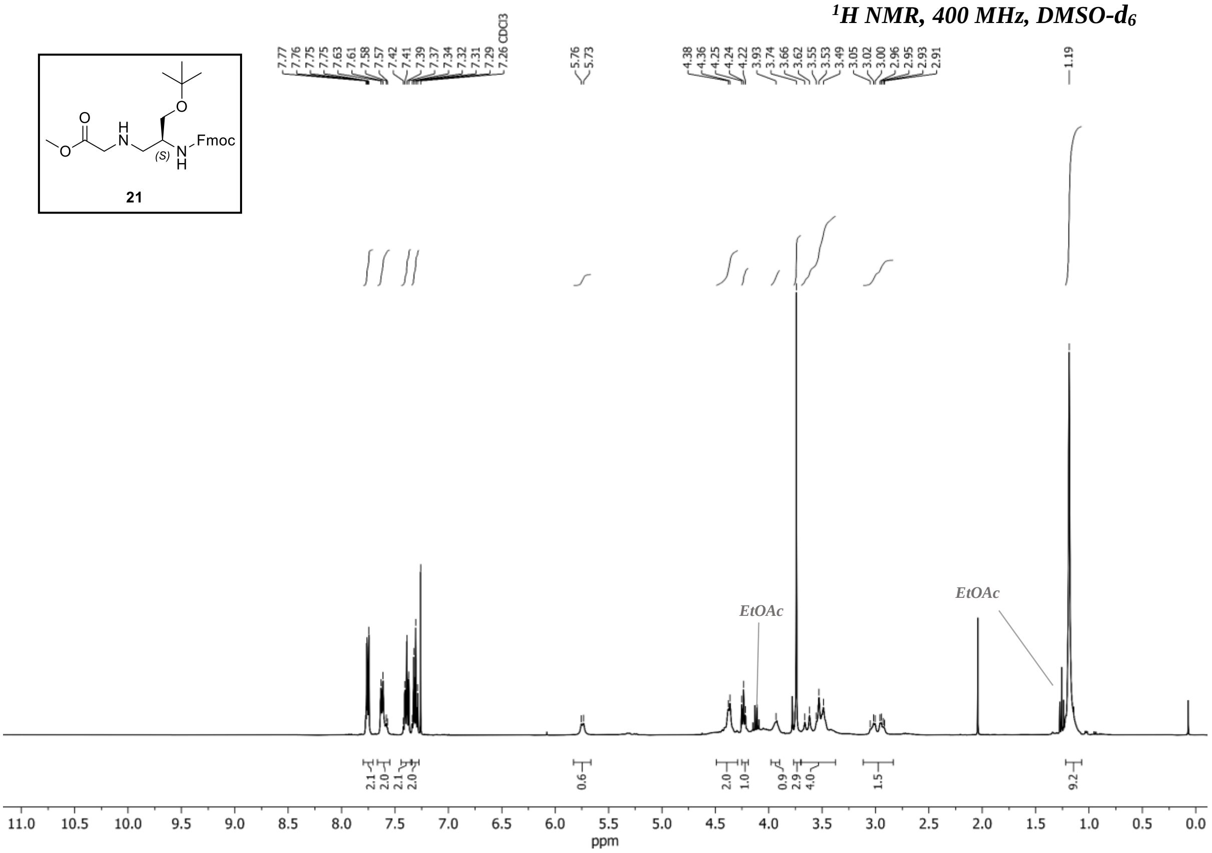

^{13}C NMR, 400 MHz, DMSO-d_6

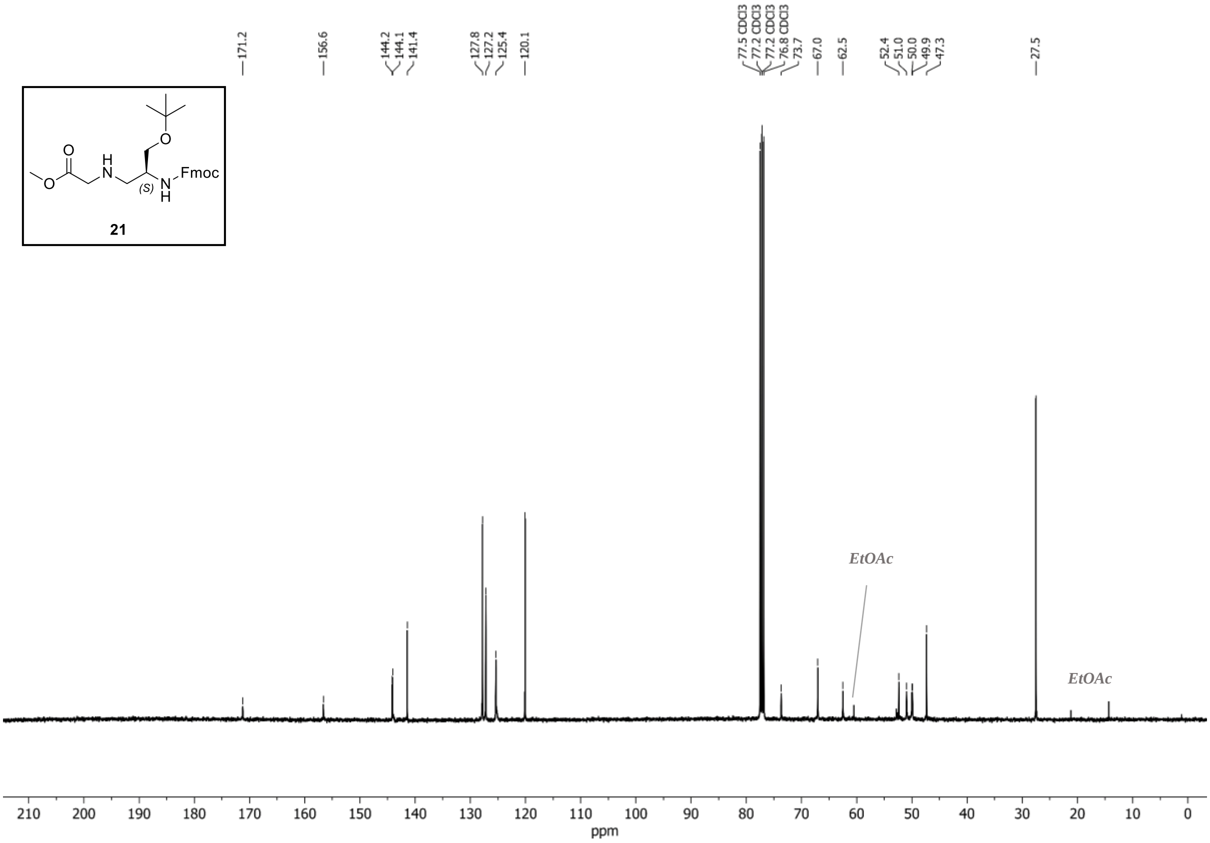

12. Copies of the LC-MS traces for compounds *1*, *4*, *7*, *8*, *9*, *11*, *13*, *16*, *21*

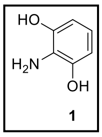

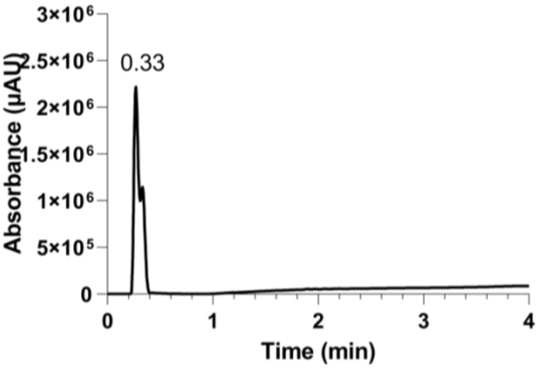

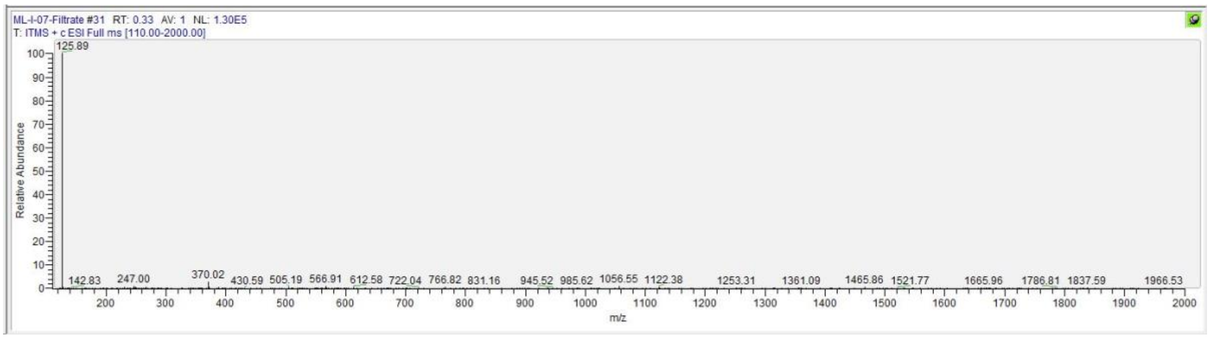

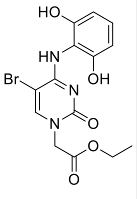
HO
HN
Br
OH
N
O
N
O
O

4

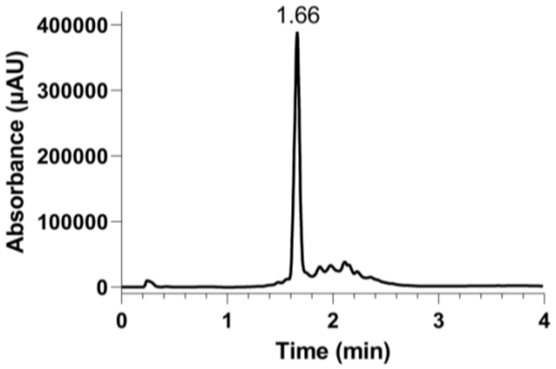
1.66
Absorbance (µAU)
400000
300000
200000
100000
0
0
1
2
3
4
Time (min)

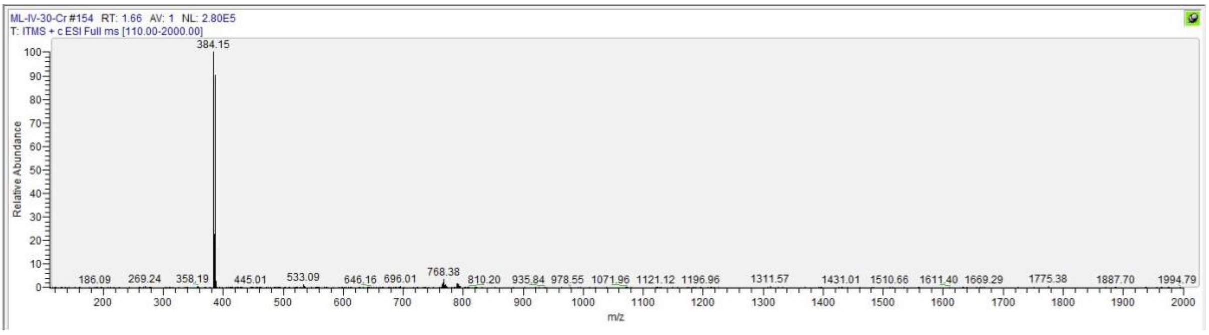
ML-IV-30-Cr #154 RT: 1.66 AV: 1 NL: 2.80E5
T: ITMS + c ESI Full ms [110.00-2000.00]
384.15
186.09 269.24 358.19 445.01 533.09 646.16 696.01 768.38 810.20 935.84 978.55 1071.96 1121.12 1196.96 1311.57 1431.01 1510.66 1611.40 1669.29 1775.38 1887.70 1994.79
Relative Abundance
m/z

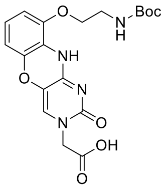
O
N
Boc
H
NH
O
N
N
O
OH
O

7

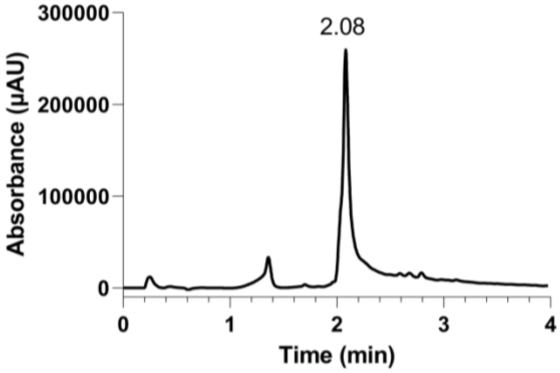
2.08
Absorbance (µAU)
300000
200000
100000
0
0
1
2
3
4
Time (min)

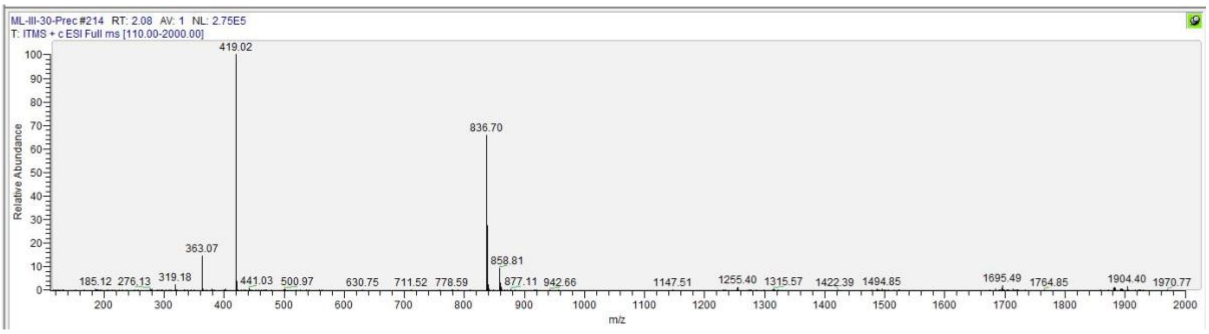
ML-III-30-Prec #214 RT: 2.08 AV: 1 NL: 2.75E5
T: ITMS + c ESI Full ms [110.00-2000.00]
419.02
836.70
363.07
858.81
185.12 276.13 319.18 441.03 500.97 630.75 711.52 778.59 877.11 942.66 1147.51 1255.40 1315.57 1422.39 1494.85 1695.49 1764.85 1904.40 1970.77
Relative Abundance
m/z

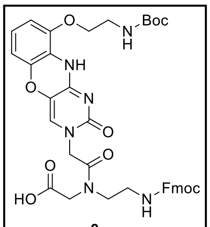
Boc
H
NH
O
N
O
N
O
O
HO
N
Fmoc
H

8

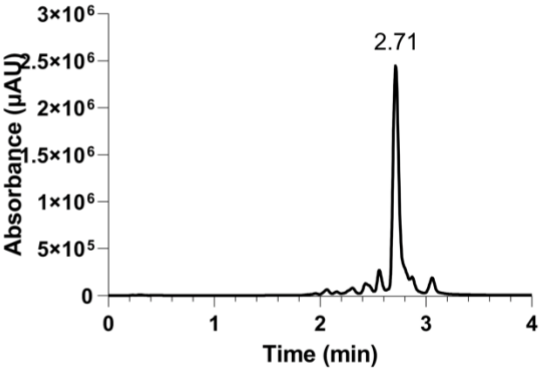
2.71
Absorbance (μAU)
Time (min)

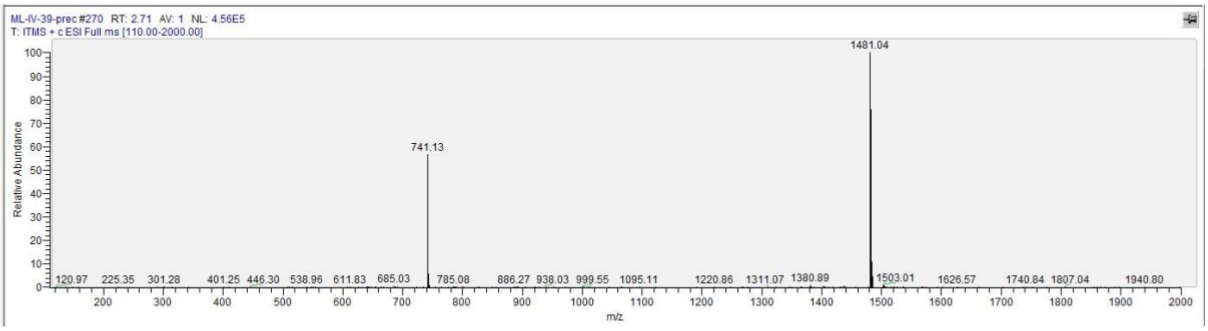
ML-IV-39-prec #270 RT: 2.71 AV: 1 NL: 4.56E5
T: ITMS + c ESI Full ms [110.00-2000.00]
Relative Abundance
m/z
1481.04
741.13
120.97 225.35 301.28 401.25 446.30 538.96 611.83 685.03 785.08 886.27 938.03 999.55 1095.11 1220.86 1311.07 1380.89 1503.01 1626.57 1740.84 1807.04 1940.80

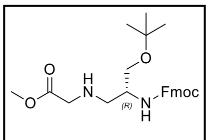
O
O
H
N
(R)
N
Fmoc
H
O

9

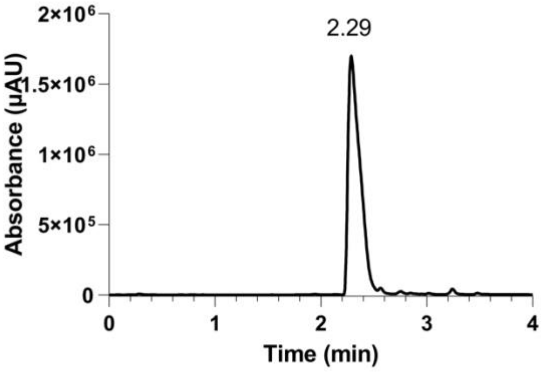
2.29
Absorbance (μAU)
Time (min)

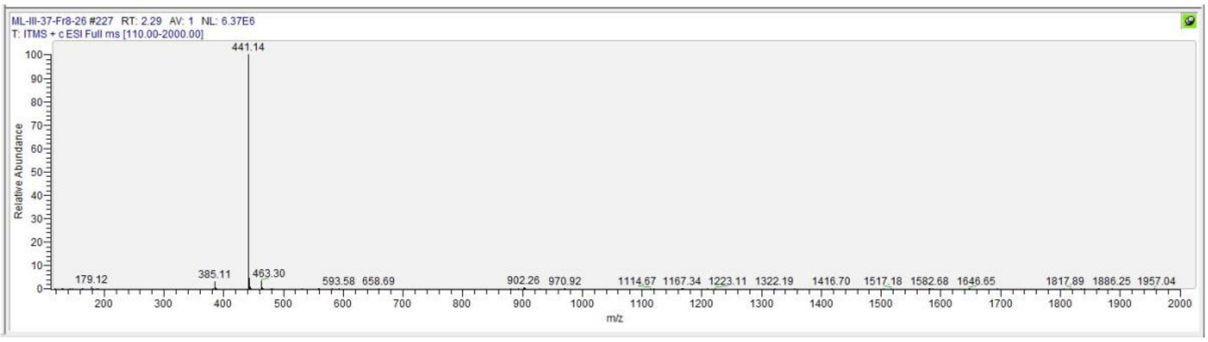
ML-III-37-Fr8-26 #227 RT: 2.29 AV: 1 NL: 6.37E6
T: ITMS + c ESI Full ms [110.00-2000.00]
Relative Abundance
m/z
441.14
179.12 385.11 463.30 593.58 658.69 902.26 970.92 1114.67 1167.34 1223.11 1322.19 1416.70 1517.18 1582.68 1646.65 1817.89 1886.25 1957.04

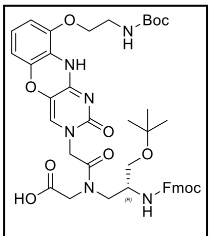

O
N
H
Boc
NH
N
O
N
O
O
O
O
HO
N
N
H
Fmoc

11

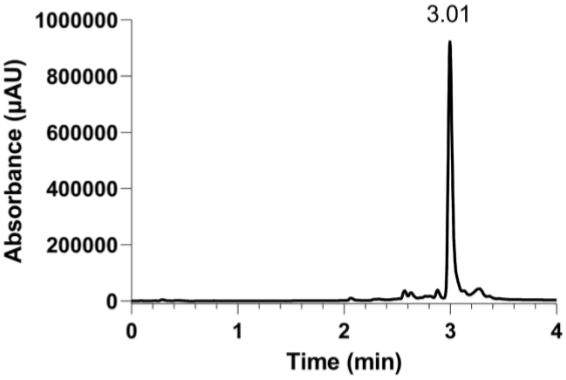

3.01
Absorbance (μAU)
1000000
800000
600000
400000
200000
0
0
1
2
3
4
Time (min)

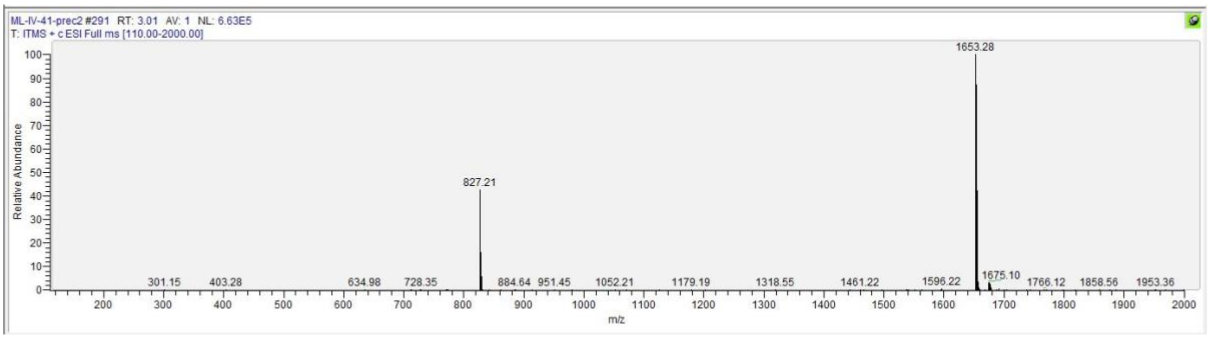

ML-IV-41-prec2 #291 RT: 3.01 AV: 1 NL: 6.63E5
T: ITMS + c ESI Full ms [110.00-2000.00]
Relative Abundance
m/z
1653.28
827.21
301.15
403.28
634.98
728.35
884.64
951.45
1052.21
1179.19
1318.55
1461.22
1596.22
1675.10
1766.12
1858.56
1953.36

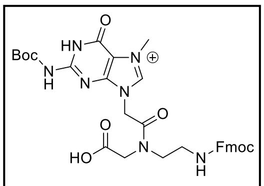

Boc
N
H
HN
O
N
N
N
⊕
O
O
HO
N
N
H
Fmoc

13

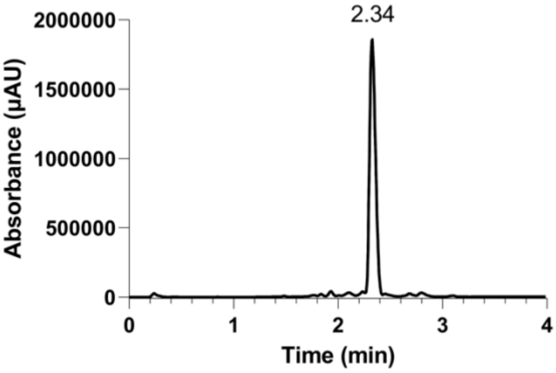

2.34
Absorbance (μAU)
2000000
1500000
1000000
500000
0
0
1
2
3
4
Time (min)

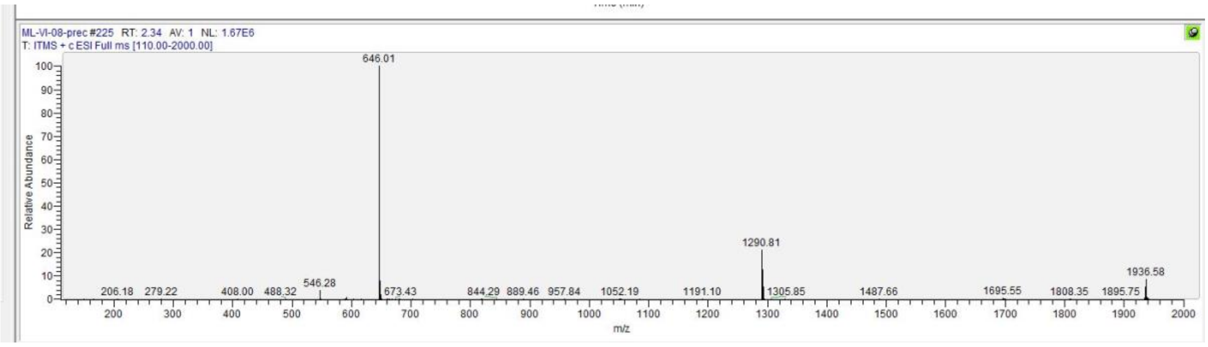

ML-VI-08-prec #225 RT: 2.34 AV: 1 NL: 1.67E6
T: ITMS + c ESI Full ms [110.00-2000.00]
Relative Abundance
m/z
646.01
1290.81
1936.58
206.18
279.22
408.00
488.32
546.28
673.43
844.29
889.46
957.84
1052.19
1191.10
1305.85
1487.66
1695.55
1808.35
1895.75

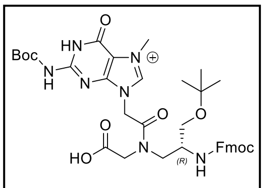
Boc
HN
O
N
H
N
N
N
⊕
O
O
HO
N
(R)
N
H
Fmoc

16

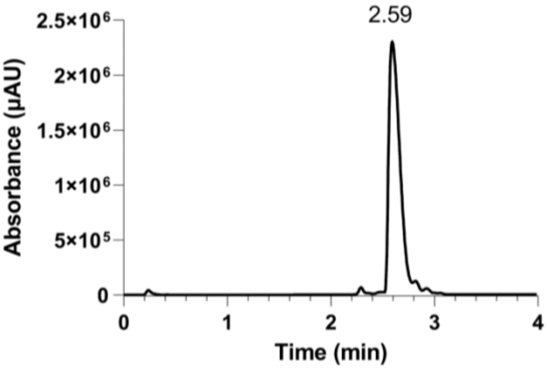
2.59
Absorbance (μAU)
Time (min)

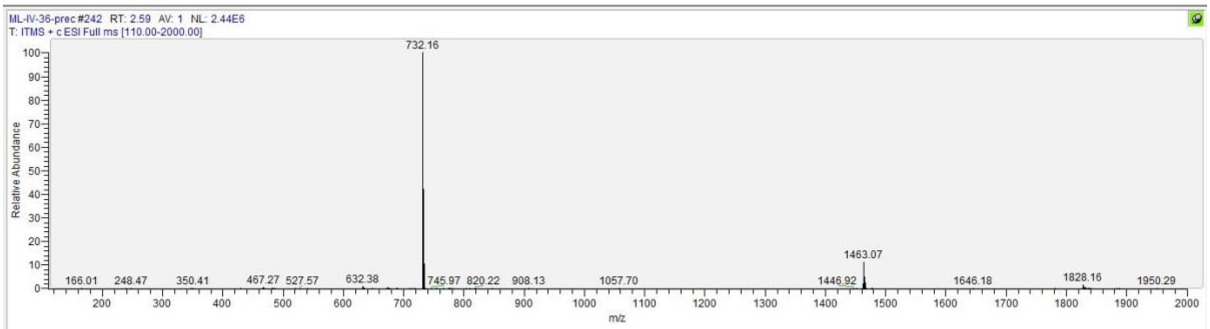
ML-IV-36-prec #242 RT: 2.59 AV: 1 NL: 2.44E6
T: ITMS + c ESI Full ms [110.00-2000.00]
732.16
1463.07
166.01
248.47
350.41
467.27
527.57
632.38
745.97
820.22
908.13
1057.70
1446.92
1646.18
1828.16
1950.29
Relative Abundance
m/z

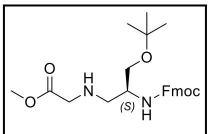
O
O
H
N
(S)
N
H
Fmoc

21

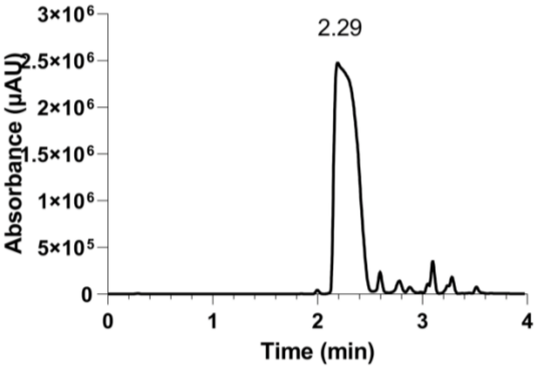
2.29
Absorbance (μAU)
Time (min)

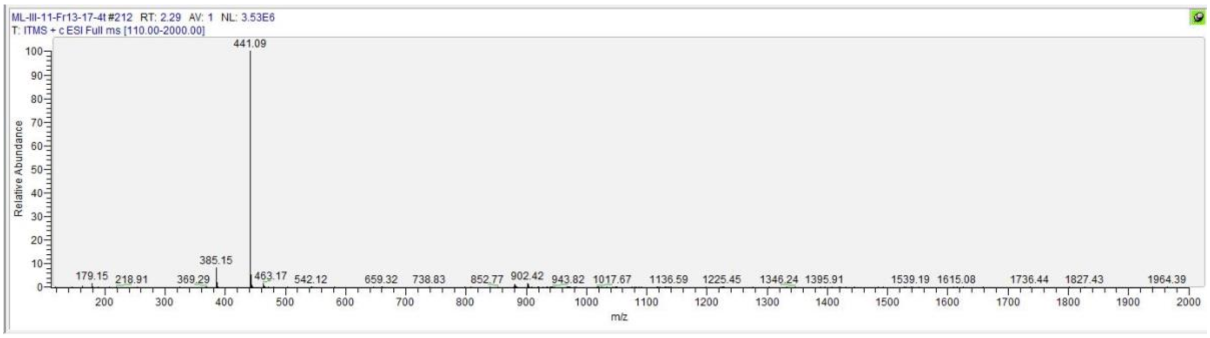
ML-III-11-Fr13-17-4t #212 RT: 2.29 AV: 1 NL: 3.53E6
T: ITMS + c ESI Full ms [110.00-2000.00]
441.09
385.15
179.15
218.91
369.29
463.17
542.12
659.32
738.83
852.77
902.42
943.82
1017.67
1136.59
1225.45
1346.24
1395.91
1539.19
1615.08
1736.44
1827.43
1964.39
Relative Abundance
m/z

SFC OD-H

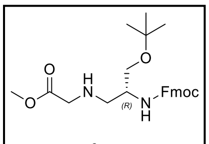

9

>99% ee

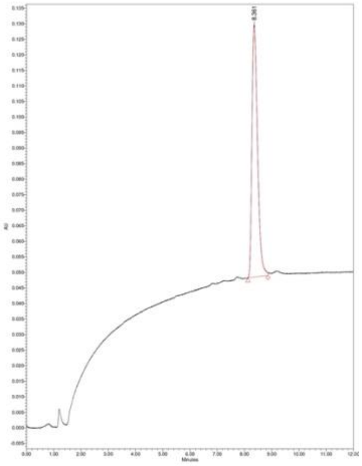

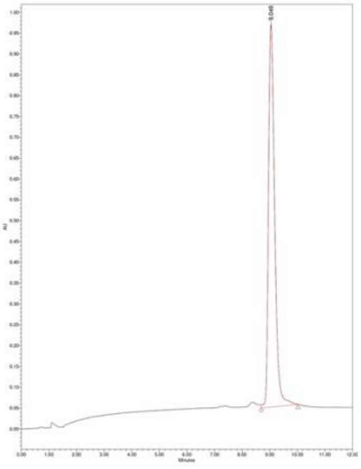

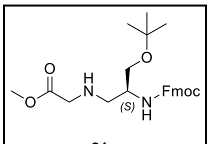

21

>99% ee

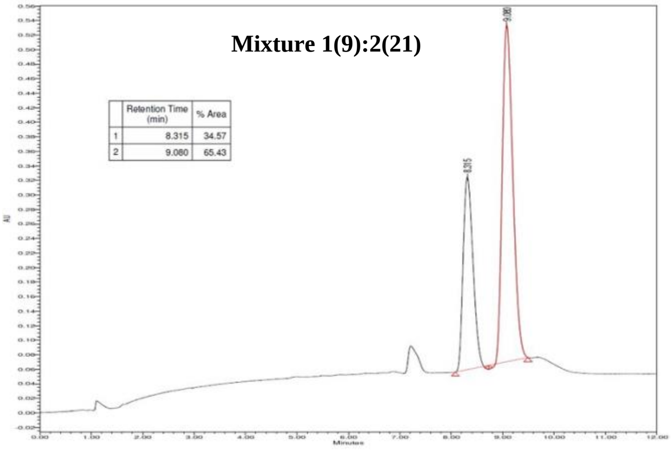

13.References

(1) Ausin, C.; Ortega, J. A.; Robles, J.; Grandas, A.; Pedroso, E. Synthesis of amino- and guanidino-G-clamp PNA monomers. *Org Lett* **2002**, *4* (23), 4073-4075,
(2) Debaene, F.; Da Silva, J. A.; Pianowski, Z.; Duran, F. J.; Winssinger, N. Expanding the scope of PNA-encoded libraries: divergent synthesis of libraries targeting cysteine, serine and metallo-proteases as well as tyrosine phosphatases. *Tetrahedron* **2007**, *63* (28), 6577-6586,
(3) Hibino, M.; Aiba, Y.; Shoji, O. Cationic guanine: positively charged nucleobase with improved DNA affinity inhibits self-duplex formation. *Chem Commun (Camb)* **2020**, *56* (17), 2546-2549,
(4) Bondebjerg, J.; Grunnet, M.; Jespersen, T.; Meldal, M. Solid-phase synthesis and biological activity of a thioether analogue of conotoxin G1. *Chembiochem* **2003**, *4* (2-3), 186-194. Avitabile, C.; Moggio, L.; Malgieri, G.; Capasso, D.; Di Gaetano, S.; Saviano, M.; Pedone, C.; Romanelli, A. gamma Sulphate PNA (PNA S): highly selective DNA binding molecule showing promising antigene activity. *PLoS One* **2012**, *7* (5), e35774,
(5) Song, Y.; Rodgers, V. G.; Schultz, J. S.; Liao, J. Protein interaction affinity determination by quantitative FRET technology. *Biotechnol Bioeng* **2012**, *109* (11), 2875-2883,
(6) Chakraborty, S.; Nunez, D.; Hu, S. Y.; Domingo, M. P.; Pardo, J.; Karmenyan, A.; Eva Ma, G.; Chiou, A. FRET based quantification and screening technology platform for the interactions of leukocyte function-associated antigen-1 (LFA-1) with intercellular adhesion molecule-1 (ICAM-1). *PLoS One* **2014**, *9* (7), e102572,
